# Novel noscapine derivative induces AML differentiation by dual-engaging DHODH and mitochondrial lipids to trigger a metabolic–epigenetic–transcriptional cascade

**DOI:** 10.1016/j.apsb.2026.05.019

**Published:** 2026-05-22

**Authors:** Shuting Shen, Defeng Li, Youping Zhang, Shiwei Li, Lei Li, Wenhao Shen, Yujie Ma, Wenhao Zhang, Rong Tao, Wei Wang, Biao Jiang, Fang Bai, Chuanxu Liu, Yongqiang Zhang, Qianqian Yin

**Affiliations:** a Shanghai Institute for Advanced Immunochemical Studies, ShanghaiTech University, Shanghai 201210, China; b School of Life Science and Technology, ShanghaiTech University, Shanghai 201210, China; c Department of Lymphoma, Fudan University Shanghai Cancer Center, Shanghai 200032, China; d Shanghai Frontiers Science Center of Optogenetic Techniques for Cell Metabolism, Shanghai Key Laboratory of New Drug Design, and School of Pharmacy, East China University of Science and Technology, Shanghai 200237, China; e Department of Hematology, Xinhua Hospital, Shanghai Jiao Tong University School of Medicine, Shanghai 200092, China; f Department of Pharmacology and Toxicology and BIO5 Institute, University of Arizona, Tucson, AZ 85721, USA; g CAS Key Laboratory of Synthetic Chemistry of Natural Substances, Shanghai Institute of Organic Chemistry, Chinese Academy of Sciences, Shanghai 200032, China

**Keywords:** Acute myeloid leukemia, Differentiation therapy, Noscapine derivative, Novel DHODH inhibitor, Protein‒lipid dual engagement, *De novo* pyrimidine synthesis, EP300/CREBBP, Transcriptional reprogramming

## Abstract

Acute myeloid leukemia (AML) is a heterogeneous and devastating hematologic malignancy characterized by differentiation blockage and immature progenitor accumulation, positioning differentiation therapy as a promising therapeutic strategy. However, clinical success is largely confined to acute promyelocytic leukemia (APL) and isocitrate dehydrogenase (IDH)-mutated AML, leaving most AML subtypes with unmet needs. Herein, novel noscapine derivative ES428 is discovered that induces AML differentiation and exhibits potent anti-AML efficacy across diverse AML cell lines, primary patient samples, as well as cell line- and patient-derived xenograft models. Target deconvolution with combinatorial strategies identifies dihydroorotate dehydrogenase (DHODH), a rate-limiting enzyme in *de novo* pyrimidine synthesis, as the direct functional target. Integration of molecular dynamics simulations and comprehensive structure-activity relationship studies elucidates ES428’s unique mechanism *via* simultaneous engagement with DHODH and mitochondrial membrane lipids. This dual-engagement underpins ES428’s enhanced target engagement, efficacy, and selectivity in physiologically relevant mitochondrial membrane environment, potentially through stabilizing ES428-DHODH interaction *in situ* and facilitating ES428’s selective mitochondrial localization. Furthermore, ES428 triggers a mechanistic cascade linking decreased pyrimidine synthesis, reduced O-linked *N*-acetylglycosylation (O-GlcNAcylation), EP300/CREBBP catalytic inhibition, and transcriptional reprogramming. Our findings identify promising lead candidates, establish a novel DHODH-targeting strategy, and provide important mechanistic insights to advance differentiation therapies for myeloid malignancies.

## Graphical abstract

Novel noscapine derivative ES428 simultaneously engages DHODH and mitochondrial membrane lipids, and triggers a cascade that links pyrimidine metabolism, EP300/CREBBP acetyltransferase inhibition, and transcriptional reprogramming to induce AML differentiation.

**Figure:**
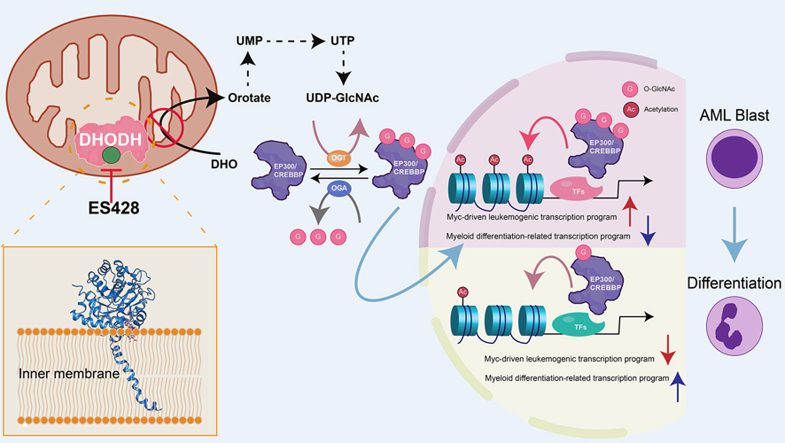

## 1 Introduction

Acute myeloid leukemia (AML) is a devastating hematologic malignancy characterized by uncontrolled proliferation, impaired differentiation, and accumulation of immature myeloid progenitors^1^. Current treatments predominantly rely on intensive combination chemotherapy and allogeneic stem cell transplantation^2^^,^^3^. However, the 5-year survival rate for adult AML patients remains unsatisfactory, with even poorer prognosis in elder patients^4^^,^^5^, underscoring the urgent need for novel treatments.

Despite genetic heterogeneity, differentiation arrest is a hallmark of AML and represents a vulnerability^6^. Differentiation therapy has shown remarkable success in acute promyelocytic leukemia (APL)^7^. All-*trans* retinoic acid (ATRA) and arsenic trioxide (ATO) have revolutionized APL treatment by targeting PML-RAR*α* fusion protein and promoting terminal maturation, transforming a fatal diagnosis into a curable disease^8^. However, the majority of non-APL AML subtypes remain resistant to ATRA-based therapies. Recent advances have expanded differentiation therapy to several emerging strategies, such as epigenetic (*e.g*., targeting DNA methyltransferase) and metabolic (*e.g.*, targeting mutant isocitrate dehydrogenase) therapies, with differentiation induction as a major mechanism for therapeutic efficacy^9^^,^^10^. Nevertheless, clinical success remains largely confined to APL (∼10% of AML cases) and IDH-mutated AML (∼20%), underscoring the unmet need for novel and effective differentiation therapies across the broader AML spectrum.

Dihydroorotate dehydrogenase (DHODH) catalyzes the rate-limiting step in *de novo* pyrimidine biosynthesis, bridging nucleotide metabolism with cellular bioenergetics and redox regulation^11^^,^^12^. The oncogenic relevance positions DHODH as a targetable metabolic dependency across diverse malignancies^13^. Hematologic malignancies, notably AML, have shown selective vulnerability to DHODH inhibition by impairing leukemic cell survival and overcoming differentiation arrest, stimulating significant interest in drug development and clinical translation^14^, ^15^, ^16^, ^17^, ^18^, ^19^, ^20^. Despite the preclinical promise, clinical translation has encountered significant challenges, including insufficient clinical benefit, potential toxicity concerns, and inadequate biomarkers for patient response^21^. From drug discovery perspective, DHODH’s unique localization within the inner mitochondrial membrane and its critical reliance on lipid‒protein interaction for biological function^22^, ^23^, ^24^ present underexplored opportunities, while the absence of structural elucidation within native mitochondrial membrane hinders rational drug design. Therefore, novel DHODH-targeted therapeutic strategies and comprehensive mechanistic understanding are required to advance DHODH-directed therapies in AML and beyond.

Natural products and derivatives remain indispensable in modern drug discovery due to their evolutionarily optimized structures targeting biological macromolecules, along with vast chemical diversity^25^. Over 50% of US Food and Drug Administration-approved small-molecule drugs trace natural origins, exemplified by vinblastine and paclitaxel revolutionizing cancer treatment^26^. Our previous work and others have expanded their application to AML differentiation therapy^27^^,^^28^, highlighting the significant potential to identify new bioactive entities and mechanisms. Traditional medicinal herb-derived natural products exhibit significant pharmacological potentials and safety profiles from centuries of use^29^, offering privileged scaffolds for therapeutic development. Noscapine, an alkaloid from opium poppy originally as a non-addictive antitussive drug since the mid-1950s^30^, exemplifies this potential. Its potential anticancer properties, favorable bioavailability, and low toxicity have repositioned it as a promising chemotherapeutic framework^31^.

Here, we developed a focused library of medicinal plant-derived natural products and derivatives, followed by phenotypic screening to identify novel AML differentiation therapeutics. A novel noscapine derivative ES428 was identified that demonstrated robust differentiation-inducing activity and potent anti-AML efficacy across diverse AML cell lines, primary patient samples, and xenograft models. Mechanistic studies identified ES428 as a novel DHODH inhibitor that simultaneously engages DHODH and mitochondrial membrane lipids. This dual-engagement represents a unique DHODH-targeting paradigm that underpins ES428’s superior target engagement, efficacy, and selectivity in physiological settings, potentially by stabilizing ES428–DHODH interaction *in situ* and promoting ES428’s selective mitochondrial localization. Using ES428 as a probe, we further delineated a mechanistic cascade linking pyrimidine metabolism, post-translational O-linked *N*-acetylglycosylation (*O*-GlcNAcylation), EP300/CREBBP acetyltransferase activity, and transcriptional remodeling. Our findings provide promising lead candidates and offer novel mechanistic and translational insights to broaden differentiation therapies for myeloid malignancies.

## 2 Materials and methods

A detailed description of the materials and methods for cell proliferation assays, cell differentiation assays, cell cycle analysis, cell apoptosis assay, plasmid construction and cell transfection, quantitative real-time PCR, Western blotting, colocalization imaging, recombinant DHODH expression and purification, DHODH enzymatic assays, surface plasmon resonance assay, TSA and CETSA assays, pulldown assay, immunoprecipitation, protein structure preparation and molecular docking, molecular dynamics simulations, target fishing, RNA-seq analysis, metabolic profiling analysis and chemical synthesis with NMR spectra can be found in the Supporting Information materials and methods, and in Supporting Data 1 and Data 2.

### 2.1 Reagents and antibodies

Uridine (Sigma‒Aldrich, U3003), UDP-GlcNAc (Sigma‒Aldrich, U4375), ATRA (Sigma‒Aldrich, R2625), Vitamin D3 (MedChemExpress, HY-15398), UMP (MedChemExpress, HY-N8060), Antimycin A (Sigma‒Aldrich, A8674), Orotic acid (Sigma‒Aldrich, O8402), l-Dihydroorotic acid (Sigma‒Aldrich, D7128), Riboflavin phosphate sodium (Sigma‒Aldrich, F2253), Brequinar (MedChemExpress, HY-108325), A-485 (TargetMol, T14073), CTPB (Active Motif, 14065), Decylubiquinone (Cayman, 21027). CD11b-APC antibody (ICRF44) (Thermo Fisher Scientific Cat# 17-0118-42, RRID: AB_2016659), CD14 APC antibody (Thermo Fisher Scientific Cat# 17-0149-42, RRID: AB_10669167), CD45-APC antibody (Thermo Fisher Scientific Cat# MHCD4505, RRID: AB_10372216), DHODH antibody (Santa Cruz Biotechnology Cat# sc-166348, RRID: AB_2091729), O-GlcNAc antibody (Abcam Cat# ab2739, RRID: AB_303264), mGPDH antibody (Abcam Cat# ab188585, RRID: AB_3068580), *β*-Actin antibody (Cell Signaling Technology Cat# 4967, RRID: AB_330288), H3 antibody (Cell Signaling Technology Cat# 4499, RRID: AB_10544537), H3K18Ac antibody (Cell Signaling Technology Cat# 9675, RRID: AB_331550), H3K27Ac antibody (Cell Signaling Technology Cat# 8173, RRID: AB_10949503), c-Myc antibody (Cell Signaling Technology Cat# 5605, RRID: AB_1903938), GAPDH antibody (ABclonal Cat# A19056, RRID: AB_2862549), *β*-Tubulin antibody (ABclonal Cat# A12289, RRID: AB_2861647), Rabbit Control IgG (ABclonal Cat# AC005, RRID: AB_2771930), DDDDK-Tag Rabbit mAb (ABclonal Cat# AE063, RRID: AB_2771920). MitoTox™ Complex II + III OXPHOS Activity assay (Abcam, ab109905).

### 2.2 Cell lines and cell culture

The human AML cell lines U937 (RRID: CVCL_0007), NB4 (RRID: CVCL_0005), HL-60 (RRID: CVCL_0002), Kasumi-1 (RRID: CVCL_0589), THP-1 (RRID: CVCL_0006), and OCI-AML3 (RRID: CVCL_1844), as well as human embryonic kidney cell line HEK-293T (RRID: CVCL_0063) were obtained from the American Type Culture Collection (ATCC) during 2019‒2020. All cell lines were authenticated by short tandem repeat (STR) profiling immediately after acquisition and prior to experimental use. Mycoplasma contamination was routinely monitored using the MeilunBio PCR Detection Kit (MA0349) at 2-month intervals, with all tests yielding negative results. No bacterial or fungal contamination was detected during culture. U937, NB4, NB4-LR1 (RRID: CVCL_8811) and NB4-LR2 (RRID: CVCL_U081) cells were cultured in RPMI 1640 medium (Gibco, Thermo Fisher Scientific, cat. no. 21875091) supplemented with 10% (*v*/*v*) FBS (Gibco, Thermo Fisher Scientific, cat. no. 10500064) and 1% (*v*/*v*) penicillin–streptomycin (Gibco, Thermo Fisher Scientific, cat. no. 15070063). HL-60 and Kasumi-1 cells were cultured in RPMI 1640 medium supplemented with 20% (*v*/*v*) FBS and 1% (*v*/*v*) penicillin–streptomycin. THP-1 cells were cultured in RPMI 1640 medium supplemented with 10% (*v*/*v*) FBS, 1% (*v*/*v*) penicillin–streptomycin, and 50 μmol/L 2-mercaptoethanol. OCI-AML3 cells were cultured in IMDM medium (Gibco) supplemented with 15% (*v*/*v*) FBS, 1% (*v*/*v*) penicillin–streptomycin. HEK-293T cells were cultured in DMEM medium (Meilunbio, MA0212) supplemented with 10% (*v*/*v*) FBS, 1% (*v*/*v*) penicillin–streptomycin. PBMCs from healthy adult donors were purchased from Shanghai Miao-tong Biological Technology (PB009C-1, HemaCare). Cell lines were maintained in a humidified incubator at 37 °C and 5% CO_2_.

### 2.3 Animals experiments

4–6-week-old male NCG mice (Strain NO. T001475) were purchased from the GemPharmatech Co., Ltd. (Nanjing, China) and housed in a specific pathogen-free facility with controlled temperature and humidity under a 12-h light/dark cycle.

For establishment and analysis of xenograft murine models, U937 cells stably transfected with luciferase reporter were intravenously (i.v.) injected into highly immune-deficient NCG mice. Two days post-transplantation, mice were grouped and treated with vehicle or ES428 (30 mg/kg) daily following a 2-week intermittent schedule (5-day-on/2-day-off per week) by i.v. administration. Drugs were formulated in DMSO/20% 2-hydroxylpropyl-*β*-cyclodextrin/Saline (1:1:8, *v*/*v*). Leukemic burden was monitored through bioluminescence following i.v. injection of d-luciferin at 150 mg/kg (cat.122799, PerkinElmer Envision, California). Mice were humanely euthanized by CO_2_ inhalation when the disease signs occurred, such as hunched back, loose fur, decreased physical activity, or apparent weight loss (>15%). Spleens were harvested and homogenized into a single-cell suspension in PBS with 2% FBS. Bone marrow cells were flushed from femurs and tibias with PBS. The leukemic-infiltrated tissue (*e.g*., liver) were minced and digested with an enzyme mixture of collagenase-V (1 mg/mL, cat#C9263, Sigma‒Aldrich) and DNase-I (1 mg/mL, 10104159001, Sigma‒Aldrich) at 37 °C. Red blood cells were lysed in Red Blood Cell Lysis Solution (cat# B541001, Sangon Biotech, Shanghai), and cell suspensions were filtrated through a 70 μm strainer to remove cell aggregates. Leukemic engraftment and differentiation assessment were analyzed by flow cytometry using human specific CD45 or CD11b antibody and H&E staining.

U937 cells overexpressing DHODH or NC control (5 × 10^6^) were implanted subcutaneously in a Matrigel matrix under the right flank of each mouse and allowed to grow to the pre-specified size of 100 mm^3^. Mice were treated with vehicle or ES428 *via* i.v. injection. Tumor size was measured utilizing electronic calipers every day during the treatment period. Tumor-bearing mice were euthanized by CO_2_ inhalation conformed to the following termination criteria: when the tumor volume reached 1500 mm^3^, or the tumor size exceeded 1.5 cm in diameter, or when the animals became moribund with severe weight loss (>15%), or mice showed signs of distress.

Primary AML mononuclear cells were isolated by Ficoll density centrifugation and were injected intravenously into NCG mice. Disease onset was defined as mentioned above. Leukemic cells from spleen or BM were serially passaged (i.v.; 2 × 10^6^ cells/mouse) into secondary NCG recipients. Seven days post-engraftment, mice received treatment following the same dosing regimen in U937 xenograft. Engraftment and differentiation were assessed as described above.

All procedures were approved by the Institutional Animal Care and Use Committee of ShanghaiTech University (approval # 20201026001).

### 2.4 Human samples

Informed consent was obtained from all patients in accordance with the Declaration of Helsinki, and all manipulations were approved by the Medical Science Ethics Committee of Xinhua Hospital, Shanghai Jiao Tong University (permission No. XHEC-C-2017-171).

### 2.5 Statistical analysis

Data are presented as mean ± SD, with n denoting biological replicates. Cellular experiments included at least three independent replicates. Statistical tests were conducted using GraphPad Prism using two-tailed unpaired *t*-tests for two-group comparisons, one-way ANOVA for multiple-groups, and Log-rank test for survival curves. Significance was set at ∗*P* < 0.05, ∗∗*P* < 0.01, ∗∗∗*P* < 0.001.

## 3 Results

### 3.1 Phenotypic screening identified ES428 as a potent AML differentiation inducer

**Figure 1:**
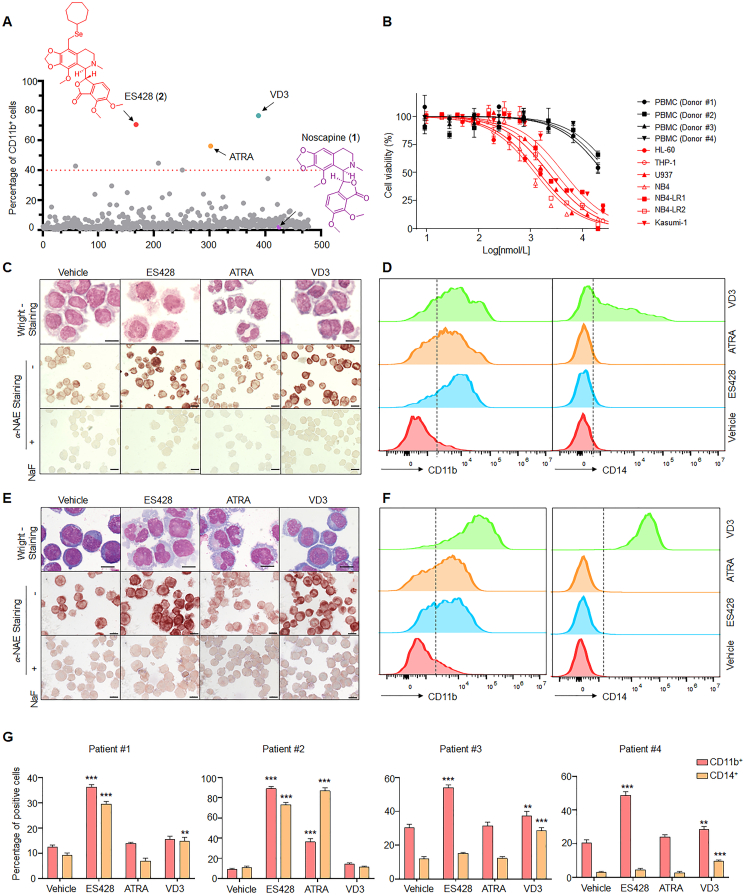
ES428 induces differentiation in AML cell lines and primary patient samples. (A) Graphical presentation of screening results of ∼500 compounds performed in U937 cells treated with an approximate IC_50_ concentration of each compound for 3 days. CD11b^+^ cells were measured by flow cytometry. Each dot represents one compound. Chemical structure of ES428 (red dot) and noscapine (purple dot) are shown. Yellow and green dots are indicative of control compounds ATRA and VD3, respectively. (B) Multiple cancer cell lines and PBMC cells from four healthy donors were treated with ES428 at different doses, and growth inhibition was evaluated by the CCK-8 assay. (C‒F) U937 cells (C and D) and THP-1 cells (E and F) were treated with ES428 at 2 μmol/L, ATRA at 1 μmol/L, or VD3 at 0.1 μmol/L for 3 days and the differentiation-inducing capacities were evaluated. (C and E) Wright’s staining morphology (top) and *α*-NAE staining in the absence (middle) or presence (bottom) of sodium fluoride (NaF). Scale bar, 20 μm. (D and F) Flow cytometry analysis of myeloid differentiation cell surface antigens CD11b and CD14 for treatment as indicated compounds. (G) The percentage of anti-human CD11b^+^ and CD14^+^ cells in four representative primary AML patient samples were measured by flow cytometry following treatment with compounds as indicated for 3 days. Error bars represent the mean ± SD of triplicate samples in an independent experiment. ∗*P* < 0.05, ∗∗*P* < 0.01 and ∗∗∗*P* < 0.001 against the vehicle group.

We prioritized the strategic derivatization of natural product scaffolds from traditional medicinal plants such as noscapine^30^ and curcumol^32^, and constructed an in-house library of ∼500 structurally diverse compounds to explore therapeutic opportunities for AML differentiation. Utilizing ITGAM/CD11b as a myeloid differentiation marker, phenotypic screening in U937 cells, cell line of the AML-M5 subtype according to the FAB (French–American–British) classification^33^, identified novel noscapine derivative ES428 (**2**) as the most potent compound (Fig. 1A). ATRA and 1,25(OH)_2_-vitamin D3 (VD3) served as controls. ES428’s cellular activity was further verified and it demonstrated broad anti-proliferative effects across diverse AML cell lines with selective cytotoxicity sparing normal peripheral blood mononuclear cells (PBMCs) (Fig. 1B), indicating a favorable therapeutic window. ES428 triggered dose-dependent inhibition of AML cell growth at non-cytotoxic concentrations, concomitant with S-phase cell cycle arrest (Supporting Information Fig. S1A‒S1C). Furthermore, ES428-induced differentiation was confirmed in U937 cells and THP-1 cells (FAB-M5 subtype) through Giemsa-Wright staining showing characteristic morphological features, including chromatin condensation and reduced nucleus-to-cytoplasm ratio (Fig. 1C and E, top). Additionally, cytochemical analysis using alpha-naphthyl butyrate esterase (*α*-NAE) staining (Fig. 1C and E, bottom) and flow cytometric analysis of myeloid differentiation markers CD11b and CD14 (Fig. 1D–F, and Supporting Information Fig. S2A and S2B) further corroborated ES428’s differentiation-inducing capacity. The ED_50_ (effective dose for 50% of the maximal differentiation activity) was 1‒2 μmol/L in U937 and THP-1 cells (Fig. S2C). Additionally, ES428 also induced differentiation across a range of AML cell lines, including HL-60 (M2 subtype, Fig. S2D and S2E), and OCI-AML3 cells (M4 subtype, Fig. S2F). Notably, differential sensitivity to differentiation induction was observed across different subtypes, with reduced responsiveness in Kasumi-1(M2b subtype with AML1-ETO fusion), NB4 (M3 subtype with PML-RAR*α* fusion), and ATRA-resistant NB4-R1 and NB4-R2 cells post-ES428 treatment (Fig. S2G and S2H). Together, ES428 induced AML differentiation accompanied by S-phase arrest across multiple AML cell lines, which differs from noscapine (**1**) and its well-known 9′-brominated analog (**3**) that triggered G2/M arrest *via* microtubule targeting^30^ and failed to promote AML differentiation (Supporting Information Fig. S3A–S3C). This finding suggests ES428’s novel mechanism responsible for AML differentiation distinct from microtubule targeting.

To extend the therapeutic potential of ES428, primary AML samples (mononuclear cells from bone marrow) from *de novo* and relapsed patients were assessed (Fig. 1G and Fig. S2I). Patient demographics are detailed in Supporting Information Table S1. ES428 induced differentiation in 56% (5/9) of the AML samples tested, with a pronounced response rate in the myelomonocytic and monocytic subtypes (FAB M4 and M5), where 80% (4/5) of cases exhibited sensitivity. Conversely, two non-responding samples, corresponding to M2b-AML-ETO^+^ and M3-PML-RAR*α*^+^ subtype, aligned with the observed insensitivity in Kasumi-1 and NB4 cell lines, respectively. Together, these results demonstrated ES428’s broad and potent differentiation-inducing activity across AML cell lines and primary patient samples, particularly in M4 and M5 subtypes.

### 3.2 ES428 induces myeloid differentiation and exhibits anti-AML efficacy in murine xenograft models

**Figure 2:**
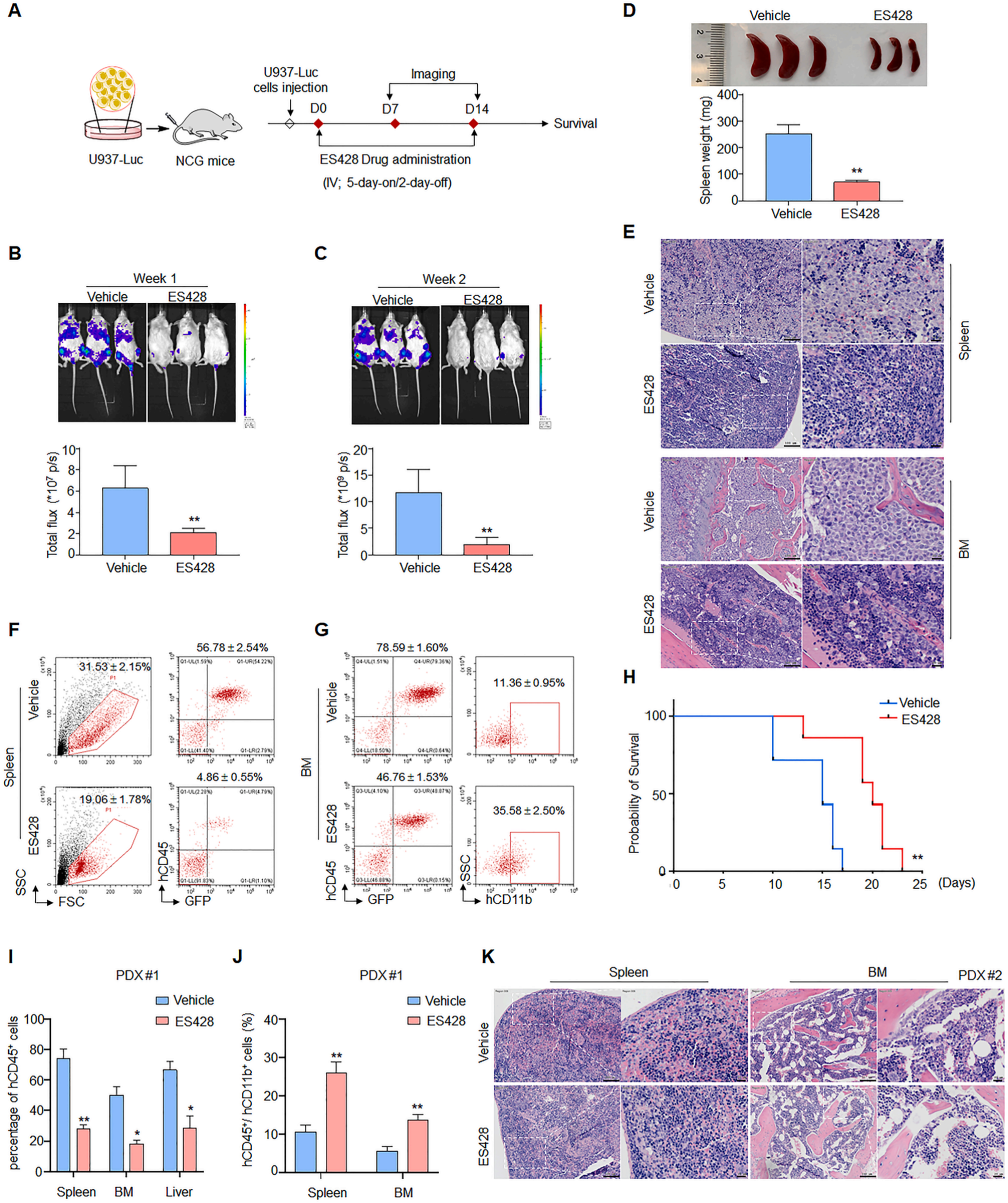
ES428 exhibits potent anti-AML efficacy and induces leukemic cell differentiation *in vivo*. (A) Schematic of the xenograft murine model. Luciferase-expressing U937 cells were injected i.v. in NCG mice. U937-Luc tumor-bearing mice were grouped and treated with vehicle and ES428 (30 mg/kg) daily following a 2-week intermittent schedule (5-day-on/2-day-off per week) by i.v. administration (*n* ≥ 6 for each group). Mice were humanely euthanized at the pre-defined endpoint (see the “Method”). (B,C) U937-Luc tumor-bearing mice of each group were analyzed for tumor growth through bioluminescence imaging at week 1 (B) and week 2 (C) post-treatment of ES428. Quantitative results were analyzed for each group and expressed as total photon values per second (Total Flux [p/s]). (D‒H) The mice were humanely euthanized at the pre-defined endpoint. The appearance (top) and weight (bottom) of spleen from vehicle or ES428-treated leukemic mice are shown in (D). The hematoxylin and eosin (H&E) staining of spleen (top) and bone marrow (bottom) of each group were shown in (E). Scale bar (left), 100 μm; scale bar (right, enlarged), 20 μm. The percentage of anti-human CD45^+^/GFP^+^ cells of the spleen (F) and bone marrow (G, left) of each group (*n* ≥ 3) was evaluated by flow cytometry analysis. The percentage of anti-human CD11b^+^ cells of the bone marrow (G, right) was evaluated by flow cytometry analysis. The survival (%) and lifetime (days) of xenograft mice in each group were recorded (*n* ≥ 6 for each group), and Kaplan-Meier survival analysis was shown (H). *P* value was determined by the log-rank test. (I‒K) The PDX mice were grouped post-inoculation and treated with vehicle or ES428 utilizing dosing schedule as described in (A). Flow cytometry analysis of percentage of human CD45^+^ cells in the spleen, BM and liver samples harvested from vehicle or ES428-treated PDX-#1 leukemic mice (*n* ≥ 3 for each group) in (I). The percentage of anti-human CD45^+^CD11b^+^ cells of the spleen and bone marrow samples derived from vehicle or ES428-treated PDX-#1 leukemic mice by flow cytometry analysis (J). (K) The H&E staining of spleen and bone marrow samples from vehicle or ES428-treated PDX-#2 mice were shown. Scale bars: 100 and 20 μm (enlarged). ∗*P* < 0.05, ∗∗*P* < 0.01 and ∗∗∗*P* < 0.001 against the vehicle-treated group.

We further investigated ES428’s pharmacokinetics, tolerability, and efficacy *in vivo*. Pharmacokinetic analysis revealed a favorable profile with a ∼7-h half-life and a single 2 mg/kg intravenous dose achieved plasma concentration (∼1 μmol/L) near the *in vitro* cellular ED_50_ (Supporting Information Fig. S4A and S2B). ES428 was well tolerated in NCG mice following intravenous administration at 30 mg/kg for two weeks, with no significant impact on body weight (Fig. S4C). U937 cells were stably transfected with a luciferase-GFP reporter construct, and then implanted intravenously into NCG mice to establish an AML xenograft model (Fig. 2A). ES428 treatment significantly reduced leukemic burden evidenced by bioluminescence imaging at both week 1 (Fig. 2B) and week 2 (Fig. 2C) post-treatment. Vehicle-treated mice developed typical AML features, including spleen enlargement and leukemic infiltration in multiple organs, While ES428 administration markedly reduced spleen size and weight (Fig. 2D), and decreased leukemic cell infiltration in the spleen and bone marrow (BM) through histological staining (Fig. 2E). Flow cytometry analysis further confirmed a significant reduction of infiltrating human CD45^+^/GFP^+^ leukemic cells in spleen, BM, and liver following ES428 administration (Fig. 2F, G and Fig. S4D). Importantly, ES428 induced myeloid differentiation *in vivo*, as evidenced by increased CD11b^+^ cells in BM of ES428-treated mice (Fig. 2G). Furthermore, ES428 treatment prolonged the survival of U937 xenograft mice (Fig. 2H). We next evaluated ES428’s efficacy in two patient-derived xenograft (PDX) models *in vivo*. In vehicle-treated PDX-#1 mice, significant infiltration of human CD45^+^ AML malignant cells was detected in the spleen, BM and liver *via* flow cytometry, which was markedly reduced following ES428 treatment (Fig. 2I). The differentiation-inducing effect of ES428 was confirmed by the increased presence of human CD11b^+^ cells in the spleen and BM (Fig. 2J). Histological analysis revealed that ES428 exhibited only modest inhibition of leukemic cell infiltration in spleen and BM compared to the vehicle group in PDX-#2 (Fig. 2K), a model exhibiting relatively low engraftment efficiency. Together, these findings demonstrated that ES428 induced myeloid differentiation and exerted therapeutic efficacy *in vivo*.

### 3.3 Multi-omics revealed ES428-induced differentiation associated with pyrimidine biosynthesis inhibition

**Figure 3:**
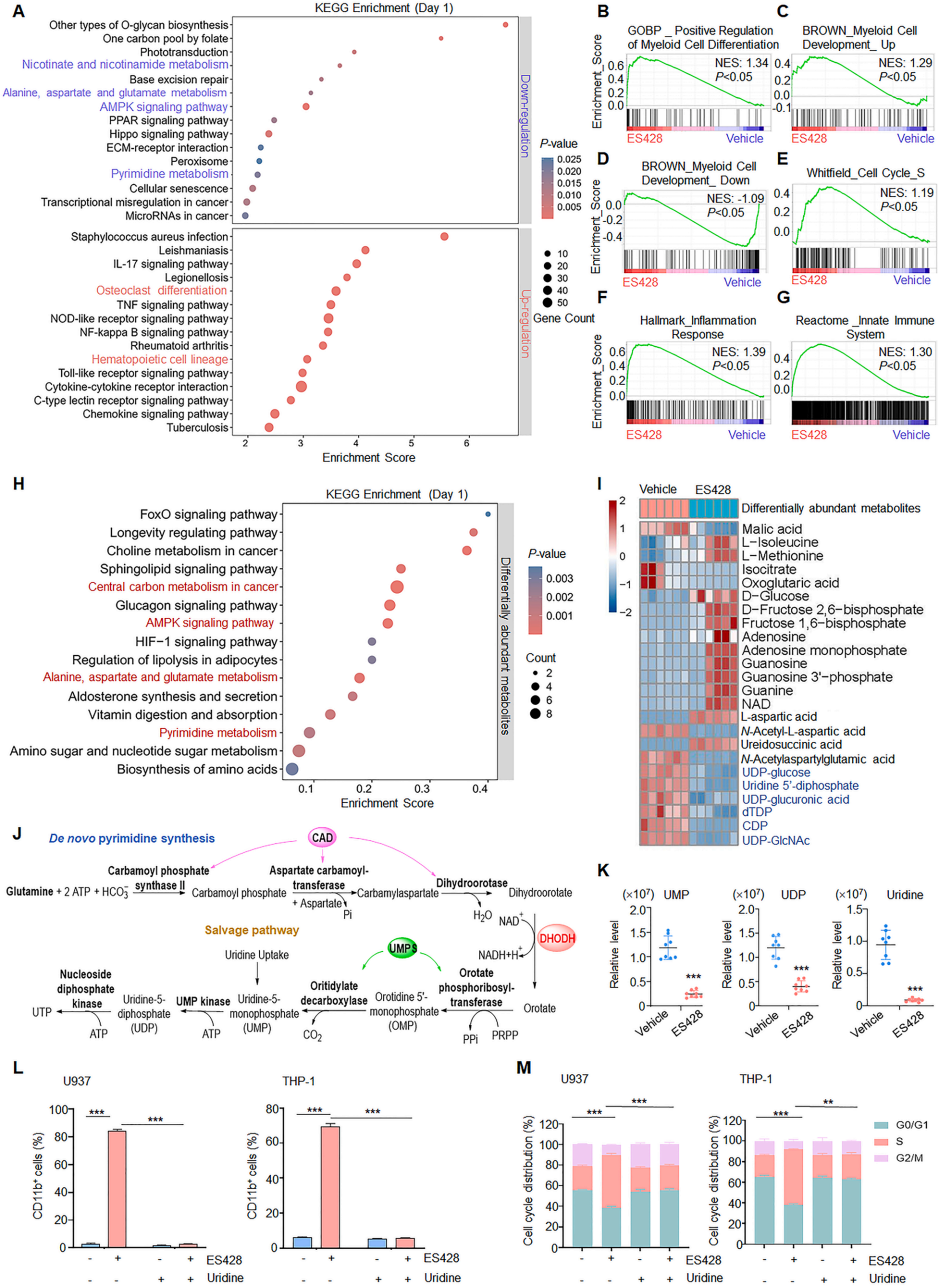
The anti-AML efficacy of ES428 is closely associated with inhibition of pyrimidine biosynthesis. (A) KEGG analysis for the top 15 enriched pathways among downregulated (top) and upregulated genes (bottom) following ES428 treatment at early time point (Day 1). (B‒G) GSEA analysis of transcriptional profiling upon ES428 treatment at Day 1. Enrichment plots of gene sets for “GOBP_Positive Regulation of Myeloid Cell Differentiation” (B), “BROWN_Myeloid Cell Development_Up” (C), “BROWN_Myeloid Cell Development_Down” (D) “Whitfield_Cell Cycle_S” (E), “Hallmark_Inflammation Response” (F), and “Reactome_Innate Immune System” (G) were shown. NES, normalized enrichment score. (H) KEGG analysis for the top 15 enriched pathways of differentially abundant metabolites following ES428 treatment at Day 1. (I) Heatmap of expression profile of differentially abundant metabolites following ES428 treatment at different time points that are related to enriched metabolic pathways highlighted with red in (H). Metabolites related to pyrimidine biosynthesis pathway were highlighted with blue. (J) Schematic of the *de novo* pyrimidine synthesis and the salvage pathway. Multifunctional enzyme CAD: carbamoyl-phosphate synthetase II/aspartate transcarbamylase/dihydroorotase; Bifunctional enzyme UMPS: 5′-monophosphate synthase. (K) The levels of intracellular UMP, UDP and uridine were measured by LC–MS/MS analysis upon ES428 treatment for 24 h in U937 cells (*n* = 8). (L, M) U937 and THP-1 cells were incubated with ES428 at 2 μmol/L in the presence or absence of excess uridine of 100 μmol/L for 3 days. The differentiation-inducing efficacy was evaluated by anti-human CD11b staining and FACS analysis (L). Percentages of cell populations in G0/G1, G2/M, and S-phases of were shown in (M). Results in (K‒M) are representative of at least three independent experiments. ∗*P* < 0.05, ∗∗*P* < 0.01 and ∗∗∗*P* < 0.001 against the vehicle group and between the line-pointed group.

To elucidate ES428’s mechanism of action, RNA sequencing was conducted on U937 cells post-ES428 treatment at different time points (Supporting Information Fig. S5A). KEGG analysis revealed consistent upregulation of hematopoietic lineage and osteoclast differentiation at both early (Day 1, Fig. 3A) and later time points (Days 3 and 5, Fig. S5B and S5C), aligning with the ES428-induced myeloid differentiation. Correspondingly, pathways related to myeloid cell maturation and functions, such as innate immune and inflammation response, were also enriched. Consistently, the profound phenotypic relevance was further validated by Gene set enrichment analysis (GSEA) (Fig. 3B‒G). Intriguingly, KEGG analysis of downregulated genes showed significant enrichment of several metabolic pathways at early time point (blue highlight, Fig. 3A), implicating potential involvement of metabolic alterations. Subsequent non-targeted metabolomic profiling was performed (Supporting Information Fig. S6A), and KEGG analysis revealed significant enrichment of multiple metabolic pathways at both early (Fig. 3H) and later time points (Fig. S6B and S6C) post-ES428 treatment. Both transcriptomic and metabolomic profiling revealed consistent and sustained enrichment of pyrimidine metabolism, accompanied by significant decrease of associated metabolites following ES428 treatment (Fig. 3I and Fig. S6D and S6E). These findings suggest that ES428 disrupts pyrimidine biosynthesis. Pyrimidine biosynthesis provides essential precursors for biological macromolecules such as nucleic acids and glycoproteins^34^, which occurs *via* two principal routes: the *de novo* synthesis and salvage pathway (Fig. 3J). The *de novo* UMP synthesis involves six reactions catalyzed by three key enzymes, while salvage pathways utilize extracellular uridine converted to UMP. HPLC‒MS/MS analysis confirmed significant depletion of intracellular UMP, UDP, and uridine levels (Fig. 3K). Phenotypically, excess uridine supplementation completely abrogated ES428-induced differentiation (Fig. 3L) and S-phase arrest (Fig. 3M) in U937 and THP-1 cells. These findings supported pyrimidine biosynthesis inhibition as the primary mechanism underlying ES428’s anti-AML efficacy.

### 3.4 DHODH is identified as ES428’s direct functional target

**Figure 4:**
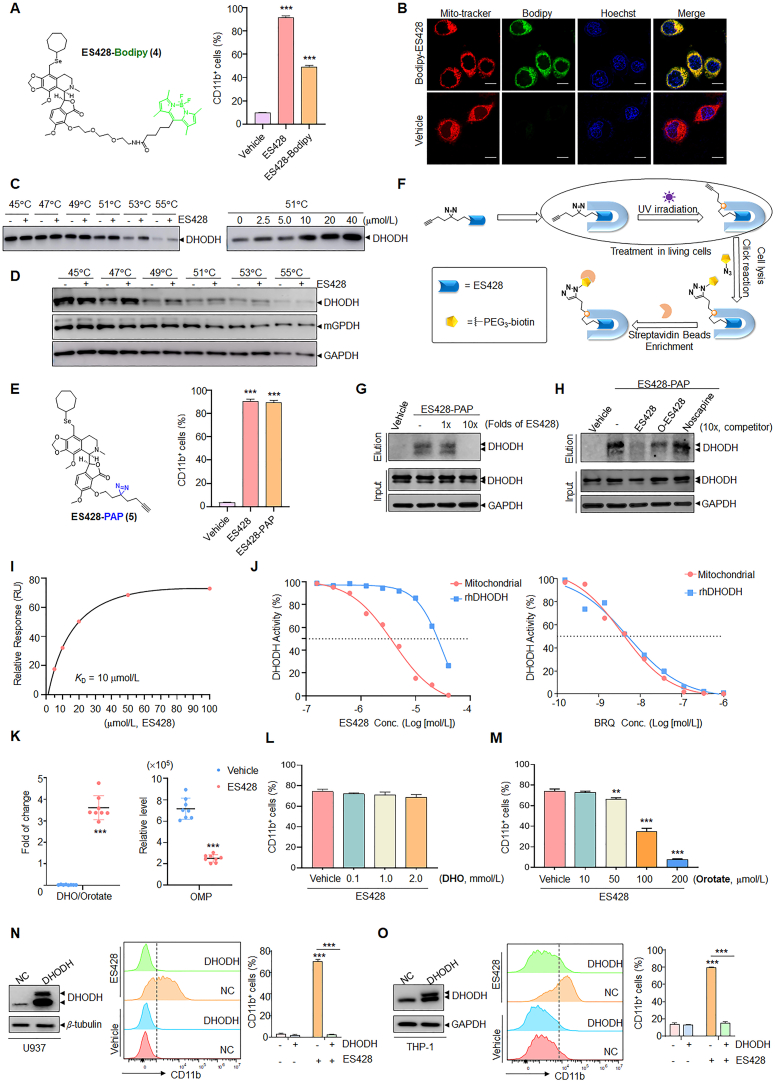
DHODH is identified as the direct functional target responsible for ES428’s anti-AML efficacy. (A) Chemical structure (left) and differentiation-inducing activity of Bodipy-ES428 in U937 cells treated with indicated compounds for 3 days, assessed by flow cytometry analysis of CD11b surface marker expression (right). (B) Co-localization images of Bodipy-ES428 and Mito-tracker. Red fluorescence indicates the mitochondria, green fluorescence indicates the Bodipy-ES428 probe, Blue fluorescence indicates the nucleus, and the merged image shows overlapping signals. (C) The purified recombinant DHODH protein with a 30-residue N-terminal deletion was incubated with or without ES428 for 30 min. The samples were heat-treated at different temperatures as indicated in (C, left) or at constant temperature across different concentrations as indicated in (C, right) for 3 min. The remaining protein in the supernatant was detected by Western blotting utilizing specific antibody against DHODH. (D) The in-cell target engagement of ES428 in intact cells using CETSA. The U937 cells were treated with or without ES428 for 12 h prior to heating at different temperatures as indicated. Cells were then lysed and the remaining protein in the supernatants were detected by western blotting. GAPDH was detected as loading control and mGPDH was used here as a negative control. (E) Chemical structure of ES428-PAP (the photoaffinity probe). U937 cells were treated with ES428 or ES428-PAP at equivalent dose of 2 μmol/L for 3 days and the percentage of anti-human CD11b^+^ cells were measured by flow cytometry. (F) Schematic workflow of pulldown assays based on ES428-PAP. (G) U937 cells were pre-treated with or without ES428 at different concentrations as indicated (competition) for 2 h following incubation with ES428-PAP for 6 h. The streptavidin beads-based pull down was performed and the interacting complex were eluted and detected by western blotting. Input panels were utilized for normalization. (H) U937 cells were pre-treated with different compounds as indicated at equivalent concentration (10 × , competition) for 2 h following incubation with ES428-PAP for 6 h. Drug-target enrichment assay was performed as described in (G). (I) A concentration series of ES428 was applied for binding through SPR-based interaction analyses. The corresponding plot of steady-state binding data from the end of the association phases against analyte concentrations were used to calculate the steady-state affinity (*K*_D_). (J) The inhibitory effect of ES428 (left) and reference compound BRQ (right) using a kinetic DHODH enzymatic assay *in vitro* based on rhDHODH or mitochondrial isolation derived from AML cells as the enzyme source. (K) The levels of intracellular DHO, Orotate and OMP were measured by LC‒MS/MS analysis following ES428 treatment for 24 h in U937 cells. (L, M) U937 cells were incubated with ES428 of 2 μmol/L in the presence or absence of DHO (L) and Orotate (M) as indicated for 3 days. The differentiation-inducing activity was evaluated by anti-human CD11b staining and FACS analysis. (N, O) U937 cells (N) and THP-1 cells (O) stably overexpressing DHODH were treated with ES428 for 3 days and the differentiation-inducing activity was evaluated by anti-human CD11b staining and FACS analysis. The protein level of DHODH overexpression was detected through western blotting. Results are representative of at least three independent experiments. Error bars represent the mean ± SD of triplicate samples in an independent experiment. ∗*P* < 0.05, ∗∗*P* < 0.01 and ∗∗∗*P* < 0.001 against the vehicle-treated or between the line-pointed group.

We further integrated computational and experimental approaches to identify ES428’s target acting on pyrimidine biosynthesis. MAI-Target Fisher, an in-house computational model, was utilized for genome-wide target prediction. Given that selenium atom is not supported by the force field for docking, we designed sulfur-(S-ES428) and oxygen-substituted (O-ES428) analogs (Supporting Information Fig. S7A). S-ES428 preserved differentiation-inducing activity with a slight reduction (Fig. S7B), indicating the identical target as ES428. In contrast, O-ES428 significantly lost activity. Using S-ES428 as a tool compound, target fishing identified 17 candidates with Glide docking scores below −8.0, and DHODH emerged as the top-ranking (Fig. S7C). Furthermore, we synthesized a Bodipy-labeled fluorescent probe (Bodipy-ES428, **4**) by attaching at the 7-position, which maintained differentiation-inducing capacity (Fig. 4A and Supporting Information Fig. S8A). Intriguingly, live-cell imaging using Bodipy-ES428 alongside cell-permeant mitochondria-labeling dye MitoTracker revealed strong co-localization with mitochondria (Fig. 4B). This finding indicated ES428’s intrinsic mitochondrial-targeting feature, distinct from engineered strategies conjugating with lipophilic cations such as triphenylphosphonium (TPP)^35^. This further implied potential interactions of ES428 with mitochondrial protein. DHODH is the sole mitochondrial enzyme in pyrimidine biosynthesis that catalyzes the rate-limiting FMN-dependent oxidation of dihydroorotate (DHO) to orotate *via* coenzyme Q (CoQ, ubiquinone)-mediated electron transfer. Consequently, DHODH was prioritized for further validation as direct target.

Thermal shift assay (TSA) demonstrated that ES428 increased the thermal stability of recombinant human DHODH (rhDHODH) protein (Fig. 4C). Cellular thermal shift assay (CETSA) further confirmed target engagement in intact cells (Fig. 4D). Due to the absence of covalent reactive moieties in ES428, we designed a photoaffinity probe (ES428-PAP, **5**) incorporating diazirine moiety and alkyne handle at the 7-position to enable covalent capture of potential target (Fig. S8B). ES428-PAP preserved differentiation-inducing activity comparable to ES428 (Fig. 4E). *In situ* photoaffinity labeling followed by pulldown assays (Fig. 4F) revealed significant enrichment of DHODH by ES428-PAP, which was effectively competed by excess ES428 but not by inactive O-ES428 analog or noscapine (Fig. 4G and H). Surface plasmon resonance (SPR) assay determined ES428’s binding affinity to DHODH with a *K*_D_ of ∼10 μmol/L *via* non-linear regression of steady-state binding responses (Fig. 4I and Fig. S8C). ES428 treatment did not affect DHODH protein abundance in AML cells, as shown in Fig. S8D. We further evaluated whether target engagement translated to enzymatic inhibition (Fig. 4J). *In vitro* kinetic enzymatic assays with rhDHODH showed relatively weak inhibition with an EC_50_ of ∼17 μmol/L, inconsistent with ES428’s cellular differentiation-inducing potency (ED_50_ of 1‒2 μmol/L). Notably, when utilizing isolated mitochondrial fractions from AML cells as the enzyme source, ES428 exhibited enhanced inhibitory potency (EC_50_ 3‒4 μmol/L), closely matching cellular potency. Conversely, brequinar (BRQ), a well-known DHODH inhibitor, exhibited consistent inhibitory potency across both assays. These findings indicated that ES428’s efficacy against DHODH depends on the native mitochondrial context. Moreover, metabolite profiling confirmed that ES428 inhibited cellular DHODH catalytic activity, as evidenced by elevated DHO-to-orotate ratio and decreased downstream OMP level (Fig. 4K). Functionally, supplementation with the product orotate or downstream metabolites (OMP, UMP), but not substrate DHO, significantly reversed ES428-induced differentiation in U937 and THP-1 cells (Fig. 4L, M and Fig. S8E and S8F). Consistently, DHODH overexpression completely abolished ES428-induced AML differentiation in U937 and THP-1 cells (Fig. 4N and O), while DHODH knockout triggered spontaneous cell differentiation and reduced susceptibility to ES428 induction (Fig. S8G‒J), underscoring DHODH as the functional target of ES428 for differentiation induction. *In vivo*, ES428 significantly inhibited tumor growth without affecting body weight in U937 subcutaneous xenograft model (Supporting Information Fig. S9A and S9B). Furthermore, ES428 treatment promoted AML cell differentiation *in vivo*, as evidenced by increased CD11b expression (Fig. S9C and S9D). By contrast, tumors derived from DHODH-overexpressing U937 cells exhibited enhanced growth while demonstrated reduced sensitivity to ES428-induced tumor growth arrest and AML cell differentiation relative to controls. Collectively, these findings strongly supported DHODH as ES428’s direct functional target underlying its anti-AML efficacy. Additionally, optimized molecular docking study was conducted to define ES428’s binding pocket in DHODH by utilizing a modeled full-length human DHODH structure developed through homology modeling integrating AlphaFold2 predictions^36^ with available truncated DHODH crystal structure (PDB: 4IGH, residues 32‒395) as a template. The docking result revealed ES428’s preferential binding to the CoQ binding pocket as indicated by lower binding free energy (Supporting Information Fig. S10A and B), which was further validated by reversal of ES428-induced differentiation using membrane-permeable CoQ derivatives (Fig. S10C‒E). Despite functional coupling of DHODH and mitochondrial complex III *via* CoQ redox-cycling, ES428 didn’t inhibit complex III activity in enzymatic assay *in vitro* using isolated mitochondria fraction (Supporting Information Fig. S11), further underscoring its selectivity for DHODH-targeting.

### 3.5 ES428 exerts a unique mechanism of action through dual engagement of DHODH and mitochondrial membrane lipids

**Figure 5:**
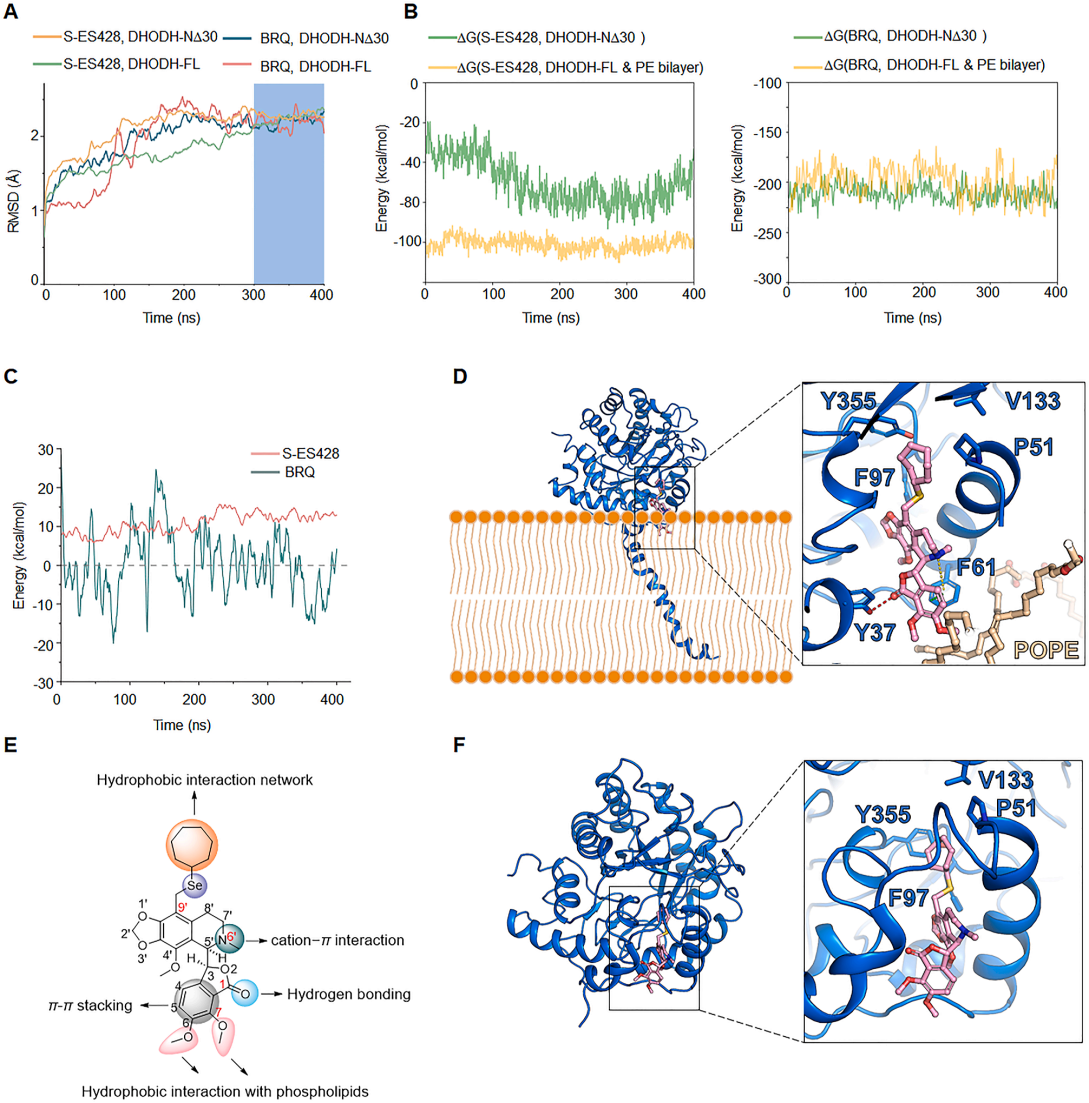
Molecular dynamics simulations reveal S-ES428’s unique binding mechanism *via* dual engagement of DHODH and mitochondrial lipids. (A) RMSD curves of the molecular dynamics simulations trajectory analysis of truncated DHODH (DHODH-NΔ30) with S-ES428 or BRQ, full-length DHODH (DHODH-FL) with S-ES428 or BRQ from the beginning to the end of 400 ns. The stable regions were highlighted in blue on the RMSD curve. (B) Binding free energy calculations (Δ*G*_Bind_) were performed for S-ES428 or BRQ during the trajectory simulations comparing the two scenarios of full-length DHODH on a PE bilayer *versus* DHODH-NΔ30 without a PE bilayer. (C) The change of interaction energies of S-ES428 or BRQ was calculated over the course of molecular dynamics simulations, comparing the lipid-bound full-length DHODH complex to the full-length DHODH protein alone. (D) Clustered interaction pattern of S-ES428 with lipid-bound full-length DHODH complexes over the last 100 ns of trajectory. The protein residues surrounding the ligand S-ES428 constituted the interacting sites. The cation‒*π* interaction was shown as yellow dashed lines, the *π‒π* stacking was shown as green dashed lines, and the hydrogen bond was shown as red dashed lines. (E) Schematic of binding sites of S-ES428 with DHODH in the presence of PE bilayer. (F) Clustered interaction pattern of S-ES428 with DHODH-NΔ30 truncation without PE bilayer over the last 100 ns of trajectory. The protein residues surrounding the ligand S-ES428 constituted the interacting sites.

As depicted in Fig. 4J, ES428’s efficacy targeting DHODH relies on the native mitochondrial context, yet *in situ* membrane-bound protein structures remains elusive. Molecular dynamics (MD) simulations offer a powerful alternative. To elucidate ES428–DHODH interactions in physiologically relevant mitochondrial lipid environment, we conducted all-atom MD simulations of full-length DHODH (DHODH-FL) with a phosphatidylethanolamine (PE) bilayer model, the most abundant mitochondrial membrane lipids. For comparison, we also performed MD simulations of the truncated DHODH-ΔN30 variant lacking the N-terminal mitochondrial targeting and transmembrane segments essential for DHODH’s proper localization^37^. S-ES428 served as the tool compound and BRQ as the reference. The root mean square deviation (RMSD) of backbone atoms was monitored, with stable regions identified for further cluster analysis (Fig. 5A). The interaction energy (Δ*G*_Bind_) calculations over the course of simulation trajectories revealed that S-ES428–DHODH interaction was significantly stabilized in the presence of lipid bilayer (Fig. 5B and C). Conversely, BRQ’s interaction energy showed no significant variation irrespective of lipid presence. This underscored the important role of mitochondrial lipids presence in stabilizing S-ES428–DHODH interaction. Furthermore, structural clustering of stable MD trajectory segments was conducted to provide detailed interaction insights. A unique binding mode of S-ES428 to lipid-bound DHODH was shown (Fig. 5D and E). The 9′-cycloheptyl group formed hydrophobic contacts with Y355, F97, V133, and P51; the *N*6′-methyl nitrogen and benzene ring engaged in cation‒*π* and *π‒π* stacking with F61; the lactone moiety at position 1 formed a hydrogen bond with Y37. Notably, S-ES428 spontaneously inserted into the lipid bilayer, establishing hydrophobic interactions *via* 6- and 7-methoxy groups with lipid tails. For the DHODH-NΔ30 variant, clustered interactions were primarily hydrophobic contacts *via* the 9′-cycloheptyl group, while the 6- and 7-methoxy groups tended to be solvent-exposed in the absence of lipids (Fig. 5F). In contrast, BRQ’s binding to lipid-bound DHODH resembled that of DHODH-ΔN30 without lipid bilayer, involving hydrophobic contacts with L57, A58, L66, L67, F61, F97, M110, and hydrogen bonds with R135 and Q46 (Supporting Information Fig. S12). Together, MD simulations revealed ES428’s unique dual-engagement mechanism *via* simultaneous interactions with DHODH’s CoQ binding pocket and mitochondrial membrane lipids.

**Figure 6:**
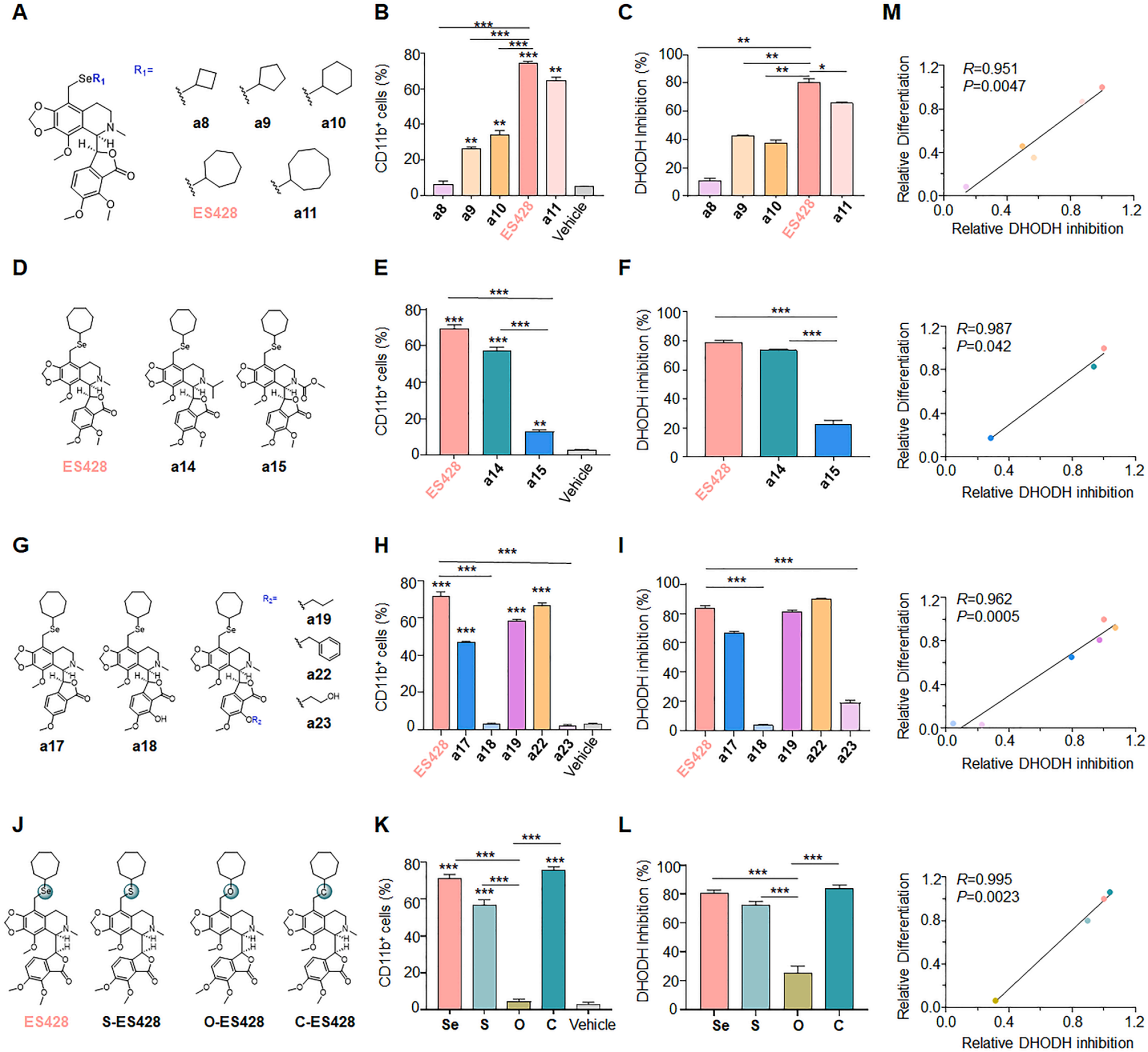
Comprehensive SAR studies substantiate the ES428-DHODH binding mode. Chemical structure of modified analogs at the 9′ position (A), 6′ position (D), 7 position (G), and Se-/S-/O-/C-substituted analogs (J) are shown. The differentiation-inducing activities of different analogs were shown in (B), (E), (H) and (K), following incubation at equivalent dose of 2 μmol/L for 3 days in U937 cells. The DHODH enzymatic inhibitory activities of different analogs as shown in (C), (F), (I) and (L), utilizing the mitochondrial isolation-based kinetic enzymatic assay at equivalent dose of 10 μmol/L. (M) Correlation between cellular potency in differentiation induction and the DHODH enzymatic inhibitory potency of different analogs as indicated. *R*, the Pearson correlation coefficient. Results are representative of at least three independent experiments. Error bars represent the mean ± SD of triplicate samples in an independent experiment. ∗*P* < 0.05, ∗∗*P* < 0.01 and ∗∗∗*P* < 0.001 against the vehicle-treated or between the line-pointed group.

We further performed comprehensive structure‒activity relationship (SAR) studies to substantiate ES428’s unique binding mechanism. Derivatives with bulky cycloalkyl groups at the 9′ position exhibited differentiation-inducing potential (Supporting Information Fig. S13A and B). The lipophilicity of 9′-substituents positively correlated with both cellular differentiation-inducing potency and DHODH enzymatic inhibition utilizing enzymatic assay based on isolated mitochondrial fraction (**a8‒a11**, Fig. 6A‒C), emphasizing the role of hydrophobic interactions *via* 9′ position. Modifications at the 6′ position revealed that an *N*-isopropyl substitution (**a14**) retained both DHODH enzymatic inhibition and differentiation-inducing activity, whereas urea analog (**a15**) with an electron-deficient nitrogen exhibited reduced potency (Fig. 6D‒F), supporting the role of cation‒*π* interaction. Conversion of the lactone moiety at position 1 to a cyclic ether (**a16**), which was anticipated to disrupt hydrogen bonding, markedly diminished differentiation induction (Supporting Information Fig. S13C and D). Substitutions at the 7-position ascertained the involvement of lipid engagement (Supporting Information Fig. S14A and B). Removal of the methoxy group (**a17**) partially impaired differentiation induction and DHODH inhibition, while hydrophobic but not hydrophilic substitutions were well tolerated at 7-position, further reinforcing the importance of direct hydrophobic interaction with lipids (Fig. 6G‒I). This further corroborated the suitability of hydrophobic linker attachment at 7-position to develop ES428’s active probes. Additionally, membrane permeability predictions indicated minimal impact from polar group introduction compared to ES428 (Supporting Information Fig. S15). Next, we aim to further substantiate the functional importance of ES428’s lipid-engaging mechanism for its efficacy. The DHODH-ΔN30 mutant lacks the N-terminal mitochondrial targeting and membrane-anchoring segment, resulting in defective mitochondrial localization. Consequently, DHODH-ΔN30 cannot sustain the mitochondrial DHODH activity required for *de novo* pyrimidine biosynthesis in cells, challenging the direct assessment of ES428-induced cellular differentiation *via* expressing this membrane-unbound mutant in AML cells. As an alternative, we performed the kinetic enzymatic assays utilizing whole-cell lysate as the enzyme source derived from cells transiently expressing either wild-type DHODH or the DHODH-ΔN30 mutant, the latter retaining *in vitro* catalytic activity despite defective mitochondrial localization. ES428 exhibited attenuated inhibitory potency against DHODH-ΔN30 compared to wild-type DHODH, whereas BRQ showed comparable inhibition of both variants (Fig. S14C and D). Additionally, SPR assays were conducted to assess ES428’s direct interaction with lipids by measuring its binding to POPC liposomes immobilized on an L1 sensor chip, mimicking membrane phospholipids bilayer. POPC was selected as it is a standard lipid commonly used in liposome research. SPR analysis demonstrated that ES428 bound POPC liposomes with high affinity (*K*_D_ ∼ 4.57 μmol/L) (Fig. S14E). Notably, MD simulations indicated that the 9′-sulfur atom did not directly contributed to binding (Fig. 5D). Further SAR (Fig. 6J‒L) demonstrated that S-ES428 and the carbon-substituted analog (C-ES428) maintained substantial DHODH inhibition and differentiation-inducing activity, with C-ES428 exhibiting efficacy comparable to ES428. In contrast, O-ES428 displayed marked reduction, consistent with prior results (Fig. S7B). We proposed that the differential activities may arise from variations in bond angles among these substituents. Moreover, strong correlations between DHODH inhibition and differentiation potency further validated DHODH inhibition as the primary mechanism underlying ES428-induced differentiation (Fig. 6M). Collectively, comprehensive SAR studies corroborated the unique binding mode defined by MD simulations. ES428’s direct interaction with lipids and the functional importance of lipid engagement for its mechanism of action was further underscored.

ES428’s unique DHODH-lipid dual engagement mechanism establishes a unique DHODH-targeting paradigm that enhances target engagement, differentiation-inducing potency, and selectivity in physiological settings. We proposed this superior pharmacological performance under physiological condition arising from stabilized ES428–DHODH interactions *in situ* indicated by MD simulations (Fig. 5B and C), and ES428’s selective mitochondrial localization evidenced by live-cell imaging (Fig. 4B). Notably, the hydrophobic interaction between ES428 and membrane lipid tails is unlikely to be specific to particular phospholipid species. We propose that this DHODH–lipid dual-engagement mechanism confers ES428’s mitochondrial targeting capability, facilitating its favorable positioning and preferential interactions with mitochondrial membrane lipids adjacent to the DHODH binding pocket.

### 3.6 EP300/CREBBP catalytic inhibition-mediated transcriptional regulation is essential for ES428-induced AML differentiation

**Figure 7:**
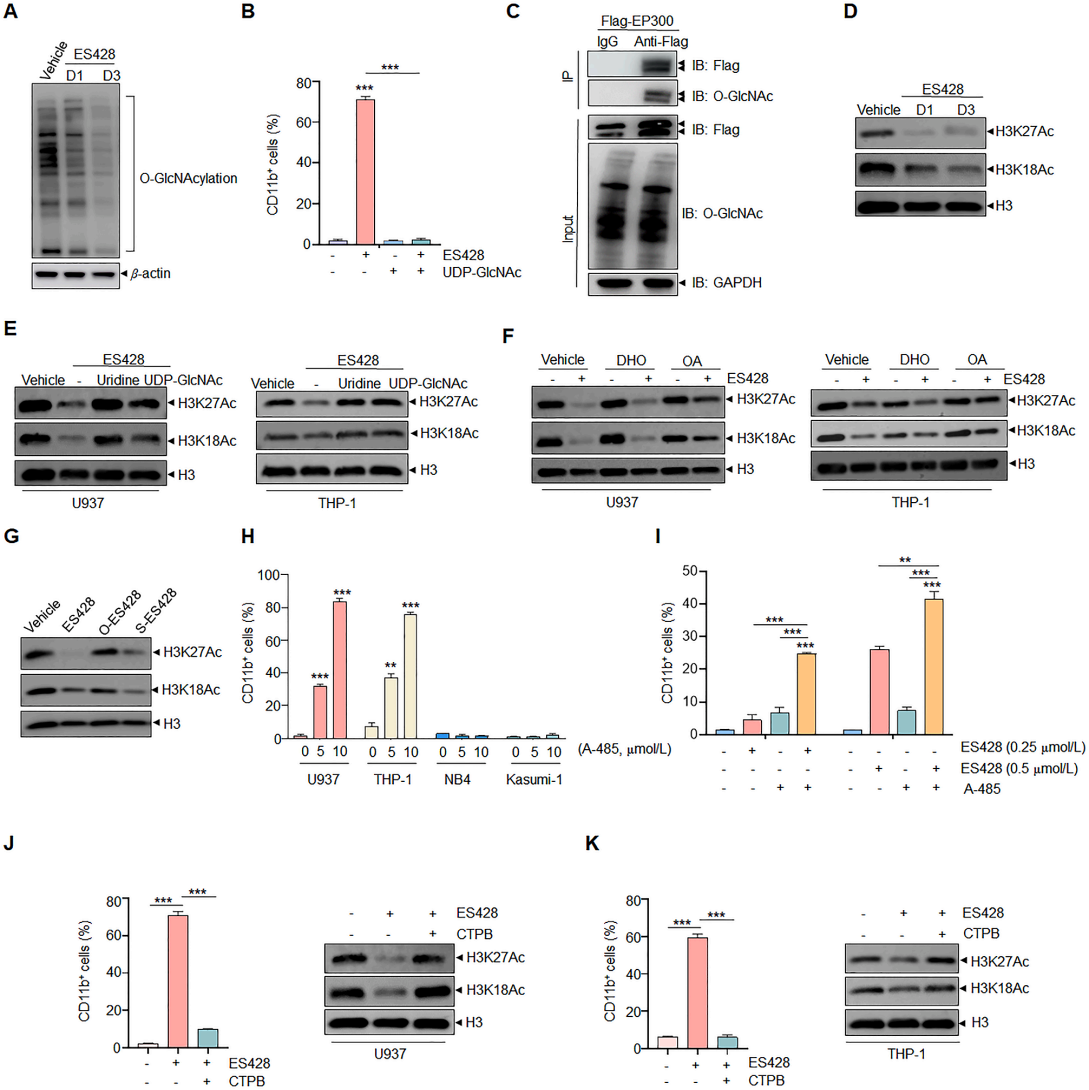
EP300/CREBBP catalytic inhibition is critical for ES428-induced AML differentiation. (A) U937 cells were incubated with ES428 at 2 μmol/L for the indicated days. The global protein O-GlcNAcylation level was detected by Western blotting. (B) U937 cells were incubated with ES428 at 2 μmol/L with or without the addition of excess UDP-GlcNAc for 3 days. The differentiation-inducing activity was evaluated by anti-human CD11b staining and flow cytometry analysis. (C) HEK293T cells that transiently overexpressed Flag-EP300 were harvested and the immunoprecipitation assay using anti-FLAG agarose beads was performed. The immunoprecipitated protein complex (top panel) and the corresponding lysates (input, bottom panel) were detected with antibodies against the FLAG-tag and *O*-GlcNAc antibody, and GAPDH was used as a loading control. (D) U937 cells were incubated with or without ES428 at 2 μmol/L for different time points as indicated. The expression of proteins as indicated was detected by Western blotting. (E, F) U937 cells (left) and THP-1 cells (right) were incubated with ES428 at 2 μmol/L with or without the addition of excess metabolites as indicated for 3 days. (E) Uridine, 100 μmol/L; UDP-GlcNAc, 100 μmol/L; (F) DHO, 500 μmol/L; Orotate, 500 μmol/L. The expression of proteins as indicated was detected by Western blotting. (G) U937 cells were incubated with ES428 or different analogs as indicated at equivalent dose of 2 μmol/L for 3 days. The expression of proteins as indicated was detected at Day 1 by Western blotting. (H) Multiple AML cell lines were incubated with A-485 at the indicated doses for 3 days. The differentiation-inducing activity was evaluated by anti-human CD11b staining and FACS analysis. (I) U937 cells were incubated with ES428 at concentrations as indicated in the presence or absence of A-485 of 1 μmol/L for 3 days. The differentiation-inducing activity was evaluated by anti-human CD11b staining and flow cytometry analysis. (J, K) U937 cells (J) and THP-1 cells (K) were incubated with ES428 at 2 μmol/L in the presence or absence of CTPB at 200 μmol/L for 3 days. The differentiation-inducing activity was evaluated by anti-human CD11b staining and FACS analysis (left). The expression of proteins as indicated was detected by Western blotting (right). Results in (B), (H), (I), (J) and (K) are representative of at least three independent experiments. Error bars represent the mean ± SD of triplicate samples in an independent experiment. ∗*P* < 0.05, ∗∗*P* < 0.01 and ∗∗∗*P* < 0.001 against the vehicle group and between the line-pointed group.

The mechanism underlying DHODH inhibition-induced AML differentiation remains incompletely understood. Utilizing ES428 as a probe, we further investigated the downstream molecular basis. Consistent with previous studies^14^, ES428 treatment resulted in a global reduction of O-GlcNAcylation modification of proteins (Fig. 7A). Supplementation with UDP-GlcNAc, the substrate for O-GlcNAcylation, significantly abrogated ES428-induced differentiation (Fig. 7B). We further explored potential O-GlcNAcylated protein(s) underlying ES428-induced AML differentiation. O-GlcNAc modification regulates a variety of proteins involved in multiple biological processes, including chromatin modifying enzymes such as histone acetyltransferase and methyltransferase^38^^,^^39^. The preliminary profiling of several histone marks at both histone H3 and H4 following ES428 treatment revealed a pronounced decrease in acetylation levels at histone H3 lysine residues K18 and K27(H3K18Ac and H3K27Ac), which are well-characterized hot spots catalyzed by the acetyltransferases EP300 and CREBBP (referred to as EP300/CREBBP, Supporting Information Fig. S16A). This observation suggests a potential functional connection between EP300/CREBBP and ES428-induced differentiation. EP300, as a key lysine acetyltransferase and transcriptional coactivator, was identified as an O-GlcNAcylated candidate^40^. Immunoprecipitation verified robust O-GlcNAcylation of EP300 (Fig. 7C), as well as its homologous counterpart CREBBP (Fig. S16B). EP300/CREBBP plays important roles in hematopoiesis and leukemogenesis^41^, although DepMap analysis revealed selective dependency of AML cells on EP300 over its homologous counterpart CREBBP (Fig. S16C). ES428 significantly inhibited the acetyltransferase activity of EP300/CREBBP, as evidenced by time-dependent reduction in H3K18Ac and H3K27Ac levels (Fig. 7D). This inhibition was significantly reversed by uridine or UDP-GlcNAc supplementation in U937 and THP-1 cells (Fig. 7E), accompanied by abrogation of AML differentiation. Importantly, supplementation with orotate, but not DHO, significantly restored EP300/CREBBP catalytic activity in ES428-treated U937 and THP-1 cells (Fig. 7F), linking EP300/CREBBP catalytic inactivation to ES428-mediated DHODH inhibition. Furthermore, ES428 and its active analog S-ES428, but not inactive O-ES428, significantly decreased H3K18Ac and H3K27Ac levels, correlating with their differentiation potency (Fig. 7G). Likewise, BRQ mirrored this effect (Fig. S16D), suggesting EP300/CREBBP inhibition as a common downstream event following DHODH inhibition. Moreover, pharmacological inhibition of EP300/CREBBP with selective catalytic inhibitor A-485 dose-dependently induced differentiation in U937 and THP-1 cells (Fig. 7H), accompanied by significantly reduced H3K18Ac and H3K27Ac levels (Fig. S16E). In contrast, inhibitors targeting other HAT family members, including PCAF (NSC694621, PCAF-IN-2), MOF (MG149), and Tip60 (NU9056), did not induce comparable differentiation (Fig. S16F), supporting the specific involvement of EP300/CREBBP. Additionally, A-485 mimicked ES428’s differentiation response, showing insensitivity in NB4 and Kasumi-1 cells. Furthermore, A-485 potentiated ES428-induced differentiation at low doses in U937 cells (Fig. 7I). Conversely, co-treatment with the EP300/CREBBP activator CTPB^42^ significantly abolished ES428-induced differentiation and restored EP300/CREBBP catalytic activity in U937 and THP-1 cells (Fig. 7J and K). Together, these results demonstrated that EP300/CREBBP catalytic inhibition *via* decreased O-GlcNAcylation is critical for ES428-induced AML differentiation.

**Figure 8:**
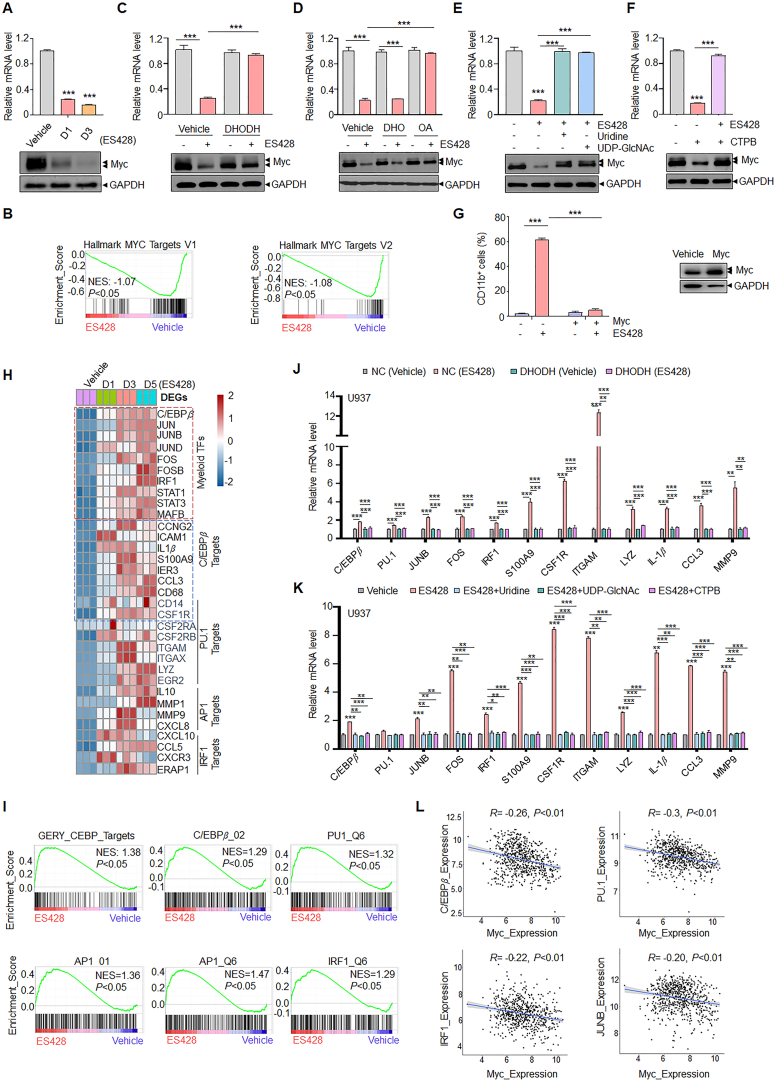
EP300/CREBBP inhibition-mediated transcriptional reprogramming is invovled in ES428-induced myeloid differentiation. (A) U937 cells were incubated with ES428 at 2 μmol/L for different time points as indicated, the mRNA (top) and protein (bottom) levels of Myc were detected by quantitative real-time PCR and western blotting, respectively. GAPDH was detected as the loading control. (B) Enrichment plots of gene sets through GSEA analysis of transcriptional profiling following ES428 treatment at Day 1 are shown. NES, normalized enrichment score. (C) U937 cells stably overexpressing DHODH were treated with ES428 for 3 days. The mRNA (top) and protein (bottom) level of Myc were detected at Day 1 by quantitative real-time PCR and western blotting, respectively. (D‒F) U937 cells were incubated with ES428 at 2 μmol/L with or without the addition of excess metabolites as indicated. DHO and Orotate (D); Uridine or UDP-GlcNAc (E) as well as CTPB (F). The mRNA (top) and protein (bottom) level of Myc were detected at Day 1 by quantitative real-time PCR and Western blotting, respectively. (G) U937 cells stably overexpressing Myc were treated with or without ES428 and the differentiation-inducing activity was evaluated by anti-human CD11b staining and FACS analysis. The protein level of Myc overexpression was detected through Western blotting. (H) Heatmap of expression profile of DEGs following ES428 treatment at different time points. These genes are related to several important myeloid TFs involved in myeloid differentiation, and their representative target genes. The myeloid TFs are shown as red dashed box, and C/EBP*β* target genes are shown as blue dashed box. (I) Enrichment plots of gene sets through GSEA analysis of transcriptional profiling following ES428 treatment at Day 1 are shown. NES, normalized enrichment score. (J, K) U937 cells stably overexpressing DHODH were treated with ES428 (J); and U937 cells were incubated with ES428 at 2 μmol/L with or without the addition of excess uridine, UDP-GlcNAc or CTPB. The mRNA levels of myeloid TFs and their representative target genes as indicated were detected at Day 1 by quantitative real-time PCR. (L) The correlation analysis of gene expression of Myc (*x* axis) and C/EBP*β*, PU1, IRF1, JUNB (*y* axis) at mRNA levels based on BeatAML2.0 database. Results in (A), (C‒G), (J) and (K) are representative of at least three independent experiments. Error bars represent the mean ± SD of triplicate samples in an independent experiment. ∗*P* < 0.05, ∗∗*P* < 0.01 and ∗∗∗*P* < 0.001 against the vehicle group and between the line-pointed group.

EP300/CREBBP imparts acetylation marks through its catalytic activity and is typically involved in transcriptional regulation of cell-type-specific genes to maintain cell identity^43^. Our transcriptome profiling upon ES428 treatment revealed significant transcriptional alterations (Fig. S5A), suggesting the potential involvement of EP300/CREBBP inhibition in transcriptional regulation. EP300/CREBBP has been implicated as a master regulator of enhancer-driven oncogenes such as Myc, a critical genetic dependency in AML pathogenesis^43^^,^^44^. Accordingly, ES428 time-dependently reduced Myc expression at both mRNA and protein levels in U937 and THP-1 cells (Fig. 8A and Supporting Information Fig. S17A), and GSEA verified the inhibition of Myc-driven transcription program (Fig. 8B and Fig. S17B). Importantly, both DHODH overexpression and supplementation with orotate, but not DHO, significantly restored Myc level in ES428-treated U937 and THP-1 cells (Fig. 8C, D and Fig. S17C), thereby linking Myc silencing to ES428-mediated DHODH inhibition. Furthermore, EP300/CREBBP inhibitor A-485 mimicked the rapid downregulation of Myc (Fig. S17D), while EP300/CREBBP activator CTPB co-treatment and supplementation with uridine or UDP-GlcNAc significantly restored Myc expression (Fig. 8E, F and Fig. S17E and S17F). Moreover, Myc overexpression significantly attenuated ES428-induced differentiation (Fig. 8G). These findings demonstrated Myc repression *via* EP300/CREBBP catalytic inhibition as an important downstream event underlying ES428-induced differentiation.

Beyond Myc downregulation, ES428 upregulated multiple myeloid differentiation-associated TFs^45^ and their targets involved in myeloid maturation and inflammatory response (Fig. 8H), consistent with ES428’s phenotype. GSEA substantiated enrichment of these TF target sets post-ES428 treatment (Fig. 8I and Fig. S17G). Importantly, ES428-induced upregulation of these TFs and their representative targets was significantly reversed by DHODH overexpression (Fig. 8J and Fig. S17H), indicating a mechanistic link to DHODH inactivation. Similarly, this reversal was also observed through CTPB co-treatment and supplementation with uridine or UDP-GlcNAc in U937 and THP-1 cells (Fig. 8K and Fig. S17I), underscoring the reliance on EP300/CREBBP catalytic inhibition. Additionally, Myc knockdown partially recapitulated ES428-induced TFs activation pattern based on previously published RNA-seq data (Fig. S17J). In silico analysis of primary AML blasts showed negative correlations between Myc expression and level of key TFs including C/EBP*β*, PU.1, JUNB, and IRF1 (Fig. 8L). Together, these results indicated that EP300/CREBBP catalytic inhibition orchestrated transcriptional reprogramming by suppressing Myc-driven leukemogenic program and activating myeloid differentiation-associated TF networks. Consistent with resistance to ES428-induced differentiation, Kasumi-1 cells showed no significant changes in H3 acetylation marks, Myc and differentiation-related key TFs, indicating minimal EP300/CREBBP inhibition. Despite reduced H3K18/27Ac and Myc levels in NB4 cells, upregulation of differentiation-promoting TFs remained limited, indicating incomplete transcriptional switching (Supporting Information Fig. S18A‒D). Moreover, overexpression of AML1-ETO or PML-RAR*α* in U937 cells significantly diminished ES428-induced differentiation, concomitant with impaired induction of key myeloid differentiation-related TFs, directly implicating these specific oncoproteins in resistance to differentiation upon DHODH inhibition (Fig. S18E‒J). Further, combining ES428 with low-dose ATRA significantly enhanced differentiation in NB4 cells (Fig. S18K), likely by relieving PML-RAR*α*-mediated transcriptional repression.

FAB M4 and M5 subtypes exhibited susceptibility to ES428-induced differentiation, while no significant variation in DHODH, EP300 or CREBBP expression across AML subtypes or recurrent genetic abnormalities was shown through BeatAML dataset (Supporting Information Fig. S19). Instead, these subtypes exhibited lower O-GlcNAc transferase (OGT) and Myc levels, alongside higher expression of differentiation-promoting TFs such as PU.1 and C/EBP*β* (Supporting Information Fig. S20). We proposed that low OGT might facilitate EP300/CREBBP catalytic inhibition *via* decreasing O-GlcNAcylation, and the intrinsically low Myc and high differentiation-associated TFs signature establishes a “primed” state favoring differentiation upon DHODH inhibition.

## 4 Discussion

Differentiation arrest is a hallmark of myeloid malignancies, positioning differentiation therapy as a promising therapeutic strategy. Although differentiation therapy has revolutionized treatment for APL and IDH-mutant AML, effective options for most AML subtypes remain an unmet need. To broaden its applicability, our study leverages drug repositioning of natural products derived from traditional medicinal plants and couples with phenotypic screening to explore therapeutic opportunities for AML differentiation.

The discovery of ES428 exemplifies the potential of natural scaffold-based drug repositioning, transforming noscapine’s antitussive and mitotic-disrupting activities into ES428’s AML differentiation-inducing capacity. It also underscores the advantage of phenotypic screening in identifying novel therapies and mechanisms, particularly for undefined or intricate targets^46^. However, target identification of natural products is quite challenging. Here, we employed combinatorial target deconvolution strategies integrating multi-omics, computational target fishing, label-free biophysical assays, photoaffinity labeling, enzymatic assays, and cellular functional studies, to rigorously validate DHODH as ES428’s direct functional target underling AML differentiation.

DHODH represents a promising therapeutic target for diverse diseases, including viral infections, autoimmune disorders, and cancer. Nevertheless, anti-cancer application of clinical available DHODH inhibitors remains limited, due in part to suboptimal efficacy, low target selectivity, and undesirable off-target effects such as kinase inhibition. Currently, very few DHODH inhibitors have progressed to clinical use, underscoring the urgent need for new strategies with improved therapeutic efficacy and selectivity. DHODH’s localization within the inner mitochondrial membrane and its critical reliance on lipid‒protein interaction are underexplored for drug discovery^22^, ^23^, ^24^, as conventional *in vitro* enzymatic assays or structure-based virtual screening typically neglect DHODH’s native membrane-associated features^20^^,^^47^^,^^48^. In this context, ES428’s DHODH–lipid dual engagement mechanism stabilizes the enzyme–inhibitor complex *in situ* and confers ES428 intrinsic mitochondria targeting, establishing a unique DHODH-targeting paradigm that leverages DHODH’s native membrane association to enhance therapeutic efficacy, selectivity, and safety in physiological settings. This dual-engagement mode may serve as an “anchor”, stabilizing target binding and interaction *in situ*; and a “filter,” likely concentrating drug locally in mitochondria and restricting drug diffusion into the cytosol and nucleus. These effects combine to enhance on-target potency under physiological conditions while minimizing off-target interactions. Additionally, dual-engagement might impose spatial constraints to enhance targeting selectivity for DHODH over other structurally related CoQ-binding proteins (*e.g.*, respiratory complex)^22^. This strategy holds promise to advance DHODH-directed therapies for AML and implicates drug development targeting other membrane-associated targets.

Using ES428 as a probe, we delineated a mechanistic cascade linking pyrimidine synthesis, post-translation O-GlcNAcylation, EP300/CREBBP acetyltransferase activity, and transcriptional regulation underlying DHODH inhibition-induced AML differentiation. EP300 and its homologue CREBBP are key acetyltransferases and transcriptional coactivators with context-dependent tumor-suppressive and oncogenic roles in hematopoiesis and AML pathogenesis^49^, ^50^, ^51^. Pharmacologic inhibition of EP300/CREBBP has shown therapeutic potential in hematologic malignancies, including AML^52^. Here, we emphasized the critical role of EP300/CREBBP catalytic inactivation *via* decreased O-GlcNAcylation in DHODH inhibition-triggered AML differentiation, which subsequently orchestrated transcriptional reprogramming by suppressing Myc-driven leukemogenic program while activating differentiation-promoting TFs networks. A limitation of our study is that it does not resolve the individual contributions of EP300 and CREBBP to AML differentiation in response to DHODH inhibition. Although EP300 and CREBBP share high sequence and structural similarity, previous studies have indicated both overlapping and non-redundant functions^53^^,^^54^. Therefore, future work employing paralog-specific genetic perturbations and paralog-selective chemical tools (*e.g.*, selective degraders) will be necessary to determine whether the differentiation response depends preferentially on one paralog or requires the cooperative activity of both. Moreover, the detailed mechanism by which DHODH inhibition modulates EP300/CREBBP activity through O-GlcNAcylation requires further clarification. This motivates systematic mapping of functionally relevant O-GlcNAcylation sites, for example *via* immunoprecipitation-coupled quantitative LC–MS/MS followed by validation through site-directed mutagenesis. Notably, EP300/CREBBP function is also regulated by diverse post-translational modifications (PTMs), particularly phosphorylation^55^. Given that both O-GlcNAcylation and phosphorylation commonly target serine/threonine residues and may act competitively^56^, potential crosstalk between these modifications warrants further investigation. Determining whether these PTMs are shared or distinct between EP300 and CREBBP will further refine mechanistic understanding of DHODH inhibition-driven AML differentiation.

As a critical driver of leukemogenicity and AML maintenance, targeting long-range enhancer-driven Myc hyperactivation presents a potential therapeutic strategy for Myc-driven AML^57^^,^^58^. Additionally, previous studies also revealed that O-GlcNAc modification affected Myc protein stability^59^, indicating multi-layered regulation of Myc under DHODH inhibition. Myc suppression was at least partially essential for inducing myeloid differentiation-associated TFs, likely through Myc-driven chromatin remodeling^60^ and/or relief of Myc-mediated repression of lineage-specifying TFs such as C/EBP family members^61^.

The sensitivity of ES428 in FAB M4 and M5 subtypes likely results from lower OGT expression and a “differentiation-primed” state. These subtypes that are clinically associated with chemoresistance and unfavorable prognosis^62^^,^^63^, therefore represent prime candidates for DHODH-targeted differentiation therapy. Conversely, M2b (AML1-ETO^+^) and M3 (PML-RAR*α*^+^) subtypes appear to exhibit resistance. Their respective chimeric oncoproteins, AML1-ETO and PML-RAR*α*, are known to drive leukemogenesis through dual functions^64^^,^^65^: recruiting EP300/CREBBP to preferentially co-occupy enhancer regions to transactivate genes critical for leukemogenesis^66^, and assembling co-repressors (*e.g.*, NCoR/SMRT) and other chromatin-modifying enzymes (*e.g.*, HDACs) to repress differentiation-promoting genes such as C/EBP and PU.1^67^^,^^68^. This suggests a shared resistance mechanism likely stemming from these chimeric oncoprotein-mediated recruitments of repressive complexes, suppressing key differentiation regulators. Notably, overexpression of PML-RAR*α* or AML1-ETO conferred substantial, but not complete resistance, implicating additional cooperating factors. Besides, the differing resistance profiles impacting EP300 activity and the resultant transcriptional regulation, observed between intrinsic Kasumi-1 and NB4 cells as well as engineered cell models, further suggest a complex, and context-dependent mechanism. This complexity may involve alteration in chromatin conformation, histone modification pattern, and co-factor recruitment. Future multi-omic epigenetic profilings (*e.g.*, ChIP-seq, ATAC-seq) are warranted to define locus-specific chromatin changes and to identify therapeutic strategies to overcome resistance in M2/M3 AML. We propose that FAB-defined M4/M5 subtypes or their associated gene signatures may serve as potential biomarkers to identify AML patients most likely to benefit from DHODH-targeted differentiation therapy. Additionally, our findings also offer a mechanistic rationale for combination strategies, such as combining DHODH inhibitors with EP300/CREBBP catalytic inhibitors or other differentiation-promoting agents (*e.g.*, ATRA) to optimize therapeutic efficacy. This warrants future *in vivo* evaluation, particularly the latter, given its potential in M2/M3 AML models exhibiting intrinsic resistance to DHODH-targeted differentiation therapies.

## 5 Conclusions

ES428 has been identified as a potent AML differentiation inducer verified *in vitro* and *in vivo*, particularly in the FAB M4 and M5 subtypes. Its unique mechanism involved dual engagement with DHODH and mitochondrial lipids. This dual-engagement stabilized ES428–DHODH interaction *in situ* and promotes the selective mitochondrial targeting of ES428, thereby enhancing therapeutic efficacy and selectivity in physiological settings. ES428 orchestrates a downstream metabolic‒epigenetic‒transcriptional axis, linking pyrimidine depletion, catalytic inhibition of EP300/CREBBP, and transcriptional reprogramming to drive differentiation. Our study presents promising lead candidates, and offers novel mechanistic and translational insights to expand differentiation therapies for myeloid malignancies.

## Author contributions

Shuting Shen: Methodology, Investigation, Visualization, Writing-original draft, Writing-review & editing. Defeng Li: Methodology, Investigation, Visualization, Writing-original draft. Youping Zhang: Methodology, Investigation, Visualization. Shiwei Li: Methodology, Investigation, Visualization, Writing-original draft. Lei Li: Methodology, Visualization. Wenhao Shen: Methodology, Investigation. Yujie Ma: Resources. Wenhao Zhang: Resources. Rong Tao: Methodology, Resources. Wei Wang: Methodology, Resources. Biao Jiang: Funding acquisition, Resources, Project administration, Writing-review & editing. Fang Bai: Conceptualization, Methodology, Writing-review & editing. Chuanxu Liu: Conceptualization, Investigation, Visualization, Resources, Funding acquisition, Writing-review & editing. Yongqiang Zhang: Conceptualization, Funding acquisition, Visualization, Supervision, Writing-review & editing. Qianqian Yin: Conceptualization, Investigation, Visualization, Funding acquisition, Supervision, Writing-original draft, Writing-review & editing.

## Conflicts of interest

The authors declare no conflicts of interest.

## Appendix A Supporting information

The following are the Supporting Information to this article:
