# Supplementary material for: Novel noscapine derivative induces AML differentiation by dual-engaging DHODH and mitochondrial lipids to trigger a metabolic–epigenetic–transcriptional cascade
Source: Acta Pharm Sin B. 2026 May 22;16(8):5363–82. doi: 10.1016/j.apsb.2026.05.019 (PMC13464086; doi:10.1016/j.apsb.2026.05.019)
Supplement: Supplementary file 1 [file mmc1.pdf]

Supporting Information for

Original article

Novel noscapine derivative induces AML differentiation by dual-engaging DHODH and mitochondrial lipids to trigger a metabolic–epigenetic–transcriptional cascade

Shuting Shen[a,b,†], Defeng Li[d,†], Youping Zhang[e,†], Shiwei Li[a,b,†], Lei Li[a,b], Wenhao Shen[a,b], Yujie Ma[e], Wenhao Zhang[c,e], Rong Tao[c,e], Wei Wang[f], Biao Jiang[a,g,*], Fang Bai[a,b,*], Chuanxu Liu[c,e,*], Yongqiang Zhang[d,*], Qianqian Yin[a,*]

[a]*Shanghai Institute for Advanced Immunochemical Studies, ShanghaiTech University, Shanghai 201210, China*

[b]*School of Life Science and Technology, ShanghaiTech University, Shanghai, 201210, China*

[c]*Department of Lymphoma, Fudan University Shanghai Cancer Center, Shanghai 200032, China*

[d]*Shanghai Frontiers Science Center of Optogenetic Techniques for Cell Metabolism, Shanghai Key Laboratory of New Drug Design, and School of Pharmacy, East China University of Science and Technology, Shanghai 200237, China*

[e]*Department of Hematology, Xinhua Hospital, Shanghai Jiao Tong University School of Medicine, Shanghai 200092, China*

[f]*Department of Pharmacology and Toxicology and BIO5 Institute, University of Arizona, Tucson, AZ 85721, USA*

[g]*CAS Key Laboratory of Synthetic Chemistry of Natural Substances, Shanghai Institute of Organic Chemistry, Chinese Academy of Sciences, Shanghai 200032, China*

Received 26 June 2025; received in revised form 8 February 2026; accepted 17 April 2026

[†]These authors made equal contributions to this work.

*Corresponding authors.

E-mail addresses: jiangbiao@shanghaitech.edu.cn (Biao Jiang), baifang@shanghaitech.edu.cn (Fang Bai), liuchuanxu@shca.org.cn (Chuanxu Liu), yongqiangzhang@ecust.edu.cn (Yongqiang Zhang), yinqq@shanghaitech.edu.cn (Qianqian Yin).

1. Materials and methods

1.1 Biologic methods

Cell proliferation assays

Cells were seeded at a density of 2×10^5/ml cells and treated with the indicated compounds. Cell densities and viability were monitored at different time points post-treatment using Vi-CELL XR (Beckman coulter). For dose-response assays, cells were plated at the same density in 96- or 384-well plates. Compounds were 2-fold serially diluted in the culture medium into each well. After incubation for 48 h, proliferation was quantified by CCK-8 assay. Absorbance values were normalized to DMSO controls, and IC_{50} values were determined by nonlinear regression in GraphPad Prism.

Cell differentiation assays

Cell morphological features were assessed after Wright-Giemsa staining (BASO Diagnostic, Zhuhai, China) according to the manufacturer's instructions. The α-NAE staining was performed using a commercial α-NAE staining kit (Solarbio life sciences, G2400). Stained preparations were imaged using a Nikon Eclipse 90i microscope. The cell-surface differentiation antigens CD11b and CD14 were measured using FITC or APC-labeled antibodies with isotype controls (Beckman–Coulter) via flow cytometry. Data were processed using FlowJo_v10.8.1 software.

Recombinant DHODH expression and purification

Human DHODH (Thr31-Arg396) was cloned into pET-28a vector (RRID: Addgene_141289) and expressed in BL21(DE3) E. coli strain. Cells were grown in a 37°C shaker in Luria-Bertani (LB) medium. After induced with 0.5 mmol/L IPTG at an OD_{600} of 0.7–0.8, the cells were harvested and lysed with sonication in buffer I (50 mmol/L HEPES, pH 7.7, 300 mmol/L NaCl, 10% glycerol, 1% Triton X-100). The lysate supernatant was obtained by centrifugation at 12,000 g for 45 min and incubated with Ni-Charged Resin FF (GenScript, L00683) pre-equilibrated with buffer I. After washing with buffer II (buffer I supplemented with 50 mmol/L imidazole), recombinant DHODH protein was eluted with buffer III (buffer I supplemented with 300 mmol/L imidazole). The purified recombinant DHODH was dialyzed to remove the imidazole and concentrated via 30 kDa filter (Millipore, UFC903024).

DHODH enzymatic assays

DHODH activity was measured by DCIP reduction spectrophotometric assay[1] in 384-well plates at room temperature. The reaction mixture (pH 8.0) was composed of 50 mmol/L Tris, 0.1% Triton X-100, 150 mmol/L KCl, 0.4 μg/ml DHODH, 1 mmol/L DHO, 0.1 mmol/L

decylubiquinone, 0.06 mmol/L DCIP. After adding test compounds, the reaction was initiated by substrate addition. DCIP absorbance decrease at 600 nm was monitored kinetically using a microplate reader. DHODH activity using the isolated mitochondrial fractions was assessed using a modified DCIP-linked assay as previously described[2]. In brief, U937 cells with DHODH overexpression were collected and re-suspended in potassium phosphate buffer (0.1 mol/L, pH 7.0). The mitochondrial fractions were isolated using the Mitochondria Isolation Kit. The mitochondrial fractions were incubated in the solution of PBS (400 µl PBS for 2×10^7 cells). The reaction was initiated with 1 mmol/L DHO in 60 µl of standard reaction buffer supplemented with 0.06 mmol/L DCIP, 5 µmol/L rotenone (a complex I inhibitor), 5 µmol/L antimycin A (a complex III inhibitor), 5 mmol/L NaN_3 (a complex IV inhibitor) and 20 µl mitochondrial fraction.

Synthesis of ES428-photoaffinity probe and *in situ* photoaffinity labeling-coupled pulldown assay

Detailed synthesis of ES428-PAP was described in Chemical Synthesis. U937 Cells were pretreated with excess competitor or DMSO for 1 h before adding ES428-PAP, followed by UV irradiation (10 min) to crosslink the probe to targets. Cells were harvested, washed with cold PBS, lysed by sonication, and centrifuged to obtain lysates. Protein concentrations were quantitated by Bradford assay. A freshly pre-mixed click chemistry reaction cocktail (100 µ mol/L tert-Butyl-2,2,2-trichloroacetimidate; 2 mmol/L CuSO4; 4 mmol/L L-ascorbic acid sodium salt; 100 µmol/L Biotin-PEG3-N3; and 1 mmol/L TCEP hydrochloride) was added and incubated for 2 h at room temperature with shaking. Proteins were precipitated with acetone overnight, collected by centrifugation, washed, and resuspended in PBS with 0.1% SDS. Streptavidin agarose beads were added and incubated at 4 °C overnight. Beads were washed sequentially with PBS containing 0.1% SDS for twice, and PBS for twice before proteins were eluted and analyzed by western blot.

TSA and CETSA assays

For the TSA assay, recombinant DHODH (1 µmol/L) was incubated with or without ES428 at 37°C for 30 minutes, aliquoted into PCR tubes, and subjected to a temperature gradient-heated for 3 minutes at designated temperatures followed by incubation for 3 minutes at 25°C. Samples were then centrifuged at high speed to collect supernatants. For the CETSA assay, DHODH-overexpressing U937 cells were treated with ES428 or DMSO for 12 h. Cells were harvested, and resuspended in PBS containing protease inhibitors (1:100). Then, cells were lysed via three freeze-thaw cycles (1 minute at 37°C water bath followed by 1 minute in liquid nitrogen).

Lysates were centrifuged at 15,000 rpm, 4°C for 15 minutes, and supernatants collected, aliquoted into PCR tubes, and analyzed by western blotting.

Surface plasmon resonance assay

Recombinant DHODH (20 μg/ml) was immobilized on a CM7 sensor chip (Cytiva) to a level of around 10000 response units using a Biacore 8k (Cytiva) via amine coupling and a running buffer composed of 1×PBS-P^{+} buffer (cytiva, 280304) and 5% DMSO. Subsequently, a series of concentration gradients of ES428 were injected into the system to interact with the immobilized DHODH protein on the chip. Lipids (phosphatidylcholine) were purchased from Avanti Polar Lipids (Alabaster, AL) as lyophilized powders. Powders were dissolved in PBS to a nominal concentration of 10 mmol/L phospholipid and shaken for at least 1 h at room temperature. When fully dissolved, lipid suspensions were extruded through a 100-nm polycarbonate filter using an Avanti Mini-Extruder kit. The lipid suspension was passed though the membrane 19 times to yield a clear solution of uniformly sized liposomes. Liposomes were prepared for capture by dilution in running buffer to a concentration of 2 mmol/L phospholipid. Liposomes were captured to saturation at a flow rate of 2 μl/min with an injection time of 180 s. One flow cell was left blank to act as a reference surface. The capture of the liposomes on the L1 chip is due to reversible, hydrophobic binding. Interaction analysis was performed using a Biacore 8K instrument. All analyses were run at 25°C using an L1 sensor chip. Running buffer was 5% DMSO in phosphate-buffered saline (PBS) (0.01 mol/L phosphate, 2.7 mmol/L KCl, and 0.137 mol/L NaCl) with final pH 7.4. Before the start of each run, two 30-s consecutive pulses with 50 mmol/L NaOH: isopropanol 6:4 (*v*/*v*) (regeneration solution) were made at a flow rate of 100 μl/min to regenerate the chip surface. The resulting data required from instrument were fitted to a 1:1 binding model using Biacore Evaluation Software (Cytiva).

Protein structure preparation and molecular docking

To examine the interactions of our compounds with DHODH and assess potential differences in binding between its truncated and full-length forms, we first acquired the structures of both variants. The truncated DHODH (aa 32–395) was sourced from the RCSB Protein Data Bank (PDB Code: 4IGH). The full-length DHODH structure was computationally predicted by integrating deep learning-based AlphaFold2[3] predictions with the experimentally determined truncated DHODH structure. During the construction of the full-length structure, alignment of the truncated and predicted full-length structures was carried out using the Schrödinger Suite 2022-1[4], and the segment encompassing amino acids 32-395 was modeled based on the resolved structure, while the N-terminal region (1–31) was derived from the predicted full-length DHODH structure. Both truncated and homology-modeled full-length DHODH structures

underwent preparation with PrepWizard[5] to ensure the correct ionization state at pH 7.25 and to add any missing atoms. To pinpoint potential binding sites for docking simulations, we employed SiteMap[6] to analyze the entire protein surface of the truncated DHODH structure in the absence of any ligand molecules. A minimum of 15 points was used to identify binding sites. This analysis revealed two potential binding sites at the CoQ and FMN sites. Subsequently, the ligands ES428 and BRQ were prepared using LigPrep[7] from the Schrödinger Suite 2022-1 [4], which included processes for assigning ionization states, charges, and hydrogen atoms. Molecular docking was conducted with Glide[8], utilizing the generated grid file over the predicted binding sites. The grid box was configured with an inner size of 14×14×14 $Å^3$ for positioning the ligand's center of mass and an outer box size of 20×20×20 $Å^3$ to encompass all potential non-bonded interactions. Docking simulations were performed using Glide's SP precision, with the OPLS4 force field[9], and enhanced sampling carried out twice to increase accuracy. The complex structures derived from molecular docking simulations were optimized by Molecular Mechanics with Generalized Born and Surface Area solvation (MM/GBSA) to consider the flexibility of protein and solvation effect during binding. The MM/GBSA was conducted by prime[10] from Schrödinger Suite 2022-1, including protein atoms within 5 Å range of ligands as flexible region, and sample method was set as "minimize".

Molecular dynamics simulations

The Desmond software was utilized to perform molecular dynamics simulations on four distinct systems: i) the full-length DHODH/S-ES428 complex with a PE lipid bilayer, ii) the truncated DHODH/S-ES428 complex, iii) the full-length DHODH/Brequinar complex with PE bilayer, and iv) the truncated DHODH/Brequinar complex. The positioning of the membrane bilayers for the two membrane systems was predicted using the Positioning of proteins in membranes (PPM) 2.0 webserver[11], and the TIP3P model was used as the solvent model, with the ion concentration set at 0.15 mmol/L potassium chloride. For systems containing membrane bilayers, the sizes of the cubic Periodic Boundary Conditions (PBC) box were set to 150 × 100 × 80 Å3, whereas for systems without membrane bilayers, the box sizes were adjusted to 85 × 85 × 85 Å3. The initial S-ES428 protein–ligand complex model used for MD simulation was obtained from optimized molecular docking simulation results. Brequinar was included as a reference ligand in two systems, with the initial structure derived from crystallographic data (truncated system) and docking results with the full-length DHODH (full-length system). Each system underwent three independent 400-nanosecond simulations. Subsequent analysis of the trajectories focused on the root mean square deviation (RMSD) of the protein backbone atoms. Trajectory clustering was conducted using Schrödinger Suite 2022-1, based on the RMSD of

protein heavy atoms, and the clustered complex structures were analyzed and visualized with PyMOL[12].

Quantitative real-time PCR

RNA extraction was performed using RNA extraction kit (SparkJade, AC0205). Reverse transcription was performed using reverse transcription kit (Yeasen, 11151ES60) according to the instruction manual. The cDNA obtained from reverse transcription and qPCR SYBR Master Mix (Yeasen, 11185ES60) were used for qRT-PCR experiment. Samples were analyzed using QuantStudio 7 (ThermoFisher Scientific). Relative expression levels were calculated by the ΔΔCT value method. Microsoft Excel and Graphpad Prism were used for quantitative analysis.

Western Blotting

Cell lysate was boiled for 5 min in 1×SDS sample buffer (50 mmol/L Tris-HCl, pH 6.8; 10% glycerol; 2% SDS; 10 mmol/L DTT). Protein concentration was determined by the Bradford Assay Kit (MeilunBio, MA0079). Equal amounts of lysate were separated by SDS-PAGE and transferred to nitrocellulose membranes (Epyzime, WJ004). After blocking in 5% non-fat milk for 1 hour at room temperature, antibodies specific for the indicated proteins were incubated overnight at 4°C. Then, the membranes were washed three times with 1 × TBST buffer and incubated with secondary antibody for 1 hour at room temperature. Blots were imaged in an Amersham Imager 680 (GE Healthcare).

Cell cycle analysis

Cells were seeded at 2×10^5/ml and treated with test compounds for 24 h. Subsequently, cells were harvested by centrifugation at 1500g and 4°C for 5 minutes and washed three times with cold PBS. Then, cells were fixed overnight in 75% ethanol at -20 °C, washed with cold PBS for three times, and resuspended in 100 µL staining solution containing 10 µl RNase A and 10 µl Propidium Iodide. Following incubation at 37 °C for 30 min, samples were acquired on a CytoFLEX flow cytometer (Beckman Coulter, USA) and analyzed using ModFit LT 5.0 (Verity Software House, USA).

Cell apoptosis assay

Cells were seeded at a density of 2×10^5/ml cells and treated with test compounds. Cells were stained with Annexin V-FITC and propidium iodide according to the Annexin V-FITC/PI Apoptosis Detection Kit protocol (MeilunBio #MA0220). Data were collected on the CytoFLEX cytometer.

Plasmid construction and cell transfection

Plasmids encoding DHODH, Myc, and EP300 were generated by PCR amplification of coding sequence from cDNA. PCR products were cloned into pMSCV-puro vector (RRID:

Addgene_119745), and verified by DNA sequencing in Tsingke Biotech (Beijing, China). For retroviral transduction, HEK-293T cells were seeded and transfected with pMSCV-DHODH or pMSCV-Myc expression plasmid and packaging plasmids gag-pol (RRID: Addgene_14887) and VSVG (RRID: Addgene_14887) using PEI40000 agent (Yeasen, 40816ES03) according to the manufacturer's instructions. At 48 h post-transfection, viral supernatants were collected to infect U937 and THP-1 cells. Protein expression was confirmed by western blotting.

Colocalization imaging

Following live cells pre-treatment with or without Bodipy-ES428 for 2h, Mito-Tracker Red (1 μmol/L) and Hoechst 33342 dye (10 μg/mL) were added to the chambers with incubation for 15 min. After that, cells were washed with PBS twice and samples are ready to mount in warm 500 μL medium for imaging. Imaging was taken on a Confocal Microscope (LSM710, ZEISS).

Immunoprecipitation

Following treatment with or without ES428, cells were harvested and washed twice with ice-cold PBS. Pellets were lysed in buffer (20 mmol/L Tris-HCl, pH 8.0; 150 mmol/L NaCl; 1 mmol/L EDTA; 0.1% NP-40; 10% glycerol; 1 mmol/L DTT; 1× protease inhibitor cocktail) and briefly sonicated. Lysates were clarified by centrifugation and the supernatants incubated overnight at 4°C with the appropriate primary antibody, followed by a 4 h incubation with Protein A/G magnetic beads (Beyotime, P2108) at 4 °C. Immunocomplexes were washed more than three times with wash buffer (1× PBS + 0.5% Tween-20, pH 7.4), eluted by boiling in 1× SDS sample buffer for 10 min, and analyzed by SDS-PAGE and immunoblotting.

RNA-seq analysis

Total RNA was extracted using Trizol reagent following the manufacturer's instructions. RNA purity and concentration were assessed with a NanoDrop 2000 spectrophotometer (Thermo Scientific, USA), and integrity was verified using an Agilent 2100 Bioanalyzer (Agilent Technologies, USA). Libraries were prepared with the TruSeq Stranded mRNA LT Sample Prep Kit (Illumina, USA) according to the manufacturer's protocol. Transcriptome sequencing and analysis were performed by OE Biotech Co., Ltd. (Shanghai, China). RNA-seq reads were mapped using Hisat2 with default settings. Transcript assembly and transcript abundance quantification were performed using feature Counts. Differential expression analysis was performed using DESeq2 R package. KEGG, GSEA and GO pathway enrichment analysis were performed using ClusterProfiler R package.

Metabolic profiling analysis

The non-targeted metabolomic profiling was conducted using liquid chromatography coupled with mass spectrometry (LC–MS) by OE Biotech Co., Ltd. (Shanghai, China).

Sample preparation was performed as follows. 20 μL of L-2-chlorophenylalanine (0.3 mg/mL in MeOH) and 20 μL of lyso-PC 17:0 (0.01 mg/mL in MeOH) were added as internal standards. Pre-chilled methanol–water (4:1, *v/v*; total 1 mL) was introduced in two aliquots, followed by 200 μL chloroform. The mixture was homogenized by pipette dispersion, then sonicated in an ice-water bath for 6 min. All liquid was transferred to centrifuge tubes and sonicated for an additional 20 min. After centrifugation (13,000 rpm, 10 min, 4 °C), 1 mL of supernatant was collected and evaporated to dryness. The residue was reconstituted in 400 μL methanol-water (1:4, *v/v*), vortexed for 30 s, and sonicated for 2 min. Following a second centrifugation (13,000 rpm, 10 min, 4 °C), 150 μL of the clear supernatant was filtered through a 0.22 μm filter into LC vials and stored at -80 °C until analysis. Quality control samples were prepared by pooling equal aliquots of all extracts and processed identically.

Chromatographic separation was performed on an ACQUITY UPLC BEH C18 column (100 × 2.1 mm, 1.7 μm) using an AB ExionLC UPLC system coupled to a Q Exactive high-resolution mass spectrometer. The binary gradient elution system consisted of (A) 0.1% (*v/v*) formic acid dissolved in water and (B) 0.1% formic acid (*v/v*) dissolved in acetonitrile and separation was achieved using the following gradient: 0 min, 5% B; 1.5 min, 5% B; 3 min, 30% B; 7 min, 60% B; 9 min, 90% B; 11 min, 100% B; 13 min, 100% B; 13.2 min, 5% B; 16 min, 5% B. The flow rate was at 0.35 mL/min and column temperature was at 40 ℃. All the samples were kept at 4 ℃ during the detection, the injection volume was at 5 μL each time, and the mass range was from m/z 100 to 1000. The resolution was set at 70,000 for the full MS scans and 17,500 for HCD MS/MS scans, and the Collision energy was set at 10, 20 and 40 eV. The mass spectrometer operated as follows: spray voltage, 3500 V at negative and positive mode; capillary temperature, 320 ℃; sheath gas flow rate, 40 arbitrary units; auxiliary gas flow rate, 10 arbitrary units. The QCs were injected at regular intervals (every 6 samples) throughout the analytical run to provide a set of data from which repeatability can be assessed, and were used to evaluate the stability of the mass spectrometer system during samples detection. The acquired LC-MS raw data were analyzed by the progqenesis QI software (Waters Corporation). Data were scaled and then centered on the mean. The principal component analysis (PCA), partial least-square discriminant analysis (PLS–DA), and orthogonal partial least-square discriminant analysis (OPLS–DA) were used to build the models. Finally, statistically significant metabolites were defined as those with P values < 0.05 and VIP values > 1.

Target Fishing

To identify potential targets for ES428, we employed MAI-TargetFisher, an in-house computational method designed for genome-wide target searching with integration of ligand

similarity-based and structure-based target identification process. For the target identification, we utilized GeminiMO[13] for ligand similarity searching, the 30K pocket cluster database for protein clusters, and Glide[8] for reverse docking calculation. The SMILES representation of the small molecule served as the input for our calculations. Each target's potential was assessed based on the docking scores obtained from Glide in this study, and targets with docking scores lower than -8.0 were considered as positive targets with a high binding potential. Additionally, to eliminate any false positives, we excluded three targets that are known experimental targets of the reference molecule noscapine showing no differentiation-promoting activity, according to the BindingDB database. These excluded targets were solute carrier family 22 member 2, tubulin beta-3 chain, and multidrug and toxin extrusion protein 1.

Statistical analysis

The data are presented as the mean ± SD and n represents the number of biological replicates, unless otherwise stated. All cellular experiments consisted of a minimum of three independent replicates and were repeated at least three times. Statistical tests were performed using Microsoft Excel and GraphPad Prism Software version 10.0. For comparisons of the two groups, a two-tailed unpaired *t*-test was used. For comparisons of multiple groups, a one-way ANOVA analysis of variance was used. A Log-rank test was performed for survival curves. The levels of significance were set at $^{*}P<0.05$, $^{**}P<0.01$ and $^{***}P<0.001$. Details for each figure are included in the Figure Legends.

1.2 Chemical synthesis

General information

Unless stated otherwise, commercial reagents were used as received. Thin-layer chromatography (TLC) was conducted with 0.25 mm silica gel plates (Qingdao Haiyang Chemical China), and the compounds were visualized by exposure to UV light (254 nm) or by potassium permanganate staining. Flash chromatography was performed on silica gel 200–300 mesh (purchased from Qingdao Haiyang Chemical China) with commercial solvents (purchased from Adamas–beta®). Data for ^{1}H NMR spectra (recorded on a Bruker AM 400 Spectrometer at 400Hz) are reported as follows: chemical shift (δ ppm), multiplicity, coupling constant (Hz) and integration. Among these parameters, multiplicities were given as s (singlet), d (doublet), t (triplet), dd (double of doublets), and m (multiplet). ^{13}C NMR spectra (recorded on Bruker Spectrometers at 100 or 150 MHz) are reported in terms of chemical shift. High-resolution mass spectrometry (HRMS) was recorded on Waters LCT Premier XE spectrometer.

Synthesis of compounds a1–a6, a9, ES428 and a11.

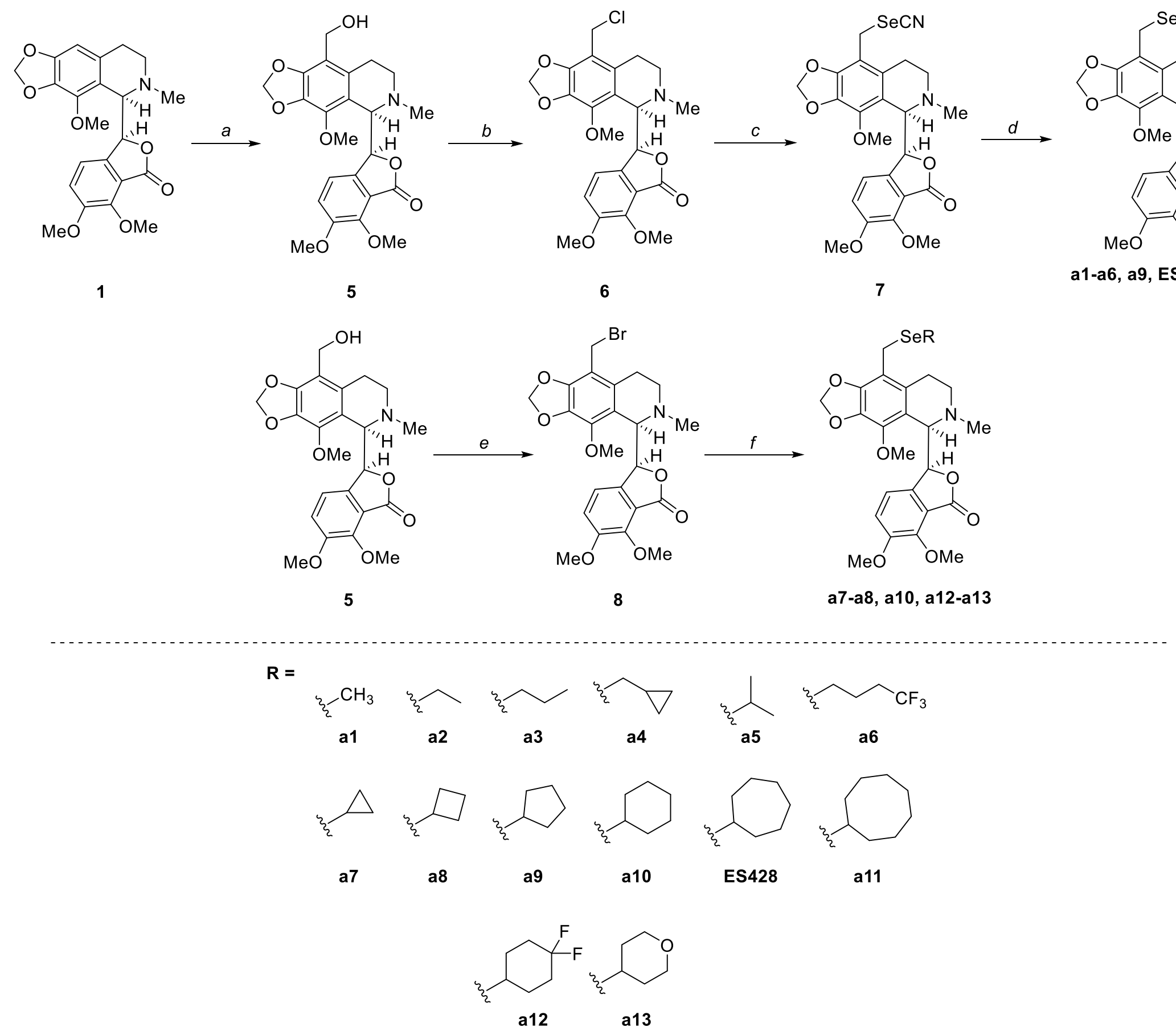

[a] Reagents and conditions: (a) paraformaldehyde, conc. HCl, 50 ºC, overnight; (b) $SOCl_2$, DCM, 0 ºC to rt, 2 h; (c) KSeCN, MeCN, 80 ºC, 5 min; (d) RBr, $NaBH_4$, EtOH, rt, 2 h, N_2; (e) PBr_3, DCM, 0 ºC to rt, 2 h; (f) RSeSeR, $NaBH_4$, EtOH, rt, 2 h, N_2.

(S)-6,7-dimethoxy-3-((R)-4-methoxy-6-methyl-9-(selenocyanatomethyl)-5,6,7,8-tetrahydro-[1,3] dioxolo[4,5-g]isoquinolin-5-yl)isobenzofuran-1(3H)-one (7). To a solution of noscapine **1** (1.80 g, 4.4 mmol) in concentrated hydrochloric acid (50 mL) was slowly added paraformaldehyde (4.00 g, 133.2 mmol). The reaction mixture was stirred at 50 °C overnight. After that, ammonium hydroxide was added to adjust the pH value to 10. The resulting solution was extracted with EtOAc, the combined organic layer was dried over anhydrous Na_2SO_4 and concentrated to give crude **5**. To a solution of **5** in DCM (44 mL) was slowly added thionyl chloride (0.59 mL) at 0 °C. Then it was stirred for 2 h. After that, the solvent was removed under vacuum to give the crude **6**. To a solution of **6** in MeCN (88 mL) was added KSeCN (0.95 g, 6.6 mmol) and the mixture was stirred at 80 °C for 5 min. Then the reaction was cooled to room temperature, diluted with water and extracted with EtOAc. The organic layer was dried over anhydrous Na_2SO_4, concentrated and purified via column chromatography on silica gel (PE/EtOAc = 1:1) to give **7** in 52% yield. ^{1}H NMR (400 MHz, Chloroform-*d*) δ 7.01 (d, J = 8.3 Hz, 1H), 6.20 (d, J = 8.2 Hz, 1H), 5.99 (d, J = 2.5 Hz, 2H), 5.52 (d, J = 4.2 Hz, 1H), 4.45 – 4.35 (m, 2H), 4.18 (d, J = 11.4 Hz, 1H), 4.09 (s, 3H), 4.03 (s, 3H), 3.86 (s, 3H), 2.76 – 2.67 (m, 1H), 2.54 (s, 3H), 2.53 – 2.47 (m, 1H), 2.47 – 2.37 (m, 1H), 1.97 – 1.83 (m, 1H). ^{13}C NMR (100 MHz, $CDCl_3$) δ 168.1, 152.3, 147.9, 147.8, 141.1, 140.9, 133.4, 130.4, 119.9, 118.5, 118.2, 117.6, 107.6, 101.5, 101.3, 81.5, 62.3, 60.9, 59.4, 56.8, 48.9, 45.8, 23.9, 23.3. HRMS (ESI-TOF) m/z calcd. for $C_{24}H_{25}N_2O_7Se$ $[M+H]^+$ 533.0821, found 533.0829.

General procedure for the synthesis of a1–a6, a9, ES428 and a11. Under N_2 atmosphere, $NaBH_4$ (15.1 mg, 0.4 mmol) was added slowly into a solution of **7** (106.3 mg, 0.2 mmol) in EtOH (1 mL). The resulting mixture was stirred for 30 min. Then alkyl bromide (0.4 mmol) was slowly added and the mixture was stirred at room temperature for 2 h. Then the reaction

was quenched with H_2O, extracted with EtOAc, and the organic layer was dried over anhydrous Na_2SO_4, concentrated and purified via column chromatography on silica gel (PE/EtOAc = 3:1) to yield **a1-a6, a9, ES428, a11.**

(S)-6,7-dimethoxy-3-((R)-4-methoxy-6-methyl-9-((methylselanyl)methyl)-5,6,7,8-tetrahydro-[1,3]dioxolo[4,5-g]isoquinolin-5-yl)isobenzofuran-1(3H)-one (a1). Light-yellow foam, 68% yield. ^{1}H NMR (400 MHz, $CDCl_3$) δ 6.89 (d, *J* = 8.3 Hz, 1H), 6.01 (d, *J* = 8.2 Hz, 1H), 5.88 (m, 2H), 5.46 (d, *J* = 4.2 Hz, 1H), 4.34 (d, *J* = 4.2 Hz, 1H), 4.02 (s, 3H), 3.94 (s, 3H), 3.79 (s, 3H), 3.66 – 3.58 (m, 3H), 2.65 – 2.55 (m, 1H), 2.47 (m, 4H), 2.33 – 2.25 (m, 1H), 1.97 (s, 3H), 1.85 – 1.78 (m, 1H). ^{13}C NMR (151 MHz, $CDCl_3$) δ 167.1, 151.2, 146.7, 145.7, 142.6, 140.3, 138.5, 132.2, 129.5, 128.1, 124.4, 124.4, 118.9, 117.4, 116.6, 110.3, 99.8, 80.7, 61.3, 59.9, 58.4, 55.8, 48.1, 44.9, 30.5, 28.7, 26.5, 22.3, 17.0, 13.1. HRMS (ESI-TOF) m/z calcd. for $C_{24}H_{28}NO_7Se$ $[M+H]^+$ 522.1026, found 522.1020.

(S)-3-((R)-9-((ethylselanyl)methyl)-4-methoxy-6-methyl-5,6,7,8-tetrahydro-[1,3]dioxolo[4,5-g]isoquinolin-5-yl)-6,7-dimethoxyisobenzofuran-1(3H)-one (a2). Light-yellow foam, 51% yield. ^{1}H NMR (400 MHz, $CDCl_3$) δ 6.96 (d, *J* = 8.2 Hz, 1H), 6.07 (d, *J* = 7.5 Hz, 1H), 5.95 (m, 2H), 5.52 (d, *J* = 3.9 Hz, 1H), 4.41 (d, *J* = 3.9 Hz, 1H), 4.09 (s, 3H), 4.01 (s, 3H), 3.86 (s, 3H), 3.72 (q, *J* = 11.8 Hz, 2H), 2.64 (q, *J* = 7.5 Hz, 3H), 2.54 (s, 4H), 2.37 (m, 1H), 1.82 (m, 1H), 1.44 (t, *J* = 7.5 Hz, 3H). ^{13}C NMR (151 MHz, $CDCl_3$) δ 168.2, 152.2, 147.7, 146.7, 141.2, 139.3, 133.3, 130.7, 120.1, 118.4, 117.84, 117.79, 112.2, 100.8, 81.9, 62.3, 61.0, 59.4, 56.8, 49.5, 46.2, 23.7, 18.2, 16.5, 15.9. HRMS (ESI-TOF) m/z calcd. for $C_{25}H_{30}NO_7Se$ $[M+H]^+$ 536.1182, found 536.1188.

(S)-3-((R)-9-((butylselanyl)methyl)-4-methoxy-6-methyl-5,6,7,8-tetrahydro-[1,3]dioxolo[4,5-g]isoquinolin-5-yl)-6,7-dimethoxyisobenzofuran-1(3H)-one (a3). Light-yellow foam, 60% yield. ^{1}H NMR (400 MHz, Chloroform-*d*) δ 6.98 (d, *J* = 8.3 Hz, 1H), 6.05 (d, *J* = 8.3 Hz, 1H), 5.99 – 5.92 (m, 2H), 5.52 (d, *J* = 4.2 Hz, 1H), 4.41 (d, *J* = 4.2 Hz, 1H), 4.09 (s, 3H), 4.02 (s, 3H), 3.86 (s, 3H), 3.80 – 3.67 (m, 2H), 3.27 – 3.12 (m, 1H), 2.72 – 2.59 (m, 2H), 2.59 – 2.47 (m, 4H), 2.43 – 2.31 (m, 1H), 1.92 – 1.76 (m, 2H), 1.75 – 1.60 (m, 1H), 1.55 – 1.36 (m, 5H). ^{13}C NMR (151 MHz, $CDCl_3$) δ 168.2, 152.2, 147.7, 146.7, 141.2, 139.3, 133.3, 130.7, 120.0, 118.4, 117.8, 112.3, 100.8, 81.9, 62.3, 61.0, 59.4, 56.8, 49.5, 46.2, 32.8, 29.7, 24.6, 23.6, 23.2, 16.8, 13.6, 1.0. HRMS (ESI-TOF) m/z calcd. for $C_{27}H_{34}NO_7Se$ $[M+H]^+$ 564.1495, found 564.1501.

(S)-3-((R)-9-(((cyclopropylmethyl)selanyl)methyl)-4-methoxy-6-methyl-5,6,7,8-tetrahydro-[1,3]dioxolo[4,5-g]isoquinolin-5-yl)-6,7-dimethoxyisobenzofuran-1(3H)-one (a4). Light-yellow foam, 70% yield. ^{1}H NMR (400 MHz, $CDCl_3$) δ 6.92 (d, *J* = 8.3 Hz, 1H), 6.02 (d, *J* = 7.2 Hz, 1H), 5.89 (dd, *J* = 7.6, 1.3 Hz, 2H), 5.47 (d, *J* = 3.1 Hz, 1H), 4.35 (d, *J* = 4.0 Hz, 1H), 4.02 (s, 3H), 3.93 (s, 3H), 3.79 (s, 3H), 3.70 (q, *J* = 11.7 Hz, 2H), 2.61 (m, 1H), 2.53 (d, *J* = 7.2 Hz, 2H), 2.47 (s, 3H), 2.30 (m, 1H), 1.78 (m, 2H), 1.06 – 0.96 (m, 1H), 0.59 – 0.48 (m, 2H), 0.21 – 0.10 (m, 2H). ^{13}C NMR (151 MHz, $CDCl_3$) δ 167.1, 151.2, 146.6, 145.6, 140.1, 138.3, 132.3, 119.0, 117.5, 116.8, 111.3, 99.8, 80.8, 61.3, 59.9, 58.4, 55.8, 48.4, 45.1, 29.5, 15.9, 10.9, 5.7. HRMS (ESI-TOF) m/z calcd. for $C_{27}H_{32}NO_7Se$ $[M+H]^+$ 562.1339, found 564.1344.

(S)-3-((R)-9-((isopropylselanyl)methyl)-4-methoxy-6-methyl-5,6,7,8-tetrahydro-[1,3]dioxolo[4,5-g]isoquinolin-5-yl)-6,7-dimethoxyisobenzofuran-1(3H)-one (a5). Light-yellow foam, 43% yield. ^{1}H NMR (400 MHz, Chloroform-*d*) δ 6.97 (d, *J* = 8.2 Hz, 1H), 6.06 (d, *J* = 8.2 Hz, 1H), 5.96 (d, *J* = 8.1, 1.4 Hz, 2H), 5.52 (d, *J* = 4.2 Hz, 1H), 4.41 (d, *J* = 4.2 Hz, 1H), 4.08 (s, 3H), 4.00 (s, 3H), 3.85 (s, 3H), 3.79 – 3.67 (m, 2H), 3.24 – 3.13 (m, 1H), 2.70 – 2.61 (m, 1H), 2.58 – 2.54 (m, 1H), 2.53 (s, 3H), 2.41 – 2.31 (m, 1H), 1.88 – 1.84 (m, 1H), 1.49 – 1.43 (m, 6H). ^{13}C NMR (151 MHz, $CDCl_3$) δ 168.2, 152.2, 147.6, 146.6, 141.2, 139.3, 133.3, 130.7, 120.0, 118.4, 117.8, 112.2, 100.8, 81.9, 62.3, 60.9, 59.4, 56.8, 49.5, 46.2, 32.8, 24.6, 23.7, 23.2, 16.8, 13.6. HRMS (ESI-TOF) m/z calcd. for $C_{26}H_{32}NO_7Se$ $[M+H]^+$ 550.1339, found 550.1348.

(S)-6,7-dimethoxy-3-((R)-4-methoxy-6-methyl-9-(((4,4,4-trifluorobutyl)selanyl)methyl)-5,6,7,8-tetrahydro-[1,3]dioxolo[4,5-g]isoquinolin-5-yl)isobenzofuran-1(3H)-one (a6). Light-yellow foam, 40% yield. ^{1}H NMR (400 MHz, $CDCl_3$) δ 6.92 (d, *J* = 8.2 Hz, 1H), 6.17 (d, *J* = 7.5 Hz, 1H), 5.87 (m, 2H), 5.52 (d, *J* = 3.9 Hz, 1H), 4.38 (d, *J* = 3.9 Hz, 1H), 4.01 (s, 3H), 3.87 (s, 3H), 3.80 (s, 3H), 3.65 (s, 2H), 2.73 (s, 1H), 2.57 (t, *J* = 7.2 Hz, 2H), 2.48 (s, 3H), 2.40 (s, 1H), 2.22 – 2.07 (m, 2H), 1.92 – 1.86 (m, 2H), 0.81 (m, 2H). ^{13}C NMR (151 MHz, $CDCl_3$) δ 167.1, 151.2, 146.7, 145.7, 140.3, 138.5, 132.2, 129.5, 126.9, 125.0, 118.9, 117.4, 116.9, 116.7, 110.5, 99.8, 80.7, 61.3, 60.0, 58.4, 55.8, 48.1, 44.9, 32.78 (q, *J* = 28.3 Hz), 28.7, 22.4, 22.0 (d, *J* = 3.4 Hz), 21.9, 15.8. HRMS (ESI-TOF) m/z calcd. for $C_{27}H_{31}F_3NO_7Se$ $[M+H]^+$ 618.1212, found 618.1208.

(S)-3-((R)-9-((cyclopentylselanyl)methyl)-4-methoxy-6-methyl-5,6,7,8-tetrahydro-[1,3]dioxolo[4,5-g]isoquinolin-5-yl)-6,7-dimethoxyisobenzofuran-1(3H)-one (a9). Light-yellow foam, 47% yield. ^{1}H NMR (400 MHz, $CDCl_3$) δ 6.97 (d, *J* = 8.3 Hz, 1H), 6.04 (d, *J* = 8.1 Hz, 1H), 5.95 (m, 2H), 5.52 (d, *J* = 3.7 Hz, 1H), 4.41 (d, *J* = 4.1 Hz, 1H), 4.09 (s, 3H), 4.01 (s, 3H), 3.85 (s, 3H), 3.78 – 3.64 (m, 2H), 3.36 – 3.26 (m, 1H), 2.63 (s, 1H), 2.52 (d, *J* = 8.0 Hz, 4H), 2.35 (t, *J* = 7.7 Hz, 1H), 2.18 – 2.00 (m, 2H), 1.89 – 1.53 (m, 10H). ^{13}C NMR (151 MHz, $CDCl_3$) δ 168.2, 152.2, 147.6, 146.5, 141.1, 139.2, 133.4, 130.7, 120.0, 118.5, 117.9, 117.7, 112.3, 100.8, 81.9, 62.3, 60.9, 59.4, 56.8, 49.5, 46.2, 38.5, 34.6, 34.4, 25.10, 25.06, 23.7, 17.1. HRMS (ESI-TOF) m/z calcd. for $C_{28}H_{34}NO_7Se$ $[M+H]^+$ 576.1495, found 576.1503.

(S)-3-((R)-9-((cycloheptylselanyl)methyl)-4-methoxy-6-methyl-5,6,7,8-tetrahydro-[1,3]dioxolo[4,5-g]isoquinolin-5-yl)-6,7-dimethoxyisobenzofuran-1(3H)-one (ES428). Light-yellow foam, 42% yield. ^{1}H NMR (400 MHz, $CDCl_3$) δ 6.97 (d, *J* = 8.3 Hz, 1H), 6.03 (d, *J* = 8.2 Hz, 1H), 5.96 (m, 2H), 5.52 (d, *J* = 4.1 Hz, 1H), 4.40 (d, *J* = 4.2 Hz, 1H), 4.09 (s, 3H), 4.02 (s, 3H), 3.85 (s, 3H), 3.70 (m, 2H), 3.19 – 3.09 (m, 1H), 2.68 – 2.59 (m, 1H), 2.53 (s, 4H), 2.40 – 2.29 (m, 1H), 2.19 – 2.07 (m, 2H), 1.87 – 1.75 (m, 1H), 1.70 (m, 4H), 1.55 (m, 3H), 1.53 – 1.39 (m, 3H); ^{13}C NMR (100 MHz, $CDCl_3$) δ 168.3, 152.1, 147.6, 146.5, 141.1, 139.2, 133.4, 130.8, 120.0, 118.5, 117.9, 117.8, 112.3, 100.8, 81.9, 63.3, 62.3, 60.9, 59.5, 56.8, 49.6, 46.3, 44.5, 41.7, 36.1, 36.1, 28.1, 28.0, 26.9, 23.7, 16.7. HRMS (ESI-TOF) m/z calcd. for $C_{30}H_{38}NO_7Se$ $[M+H]^+$ 604.1808, found 604.1802.

(S)-3-((R)-9-((cyclooctylselanyl)methyl)-4-methoxy-6-methyl-5,6,7,8-tetrahydro-[1,3]dioxolo[4,5-g]isoquinolin-5-yl)-6,7-dimethoxyisobenzofuran-1(3H)-one (a11). Light-yellow foam, 13% yield. ^{1}H NMR (400 MHz, Chloroform-*d*) δ 6.98 (d, *J* = 8.3 Hz, 1H), 6.04 (d, *J* = 8.2 Hz, 1H), 5.96 (d, *J* = 5.4 Hz, 2H), 5.53 (d, *J* = 4.2 Hz, 1H), 4.41 (d, *J* = 4.2 Hz, 1H), 4.09 (s, 3H), 4.02 (s, 3H), 3.86 (s, 3H), 2.59 – 2.48 (m, 1H), 3.73 (q, 2H), 3.29 – 3.19 (m, 1H), 2.69 – 2.61 (m, 1H), 2.54 (s, 3H), 2.40 – 2.29 (m, 1H), 2.14 – 2.04 (m, 2H), 1.87 – 1.68 (m, 6H), 1.60 – 1.44 (m, 7H). ^{13}C NMR (151 MHz, $CDCl_3$) δ 168.2, 152.2, 147.6, 146.5, 141.1, 139.2, 133.4, 130.8, 120.0, 118.5, 117.9, 112.3, 100.8, 81.9, 62.3, 60.9, 59.5, 56.8, 49.6, 46.2, 41.4, 33.5, 27.1, 26.1, 26.0, 25.9, 23.8, 16.6. HRMS (ESI-TOF) m/z calcd. for $C_{31}H_{40}NO_7Se$ $[M+H]^+$ 618.1965, found 618.1970.

General procedure for the synthesis of a7-a8, a10, a12-a13. To a solution of **5** (1.62 g, 3.7 mmol) in DCM (37 mL) was slowly added phosphorus tribromide (1 g, 3.7 mmol) at 0 °C. Then it was stirred for 2 h, the solvent was removed under vacuum to produce the crude **8**. Under N_2 atmosphere, $NaBH_4$ (140.0 mg, 3.7 mmol) was added portion wise into a solution of diselenide (3.7 mmol) in EtOH (18.5 mL). The resulting mixture was stirred for 30 min. Then crude **8** was slowly added and the mixture was stirred at room temperature for another 2 h. Then the reaction was quenched with H_2O, extracted with EtOAc, and the organic layer was dried over anhydrous Na_2SO_4, concentrated and purified via column chromatography on silica gel (PE/EtOAc = 3:1) to yield **a7-a8, a10, a12-a13**.

(S)-3-((R)-9-((cyclopropylselanyl)methyl)-4-methoxy-6-methyl-5,6,7,8-tetrahydro-[1,3]dioxolo[4,5-g]isoquinolin-5-yl)-6,7-dimethoxyisobenzofuran-1(3H)-one (a7). Light-yellow foam, 16% yield. ^{1}H NMR (400 MHz, Chloroform-*d*) δ 6.95 (d, *J* = 8.2 Hz, 1H), 6.05

(d, J = 8.2 Hz, 1H), 5.95 (d, J = 4.2 Hz, 1H), 5.52 (d, J = 4.2 Hz, 1H), 4.40 (d, J = 4.2 Hz, 1H), 4.08 (s, 3H), 3.99 (s, 3H), 3.85 (s, 3H), 3.80 (q, 2H), 2.70 – 2.61 (m, 1H), 2.60 – 2.49 (m, 1H), 2.52 (s, 3H), 2.40 – 2.31 (m, 1H), 2.05 – 1.97 (m, 1H), 1.86 – 1.76 (m, 1H), 0.99 – 0.89 (m, 2H), 0.66 – 0.57 (m, 2H). ^{13}C NMR (151 MHz, $CDCl_3$) δ 168.2, 152.2, 147.6, 146.7, 141.2, 139.3, 133.4, 130.5, 120.0, 118.4, 117.8, 112.2, 100.8, 81.8, 62.3, 61.0, 59.4, 56.8, 49.5, 46.1, 29.7, 23.7, 18.5, 8.2, 8.0, 5.2. HRMS (ESI-TOF) m/z calcd. for $C_{26}H_{30}NO_7Se$ $[M+H]^+$ 548.1182, found 548.1190.

(S)-3-((R)-9-((cyclobutylselanyl)methyl)-4-methoxy-6-methyl-5,6,7,8-tetrahydro-[1,3]dioxolo[4,5-g]isoquinolin-5-yl)-6,7-dimethoxyisobenzofuran-1(3H)-one (a8). Light-yellow foam, 26% yield. ^{1}H NMR (400 MHz, Chloroform-*d*) δ 6.97 (d, J = 8.2 Hz, 1H), 6.06 (d, J = 8.2 Hz, 1H), 5.97 (d, J = 4.5 Hz, 1H), 5.53 (d, J = 4.2 Hz, 1H), 4.41 (d, J = 4.2 Hz, 1H), 4.09 (s, 3H), 4.01 (s, 3H), 3.86 (s, 3H), 3.67 (q, 2H), 2.70 – 2.62 (m, 1H), 2.54 (s, 3H), 2.53 – 2.48 (m, 1H), 2.47 – 2.32 (m, 3H), 2.22 – 2.11 (m, 2H), 2.10 – 2.02 (m, 1H), 2.01 – 1.93 (m, 1H), 1.86 – 1.75 (m, 2H). ^{13}C NMR (151 MHz, $CDCl_3$) δ 168.2, 152.2, 147.6, 146.5, 141.2, 139.3, 133.4, 130.6, 120.0, 118.4, 117.8, 117.7, 112.2, 100.8, 81.8, 62.3, 61.0, 59.4, 56.8, 49.5, 46.2, 33.2, 31.8, 31.6, 23.7, 20.1, 16.4. HRMS (ESI-TOF) m/z calcd. for $C_{27}H_{32}NO_7Se$ $[M+H]^+$ 562.1339, found 562.1349.

(S)-3-((R)-9-((cyclohexylselanyl)methyl)-4-methoxy-6-methyl-5,6,7,8-tetrahydro-[1,3]dioxolo[4,5-g]isoquinolin-5-yl)-6,7-dimethoxyisobenzofuran-1(3H)-one (a10). Light-yellow foam, 19% yield. ^{1}H NMR (400 MHz, Chloroform-*d*) δ 6.91 (d, J = 8.3 Hz, 1H), 5.98 (d, J = 8.2 Hz, 1H), 5.94 – 5.84 (m, 2H), 5.46 (d, J = 4.1 Hz, 1H), 4.34 (d, J = 4.2 Hz, 1H), 4.02 (s, 3H), 3.94 (s, 3H), 3.79 (s, 3H), 3.65 (q, J = 11.5 Hz, 2H), 2.99 – 2.88 (m, 1H), 2.62 – 2.54 (m, 1H), 2.52 – 2.47 (m, 1H), 2.46 (s, 3H), 2.33 – 2.24 (m, 1H), 2.05 – 1.94 (m, 2H), 1.89 – 1.80 (m, 1H), 1.79 – 1.63 (m, 3H), 1.56 (q, J = 5.2, 4.5 Hz, 1H), 1.51 – 1.41 (m, 2H), 1.33 – 1.24 (m, 2H). ^{13}C NMR (151 MHz, $CDCl_3$) δ 168.2, 147.6, 146.5, 141.2, 139.3, 133.4, 130.8, 120.0, 118.5, 117.9, 117.8, 112.3, 100.8, 81.9, 62.3, 60.9, 59.4, 56.8, 49.6, 46.2, 39.9, 34.6, 34.6, 29.7, 27.0, 26.9, 25.8, 23.7, 15.7. HRMS (ESI-TOF) m/z calcd. for $C_{29}H_{35}NO_7Se$ $[M+H]^+$ 590.1652, found 590.1661.

(S)-3-((R)-9-(((4,4-difluorocyclohexyl)selanyl)methyl)-4-methoxy-6-methyl-5,6,7,8-tetrahydro-[1,3]dioxolo[4,5-g]isoquinolin-5-yl)-6,7-dimethoxyisobenzofuran-1(3H)-one (a12). Light-yellow foam, 14% yield. ^{1}H NMR (400 MHz, Chloroform-*d*) δ 6.98 (d, J = 8.3 Hz, 1H), 6.15 (d, J = 8.2 Hz, 2H), 5.98 – 5.92 (m, 2H), 5.52 (d, J = 4.3 Hz, 1H), 4.39 (d, J = 4.4 Hz, 1H), 4.09 (s, 3H), 4.00 (s, 3H), 3.87 (s, 3H), 3.75 (s, 2H), 3.13 – 3.02 (m, 1H), 2.76 – 2.66 (m, 1H), 2.60 – 2.54 (m, 1H), 2.53 (s, 3H), 2.44 – 2.33 (m, 1H), 2.18 – 2.06 (m, 3H), 1.94 – 1.79 (m, 4H). ^{13}C NMR (151 MHz, $CDCl_3$) δ 168.1, 152.2, 147.7, 146.6, 141.3, 139.5, 133.3, 130.5, 122.6, 120.0, 118.4, 117.7, 111.6, 100.8, 100.5, 81.7, 62.3, 61.0, 59.4, 56.8, 49.2, 45.9, 38.6, 36.1, 33.5 (t, J = 23.7 Hz), 30.1 (q, J = 5.2 Hz), 29.7, 29.3, 25.5, 23.4, 22.7, 16.4, 14.1, 10.9. HRMS (ESI-TOF) m/z calcd. for $C_{29}H_{34}F_2NO_7Se$ $[M+H]^+$ 626.1463, found 626.1470.

(S)-6,7-dimethoxy-3-((R)-4-methoxy-6-methyl-9-(((tetrahydro-2H-pyran-4-yl)selanyl)methyl)-5,6,7,8-tetrahydro-[1,3]dioxolo[4,5-g]isoquinolin-5-yl)isobenzofuran-1(3H)-one (a13). Light-yellow foam, 28% yield. ^{1}H NMR (400 MHz, Chloroform-*d*) δ 6.98 (d, J = 8.2 Hz, 1H), 6.12 (d, J = 8.2 Hz, 1H), 5.95 (d, J = 4.9 Hz, 2H), 5.51 (d, J = 4.3 Hz, 1H), 4.39 (d, J = 4.4 Hz, 1H), 4.09 (s, 3H), 4.00 (s, 3H), 4.00 – 3.91 (m, 3H), 3.86 (s, 3H), 3.75 (s, 2H), 3.51 – 3.39 (m, 2H), 3.22 – 3.11 (m, 1H), 2.74 – 2.64 (m, 1H), 2.60 – 2.53 (m, 1H), 2.53 (s, 3H), 2.43 – 2.33 (m, 1H), 2.07 – 1.95 (m, 2H), 1.91 – 1.75 (m, 3H). MS (ESI-TOF) m/z calcd. for $C_{28}H_{33}NO_8Se$ $[M+H]^+$ 592.1, found 592.14.

Synthesis of compounds a14 and a15.

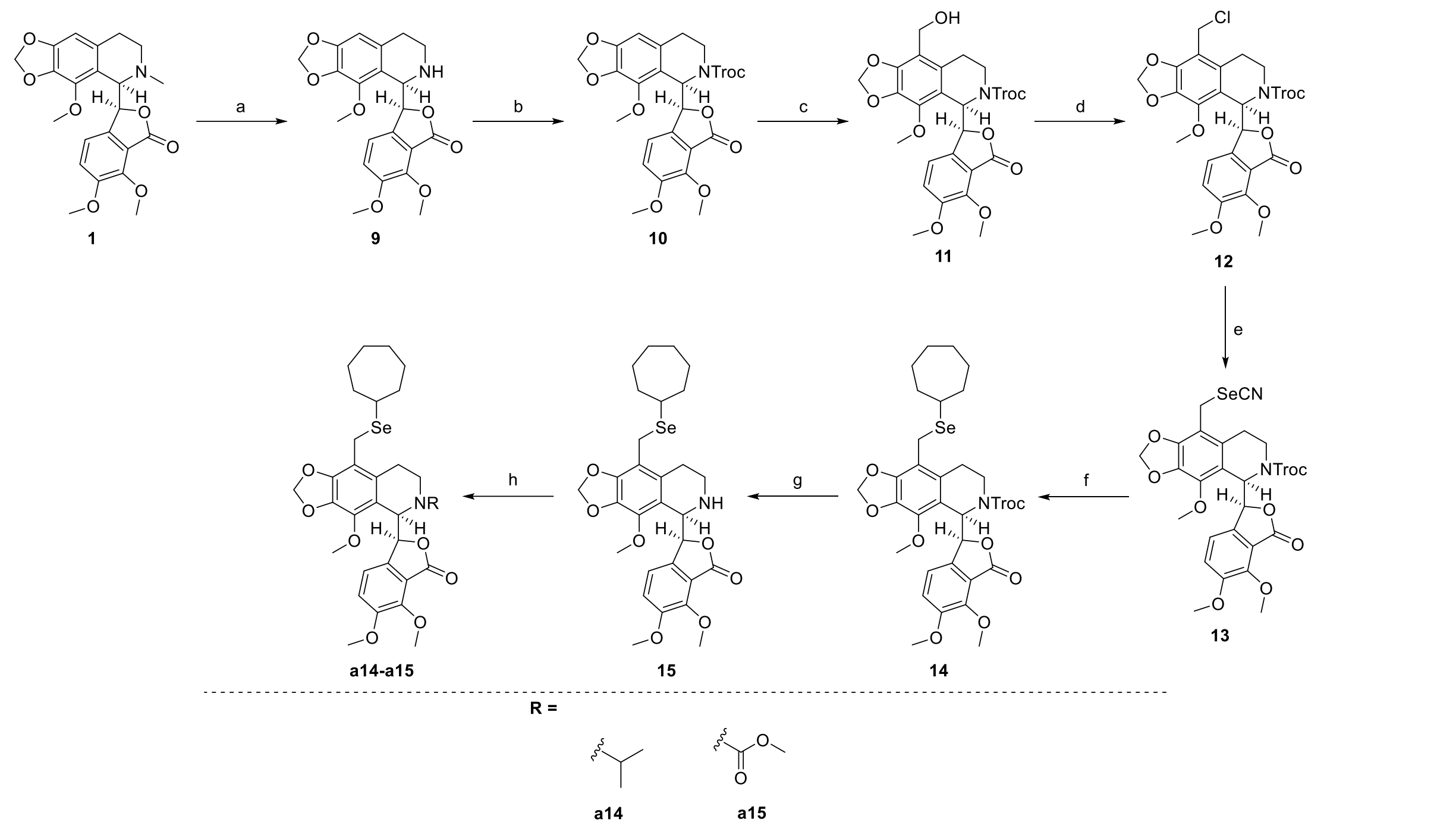

Reagents and conditions: (a): (i) *m*-CPBA, $CHCl_3$, -5 ℃, 1 h; (ii) $FeSO_4 \cdot 7H_2O$, MeOH, -5 ℃, 8 h, 60%, 2 steps; (b) TrocCl, K_2CO_3, MeCN, rt, 1 h; (c) Formaldehyde (37%-40%), conc. HCl, 1,4-dioxane, 50 ℃, overnight; (d) $SOCl_2$, DCM, 0 ℃ to rt, 2 h; (e) KSeCN, MeCN, 80 ℃, 5 min; (f) 1-Bromocycloheptaner, $NaBH_4$, EtOH, rt, 2 h, N_2; (g) Zn dust, AcOH, rt, 12 h, N_2, 12%, 6 steps; (h) for **a15**, Acetone, $Na(CN)BH_3$, AcOH, MeOH, rt, 16 h, 61%; for **a16**, MeOH, Bis(trichloromethyl) carbonate, TEA, DCM, rt, 12 h, 54%.

(S)-6,7-dimethoxy-3-((R)-4-methoxy-5,6,7,8-tetrahydro-[1,3]dioxolo[4,5-g]isoquinolin-5-yl)isobenzofuran-1(3H)-one (9). Compound **9** was prepared as described previously with minor modifications[14]. To a solution of noscapine **1** (4.13 g, 10.0 mmol) in $CHCl_3$ (100 mL) was added *m*-CPBA (3.45 g, 20.0 mmol) portion-wise under -5 °C. The reaction was kept at that temperature and stirred for 1 h. After that, cold $CHCl_3$ and cold *i*-PrOH were added and the resultant solution was washed with cold 10% NaOH, cold water and finally with 10% HCl. The organic layer was dried over anhydrous Na_2SO_4 and evaporated in vacuo to yield the N-oxide as a hydrochloride salt in the form of a light-orange foam. The crude mixture of noscapine-N-oxide·HCl was dissolved with MeOH (100 mL) and the solution was stirred at -5 °C, followed by the addition of $FeSO_4 \cdot 7H_2O$ (5.56 g, 20.0 mmol). And the mixture was stirred until noscapine-N-oxide·HCl was completely consumed. The solvent was removed in vacuo to yield crude product which was then taken up in $CHCl_3$ and washed with aqueous acidic 0.1M EDTA and subsequently 1M NaOH. The organic layer was dried over anhydrous Na_2SO_4, evaporated under reduced pressure and purified via column chromatography on silica gel (PE/EtOAc = 2:1) to yield **9** (2.40 g, 60%) as a light-yellow form. ^1H NMR (400 MHz, Chloroform-*d*) δ 6.93 (d, J = 8.3 Hz, 1H), 6.33 (s, 1H), 6.00 – 5.92 (m, 3H), 5.91 (d, J = 4.0 Hz, 1H), 4.85 (d, J = 4.0 Hz, 1H), 4.09 (s, 3H), 4.06 (s, 3H), 3.84 (s, 3H), 2.69 – 2.59 (m, 1H), 2.54 – 2.42 (m, 1H), 2.36 – 2.26 (m, 1H), 2.26 – 2.10 (m, 1H). Spectral properties matched reported values[14].

(S)-3-((R)-9-((cycloheptylselanyl)methyl)-4-methoxy-5,6,7,8-tetrahydro-[1,3]dioxolo[4,5-g]isoquinolin-5-yl)-6,7-dimethoxyisobenzofuran-1(3H)-one (15). To a solution of **9** (4 g, 10 mmol) in MeCN (100 mL) was added 2,2,2-trichloroethyl chloroformate (3.2 g, 15 mmol) dropwise following by the addition of K_2CO_3 (2.1 g, 15 mmol). The solution was stirred at room temperature for 1 h. The solvent was removed under vacuum and the residue was dissolved with water, extracted with dichloromethane, washed with brine, dried over Na_2SO_4 and concentrated to afford crude **10** as a brown oil. The crude product **10** was dissolved with

1,4-dioxane (250 mL), warmed to 50 °C and vigorous stirred. Concentrated hydrochloric acid (250 mL) and formaldehyde (37%-40% solution in water, 750 mL) were added respectively. The resulting mixture was stirred overnight kept 50 °C. After that, ammonium hydroxide was added to adjust the pH value to 10. The resulting solution was extracted with EtOAc, the organic layer was dried over anhydrous Na_2SO_4 and concentrated. The residue was dissolved with DCM (100 mL) and thionyl chloride (1.40 mL) was added portion-wise at 0 °C. Then it was stirred for 2 h, the solvent was removed under vacuum to produce crude **12**. To a solution of **12** in MeCN (200 mL) was added KSeCN (2.2 g, 15 mmol) and the mixture was stirred at 80 °C for 5 min. Then the reaction was cooled to room temperature, diluted with water and extracted with EtOAc. The organic layer was dried over anhydrous Na_2SO_4 and concentrated to afford a crude **13** as a brown foam. Under N_2 atmosphere, $NaBH_4$ (567 mg, 15 mmol) was added slowly into a solution of **13** in EtOH (100 mL). After the mixture was stirred for 30 min, bromo-cycloheptan (2.66 g, 15 mmol) was slowly added and the mixture was stirred at room temperature for 2 h. Then the reaction was quenched with H_2O, extracted with EtOAc, and the organic layer was dried over anhydrous Na_2SO_4 and concentrated to afford a crude **14** as a yellow foam. Then acetic acid (50 mL) was added to a Shrek Tube charged with the crude **14** and zinc dust (654.0 mg, 10 mmol) under N_2 atmosphere and stirred at room temperature overnight. The reaction mixture was quenched by saturated aqueous $NaHCO_3$, extracted with EtOAc, washed with brine, dried over Na_2SO_4, concentrated and purified via column chromatography on silica gel (PE/EtOAc = 3:1) to yield **15** (706.3 mg, 12%) as a yellow foam. ^{1}H NMR (400 MHz, Chloroform-*d*) δ 6.93 (d, *J* = 8.3 Hz, 1H), 6.03 – 5.96 (m, 2H), 5.90 (d, *J* = 8.2 Hz, 1H), 5.85 (d, *J* = 4.2 Hz, 1H), 4.85 (d, *J* = 4.1 Hz, 1H), 4.09 (s, 3H), 4.06 (s, 3H), 3.84 (s, 3H), 3.80 – 3.60 (m, 2H), 3.20 – 3.09 (m, 1H), 2.73 – 2.63 (m, 1H), 2.60 – 2.48 (m, 1H), 2.34 – 2.10 (m, 5H), 1.79 – 1.64 (m, 4H), 1.59 – 1.36 (m, 5H). ^{13}C NMR (150 MHz, $CDCl_3$) δ 170.8, 168.6, 152.2, 148.0, 146.6, 141.2, 130.5, 118.7, 117.8, 117.7, 113.1, 100.9, 80.7, 62.3, 59.6, 56.7, 56.7, 52.8, 41.7, 41.7, 39.2, 38.1, 36.1, 36.1, 35.2, 28.0, 26.9, 26.9, 26.9, 25.4, 21.6, 16.8. HRMS (ESI-TOF) m/z calcd. for $C_{29}H_{35}NO_7Se$ $[M+H]^+$ 590.1652, found 590.1665.

(S)-3-((R)-9-((cycloheptylselanyl)methyl)-6-isopropyl-4-methoxy-5,6,7,8-tetrahydro-[1,3]dioxolo[4,5-g]isoquinolin-5-yl)-6,7-dimethoxyisobenzofuran-1(3H)-one (a14). To a solution of **15** (117 mg, 0.2 mmol) and acetone (23.2 mg, 0.4 mmol) in MeOH (2 mL) was added $Na(CN)BH_3$ (25.1 mg, 0.4 mmol) and 2.5 μL AcOH. After stirred at room temperature for 16 h, the reaction was quenched by water, extracted with EtOAc, washed with brine, dried over Na_2SO_4, concentrated and purified via column chromatography on silica gel (PE/EtOAc = 3: 1) to product **a14** with 61% yield. ^{1}H NMR (400 MHz, Chloroform-*d*) δ 6.99 (d, *J* = 8.3 Hz, 1H), 6.13 (d, *J* = 8.2 Hz, 1H), 6.00 – 5.93 (m, 2H), 5.41 (d, *J* = 4.6 Hz, 1H), 4.62 (d, *J* = 4.7 Hz, 1H), 4.10 (s, 3H), 4.05 (s, 3H), 3.87 (s, 3H), 3.77 – 3.65 (m, 2H), 3.28 – 3.09 (m, 2H), 2.66 – 2.36 (m, 3H), 2.19 – 2.07 (m, 2H), 1.79 – 1.65 (m, 5H), 1.60 – 1.39 (m, 6H), 1.05 (d, *J* = 6.7 Hz, 3H), 0.94 (d, *J* = 6.5 Hz, 3H). MS (ESI-TOF) m/z calcd. for $C_{32}H_{41}NO_7Se$ $[M+H]^+$ 632.2, found 632.2.

Methyl(R)-9-((cycloheptylselanyl)methyl)-5-((S)-4,5-dimethoxy-3-oxo-1,3-dihydroisobenzofuran-1-yl)-4-methoxy-7,8-dihydro-[1,3]dioxolo[4,5-g]isoquinoline-6(5H)-carboxylate (a15). To a solution of **15** (117 mg, 0.2 mmol) and bis(trichloromethyl) carbonate (89.0 mg, 0.3 mmol) in dichloromethane (2 mL) was added triethylamine (61.0 mg, 0.6 mmol) portion-wise. After the solution was stirred at room temperature for 1 h, MeOH (2 mL) was added and the resulting mixture was kept stirring for another 12 h. The reaction was quenched by water, extracted with dichloromethane, washed with brine, dried over Na_2SO_4, concentrated and purified via column chromatography on silica gel (PE/EtOAc = 1 : 1) to product **a15** with 54% yield, rotational isomer ratio was 5 : 2. ^{1}H NMR (400 MHz, Chloroform-*d*) δ 7.06 (d, *J* = 27.4 Hz, 1H), 6.84 – 6.72(6.40 – 6.25) (m, 1H), 6.03 – 5.72 (m, 4H), 4.12 – 3.95 (m, 4H), 3.87 (s, 3H), 3.80 – 3.62 (m, 6H), 3.17 – 2.91 (m, 1H), 2.82 – 2.59 (m, 2H), 2.43

– 1.99 (m, 2H), 1.77 – 1.38 (m, 11H). MS (ESI-TOF) m/z calcd. for $C_{31}H_{37}NO_9Se$ $[M+H]^+$ 648.2, found 648.2.

Synthesis of compound a16.

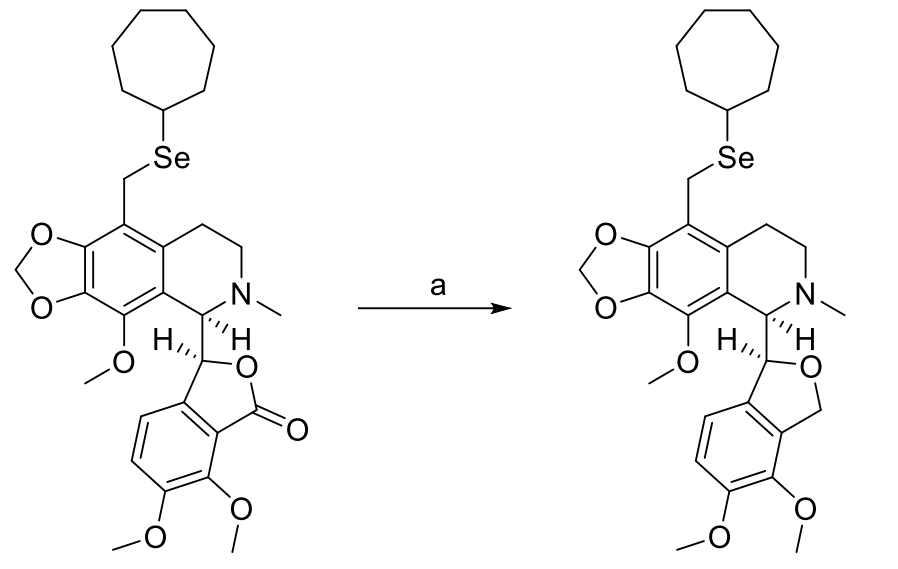

Reagents and conditions: (a) $NaBH_4$, $BF_3\cdot Et_2O$, THF, 0 ℃ to reflux, 2 h, 74%.

(R)-9-((cycloheptylselanyl)methyl)-5-((S)-4,5-dimethoxy-1,3-dihydroisobenzofuran-1-yl)-4-methoxy-6-methyl-5,6,7,8-tetrahydro-[1,3]dioxolo[4,5-g]isoquinoline (a16). A solution of **ES428** (0.30 g, 0.50 mmol) in $BF_3\cdot Et_2O$ (2.5 mL) was added dropwise to a solution of $NaBH_4$ (40 mg, 1 mmol) in THF (4 mL) at -5 °C and stirred for 1 h. Then the reaction was refluxed for another 2 h. The reaction was placed on ice and quenched with cold 10% HCl and allowed to stir for a further 1 h. The reaction mixture was then brought to pH 8 with 2.5M NaOH, extracted with dichloromethane and dried over anhydrous Na_2SO_4. Evaporation of solvent afforded a crude product, which was purified via column chromatography on silica gel (PE/EtOAc = 1 : 2) to product **a16** with 74% yield. ^1H NMR (400 MHz, Chloroform-*d*) δ 6.75 (d, *J* = 8.2 Hz, 1H), 6.56 – 6.37 (m, 1H), 5.93 – 5.87 (m, 2H), 5.81 – 5.66 (m, 1H), 5.05 – 4.80 (m, 2H), 4.65 – 4.40 (m, 1H), 3.83 (s, 3H), 3.80 (s, 3H), 3.76 – 3.66 (m, 2H), 3.62 (s, 3H), 3.50 – 3.28 (m, 1H), 3.17 – 3.04 (m, 1H), 2.99 – 2.74 (m, 2H), 2.74 – 2.47 (m, 4H), 2.26 – 2.02 (m, 2H), 1.78 – 1.64 (m, 4H), 1.63 – 1.41 (m, 6H). ^{13}C NMR (151 MHz, $CDCl_3$) δ 151.2, 142.7, 139.5, 133.2, 132.5, 125.2, 122.3, 117.5, 112.32, 112.27, 112.1, 100.7, 71.7, 63.2, 59.9, 59.0, 56.3, 55.9, 41.6, 36.1, 36.0, 31.9, 29.8, 29.7, 29.3, 28.0, 26.9, 22.7, 16.7. HRMS (ESI-TOF) m/z calcd. for $C_{30}H_{40}NO_6Se$ $[M+H]^+$ 590.2015, found 590.2023.

Synthesis of compound a17.

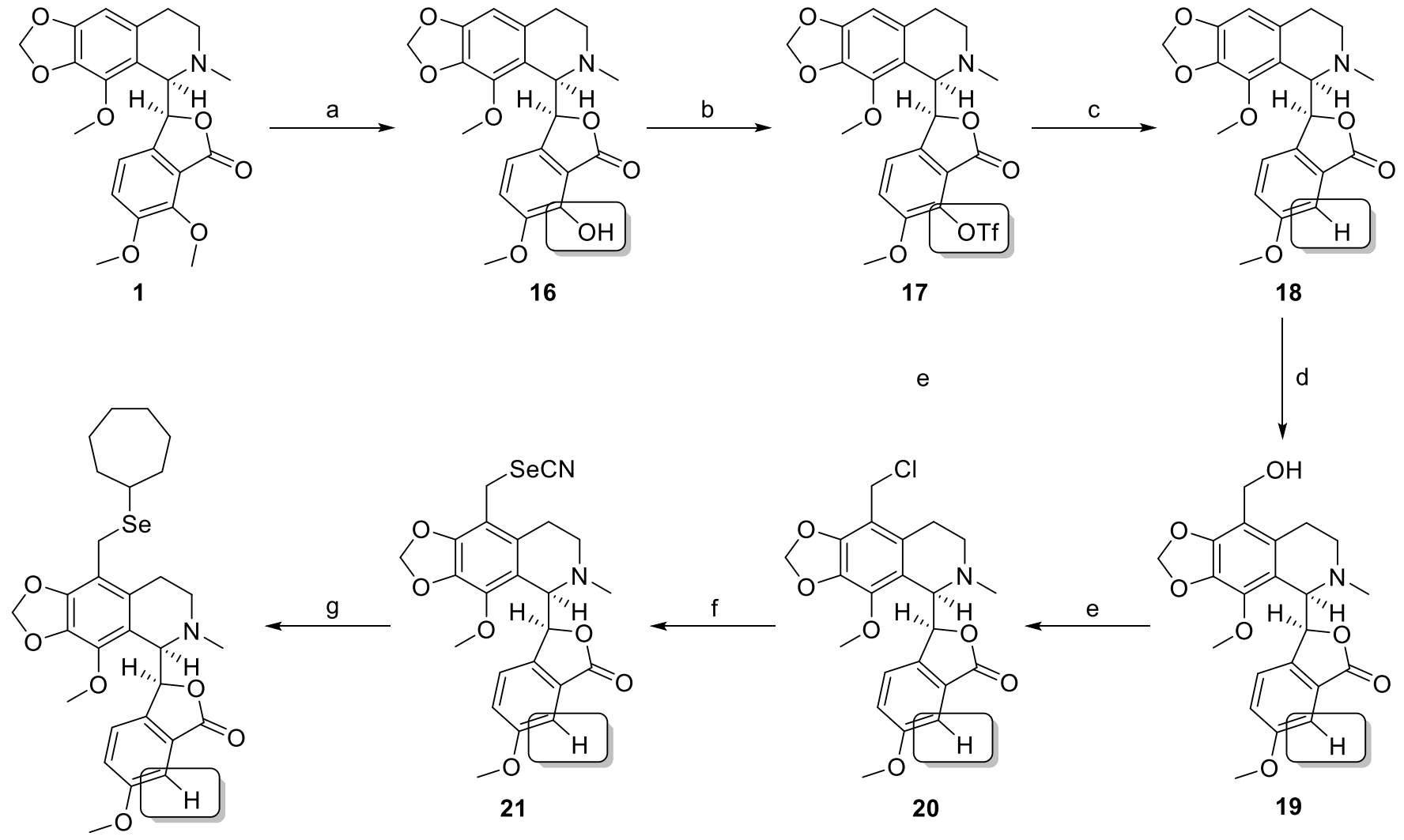

Reagents and conditions: (a) MeMgBr, BnOH, toluene, 120 ℃, 4 h, N_2; (b) Tf_2O, TEA, DMAP, DCM, rt, 2 h, 37%, 2 steps; (c) 1,3-Bis(diphenylphosphino)propane, $Pd(OAc)_2$, TEA, MeOH, DMSO, 80 ℃, 2 h, 51%; (d) paraformaldehyde, conc. HCl, 50 ℃, overnight; (e) $SOCl_2$, DCM,

0 ℃ to rt, 2 h; (f) KSeCN, MeCN, 80 ℃, 5 min; (g) 1-Bromocycloheptaner, $NaBH_4$, EtOH, rt, 2 h, N_2, 11%, 4 steps.

(S)-5-methoxy-1-((R)-4-methoxy-6-methyl-5,6,7,8-tetrahydro-[1,3]dioxolo[4,5-g]isoquinolin-5-yl)-3-oxo-1,3-dihydroisobenzofuran-4-yl trifluoromethanesulfonate (17). Compound **16** was prepared as described previously with minor modifications [15]. To a solution of benzyl alcohol (1.4 mL, 13.5 mmol) in toluene (1 mL) was slowly added MeMgBr (2.42 mL of 1.4 M in THF-toluene, 3.4 mmol) under N_2 atmosphere. The mixture stirred for 5 min then placed into an oil bath at 120 °C. Noscapine **1** (0.99 g, 2.4 mmol) was added portion wise over a period of 5 min. The resulting mixture was heated to 120 °C and stirred for 4 h then cooled to room temperature (the reaction mixture turned a dark red color). Saturated aqueous citric acid was added until the dark red color disappeared and the reaction mixture was acidic to wet pH paper. H_2O was added and the aqueous layer extracted with 1:1 EtOAc-hexanes three times to remove excess benzyl alcohol. The aqueous layer was basified to pH~9 using saturated aqueous Na_2CO_3 and then extracted with EtOAc four times. The EtOAc layers were combined and washed with 10% aqueous sodium citrate two times, 10% aqueous Rochelle salt two times, then saturated aqueous NaCl, dried over Na_2SO_4 and concentrated to give a crude product **16** as a brown oil. The crude product **16** (0.40 g, 1 mmol) was dissolved with 10 mL DCM, and Tf_2O (0.56 g, 2 mmol) was added followed by TEA (0.20 g, 2 mmol) and DMAP (24.4 mg, 0.2 mmol). The solution was stirred at room temperature for 2 h and washed with 1 M HCl three times to remove excess DMAP. Then the mixture was washed with brine, dried over Na_2SO_4, concentrated and purified via column chromatography on silica gel (DCM/MeOH = 100 : 1) to produce **17**, yield 37%. ^{1}H NMR (400 MHz, Chloroform-*d*) δ 7.09 (d, *J* = 8.5 Hz, 1H), 6.35 (d, *J* = 8.4 Hz, 1H), 6.30 (s, 1H), 5.97 – 5.92 (m, 2H), 5.65 (d, *J* = 4.1 Hz, 1H), 4.42 (d, *J* = 4.1 Hz, 1H), 4.05 (s, 3H), 3.92 (s, 3H), 2.66 – 2.57 (m, 1H), 2.54 (s, 3H), 2.40 – 2.30 (m, 2H), 1.82 – 1.70 (m, 1H). Spectral properties matched reported values [16].

(S)-6-methoxy-3-((R)-4-methoxy-6-methyl-5,6,7,8-tetrahydro-[1,3]dioxolo[4,5-g]isoquinolin-5-yl)isobenzofuran-1(3H)-one (18). Compound **18** was prepared as described previously with minor modifications [16]. To a solution of compound **17** (53.1mg, 0.1mmol) in 2mL DMSO/MeOH (1:1) was added 1,3-Bis(diphenylphosphino)propane (41.2 mg, 0.1 mmol), $Pd(OAc)_2$ (22.7 mg, 0.1 mmol) and TEA (374 mg, 3.7 mmol). The reaction was stirred at 80 ℃ for 2 h followed by cooling to room temperature. The mixture was extracted with EtOAc, washed with brine, dried over Na_2SO_4, concentrated and purified via column chromatography on silica gel (DCM/MeOH = 100 : 1) to product **18** with 51% yield. ^{1}H NMR (400 MHz, Chloroform-*d*) δ 7.30 (d, *J* = 2.4 Hz, 1H), 6.97 (d, *J* = 8.4 Hz, 1H), 6.35 – 6.29 (m, 2H), 5.97 – 5.92 (m, 2H), 5.67 (d, *J* = 4.1 Hz, 1H), 4.44 (d, *J* = 4.1 Hz, 1H), 4.05 (s, 3H), 3.84 (s, 3H), 2.65 – 2.56 (m, 1H), 2.55 (s, 3H), 2.42 – 2.28 (m, 2H), 1.97 – 1.85 (m, 1H). Spectral properties matched reported values [16].

(S)-3-((R)-9-((cycloheptylselanyl)methyl)-4-methoxy-6-methyl-5,6,7,8-tetrahydro-[1,3]dioxolo[4,5-g]isoquinolin-5-yl)-6-methoxyisobenzofuran-1(3H)-one (a17). To a solution of **18** (76.7 mg, 0.2 mmol) in concentrated hydrochloric acid (5 mL) was slowly added paraformaldehyde (0.18 g, 6 mmol). The reaction mixture was stirred at 50 °C overnight. After the reaction was complete, ammonium hydroxide was added until pH 10 at 0 °C. The resulting solution was extracted with EtOAc, the organic layer was dried over anhydrous Na_2SO_4 and concentrated to give crude **19**. To a solution of **19** in DCM (2 mL) was slowly added thionyl chloride (27.0 μL) at 0 °C. After the reaction was stirred for 2 h, the solvent was removed under vacuum to produce the crude **20**. To a solution of **20** in MeCN (4 mL) was added KSeCN (42.9 mg, 0.3 mmol) and the mixture was stirred at 80 °C for 5 min. Then the reaction was cooled fast to room temperature, diluted with water and extracted with EtOAc. The organic layer was dried over anhydrous Na_2SO_4, concentrated to give crude product **21**. Under N_2 atmosphere, $NaBH_4$ (7.57 mg, 0.2 mmol) was added slowly into a solution of **21** in EtOH (2 mL). After the

mixture was stirred for 30 min, 1-bromocycloheptaner (70.8 mg, 0.4 mmol) was slowly added and the mixture was stirred at room temperature for 2 h. Then the reaction was quenched with H_2O, extracted with EtOAc, and the organic layer was dried over anhydrous Na_2SO_4, concentrated and purified via column chromatography on silica gel (PE/EtOAc = 2: 1) to give product **a17** with 11% yield. ^{1}H NMR (400 MHz, Chloroform-*d*) δ 7.29 (d, *J* = 2.4 Hz, 1H), 6.99 (d, *J* = 8.4 Hz, 1H), 6.30 (d, *J* = 8.4 Hz, 1H), 6.00 – 5.94 (m, 2H), 5.62 (d, *J* = 4.1 Hz, 1H), 4.45 (d, *J* = 4.1 Hz, 1H), 4.03 (s, 3H), 3.84 (s, 3H), 3.75 – 3.65 (m, 2H), 3.19 – 3.08 (m, 1H), 2.69 – 2.59 (m, 1H), 2.59 – 2.50 (m, 4H), 2.39 – 2.28 (m, 1H), 2.19 – 2.08 (m, 2H), 1.88 – 1.63 (m, 6H), 1.56 – 1.39 (m, 5H). ^{13}C NMR (150 MHz, $CDCl_3$) δ 170.9, 160.3, 146.5, 140.5, 139.2, 133.4, 130.8, 129.1, 124.2, 122.1, 117.8, 112.5, 106.7, 100.9, 83.1, 60.8, 59.5, 49.8, 46.4, 41.6, 36.1, 36.1, 29.7, 28.1, 26.9, 24.0, 16.6, 16.4. HRMS (ESI-TOF) m/z calcd. for $C_{29}H_{35}NO_6Se$ $[M+H]^+$ 574.1702, found 574.1711.

Synthesis of compounds a18.

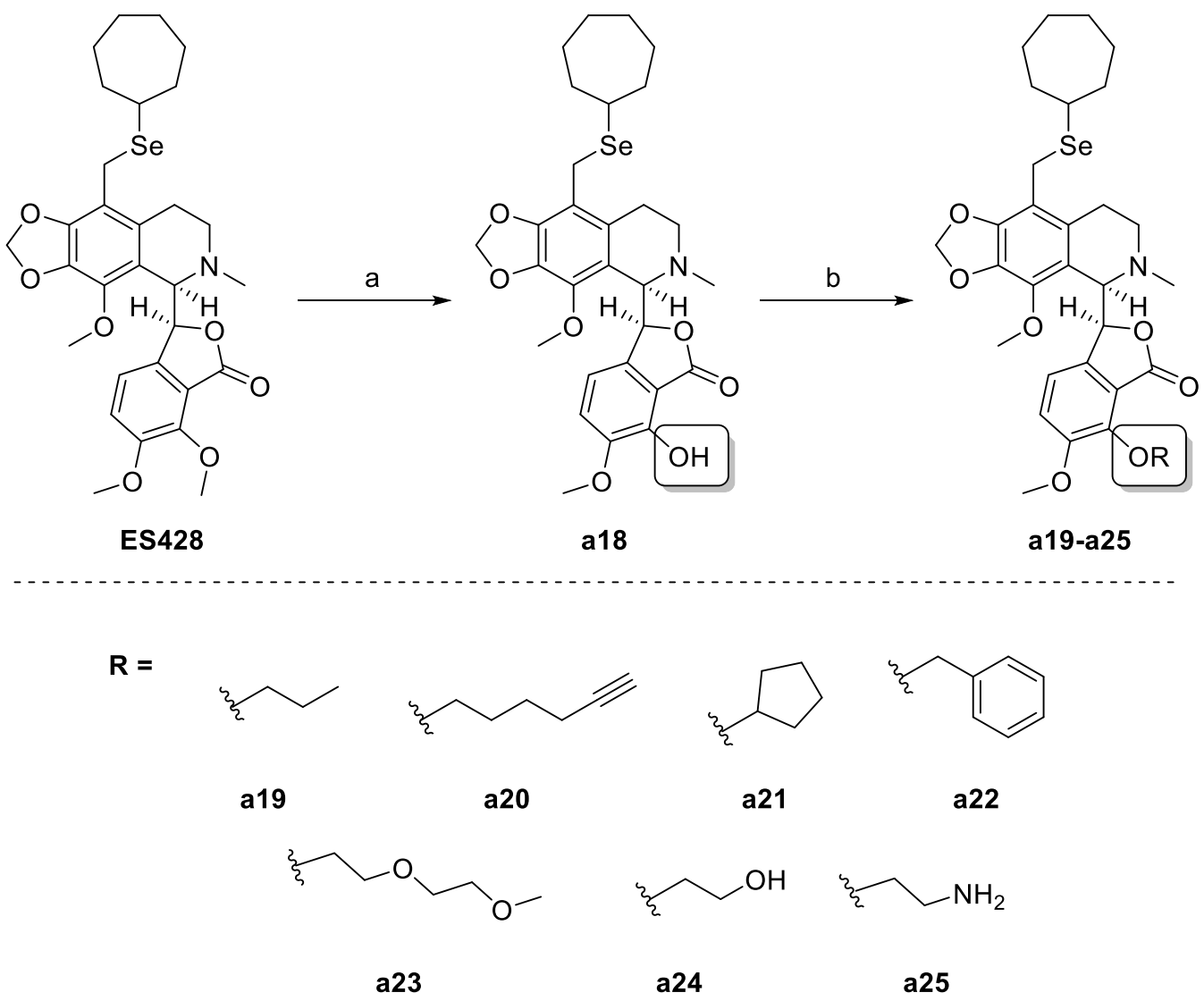

Reagents and conditions: (a) MeMgBr, BnOH, toluene, 120 ℃, 4 h, N_2; (b) RBr, K_2CO_3, acetone, reflux, 12 h, 7% - 29%, 2 steps.

(S)-3-((R)-9-((cycloheptylselanyl)methyl)-4-methoxy-6-methyl-5,6,7,8-tetrahydro-[1,3]dioxolo[4,5-g]isoquinolin-5-yl)-7-hydroxy-6-methoxyisobenzofuran-1(3H)-one (a18). To a solution of benzyl alcohol (1.45 g, 13.4 mmol) in toluene (1 mL) was slowly added MeMgBr (2.4 mL of 1.5 M in THF-toluene, 3.6 mmol) under N_2 atmosphere. The mixture stirred for 5 min then placed into an oil bath at 120 °C. **ES428** (1.00 g, 2.4 mmol) was added portion wise over a period of 5 min. The resulting mixture was heated to 120 °C and stirred for 4 h then cooled to room temperature (the reaction mixture turned a dark red color). Saturated aqueous citric acid was added until the dark red color disappeared and the reaction mixture was acidic to wet pH paper. H_2O was added and the aqueous layer extracted with 1:1 EtOAc-hexanes three times to remove excess benzyl alcohol. The aqueous layer was basified to pH~9 using saturated aqueous Na_2CO_3 and then extracted with EtOAc four times. The EtOAc layers were combined and washed with 10% aqueous sodium citrate two times, 10% aqueous Rochelle salt two times, then saturated aqueous NaCl, dried over Na_2SO_4, concentrated and purified via column chromatography on silica gel (DCM/MeOH = 50: 1) to give product **a18** with 9% yield. ^{1}H NMR (400 MHz, Chloroform-*d*) δ 6.79 (d, *J* = 8.1 Hz, 1H), 6.48 (d, *J* = 8.1 Hz, 1H), 6.00 – 5.95 (m, 2H), 4.52 (d, *J* = 15.7 Hz, 1H), 4.14 (d, *J* = 15.8 Hz, 1H), 4.05 (s, 3H), 3.89 (s, 3H), 3.79 – 3.71 (m, 1H), 3.67 (s, 2H), 3.14 – 3.05 (m, 2H), 3.02 (s, 3H), 2.14 – 2.04 (m, 2H), 1.75 – 1.61 (m, 5H), 1.60 – 1.50 (m, 4H), 1.49 – 1.39 (m, 3H). ^{13}C NMR (151 MHz, $CDCl_3$) δ 173.1, 156.4, 151.8, 148.0, 138.6, 133.9, 129.8, 122.1, 120.0, 119.1, 112.6, 111.4, 110.8, 101.4, 59.7,

57.6, 57.0, 55.8, 46.6, 42.1, 35.9, 31.9, 29.7, 28.0, 26.8, 22.7, 21.1, 16.1, 14.1. HRMS (ESI-TOF) m/z calcd. for $C_{29}H_{36}NO_7Se$ [M+H]$^+$ 590.1652, found 590.1661.

General synthetic procedure for compounds a19-a24. To a solution of **a18** (1.41 g, 2.4 mmol) in 24 mL acetone was added bromide (4.8 mmol) and K_2CO_3 (663 mg, 4.8 mmol). The solution was refluxed for 12 h. After that, it was cooled to room temperature, quenched with water, extracted with EtOAc, washed with brine, dried over Na_2SO_4, concentrated and purified via column chromatography on silica gel to yield **a19-a24**.

(S)-3-((R)-9-((cycloheptylselanyl)methyl)-4-methoxy-6-methyl-5,6,7,8-tetrahydro-[1,3]dioxolo[4,5-g]isoquinolin-5-yl)-6-methoxy-7-propoxyisobenzofuran-1(3H)-one (a19). Purified via column chromatography PE/EtOAc = 3: 1, 27% yield. ^{1}H NMR (400 MHz, Chloroform-*d*) δ 6.96 (d, *J* = 8.2 Hz, 1H), 6.01 (d, *J* = 8.3 Hz, 1H), 5.98 – 5.93 (m, 2H), 5.50 (d, *J* = 4.2 Hz, 1H), 4.40 (d, *J* = 4.2 Hz, 1H), 4.27 – 4.13 (m, 2H), 4.01 (s, 3H), 3.84 (s, 3H), 3.76 – 3.64 (m, 2H), 3.18 – 3.09 (m, 1H), 2.66 – 2.57 (m, 1H), 2.56 – 2.46 (m, 4H), 2.38 – 2.29 (m, 1H), 2.20 – 2.09 (m, 2H), 1.91 – 1.78 (m, 2H), 1.78 – 1.64 (m, 4H), 1.57 – 1.44 (m, 6H), 1.02 (t, *J* = 7.4 Hz, 3H), 0.90 – 0.80 (m, 1H). MS (ESI-TOF) m/z calcd. for $C_{32}H_{41}NO_7Se$ [M+H]$^+$ 632.2, found 632.2.

(S)-3-((R)-9-((cycloheptylselanyl)methyl)-4-methoxy-6-methyl-5,6,7,8-tetrahydro-[1,3]dioxolo[4,5-g]isoquinolin-5-yl)-7-(hex-5-yn-1-yloxy)-6-methoxyisobenzofuran-1(3H)-one (a20). Purified via column chromatography PE/EtOAc = 3: 1, 29% yield. ^{1}H NMR (400 MHz, Chloroform-*d*) δ 6.98 (d, *J* = 8.3 Hz, 1H), 6.10 (d, *J* = 8.3 Hz, 1H), 5.98 – 5.92 (m, 2H), 5.57 (d, *J* = 4.0 Hz, 1H), 4.45 (d, *J* = 3.9 Hz, 1H), 4.33 – 4.18 (m, 2H), 3.97 (s, 3H), 3.85 (s, 3H), 3.78 – 3.65 (m, 2H), 3.20 – 3.02 (m, 1H), 2.73 – 2.56 (m, 2H), 2.54 (s, 3H), 2.42 – 2.38 (m, 1H), 2.32 – 2.24 (m, 2H), 2.17 – 2.07 (m, 2H), 1.98 – 1.88 (m, 3H), 1.84 – 1.67 (m, 6H), 1.57 – 1.40 (m, 7H). MS (ESI-TOF) m/z calcd. for $C_{35}H_{43}NO_7Se$ [M+H]$^+$ 670.2, found 670.2.

(S)-3-((R)-9-((cycloheptylselanyl)methyl)-4-methoxy-6-methyl-5,6,7,8-tetrahydro-[1,3]dioxolo[4,5-g]isoquinolin-5-yl)-7-(cyclopentyloxy)-6-methoxyisobenzofuran-1(3H)-one (a21). Purified via column chromatography PE/EtOAc = 3: 1, 18% yield. ^{1}H NMR (400 MHz, Chloroform-*d*) δ 6.95 (d, *J* = 8.2 Hz, 1H), 6.02 (d, *J* = 8.2 Hz, 1H), 5.98 – 5.92 (m, 2H), 5.51 (d, *J* = 4.0 Hz, 1H), 5.18 – 5.11 (m, 1H), 4.41 (d, *J* = 4.0 Hz, 1H), 3.98 (s, 3H), 3.82 (s, 3H), 3.75 – 3.62 (m, 2H), 3.19 – 3.06 (m, 1H), 2.62 – 2.54 (m, 2H), 2.52 (s, 3H), 2.39 – 2.30 (m, 1H), 2.15 – 2.07 (m, 2H), 1.96 – 1.90 (m, 4H), 1.76 – 1.65 (m, 7H), 1.59 – 1.36 (m, 8H). MS (ESI-TOF) m/z calcd. for $C_{34}H_{43}NO_7Se$ [M+H]$^+$ 658.2, found 658.2.

(S)-7-(benzyloxy)-3-((R)-9-((cycloheptylselanyl)methyl)-4-methoxy-6-methyl-5,6,7,8-tetrahydro-[1,3]dioxolo[4,5-g]isoquinolin-5-yl)-6-methoxyisobenzofuran-1(3H)-one (a22). Purified via column chromatography PE/EtOAc = 3: 1, 24% yield. ^{1}H NMR (400 MHz, Chloroform-*d*) δ 7.59 – 7.51 (m, 2H), 7.35 – 7.27 (m, 3H), 6.94 (d, *J* = 8.2 Hz, 1H), 6.08 – 5.91 (m, 3H), 5.54 (d, *J* = 4.2 Hz, 1H), 5.33 (s, 2H), 4.43 (d, *J* = 4.0 Hz, 1H), 3.98 (s, 3H), 3.80 (s, 3H), 3.75 – 3.63 (m, 2H), 3.19 – 3.06 (m, 1H), 2.64 – 2.47 (m, 5H), 2.37 – 2.29 (m, 1H), 2.19 – 2.06 (m, 2H), 1.78 – 1.64 (m, 7H), 1.58 – 1.52 (m, 4H). MS (ESI-TOF) m/z calcd. for $C_{36}H_{41}NO_7Se$ [M+H]$^+$ 680.2, found 680.2.

(S)-3-((R)-9-((cycloheptylselanyl)methyl)-4-methoxy-6-methyl-5,6,7,8-tetrahydro-[1,3]dioxolo[4,5-g]isoquinolin-5-yl)-7-(2-hydroxyethoxy)-6-methoxyisobenzofuran-1(3H)-one (a23). Purified via column chromatography DCM/MeOH = 500: 1, 21% yield. ^{1}H NMR (400 MHz, Chloroform-*d*) δ 7.00 (d, *J* = 8.3 Hz, 1H), 6.06 (d, *J* = 8.2 Hz, 1H), 5.99 – 5.93 (m, 2H), 5.72 – 5.50 (m, 1H), 4.53 – 4.23 (m, 3H), 4.00 (s, 3H), 3.86 (s, 3H), 3.84 – 3.76 (m, 2H), 3.76 – 3.65 (m, 2H), 3.20 – 3.05 (m, 1H), 2.76 – 2.60 (m, 1H), 2.59 – 2.48 (m, 4H), 2.43 – 2.28 (m, 1H), 2.19 – 2.05 (m, 2H), 1.78 – 1.68 (m, 4H), 1.60 – 1.41 (m, 7H). MS (ESI-TOF) m/z calcd. for $C_{31}H_{39}NO_8Se$ [M+H]$^+$ 634.2, found 634.2.

(S)-7-(2-aminoethoxy)-3-((R)-9-((cycloheptylselanyl)methyl)-4-methoxy-6-methyl-5,6,7,8-tetrahydro-[1,3]dioxolo[4,5-g]isoquinolin-5-yl)-6-methoxyisobenzofuran-1(3H)-one (a24). Purified via column chromatography DCM/MeOH = 500: 1, 38% yield. ^{1}H NMR

(400 MHz, Chloroform-*d*) δ 6.98 (d, *J* = 8.3 Hz, 1H), 6.02 (d, *J* = 8.2 Hz, 1H), 5.99 – 5.93 (m, 2H), 5.55 (d, *J* = 4.2 Hz, 1H), 4.49 – 4.27 (m, 3H), 4.04 (s, 3H), 3.86 (s, 3H), 3.78 – 3.64 (m, 2H), 3.18 – 3.10 (m, 3H), 2.69 – 2.58 (m, 1H), 2.58 – 2.48 (m, 4H), 2.39 – 2.28 (m, 1H), 2.16 – 2.10 (m, 2H), 1.75 – 1.69 (m, 5H), 1.56 – 1.43 (m, 7H). ^{13}C NMR (150 MHz, $CDCl_3$) δ 169.0, 152.3, 146.5, 145.9, 140.8, 139.2, 133.4, 130.8, 120.4, 118.4, 118.3, 117.6, 112.3, 100.8, 100.8, 100.8, 82.2, 60.9, 59.5, 59.4, 56.6, 49.7, 46.3, 41.7, 41.6, 36.2, 36.2, 36.1, 36.1, 36.0, 28.1, 28.0, 26.9, 26.9, 26.9, 26.8, 23.8, 16.7. MS (ESI-TOF) m/z calcd. for $C_{31}H_{40}N_2O_7Se$ $[M+H]^+$ 633.2, found 633.2.

Synthesis of S-ES428

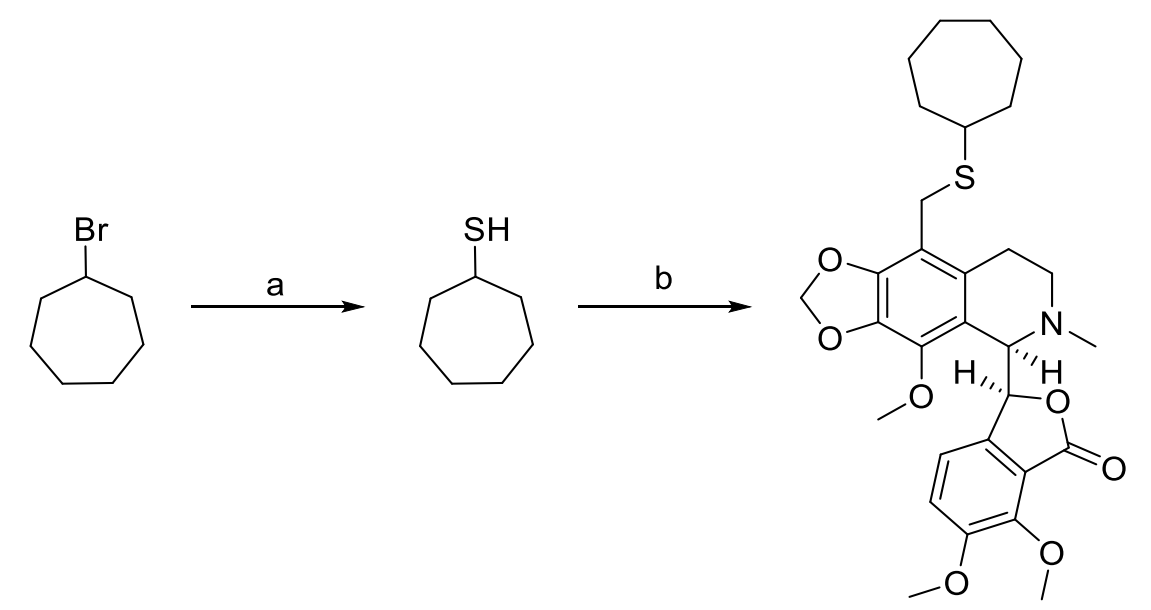

S-ES428

Reagents and conditions: (a): (i) potassium thioacetate, DMF, 120 ℃, 3 h; (ii) NaOH, EtOH, 60 ℃, 2 h; (b) **6**, DIPEA, DCM, rt, 2 h, 26%, 3 steps.

(S)-3-((R)-9-((cycloheptylthio)methyl)-4-methoxy-6-methyl-5,6,7,8-tetrahydro-[1,3]dioxolo[4,5-g]isoquinolin-5-yl)-6,7-dimethoxyisobenzofuran-1(3H)-one (S-ES428). Potassium thioacetate (91.7 mg, 0.75 mmol) was added to a solution of bromo-cycloheptan (88.5 mg, 0.5 mmol) in N,N-dimethylformamide (5 mL). The resulting mixture was warmed to 120 °C and stirred for 3 h. Then the solution was cooled down and diluted with water, extracted with EtOAc, and the combined organic layers was washed with brine, dried over anhydrous Na_2SO_3 and concentrated. The residue was dissolved with ethanol (5 mL), and 1M NaOH (1 mL) was added. The solution was stirred at 60 °C for 2 h. Then the solution was cooled down and diluted with water, extracted with EtOAc, and the combined organic layers was washed with brine, dried over anhydrous Na_2SO_3 and concentrated to give the crude cycloheptyl thiol. To a solution of the crude cycloheptyl thiol in dichloromethane (5 mL), **6** (101.2 mg, 0.2 mmol) and N,N-diisopropylethylamine (64.6 mg, 0.5 mmol) was added. Then, it was stirred for 2 h at room temperature and diluted with water. The resulting solution was extracted with dichloromethane, washed with brine, dried over Na_2SO_4. Filtered and concentrated to give the crude product. It was purified via column chromatography on silica gel (PE/EtOAc = 3: 1) to yield **S-ES428** (28.9 mg, 26%) as a light-yellow foam. ^{1}H NMR (400 MHz, Chloroform-*d*) δ 6.91 (d, *J* = 8.2 Hz, 1H), 6.00 (d, *J* = 8.5 Hz, 1H), 5.93 – 5.86 (m, 3H), 5.45 (d, *J* = 4.0 Hz, 1H), 4.34 (d, *J* = 4.4 Hz, 1H), 4.02 (s, 3H), 3.95 (s, 3H), 3.79 (s, 3H), 3.61 (s, 2H), 3.08 – 2.97 (m, 1H), 2.64 – 2.48 (m, 2H), 2.47 (s, 3H), 2.34 – 2.23 (m, 2H), 2.04 – 1.84 (m, 2H), 1.84 – 1.74 (m, 1H), 1.69 – 1.58 (m, 2H), 1.58 – 1.47 (m, 5H), 1.46 – 1.31 (m, 3H). ^{13}C NMR (151 MHz, $CDCl_3$) δ 168.2, 147.7, 147.3, 141.2, 139.7, 133.4, 131.2, 120.1, 118.4, 117.9, 117.8, 110.4, 100.9, 81.8, 62.3, 61.0, 59.4, 56.8, 53.4, 49.6, 46.2, 45.8, 44.4, 35.1, 34.9, 34.6, 28.3, 26.2, 26.1, 26.0, 25.8, 23.8, 1.0. HRMS (ESI-TOF) m/z calcd. for $C_{30}H_{37}NO_7S$ $[M+H]^+$ 556.2363, found 556.2368.

Synthesis of O-ES428

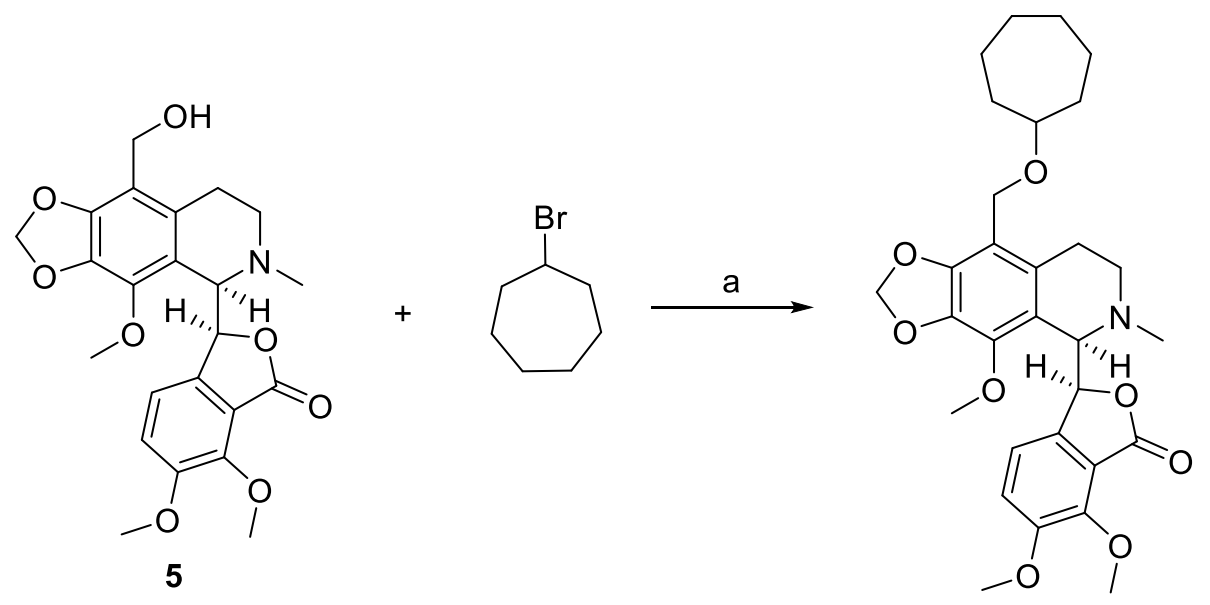

5 O-ES428

Reagents and conditions: (a) DIPEA, DCM, 50 ℃, 2 h, 74%.

(S)-3-((R)-9-((cycloheptyloxy)methyl)-4-methoxy-6-methyl-5,6,7,8-tetrahydro-[1,3]dioxolo[4,5-g]isoquinolin-5-yl)-6,7-dimethoxyisobenzofuran-1(3H)-one (O-ES428). To a solution of **5** (88.7 mg, 0.2 mmol) in dichloromethane (2 mL), bromo-cycloheptan (70.8 mg, 0.4 mmol) was added, following by the addition of N, N-diisopropylethylamine (77.5 mg, 0.6 mmol). The resulting solution was stirred at 50 °C for 2 h. After that, the solvent was removed under vacuum and the residue was purified by flash column chromatography on silica gel (PE/EtOAc = 2: 1) to give **O-ES428** (79.9 mg, 74%) as a light-yellow foam. ^{1}H NMR (600 MHz, Chloroform-*d*) δ 6.97 (d, *J* = 8.1 Hz, 1H), 6.19 (d, *J* = 8.1 Hz, 1H), 5.98 – 5.90 (m, 2H), 5.60 (s, 1H), 4.51 – 4.33 (m, 3H), 4.08 (s, 3H), 3.95 (s, 3H), 3.85 (s, 3H), 3.53 – 3.37 (m, 1H), 2.79 – 2.59 (m, 2H), 2.55 (s, 3H), 2.48 – 2.34 (m, 1H), 1.95 – 1.84 (m, 2H), 1.77 – 1.56 (m, 10H). ^{13}C NMR (150 MHz, $CDCl_3$) δ 152.2, 147.7, 140.1, 133.3, 118.3, 118.0, 111.3, 100.8, 79.4, 62.3, 61.1, 60.8, 59.3, 56.7, 45.8, 33.9, 29.7, 28.4, 27.2, 23.0, 14.1, 8.6. HRMS (ESI-TOF) m/z calcd. for $C_{30}H_{38}NO_8$ $[M+H]^+$ 540.2592, found 540.2599.

Synthesis of C-ES428

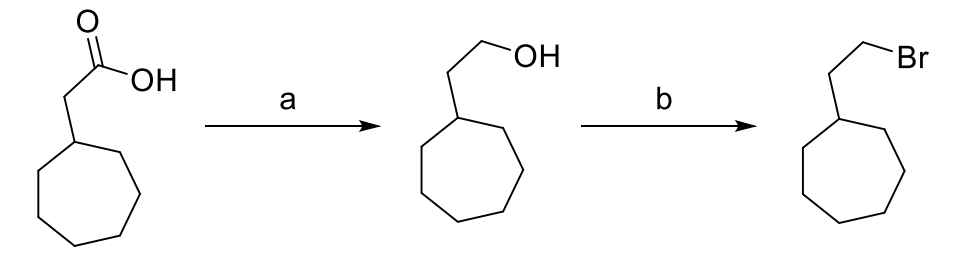

22

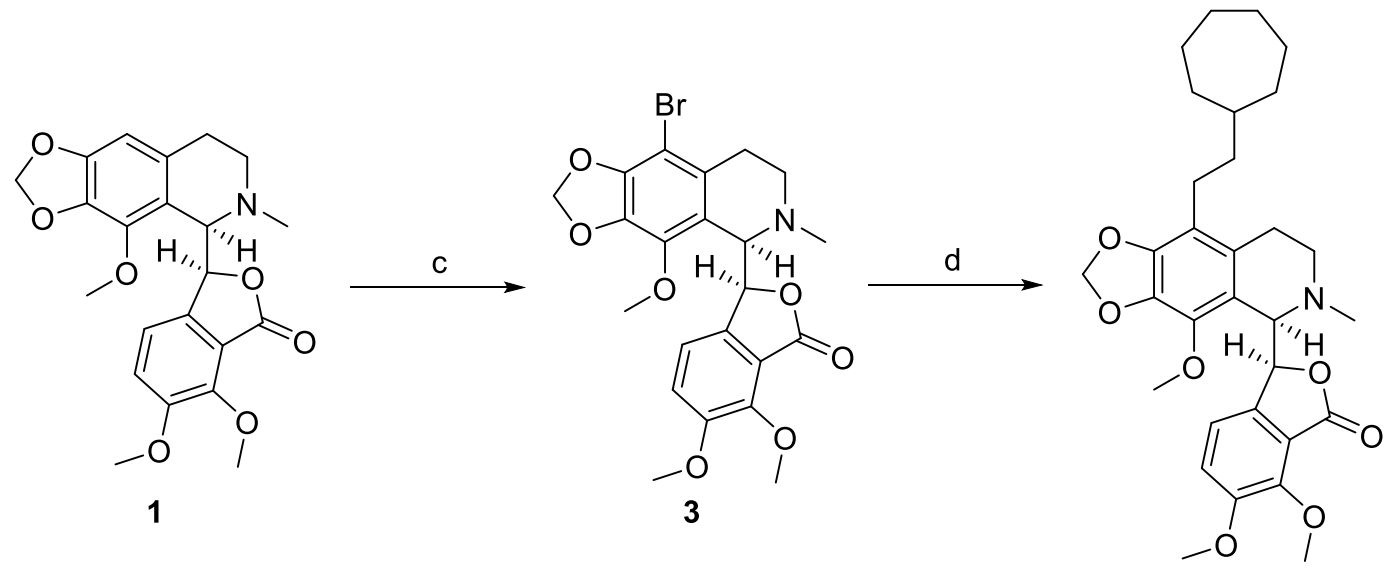

1 3 C-ES428

Reagents and conditions: (a) $LiAlH_4$, THF, rt, 4 h, 98%; (b) PBr_3, DCM, rt, 1 h; (c) HBr, Br_2, H_2O, rt, 2 h, 90%; (d) **22**, NiI_2, 4,4'-di-tert-butyl-2,2'-bipyridine, Zn dust, pyridine, $MgCl_2$, DMA, rt, 24 h, N_2, 31%.

(2-bromoethyl) cycloheptane (22). To a solution of 2-cycloheptylacetic acid (312 mg, 2.0 mmol) in 5 mL THF, $LiAlH_4$ (121 mg, 3.2 mmol) was added dropwise under 0 °C. The mixture was stirred at room temperature for 4 h followed by slowly addition of water at 0 °C until no gas production. 1M HCl was added slowly to the solution until no solid produced, Then the reaction was extracted with EtOAc, washed with saturated aqueous NaCl and the organic layer was dried over anhydrous Na_2SO_4, concentrated and purified via column chromatography (PE/EtOAc = 10:1) to produce cycloheptyl ethanol with 98% yield. ^{1}H NMR (400 MHz, Chloroform-*d*) δ 3.63 (t, *J* = 6.9 Hz, 2H), 2.52 (s, 1H), 1.75 – 1.35 (m, 13H), 1.28 – 1.10 (m, 2H). Cycloheptyl ethanol (279 mg, 1.96 mmol) was dissolved with 20 mL DCM and cooled to

0 °C followed by slowly addition of PBr_3 (637 mg, 2.4 mmol). Then the reaction mixture was warmed to room temperature and stirred for 1 h. After completed, the solution was quenched with ice water, extracted with EtOAc, washed with saturated aqueous NaCl. The organic layer was dried over anhydrous Na_2SO_4, and concentrated to yield the crude product **22**.

(S)-3-((R)-9-bromo-4-methoxy-6-methyl-5,6,7,8-tetrahydro-[1,3]dioxolo[4,5-g]isoquinolin-5-yl)-6,7-dimethoxyisobenzofuran-1(3H)-one (3). 9′-bromonoscapine **3** was prepared as described previously with minor modifications [17]. Briefly, to a solution of noscapine (4.0 g, 8.9 mmol, 1 equiv.) in 48% HBr (15 mL) was added freshly prepared 3% Br_2/H_2O (50 mL) slowly until no light-yellow precipitate formed. The reaction mixture was then stirred at room temperature for 1 h to attain completion, adjusted to pH 10 using ammonia solution to afford solid precipitate which was then recrystallized with ethanol to produce 9′-bromonoscapine **3** (3.9 g, 90%). ^{1}H NMR (400 MHz, $CDCl_3$) δ 7.02 (d, J = 8.1 Hz, 1H), 6.29 (d, J = 8.1 Hz, 1H), 6.02 (s, 2H), 5.49 (d, J = 4.3 Hz, 1H), 4.33 (d, J = 4.3 Hz, 1H), 4.09 (s, 3H), 3.98 (s, 3H), 3.87 (s, 3H), 2.78 – 2.55 (m, 2H), 2.51 (s, 3H), 2.49 – 2.43 (m, 1H), 1.99 – 1.92 (m, 1H). Spectral properties matched reported values [17].

(S)-3-((R)-9-(2-cycloheptylethyl)-4-methoxy-6-methyl-5,6,7,8-tetrahydro-[1,3]dioxolo[4,5-g]isoquinolin-5-yl)-6,7-dimethoxyisobenzofuran-1(3H)-one (C-ES428). To an 8 mL vial equipped with a stir bar was added 4,4'-di-tert-butyl-2,2'-bipyridine (12.0 mg, 0.045 mmol), 9′-bromonoscapine **3** (0.22 g, 0.45 mmol), crude product **22**, nickel iodide (14.1 mg, 0.045 mmol), Zn dust (45.7 mg, 0.7 mmol), $MgCl_2$ (42.9 mg, 0.45 mmol). The vial was sealed and placed under nitrogen before 4.5 mL of DMF was added. The solution was degassed by sparging with nitrogen while stirring for 10 minutes before addition of pyridine (35.7 mg, 0.45 mmol). Then it was stirred for 24 h at room temperature and quenched with water, extracted with EtOAc, washed with saturated aqueous NaCl. The organic layer was dried over anhydrous Na_2SO_4, and concentrated. Purification by column chromatography (PE/EtOAc = 4:1) produced **C-ES428** in 31% yield. ^{1}H NMR (400 MHz, Chloroform-*d*) δ 6.88 (d, J = 8.2 Hz, 1H), 5.97 – 5.89 (m, 2H), 5.54 (d, J = 4.0 Hz, 1H), 4.45 (d, J = 4.1 Hz, 1H), 4.09 (s, 3H), 4.01 (s, 3H), 3.85 (s, 3H), 2.70 – 2.60 (m, 1H), 2.56 (s, 3H), 2.48 – 2.37 (m, 2H), 2.36 – 2.24 (m, 1H), 1.82 – 1.71 (m, 2H), 1.69 – 1.40 (m, 12H), 1.38 – 1.15 (m, 3H). ^{13}C NMR (151 MHz, $CDCl_3$) δ 168.2, 152.2, 147.6, 146.5, 141.1, 138.4, 133.5, 130.6, 120.4, 117.8, 117.8, 117.3, 115.9, 100.6, 82.1, 62.3, 61.1, 59.5, 56.7, 50.4, 46.6, 39.3, 37.9, 34.8, 34.4, 29.7, 29.4, 28.5, 26.6, 26.5, 24.5, 23.5, 14.1. HRMS (ESI-TOF) m/z calcd. for $C_{31}H_{39}NO_7$ $[M+H]^+$ 537.2727, found 538.2806.

Synthesis of biotin-labeled ES428

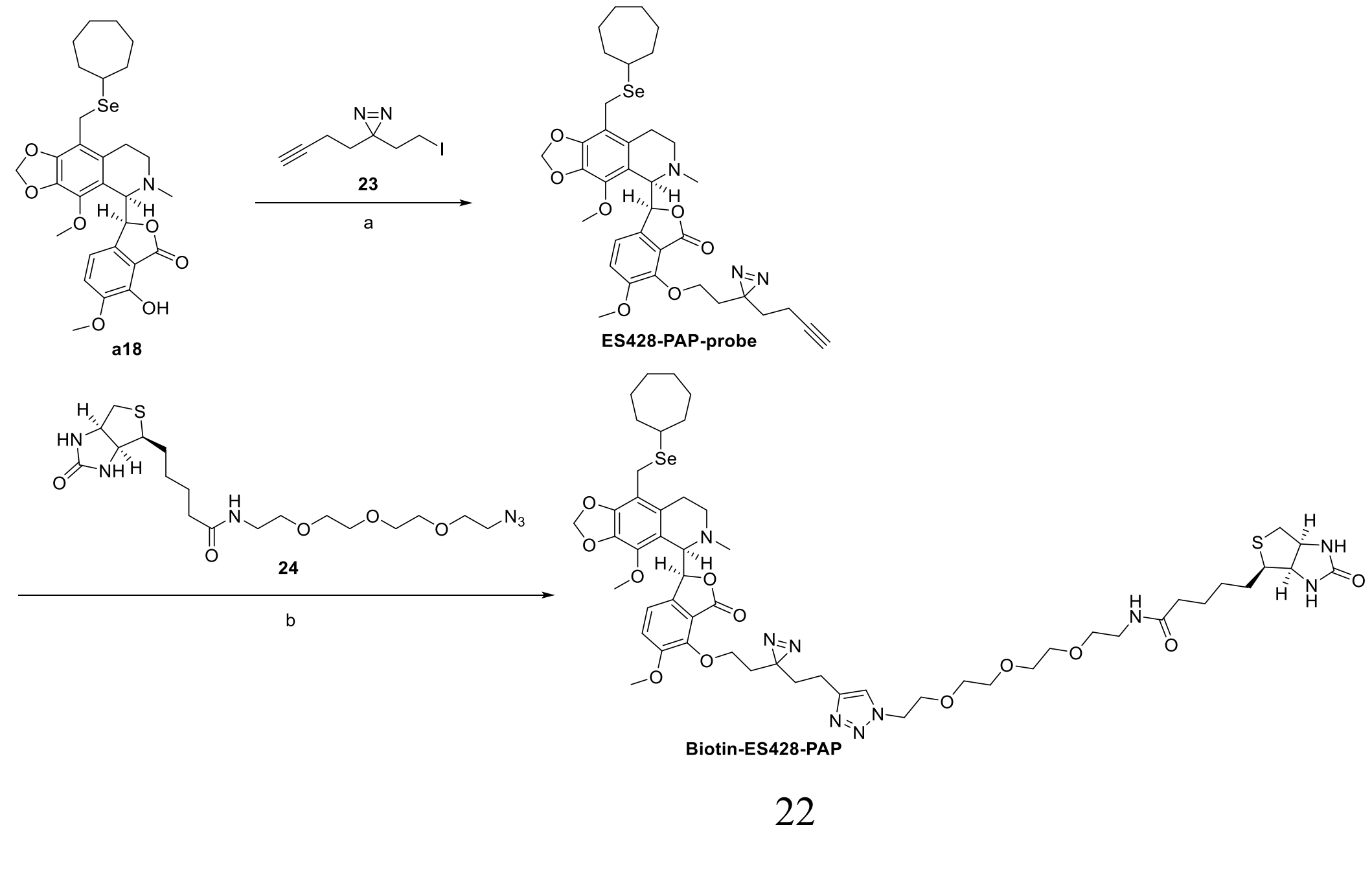

Reagents and conditions: (a) K_2CO_3, acetone, reflux, 12 h, dark, 35%; (b) $CuSO_4$, sodium ascorbate, MeOH, THF rt, 12 h, N_2, dark, 88%.

(S)-7-(2-(3-(but-3-yn-1-yl)-3H-diazirin-3-yl)ethoxy)-3-((R)-9-((cycloheptylselanyl)methyl)-4-methoxy-6-methyl-5,6,7,8-tetrahydro-[1,3]dioxolo[4,5-g]isoquinolin-5-yl)-6-methoxyisobenzofuran-1(3H)-one (ES428-PAP-probe). To a solution of compound **a18** (294 mg, 0.5 mmol) and **23** (248 mg, 1 mmol) in 5 mL acetone was added K_2CO_3 (138 mg, 1 mmol). Then it was refluxed for 12 h in darkness, the reaction was cooled to room temperature, quenched by water, extracted with EtOAc, washed with brine and dried over anhydrous Na_2SO_4. Evaporation of solvent afforded a crude product, which was purified via column chromatography (PE/EtOAc = 3: 1) to produce **ES428-PAP-probe** in 35% yield. ^{1}H NMR (400 MHz, Chloroform-*d*) δ 6.97 (d, *J* = 8.3 Hz, 1H), 6.10 – 5.99 (m, 1H), 6.00 – 5.94 (m, 2H), 5.56 – 5.51 (m, 1H), 4.49 – 4.36 (m, 1H), 4.20 – 4.07 (m, 2H), 4.00 (s, 3H), 3.85 (s, 3H), 3.76 – 3.64 (m, 2H), 3.19 – 3.09 (m, 1H), 2.69 – 2.60 (m, 1H), 2.53 (s, 3H), 2.42 – 2.27 (m, 1H), 2.20 – 2.03 (m, 4H), 1.96 (t, *J* = 2.6 Hz, 1H), 1.90 (t, *J* = 6.7 Hz, 2H), 1.80 (t, *J* = 7.6 Hz, 2H), 1.76 – 1.40 (m, 13H). ^{13}C NMR (150 MHz, $CDCl_3$) δ 168.1, 152.3, 146.5, 146.0, 140.9, 139.2, 133.3, 128.6, 127.7, 127.0, 118.4, 118.2, 112.3, 100.8, 83.0, 81.8, 69.4, 69.0, 65.4, 60.9, 59.4, 56.7, 53.4, 49.6, 46.3, 41.6, 36.1, 36.0, 33.9, 32.3, 28.0, 28.0, 26.9, 26.7, 16.7, 13.3. HRMS (ESI-TOF) m/z calcd. for $C_{36}H_{43}N_3O_7Se$ $[M+H]^+$ 710.2339, found 710.2347.

N-(2-(2-(2-(2-(4-(2-(3-(2-(((S)-1-((R)-9-((cycloheptylselanyl)methyl)-4-methoxy-6-methyl-5,6,7,8-tetrahydro-[1,3]dioxolo[4,5-g]isoquinolin-5-yl)-5-methoxy-3-oxo-1,3-dihydroisobenzofuran-4-yl)oxy)ethyl)-3H-diazirin-3-yl)ethyl)-1H-1,2,3-triazol-1-yl)ethoxy)ethoxy)ethoxy)ethyl)-5-((3aR,4R,6aS)-2-oxohexahydro-1H-thieno[3,4-d]imidazol-4-yl)pentanamide (Biotin-ES428-PAP). To a solution of **ES428-PAP-probe** (128 mg, 0.18 mmol) and **24** (80 mg, 0.18 mmol) in 0.4 mL MeOH and 1.8 mL THF was slowly added $CuSO_4$ (28.7 mg, 0.18 mmol) and sodium ascorbate (35.7 mg, 0.18 mmol) under N_2 atmosphere. The mixture was stirred at room temperature for 12 h in the dark. The solution was evaporated and purified via column chromatography (DCM/MeOH = 20 : 1) to produce **Biotin-ES428-PAP** in 88% yield. ^{1}H NMR (400 MHz, Chloroform-*d*) δ 7.58 (s, 1H), 6.96 (d, *J* = 8.3 Hz, 1H), 6.74 – 6.68 (m, 1H), 6.09 – 5.94 (m, 4H), 5.52 (d, *J* = 4.2 Hz, 1H), 5.22 – 5.15 (m, 1H), 4.54 – 4.47 (m, 3H), 4.40 (d, *J* = 4.2 Hz, 1H), 4.36 – 4.29 (m, 1H), 4.18 – 4.04 (m, 2H), 4.02 (s, 3H), 3.94 – 3.85 (m, 3H), 3.84 (s, 3H), 3.76 – 3.62 (m, 7H), 3.62 – 3.52 (m, 9H), 3.51 – 3.37 (m, 4H), 3.19 – 3.10 (m, 2H), 2.95 – 2.86 (m, 2H), 2.77 – 2.69 (m, 1H), 2.62 – 2.54 (m, 3H), 2.52 (s, 3H), 2.26 – 2.07 (m, 5H), 2.01 – 1.93 (m, 3H), 1.92 – 1.85 (m, 4H), 1.76 – 1.62 (m, 9H), 1.60 – 1.39 (m, 8H). ^{13}C NMR (150 MHz, $CDCl_3$) δ 173.3, 168.3, 163.6, 163.6, 152.4, 146.5, 146.5, 146.0, 140.9, 139.2, 133.4, 130.8, 122.4, 120.4, 118.5, 118.3, 112.3, 100.8, 81.9, 70.7, 70.6, 70.5, 70.4, 70.4, 70.1, 70.1, 70.0, 70.0, 69.9, 69.6, 69.4, 61.8, 60.9, 60.1, 59.5, 56.7, 55.4, 50.7, 50.1, 49.6, 46.3, 41.7, 40.5, 39.1, 36.1, 36.1, 35.9, 35.8, 34.0, 32.6, 29.7, 29.3, 28.1, 28.1, 26.9, 26.9, 25.5, 23.7, 20.2, 16.7. HRMS (ESI-TOF) m/z calcd. for $C_{54}H_{75}N_9O_{12}SSe$ $[M+H]^+$ 1154.4494, found 1154.4520.

2. Table S1: Information for AML patients.

Patient ID	Gender	Age	Diagnosis	Karyotype	Mutations	BM Blast (%)	Response group
1	F	61	AML-M4	Normal	$NPM1^{mut}$	51.5%	Sensitive to differentiation
2	F	77	AML-M5	Normal	$FLT3\text{-}ITD^{+}$	86.0%	
3	F	18	AML-M5	Normal	Negative	81.5%	
4	M	26	AML-M5	Normal	$FLT3\text{-}ITD^{+}$	93.5%	
5	M	34	AML-unclassified	Normal	Negative	72.0%	
6	F	58	AML-M5	Normal	Negative	72.6%	Resistance to differentiation
7	M	44	AML-M3	t (15;17)	Negative	98%	
8	F	27	AML-unclassified	+1	$ETV6^{mut}$; $KDM6A^{mut}$	90%	
9	M	63	AML-M2b	t (8;21)	Negative	82%	

3. Figure S1-S20

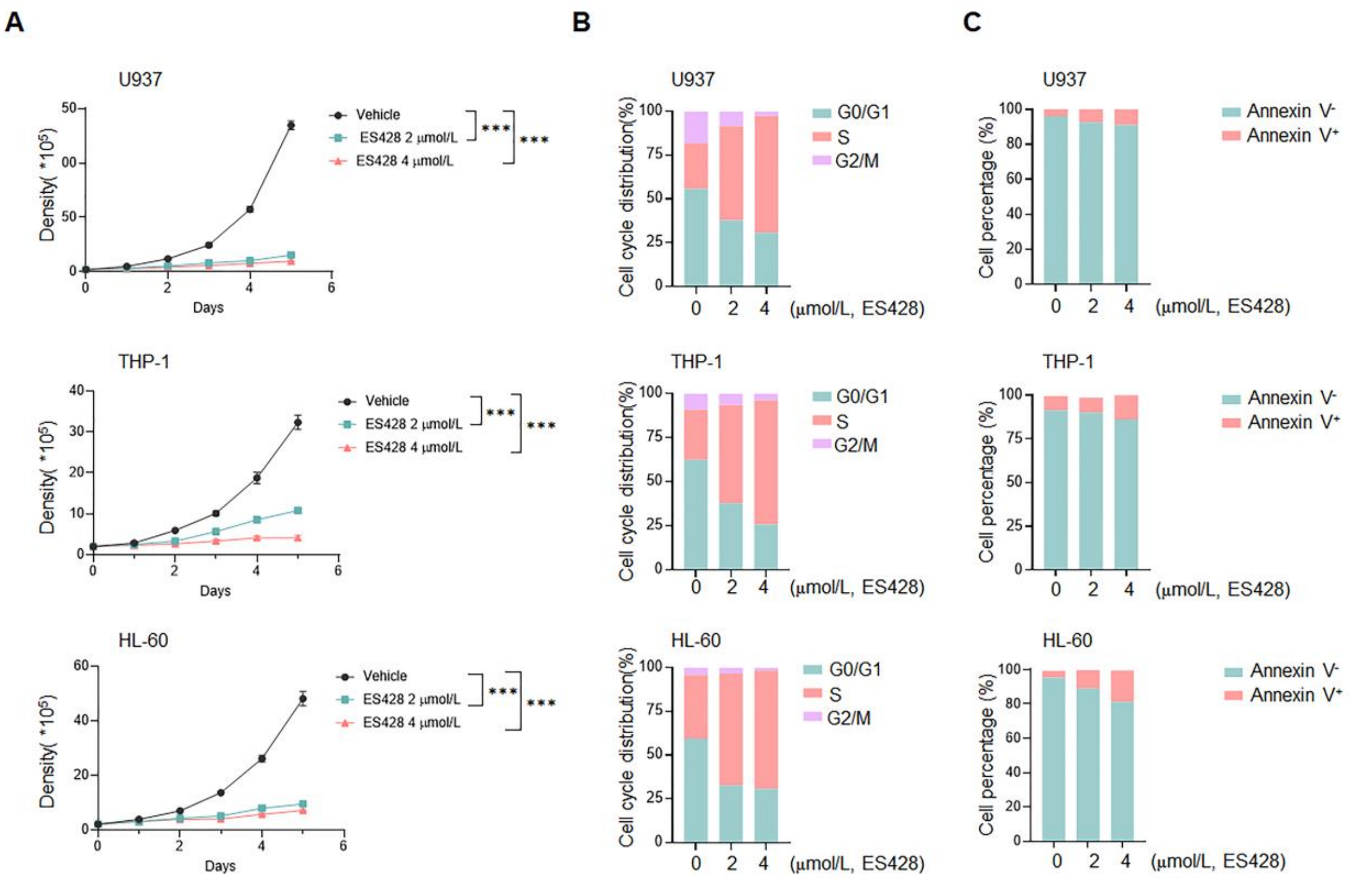

Figure S1 ES428 induces cell cycle arrest at the S phase. (A) The multiple AML cell lines were treated with ES428 at concentrations as indicated, and growth inhibition was evaluated by trypan-blue exclusion assay. (B–C) The multiple AML cell lines were treated with ES428 at concentrations as indicated for 3 days. The percentages of cells at the indicated phases of cell cycle were detected by flow cytometry (B). The apoptosis-inducing activity was evaluated by Annexin-V/PI staining. The percentage of viable cells (Annexin-V$^-$/PI$^-$) and apoptotic cells including the early-stage apoptotic cell population (Annexin-V$^+$ PI$^-$) and late-stage apoptotic cell population (Annexin-V$^+$ PI$^+$) was quantified (C). Results are representative of at least three independent experiments. Error bars in A represent the mean ± SD of triplicate samples in an independent experiment. *P<0.05, **P<0.01 and ***P<0.001 against the vehicle group.

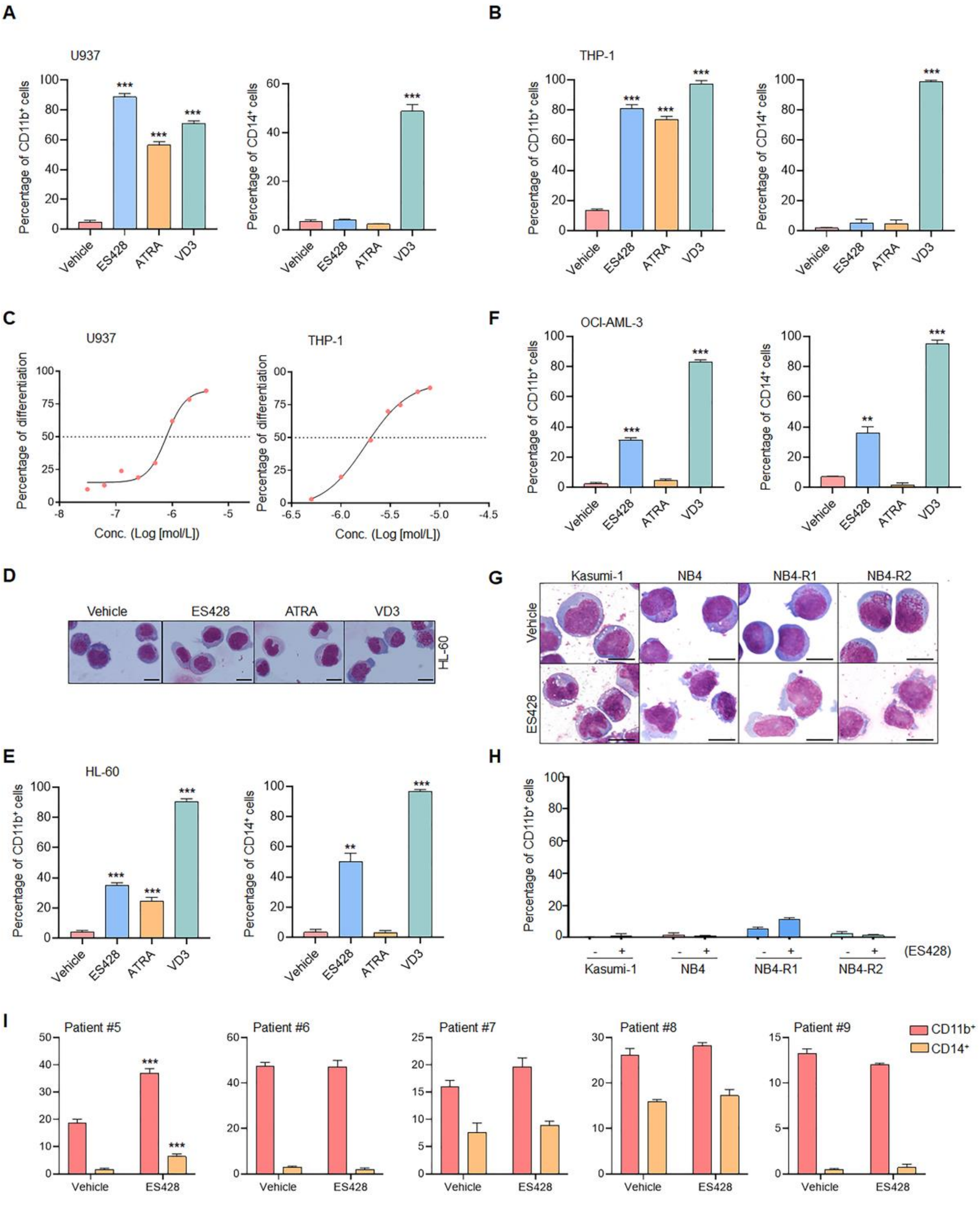

Figure S2 Evaluation of ES428's differentiation-inducing efficacy. (A–B) The percentages of $CD11b^+$ and $CD14^+$ cells were quantified in U937 cells (A) and THP-1 cells (B) following compound treatment as indicated for 3 days. (C) The ED_{50} of cellular differentiation-inducing potency was determined in both U937 and THP-1 cell lines. (D–F) HL-60 cells and OCI-AML3 cells were treated with 2 μmol/L ES428, 1 μmol/L ATRA, or 0.1 μmol/L VD3 for 3 days. Wright's staining morphology of HL-60 cells was observed (D). The quantitative results from flow cytometry analysis of the percentages of $CD11b^+$ and $CD14^+$ cells as indicated in HL-60

(E) and OCI-AML3 (F). (G–H) The AML cells indicated were treated with 2 μmol/L ES428 for 3 days. Wright's staining morphology was observed (G). The quantitative results from flow cytometry analysis of cell surface expression of the percentages of $CD11b^+$ cells as indicated (H). (I) The percentage of anti-human $CD11b^+$ and $CD14^+$ cells in primary AML patient samples were measured by flow cytometry following treatment with ES428 for 3 days. Error bars represent the mean ± SD of triplicate samples in an independent experiment. **P*<0.05, ***P*<0.01 and ****P*<0.001 against the vehicle group. Results are representative of at least three independent experiments.

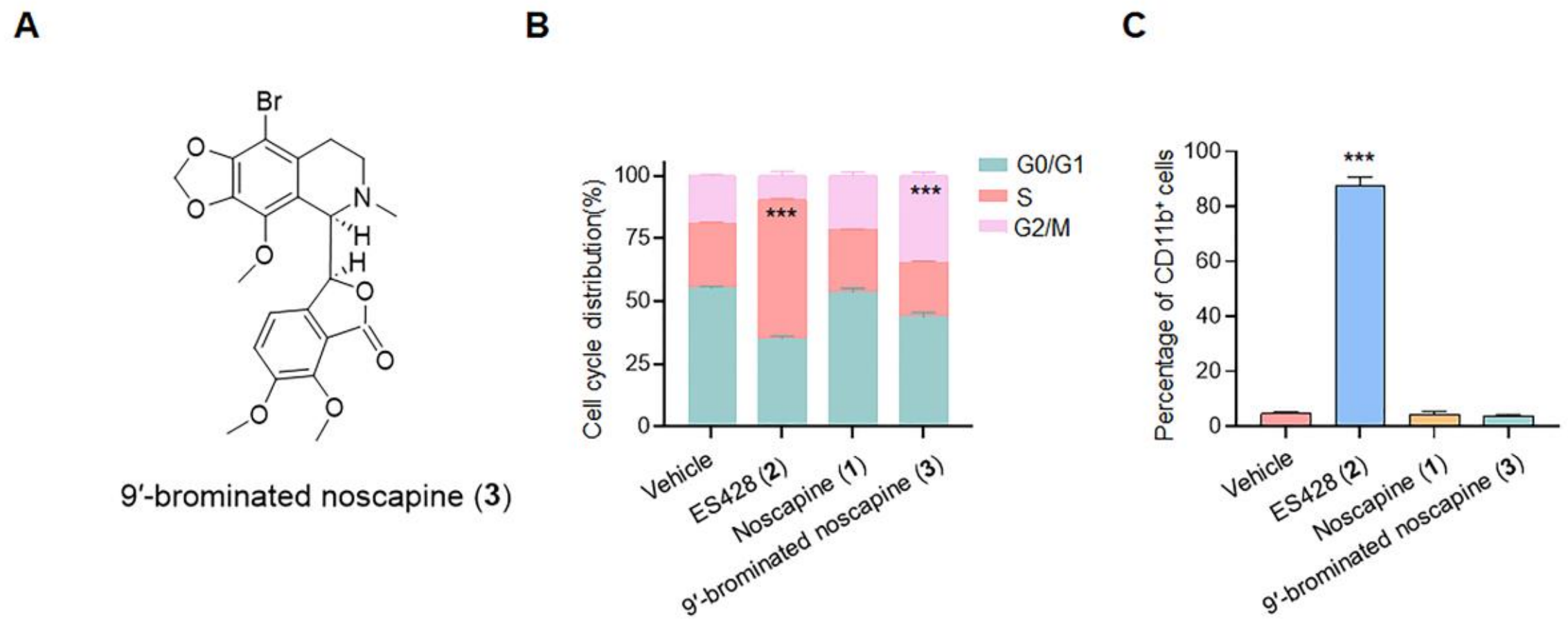

Figure S3 ES428's action in AML does not rely on microtubule inhibition. (A) Chemical structure of 9′-brominated analog (**3**) is shown. (B–C) AML cells were treated with ES428, noscapine and 9′-brominated analog at an approximate IC_{50} concentration. The percentages of cells at the indicated phases of cell cycle (B) and the percentages of $CD11b^{+}$ cells (C) were detected by flow cytometry. Error bars represent the mean ± SD of triplicate samples in an independent experiment. **P*<0.05, ***P*<0.01 and ****P*<0.001 against the vehicle group. Error bars in (B–C) are representative of at least three independent experiments.

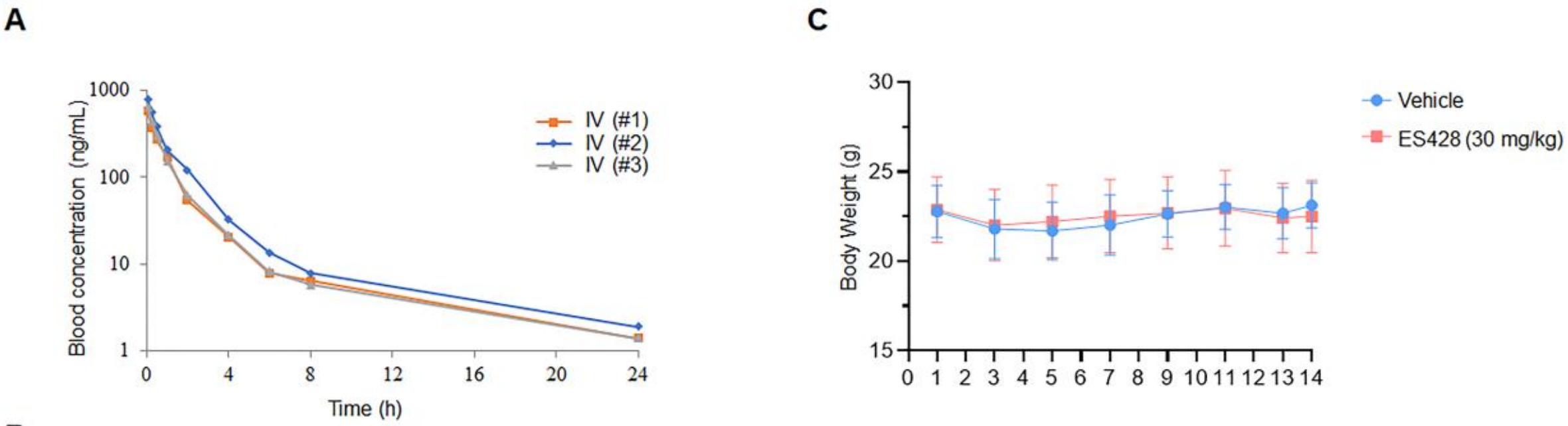

B

	Dose Level	$T_{1/2}$	T_{max}	C_{max}	$AUC_{(0-t)}$	$AUC_{(0-\infty)}$	$MRT_{(0-t)}$	$MRT_{(0-\infty)}$	V_{ss}	V_z	Cl
IV	mg/kg	h	h	µmol/L	h*µmol/L	h*µmol/L	h	h	mL/kg	mL/kg	mL/h/kg
Mean (n=3)	2.0	7.10	0.08	1.12	1.20	1.23	2.14	2.83	7873.31	28566.02	2775.39

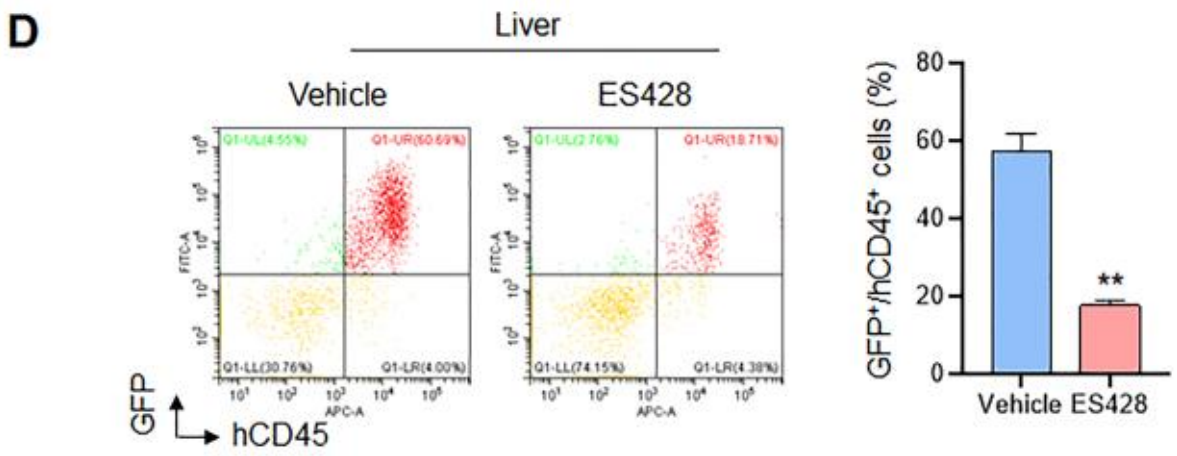

Figure S4 The *in vivo* study of ES428. (A) Plasma concentration at different time points following a single intravenous administration of 2 mg/kg ES428 in SD rats. (B) The pharmacokinetics parameters of ES428. $T_{1/2}$, elimination half-life; C_{max}, maximum (peak) plasma drug concentration; $AUC_{0\text{-}t}$, area under the plasma concentration-time curve from time zero to time t; $AUC_{0\text{-}inf}$, area under the plasma concentration-time curve from time zero to infinity; V_z, apparent volume of distribution during terminal phase; Cl, apparent total body clearance of the drug from plasma; MRT, mean residence time. (C) The body weights were observed in NCG mice following treatment with vehicle or ES428 (30 mg/kg) daily for 2 weeks by intravenous administration. (D) The mice were humanely euthanized at the pre-defined endpoint (see the Method). The percentage of anti-human $CD45^+/GFP^+$ cells in the liver from vehicle- or ES428-treated mice (n ≥ 3 for each group) was determined by flow cytometry. *P<0.05, **P<0.01 and ***P<0.001 against the vehicle group.

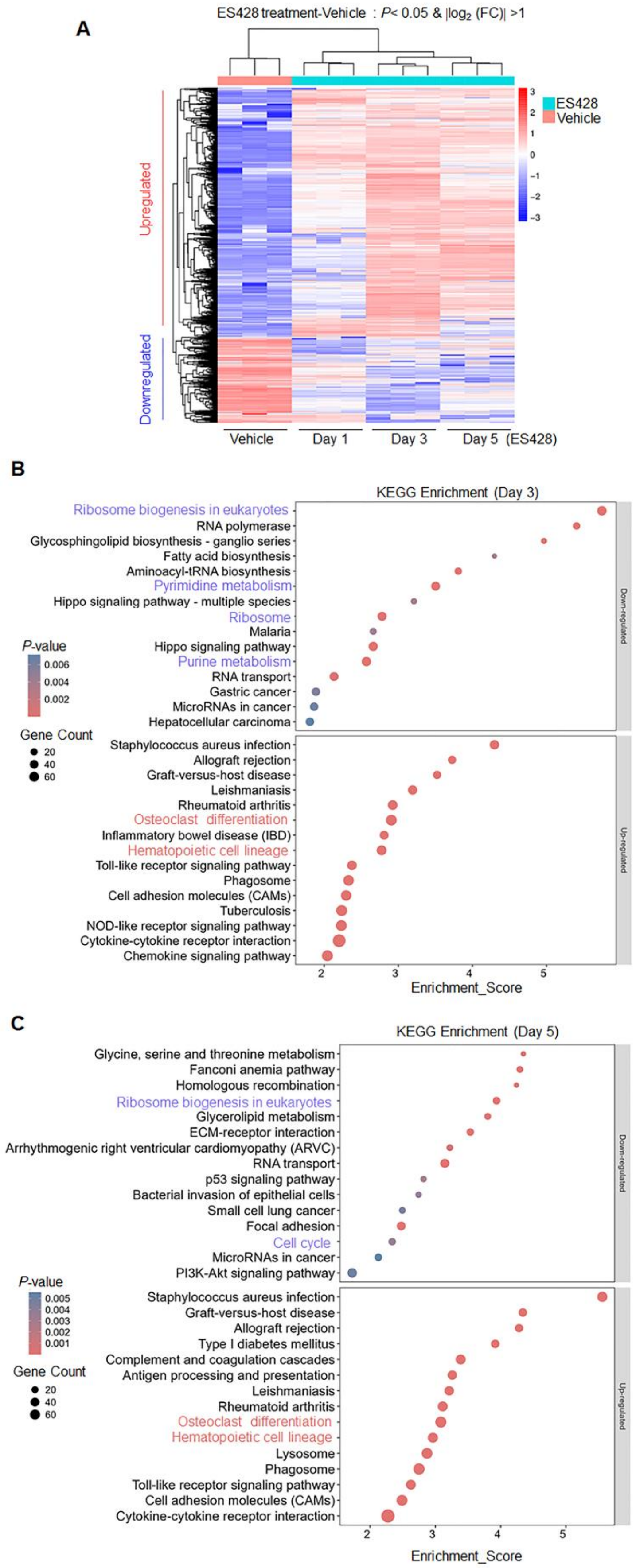

A
ES428 treatment-Vehicle : P< 0.05 & |log2 (FC)| >1
ES428
Vehicle
3
2
1
0
−1
−2
−3
Upregulated
Downregulated
Vehicle
Day 1
Day 3
Day 5 (ES428)
B
KEGG Enrichment (Day 3)
Ribosome biogenesis in eukaryotes
RNA polymerase
Glycosphingolipid biosynthesis - ganglio series
Fatty acid biosynthesis
Aminoacyl-tRNA biosynthesis
Pyrimidine metabolism
Hippo signaling pathway - multiple species
Ribosome
Malaria
Hippo signaling pathway
Purine metabolism
RNA transport
Gastric cancer
MicroRNAs in cancer
Hepatocellular carcinoma
Staphylococcus aureus infection
Allograft rejection
Graft-versus-host disease
Leishmaniasis
Rheumatoid arthritis
Osteoclast differentiation
Inflammatory bowel disease (IBD)
Hematopoietic cell lineage
Toll-like receptor signaling pathway
Phagosome
Cell adhesion molecules (CAMs)
Tuberculosis
NOD-like receptor signaling pathway
Cytokine-cytokine receptor interaction
Chemokine signaling pathway
Down-regulated
Up-regulated
P-value
0.006
0.004
0.002
Gene Count
20
40
60
2
3
4
5
Enrichment_Score
C
KEGG Enrichment (Day 5)
Glycine, serine and threonine metabolism
Fanconi anemia pathway
Homologous recombination
Ribosome biogenesis in eukaryotes
Glycerolipid metabolism
ECM-receptor interaction
Arrhythmogenic right ventricular cardiomyopathy (ARVC)
RNA transport
p53 signaling pathway
Bacterial invasion of epithelial cells
Small cell lung cancer
Focal adhesion
Cell cycle
MicroRNAs in cancer
PI3K-Akt signaling pathway
Staphylococcus aureus infection
Graft-versus-host disease
Allograft rejection
Type I diabetes mellitus
Complement and coagulation cascades
Antigen processing and presentation
Leishmaniasis
Rheumatoid arthritis
Osteoclast differentiation
Hematopoietic cell lineage
Lysosome
Phagosome
Toll-like receptor signaling pathway
Cell adhesion molecules (CAMs)
Cytokine-cytokine receptor interaction
Down-regulated
Up-regulated
P-value
0.005
0.004
0.003
0.002
0.001
Gene Count
20
40
60
2
3
4
5
Enrichment_Score

Figure S5 KEGG analysis of transcriptional profiling following ES428 treatment. (A) Clustering analysis of differentially expressed genes with fold change >2 following ES428 treatment at different time points. (B–C) KEGG analysis for the top 15 enriched pathways among downregulated (top) and upregulated genes (bottom) following ES428 treatment at Day 3 (B) and Day 5 (C). The degree of KEGG enrichment was determined by the enrichment score, *P*-value, and gene number. The sizes and colors of spots represent the number of DEGs and the *P*-value.

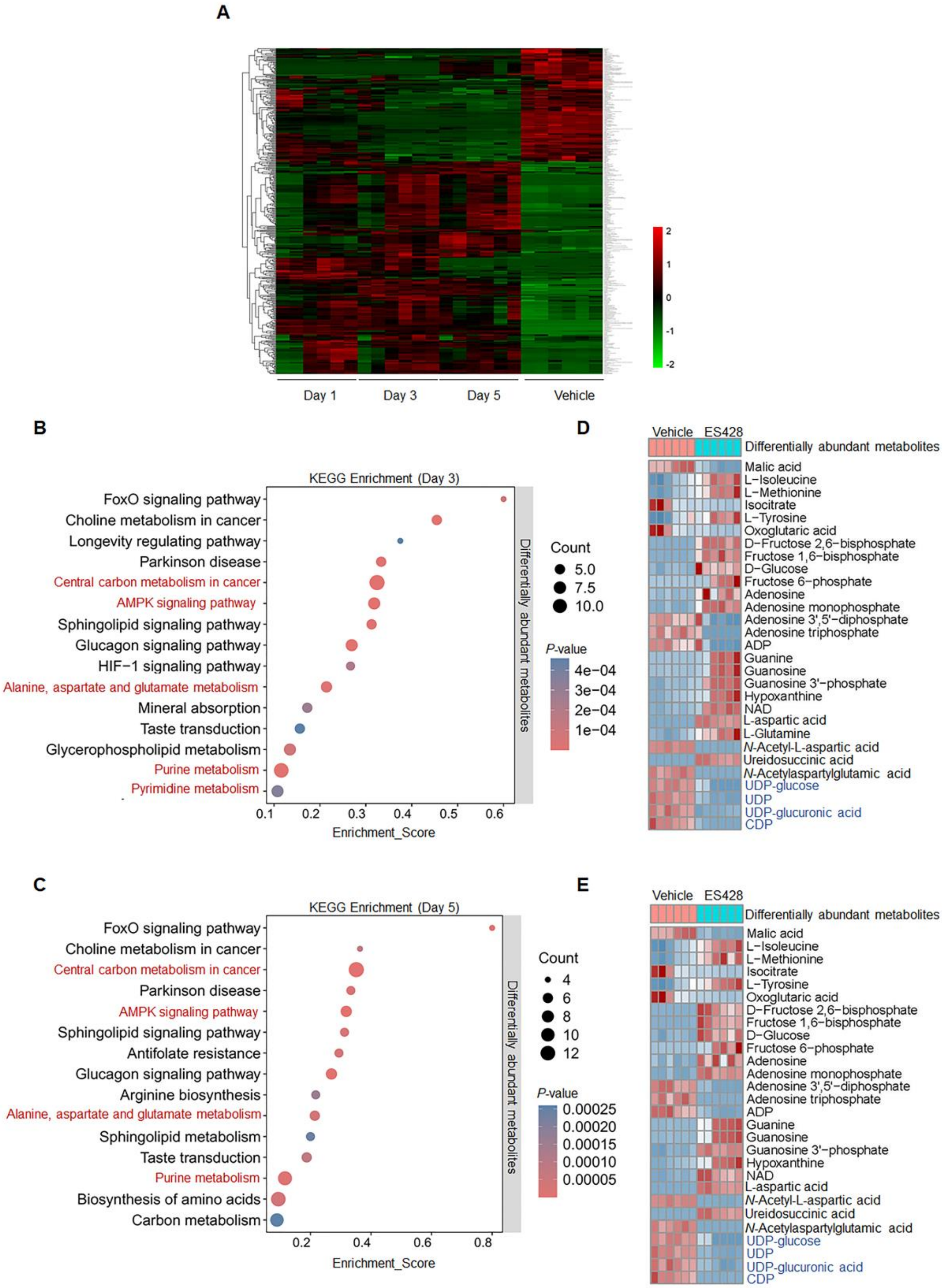

Figure S6 Non-targeted metabolic profiling following ES428 treatment. (A) Clustering analysis of differentially abundant metabolites with fold change >2 following ES428 treatment at different time points. (B–C) KEGG analysis for the top 15 enriched pathways of differentially abundant metabolites following ES428 treatment at Day 3 (B) and Day 5 (C). (D–E) Heatmap

of expression profile of differentially abundant metabolites related to several important metabolic pathways (highlighted with red in B–C) following ES428 treatment at Day 3 (D) and Day 5 (E). Different abundant metabolites related to pyrimidine biosynthesis pathway were highlighted with blue.

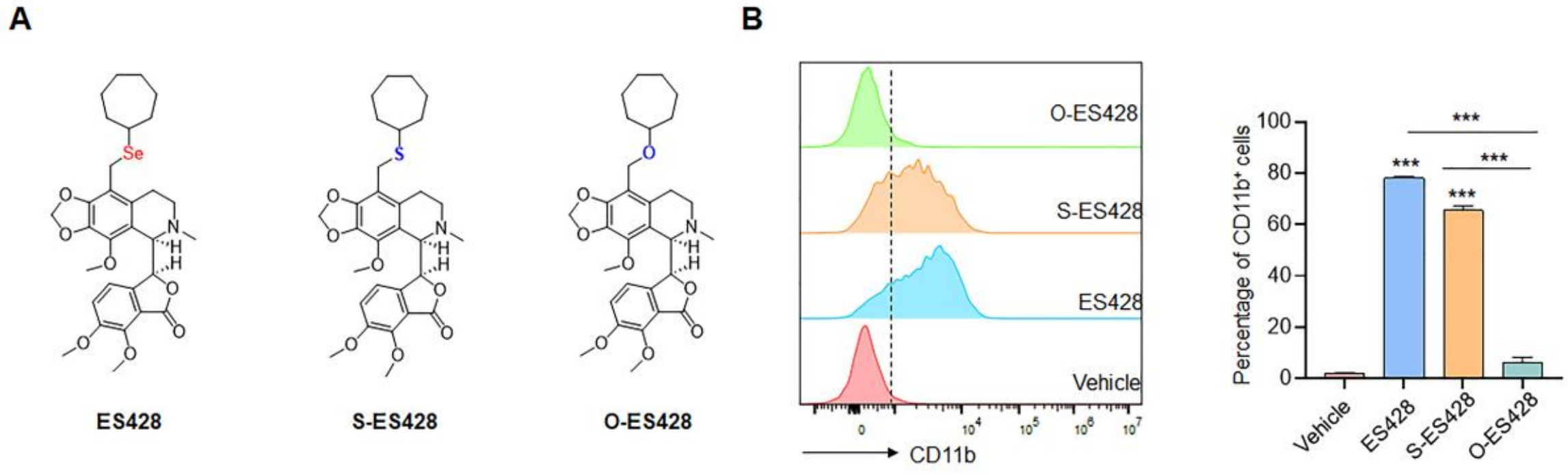

C

	Uniprot ID	Gene name	Docking score
1	Q02127	DHODH	-11.323
2	Q5QJU3	ACER2	-9.758
3	Q96MF6	COQ10A	-9.68
4	P06126	CD1A	-9.448
5	Q99424	ACOX2	-9.257
6	P00156	MT-CYB	-9.252
7	O00116	AGPS	-9.123
8	Q16850	CYP51A1	-8.645
9	Q92503	SEC14L1	-8.626
10	Q13635	PTCH1	-8.558
11	P19652	ORM2	-8.517
12	Q8NAN2	MIGA1	-8.463
13	P55058	PLTP	-8.292
14	P08574	CYC1	-8.107
15	Q71F56	MED13L	-8.084
16	Q9UBS5	GABBR1	-8.04
17	P16662	UGT2B7	-8.007

Figure S7 Computational target-fishing results of S-ES428. (A) Chemical structures of ES428, S-ES428 and O-ES428 are shown. (B) U937 cells were treated with compounds as indicated at an equivalent dose of 2 μmol/L for 3 days. Flow cytometry analysis of the percentages of $CD11b^{+}$ cells was shown. Error bars represent the mean ± SD of triplicate samples in an independent experiment. **P*<0.05, ***P*<0.01 and ****P*<0.001 against the vehicle-treated or between the line-pointed group. Results are representative of at least three independent experiments. (C) This model combines ligand-based and structure-based approaches for target identification, utilizing an innovative hybrid human protein structure database that encompasses both experimentally determined structures and those predicted by AlphaFold2. Candidate proteins with Glide docking scores below −8.0 were shown.

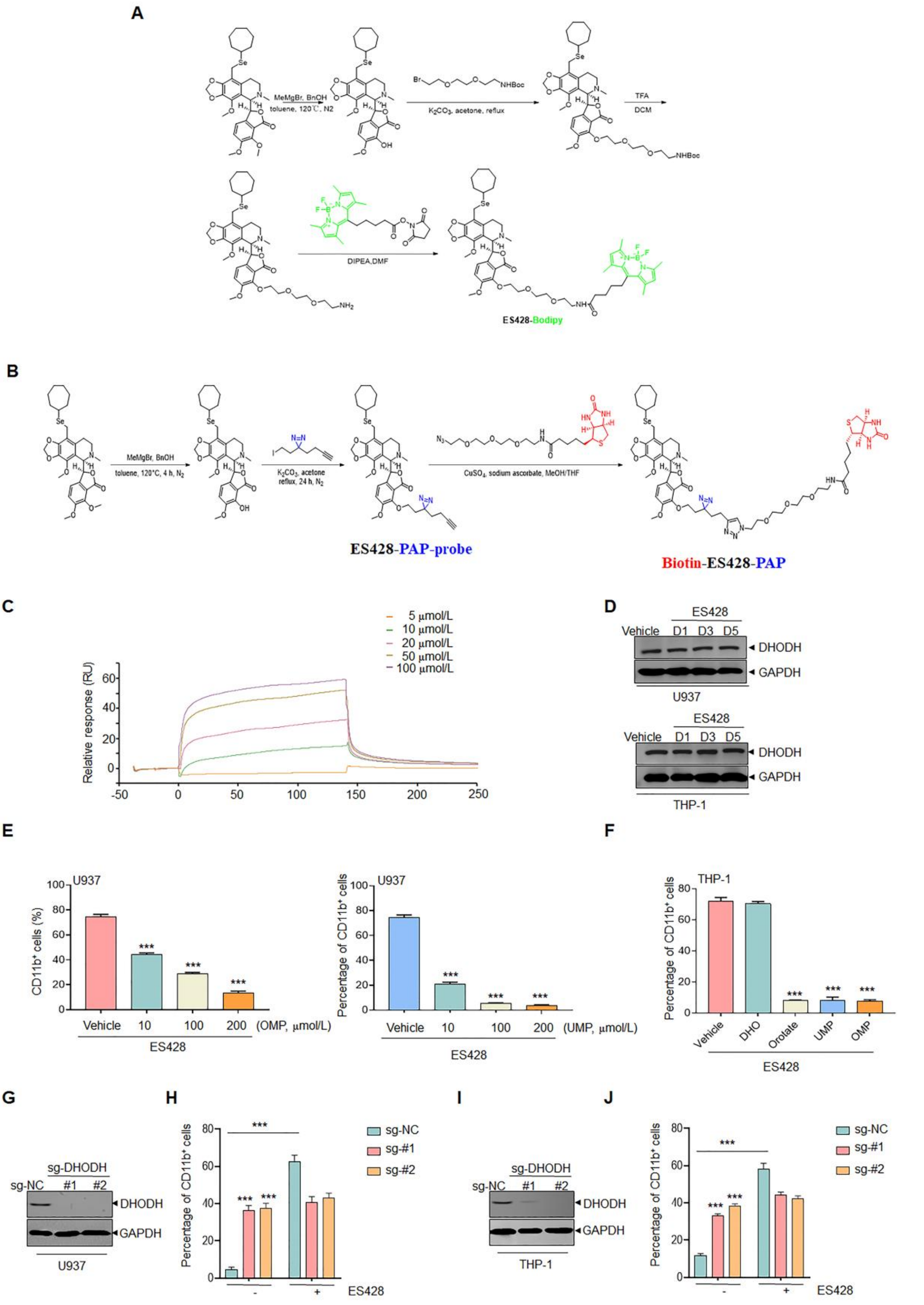

Figure S8 DHODH is the direct functional target of ES428. (A–B) Synthesis and Chemical structure of ES428-Bodipy (A) and ES428-PAP (B) probes. (C) The real-time interaction between ES428 and the rhDHODH immobilized on the sensor chip surface. (D) U937 cells

(top) and THP-1 cells (bottom) were incubated with ES428 at 2 μmol/L for different time points as indicated, the protein levels of DHODH were detected by western blotting. (E–F) U937 cells (E) and THP-1 cells (F) were incubated with ES428 of 2 μmol/L in the presence or absence of supplementation of metabolites as indicated for 3 days. The differentiation-inducing activity was evaluated by anti-human CD11b staining and FACS analysis. (G–J) U937 cells (G–H) and THP-1 cells (I–J) stably expressing DHODH sgRNA were treated with ES428 for 3 days and the differentiation-inducing activity was evaluated by anti-human CD11b staining and FACS analysis (H&J). The protein level of DHODH overexpression was detected through western blotting (G&I). Results are representative of at least three independent experiments. Error bars represent the mean ± SD of triplicate samples in an independent experiment. *$P<0.05$, **$P<0.01$ and ***$P<0.001$ against the vehicle-treated or between the line-pointed group.

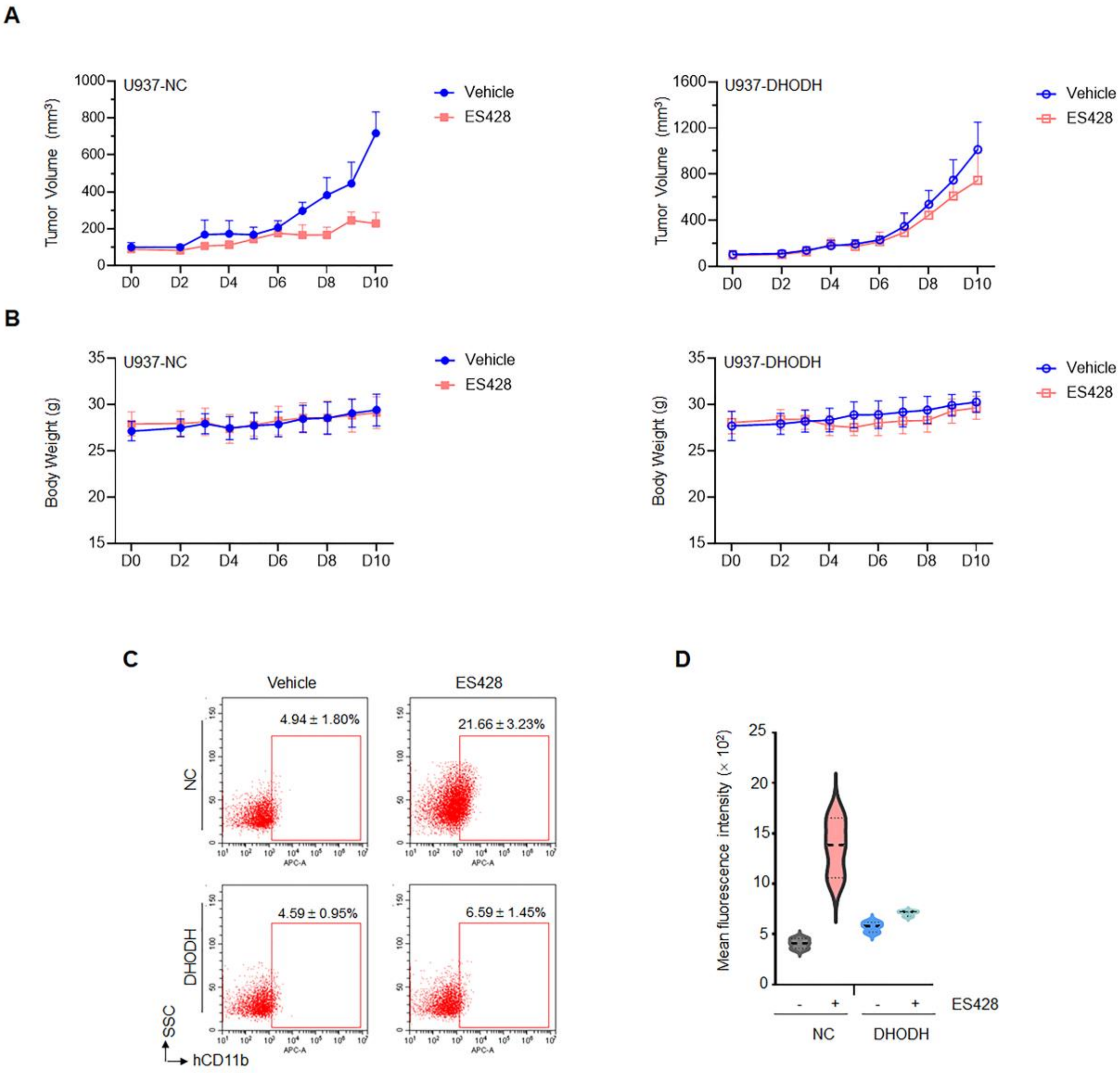

Figure S9 *In vivo* validation of DHODH as the direct functional target of ES428. (A) U937 cells overexpressing DHODH or NC control were injected i.v. in NCG mice. The tumor-bearing mice were grouped and treated with vehicle and ES428. The tumor volume was measured following intravenous administration of ES428 for continuous 10 days at a dosage of 30 mg/kg ($n \geq 6$ for each group). (B) The body weight of the four groups was measured following treatment as indicated. (C–D) Mice were humanely euthanized at the pre-defined endpoint (see the "Method"). The percentage of anti-human $CD11b^+$ cells of the single cell suspensions derived from tumor tissue of each group ($n \geq 3$) was evaluated by flow cytometry analysis (C). The MFI were quantified in (D). Error bars represent the mean ± SD in each group.

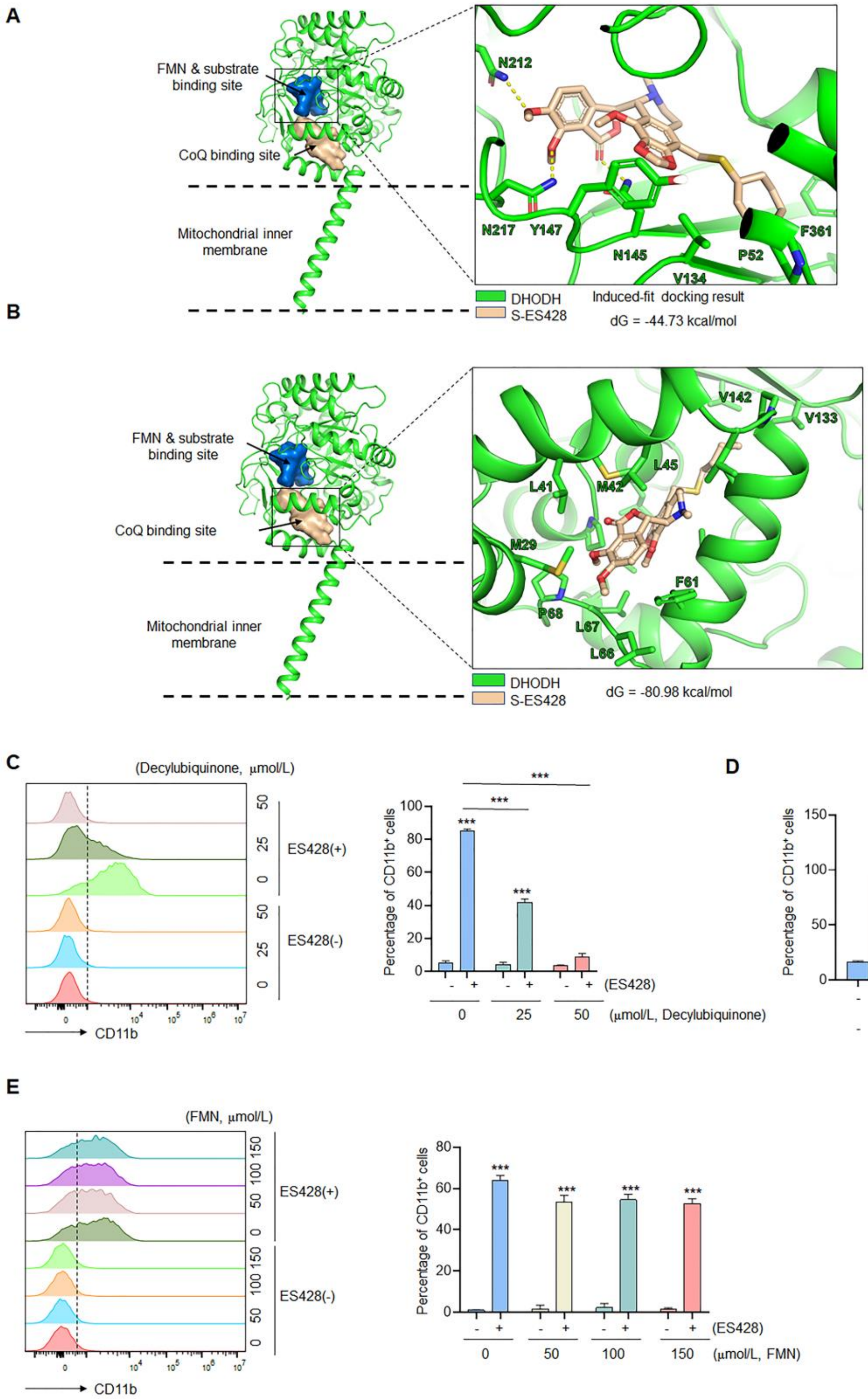

Figure S10 ES428 preferentially occupies the CoQ-binding pocket of DHODH. (A–B) The full-length DHODH chimera structure was developed through homology modeling with a truncated DHODH crystal structure (PDB: 4IGH, residues 32–395) as a template. SiteMap analysis pinpoints two potential ligand-binding sites in DHODH: one corresponding to the

FMN-binding site and the substrate positioning site, and the other one to the CoQ binding cavity. ES428 was docked into the FMN & substrate binding site (A) and the CoQ binding site (B) of DHODH. The Gibbs free energy change (dG) was calculated. ES428's preferential binding to the CoQ-binding pocket was indicated by lower binding free energy. (C–E) U937 cells were incubated with ES428 in the presence or absence of decylubiquinone (C), MitoQ (D), and FMN (E) at concentrations as indicated for 3 days. The differentiation-inducing activity was evaluated by anti-human CD11b staining and FACS staining. Error bars represent the mean ± SD of triplicate samples in an independent experiment. *P<0.05, **P<0.01 and ***P<0.001 against the vehicle-treated or between the line-pointed group. Results in (C–E) are representative of at least three independent experiments.

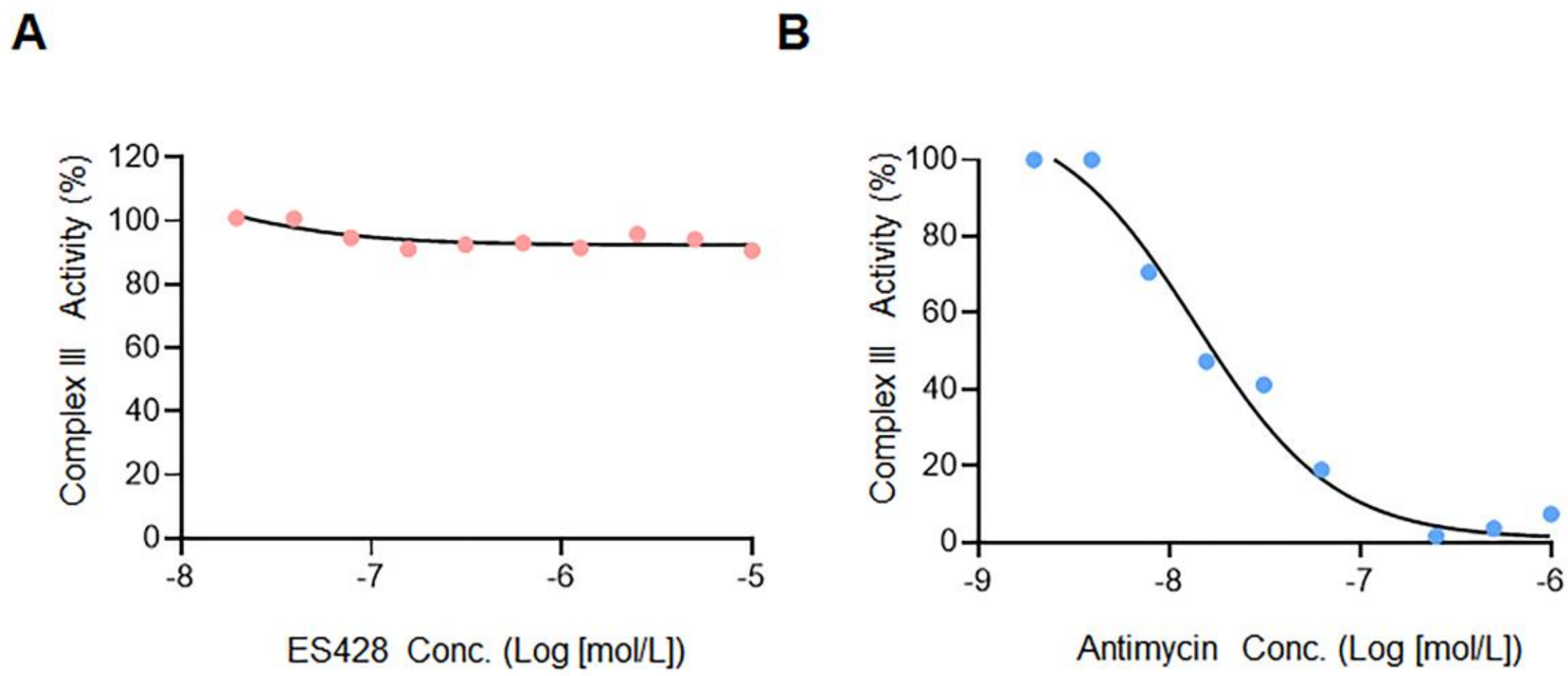

Figure S11 The *in vitro* enzyme activity of Complex III. (A–B**)** The effects of ES428 (A) and positive control compound antimycin (B) were assessed through a commercial mitochondrial complex III activity assay using isolated mitochondria fraction.

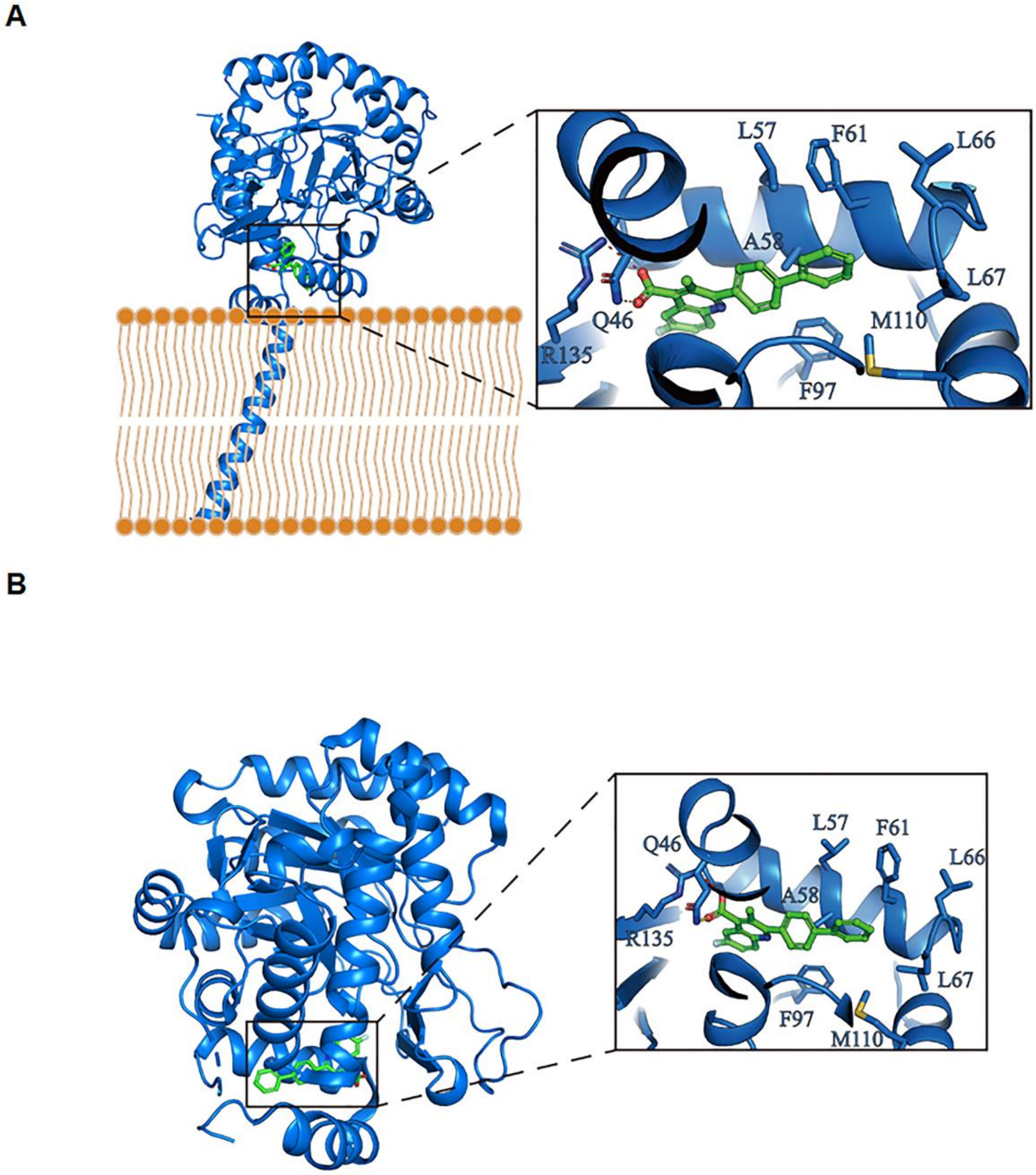

Figure S12 The interaction patterns between BRQ and DHODH obtained through the MD simulations. Clustered interaction pattern between BRQ and full-length DHODH with lipid-bound complexes (A) and with DHODH-NΔ30 in the absence of a PE bilayer (B) over the last 100 ns of trajectory. The protein residues surrounding the ligand constituted the interacting sites. The hydrogen bonds with R135 and Q46 were shown as yellow and red dashed lines, respectively.

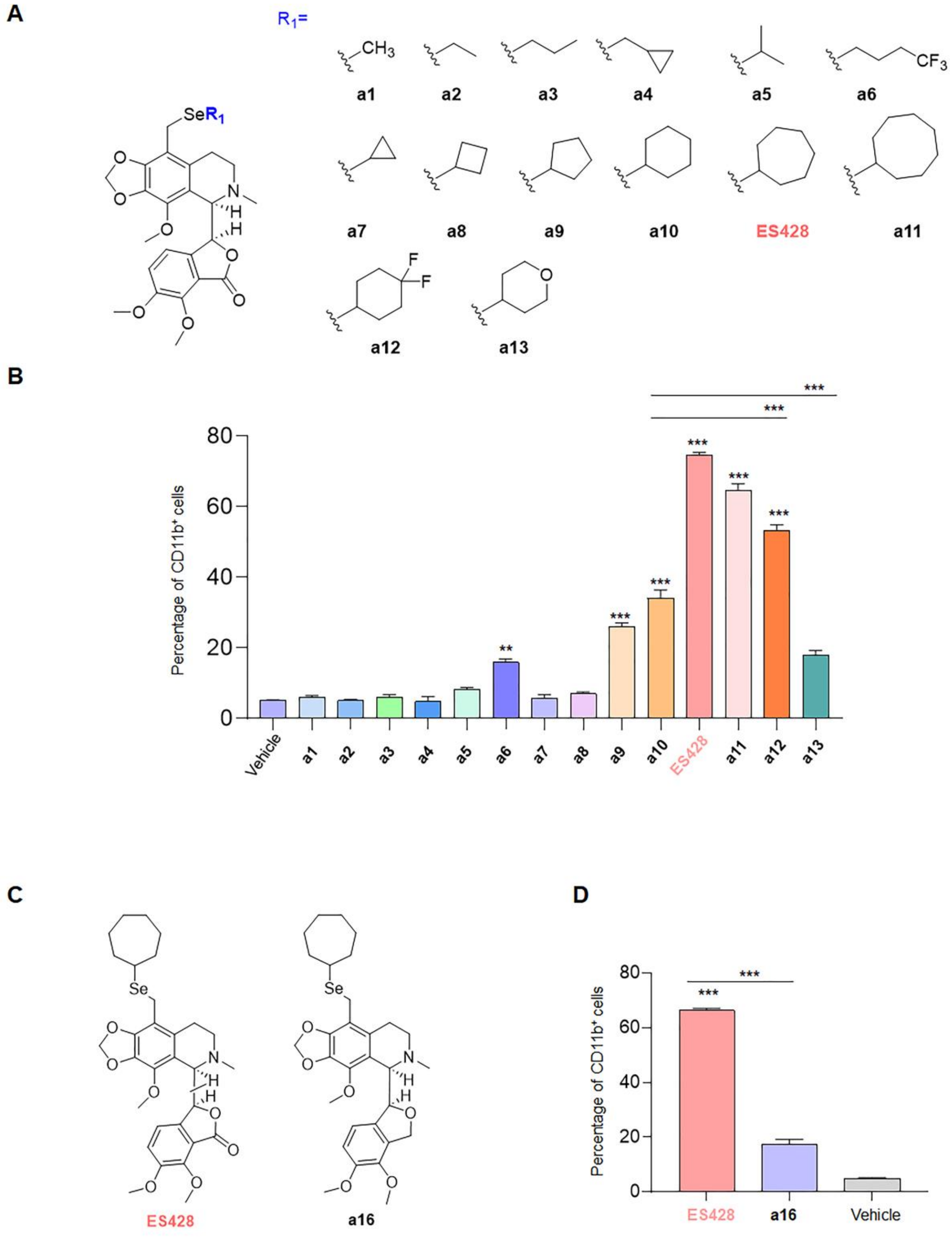

Figure S13 The differentiation-inducing activities of analogs modified at the 9′ and 1 position of ES428. (A) Chemical structures of modified analogs at the 9′ position. (B) U937 cells were treated with different analogs as indicated at equivalent dose of 2 μmol/L for 3 days. Flow cytometry analysis of the percentages of $CD11b^+$ cells was shown. (C) Chemical structures of cyclic ether analog **a16**. (D) U937 cells were treated with ES428 or **a16** at a concentration of 2 μmol/L for 3 days. Flow cytometry analysis of the percentages of $CD11b^+$ cells was shown. Error bars represent the mean ± SD of triplicate samples in an independent experiment.

P<0.05, ***P*<0.01 and ****P*<0.001 against the vehicle-treated or between the line-pointed group. Results in (B) and (D) are representative of at least three independent experiments.

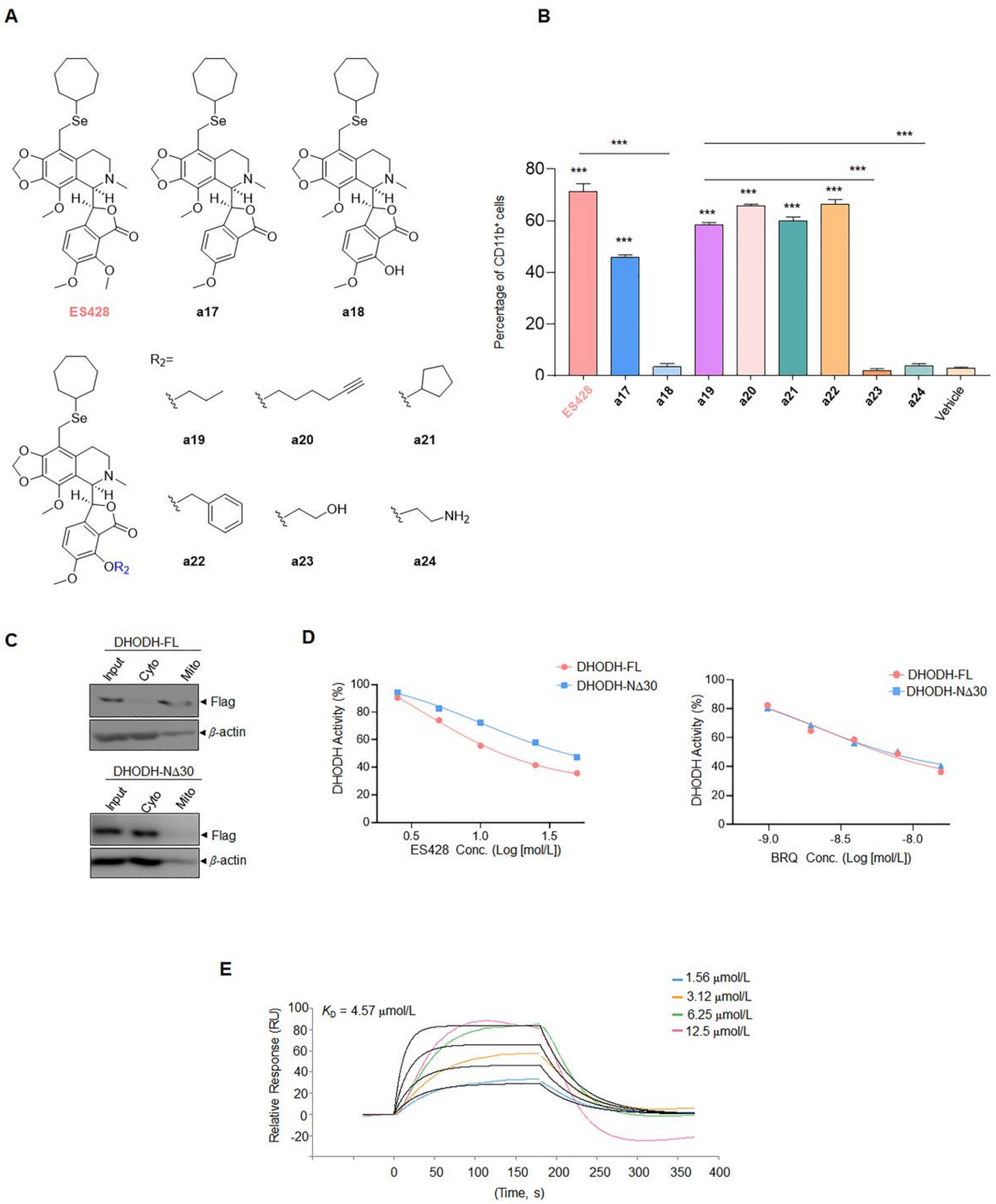

Figure S14 The functional involvement of ES428's interaction with lipids. (A) Chemical structures of modified analogs of ES428 at the 7-position. (B) U937 cells were treated with different analogs as indicated at equivalent dose of 2 μmol/L for 3 days. Flow cytometry analysis of myeloid differentiation cell surface antigens CD11b were shown. (C) Western blotting showing DHODH protein levels in cytosolic and mitochondrial fractions from 293T cells that express the indicated DHODH constructs. (D)The inhibitory effect of ES428 (left) and reference compound BRQ (right) using a kinetic DHODH enzymatic assay *in vitro* based on cell lysate derived from 293T cells that express the indicated DHODH constructs as the enzyme source. (E) ES428 was applied for binding with POPC liposome surface immobilized

on the L1 sensor chip through SPR-based interaction analyses. Error bars represent the mean ± SD of triplicate samples in an independent experiment. *$P<0.05$, **$P<0.01$ and ***$P<0.001$ against the vehicle-treated group or between the line-pointed group.

Compound	ES428	a14	a15	a17	a18	a19	a22	a23
Caco-2 permeability	-4.82	-4.851	-4.807	-4.757	-4.885	-4.679	-4.803	-4.815

Figure S15 The cell membrane permeability prediction. The cell membrane permeability prediction of a series of substituted analogs was performed through ADMETlab 3.0.

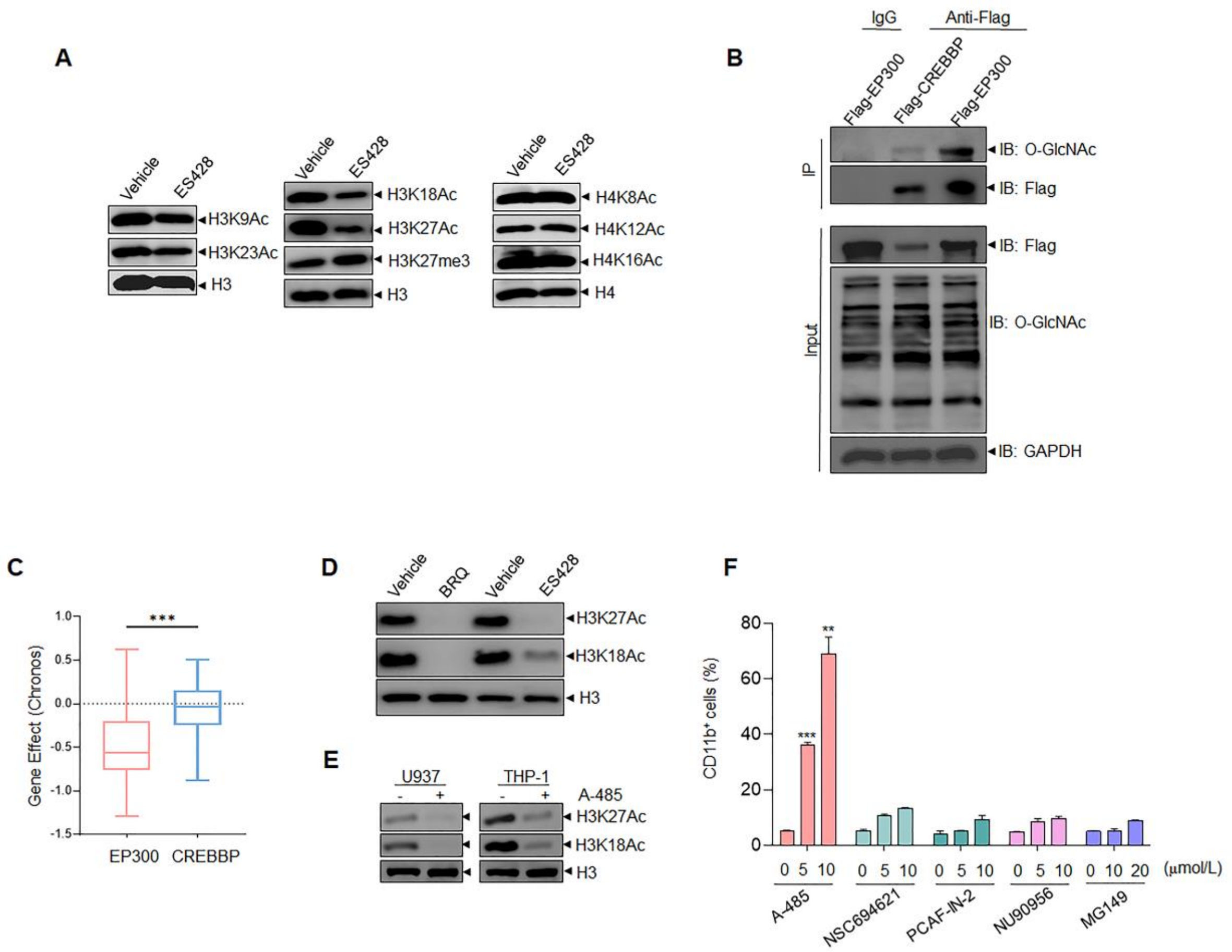

Figure S16 EP300/CREBBP catalytic inhibition was functionally invovled in ES428-induced differentiation. (A) U937 cells were incubated with ES428 at 2 μmol/L for 1 day. The expression of histone marks as indicated was detected by western blotting. (B) HEK293T cells that transiently overexpressed Flag-EP300 or Flag-CREBBP were harvested and the immunoprecipitation assay using anti-FLAG agarose beads was performed. The immunoprecipitated protein complex (top panel) and the corresponding lysates (input, bottom panel) were detected with antibodies against the FLAG-tag and O-GlcNAc antibody. (C) The dependency scores analysis of EP300 and CREBBP in AML using the Cancer Dependency Map (DepMap) data. A score getting close to -1 represents the gene is essential. (D) U937cells were incubated with ES428 at 2 μmol/L or BRQ at 0.5 μmol/L for 3 days. The expression of proteins as indicated was detected by western blotting. (E) U937 cells and THP-1 cells were incubated with A485 at 5 μmol/L for 1 day. The expression of proteins as indicated was detected by western blotting. (F) U937 cells were treated with different inhibitors as indicated at different concentrations for 3 days. Flow cytometry analysis of myeloid differentiation cell surface antigens CD11b were shown. Error bars represent the mean ± SD of triplicate samples in an independent experiment. **P*<0.05, ***P*<0.01 and ****P*<0.001 against the vehicle-treated group or between the line-pointed group.

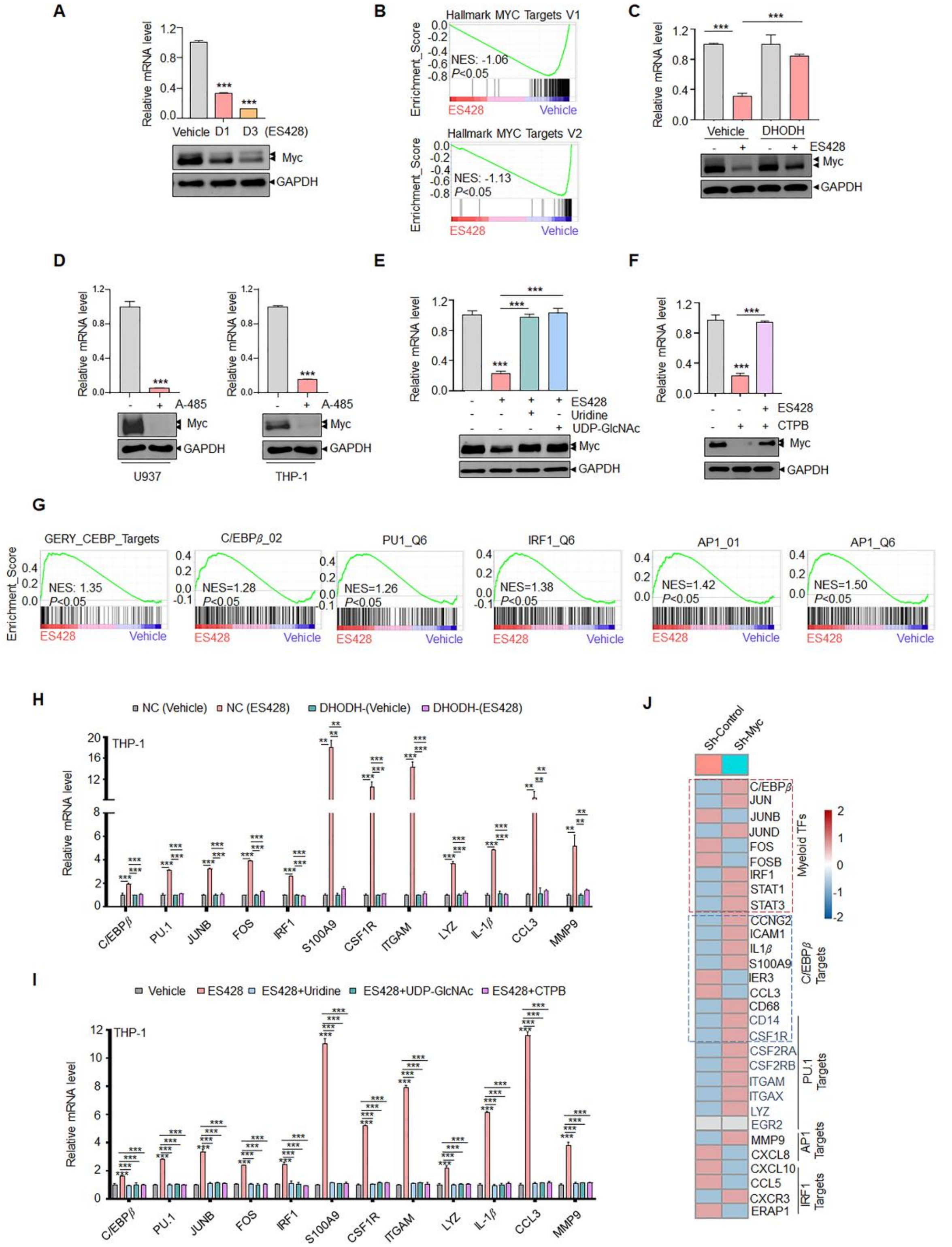

Figure S17 EP300/CREBBP inhibition-mediated transcriptional regulation was critical for ES428-induced differentiation. (A) THP-1 cells were incubated with ES428 at 2 μmol/L for different time points as indicated, the mRNA (top) and protein (bottom) levels of Myc were detected by quantitative real-time PCR and western blotting, respectively. GAPDH was detected as the loading control. (B) Enrichment plots of gene sets for through GSEA analysis

of transcriptional profiling following ES428 treatment at Day 3 are shown. NES, normalized enrichment score. (C) THP-1 cells stably overexpressing DHODH were treated with ES428 for 3 days. The mRNA (top) and protein (bottom) level of Myc were detected by quantitative real-time PCR and western blotting, respectively. (D) U937 and THP-1 cells were incubated with A-485 at 5 μmol/L. The mRNA (top) and protein (bottom) levels of Myc were detected at day 1 by quantitative real-time PCR and western blotting, respectively. (E–F) THP-1 cells were incubated with ES428 at 2 μmol/L with or without the addition of excess uridine or UDP-GlcNAc (E) as well as CTPB (F). The mRNA (top) and protein (bottom) levels of Myc were detected at day 1 by quantitative real-time PCR and western blotting, respectively. (G) Enrichment plots of gene sets for through GSEA analysis of transcriptional profiling following ES428 treatment at Day 3 are shown. NES, normalized enrichment score. (H–I) THP-1 cells stably overexpressing DHODH were treated with ES428 (H); and THP-1 cells were incubated with ES428 at 2 μmol/L with or without the addition of excess uridine, UDP-GlcNAc or CTPB (I). The mRNA levels of myeloid TFs and their representative target genes as indicated were detected at day 1 by quantitative real-time PCR. (J) Heatmap of expression profile of differentially expressed genes following Myc knockdown in MOLM-13 cells based on previously published RNA-seq data (GSE216977). These genes are related to several important myeloid TFs involved in myeloid differentiation, and their representative target genes. The myeloid TFs are shown as red dashed box and C/EBPβ target genes are shown as blue dashed box. Error bars represent the mean ± SD of triplicate samples in an independent experiment. **P*<0.05, ***P*<0.01 and ****P*<0.001 against the vehicle-treated group or between the line-pointed group.

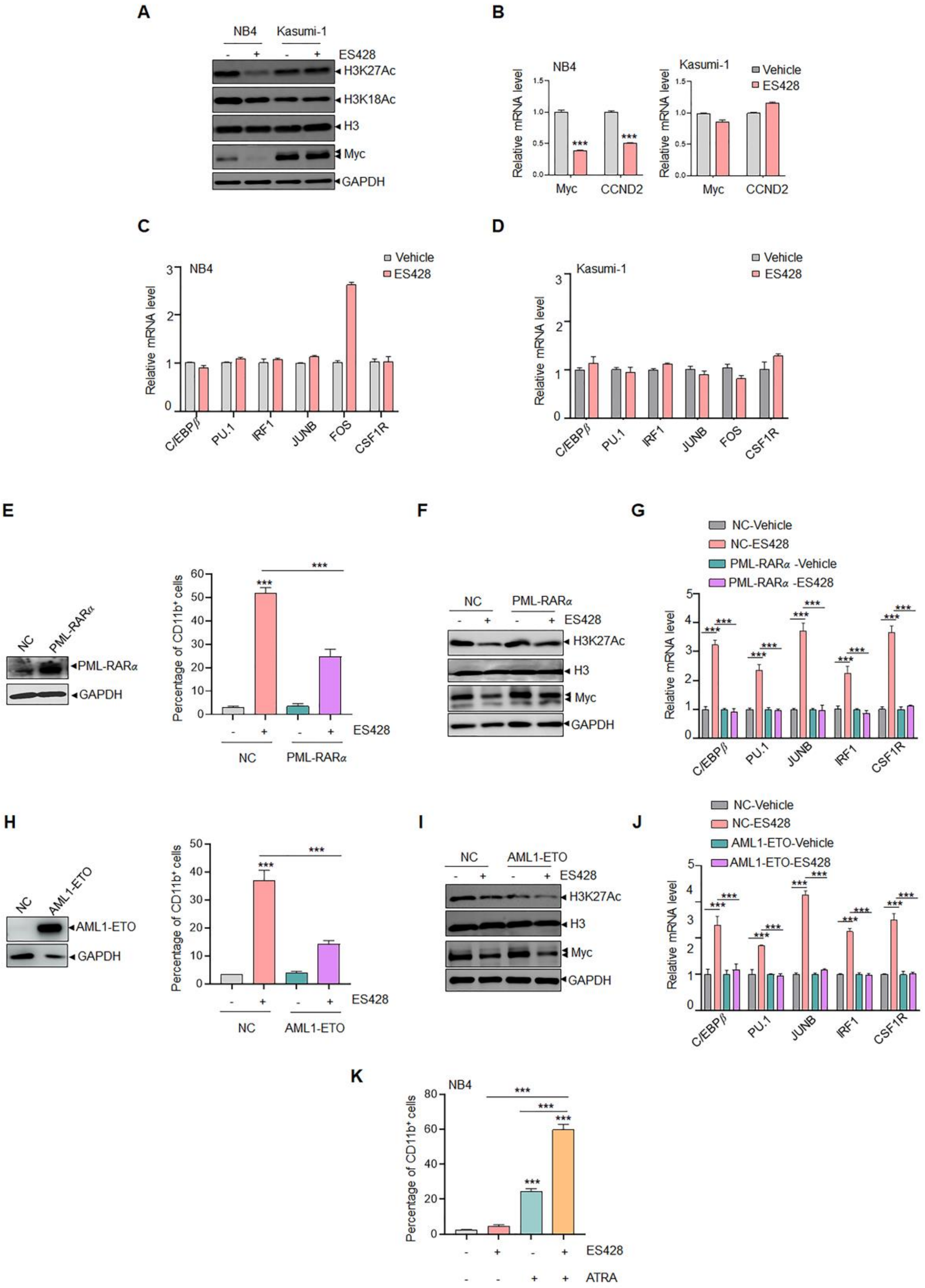

Figure S18 PML-RARα^+ and AML1-ETO$^+$ cells exerted resistance to ES428-induced differentiation. (A) NB4 and Kasumi-1 cells were incubated with ES428 at 2 μmol/L for 3 days. The protein levels as indicated were detected by western blotting. (B–D) NB4 and Kasumi-1 cells were incubated with ES428 at 2 μmol/L. The mRNA levels as indicated were detected at

day 1 by quantitative real-time PCR. (E–J) U937 cells stably overexpressing PML-RAR*α* (E–G) and AML1-ETO (H–J) were treated with ES428 for 3 days and the differentiation-inducing activity was evaluated by anti-human CD11b staining and FACS analysis. The protein level was detected through western blotting (E and H). The protein levels as indicated were detected by western blotting (F and I). The mRNA levels representative genes as indicated were detected at day 1 by quantitative real-time PCR (G and J). (K) NB4 cells were incubated with ES428 at 2 μmol/L with or without the addition of ATRA at 1 nmol/L for 3 days. The differentiation-inducing activity was evaluated by anti-human CD11b staining. Results are representative of at least three independent experiments. Error bars represent the mean ± SD of triplicate samples in an independent experiment. *P<0.05, **P<0.01 and ***P<0.001 against the vehicle group and between the line-pointed group.

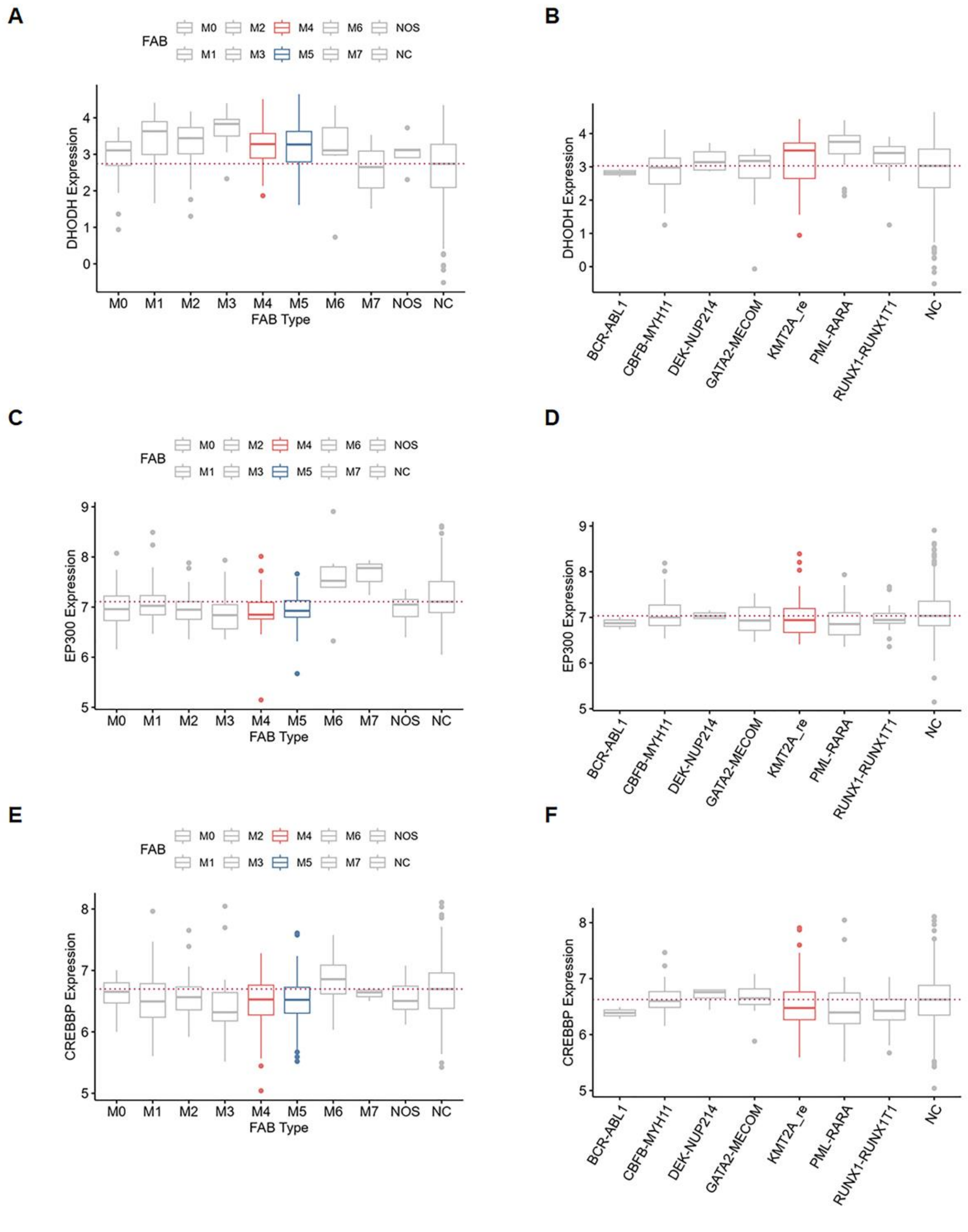

Figure S19 The related gene expression across different AML subtypes defined by FAB classification or recurring genetic abnormalities. The normalized gene expression of DHODH (A–B), EP300 (C–D), and CREBBP (E–F) in AML patients with different maturation states determined by FAB classification (A, C, E) and recurring genetic abnormalities (B, D, F) in the BeatAML2.0 dataset. AML FAB M4 and M5 patients are indicated in red and blue in (A, C, E), respectively. AML with rearrangement of lysine methyltransferase 2a gene (KMT2A_re) is indicated in red in (B, D, F). KMT2A_re is commonly known as mixed lineage leukemia (MLL) and predominantly associated with FAB-M4/M5 subtypes.

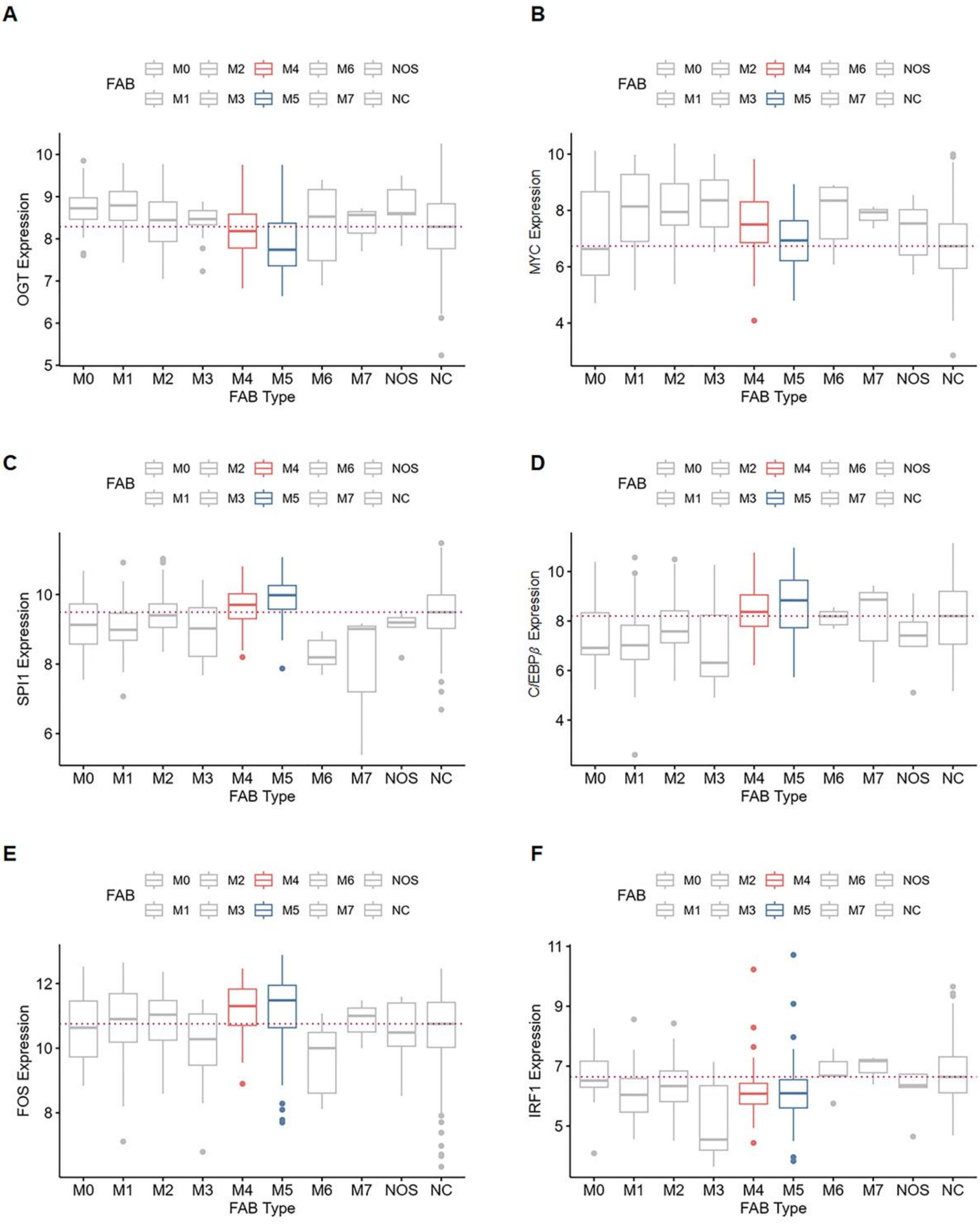

Figure S20 The expression of key hematopoietic TFs across different AML subtypes defined by the FAB classification. The normalized gene expression of OGT (A), Myc (B), SPI1 (C), C/EBPβ (D), FOS (E) and IRF1 (F) of AML patients with different maturation states determined by FAB classification in the BeatAML2.0 dataset. AML FAB M4 and M5 patients are indicated in red and blue, respectively.

4. NMR spectra of compounds

^{1}H NMR of compound **3**

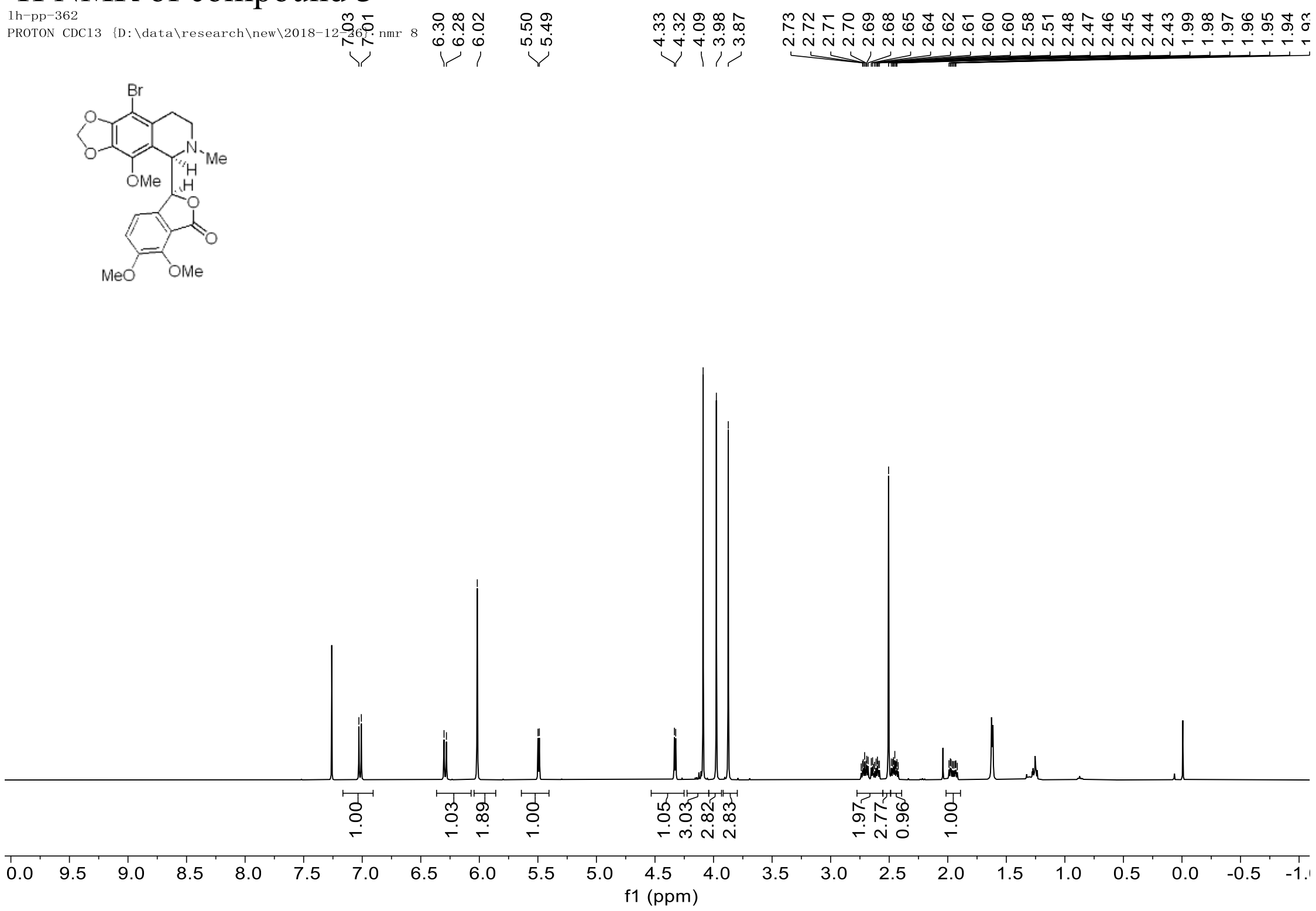

^{1}H NMR of compound **7**

Y30161073-0403-372

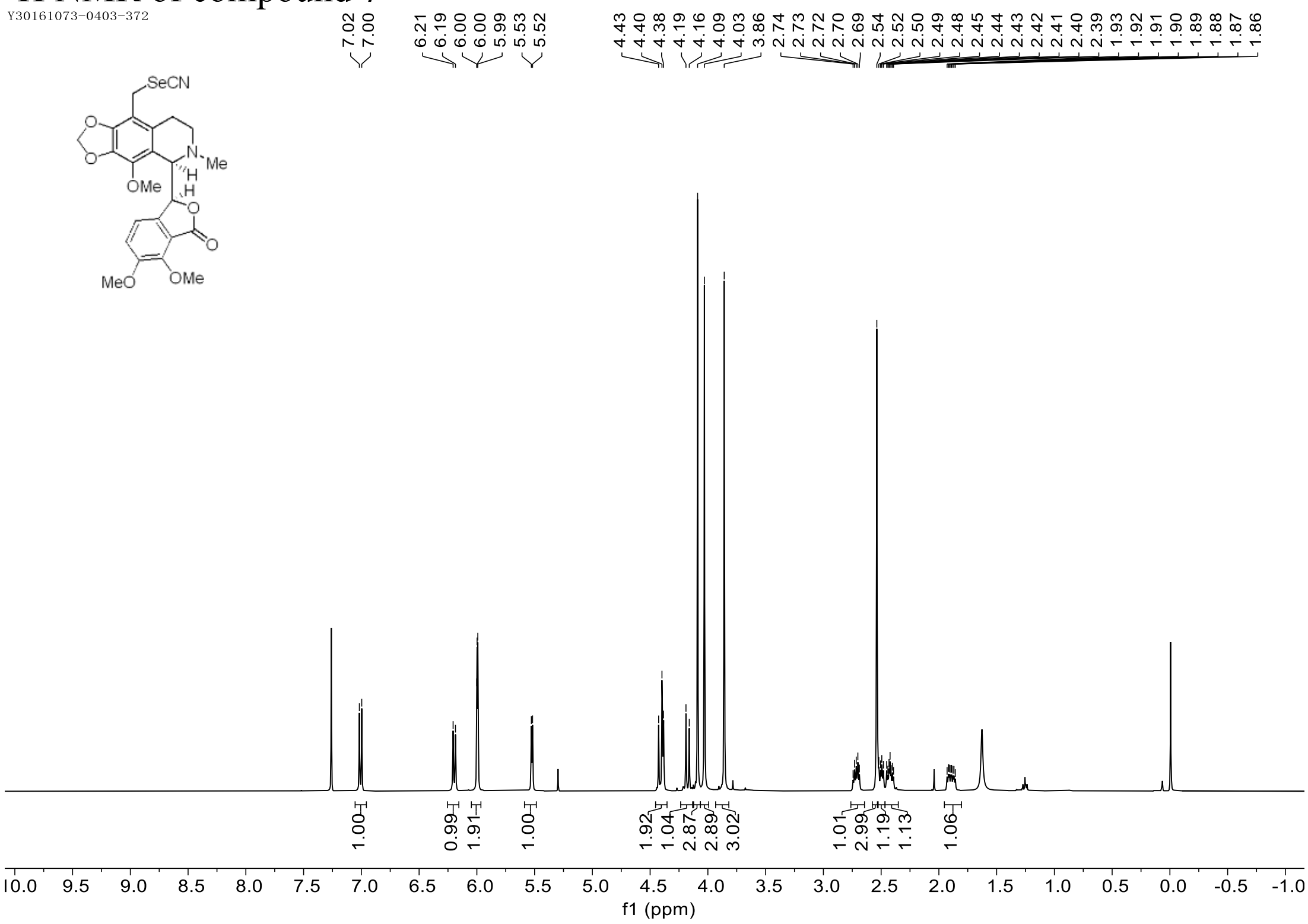

^{13}C NMR of compound **7**

Y30161073-0319-372

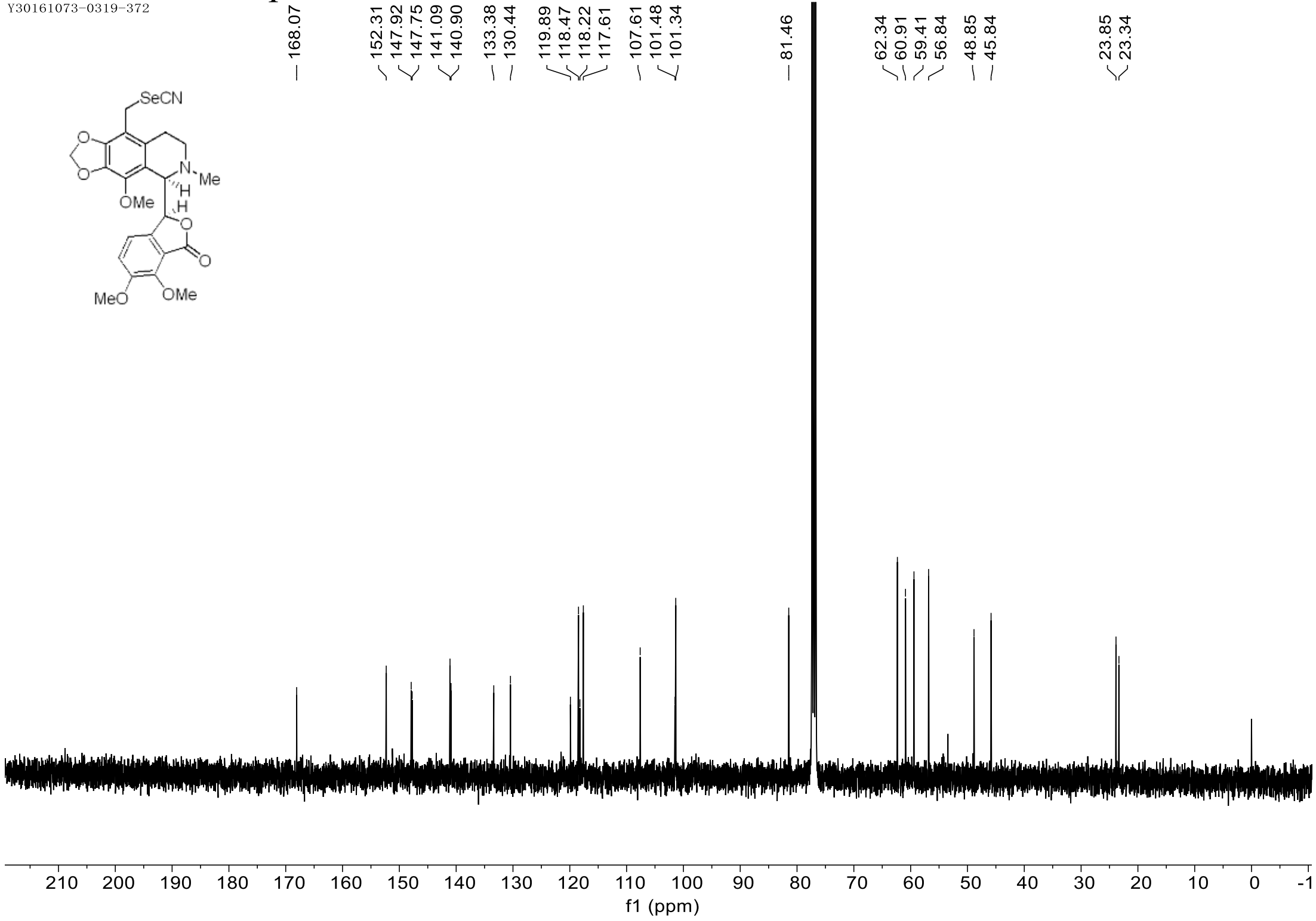

^1H NMR of compound **a1**

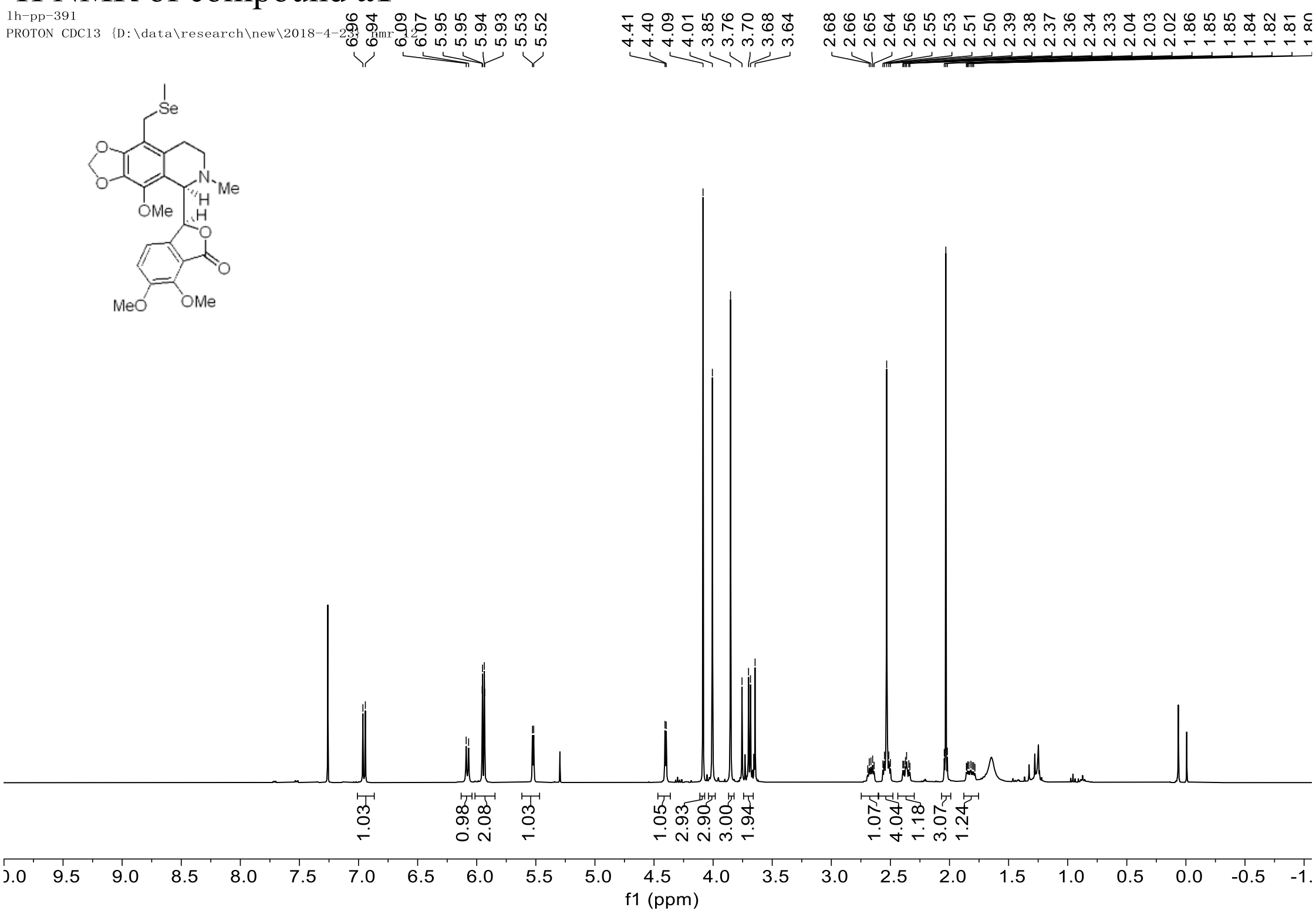

^{13}C NMR of compound **a1**

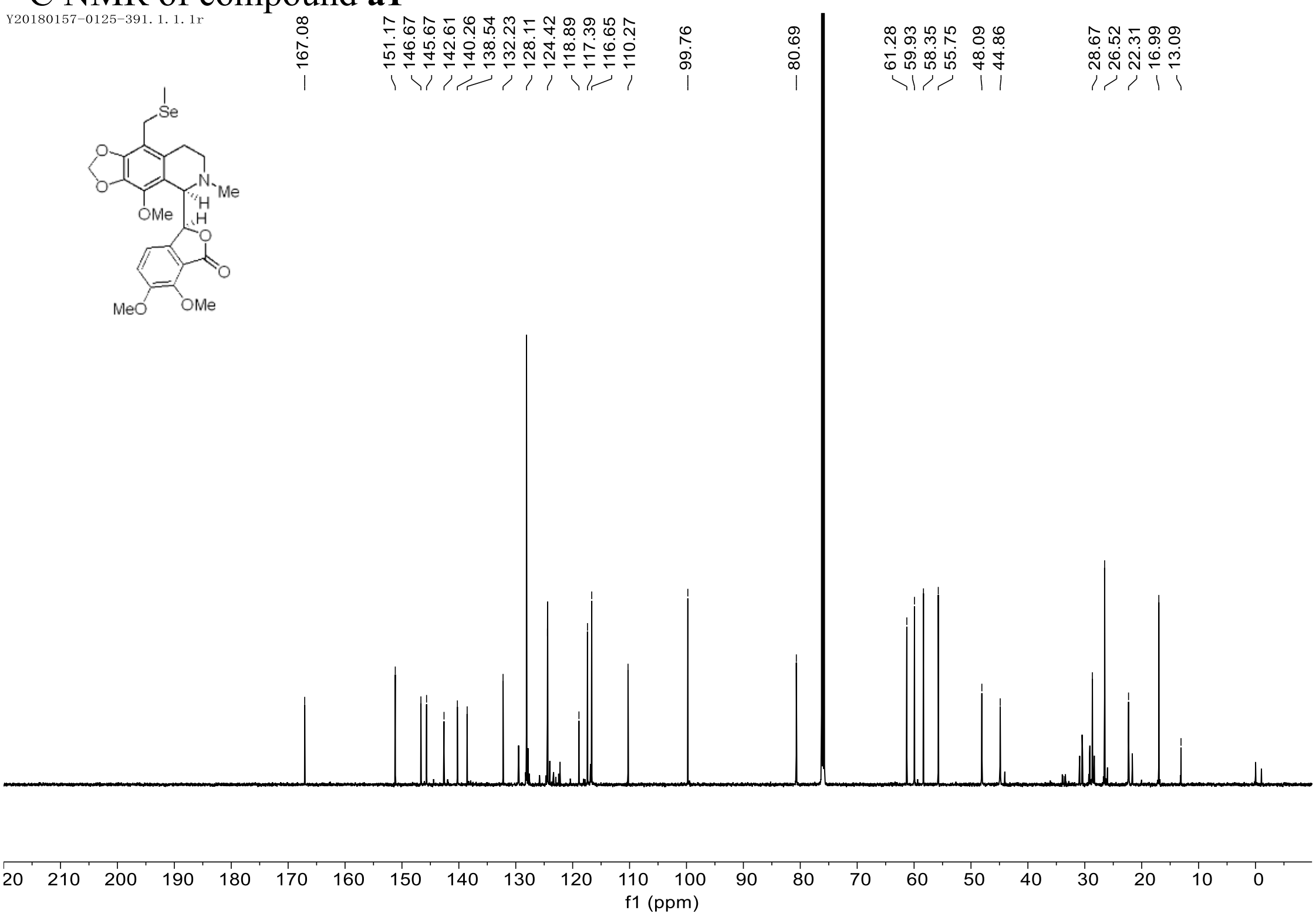

^{1}H NMR of compound **a2**

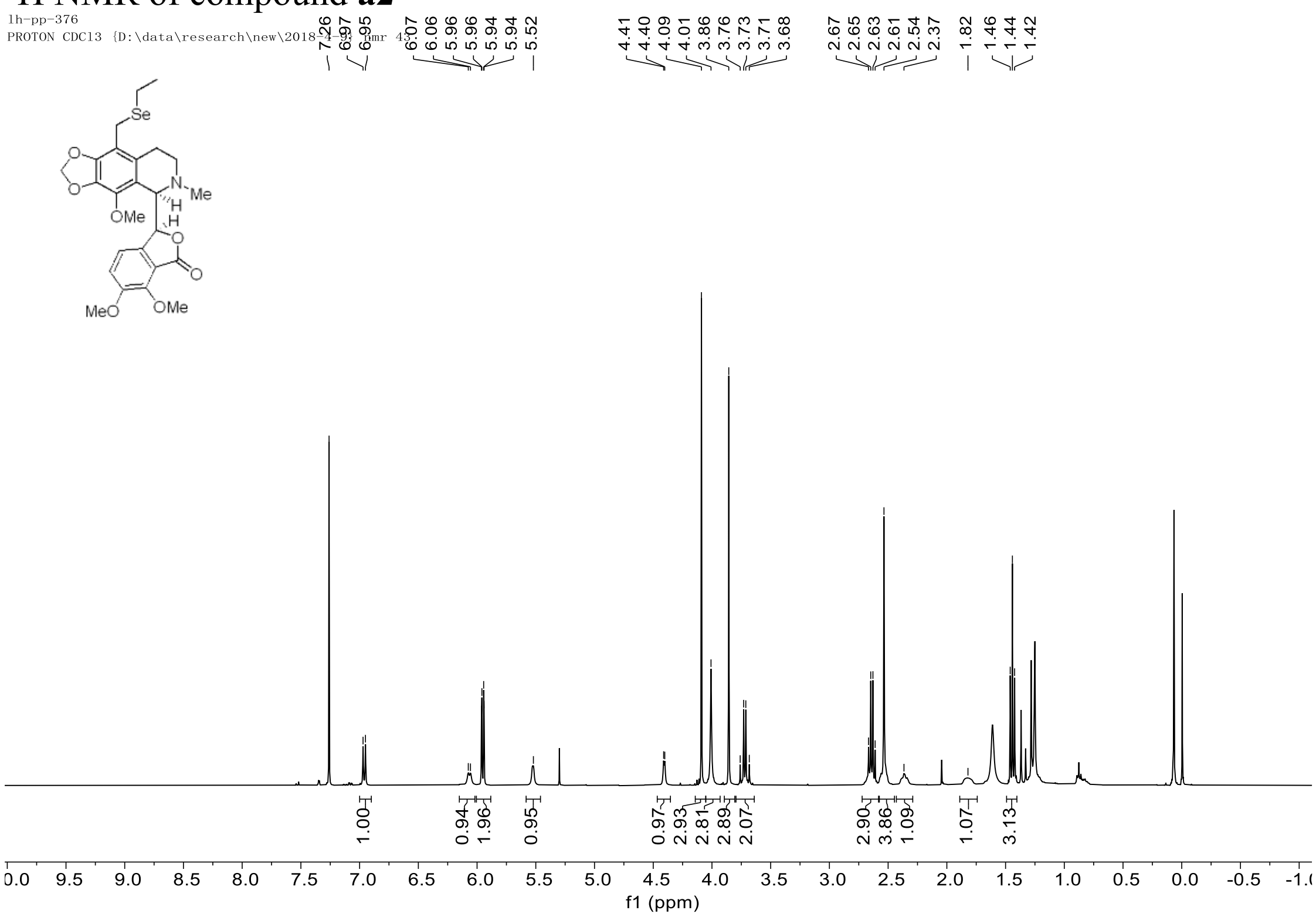

^{13}C NMR of compound **a2**

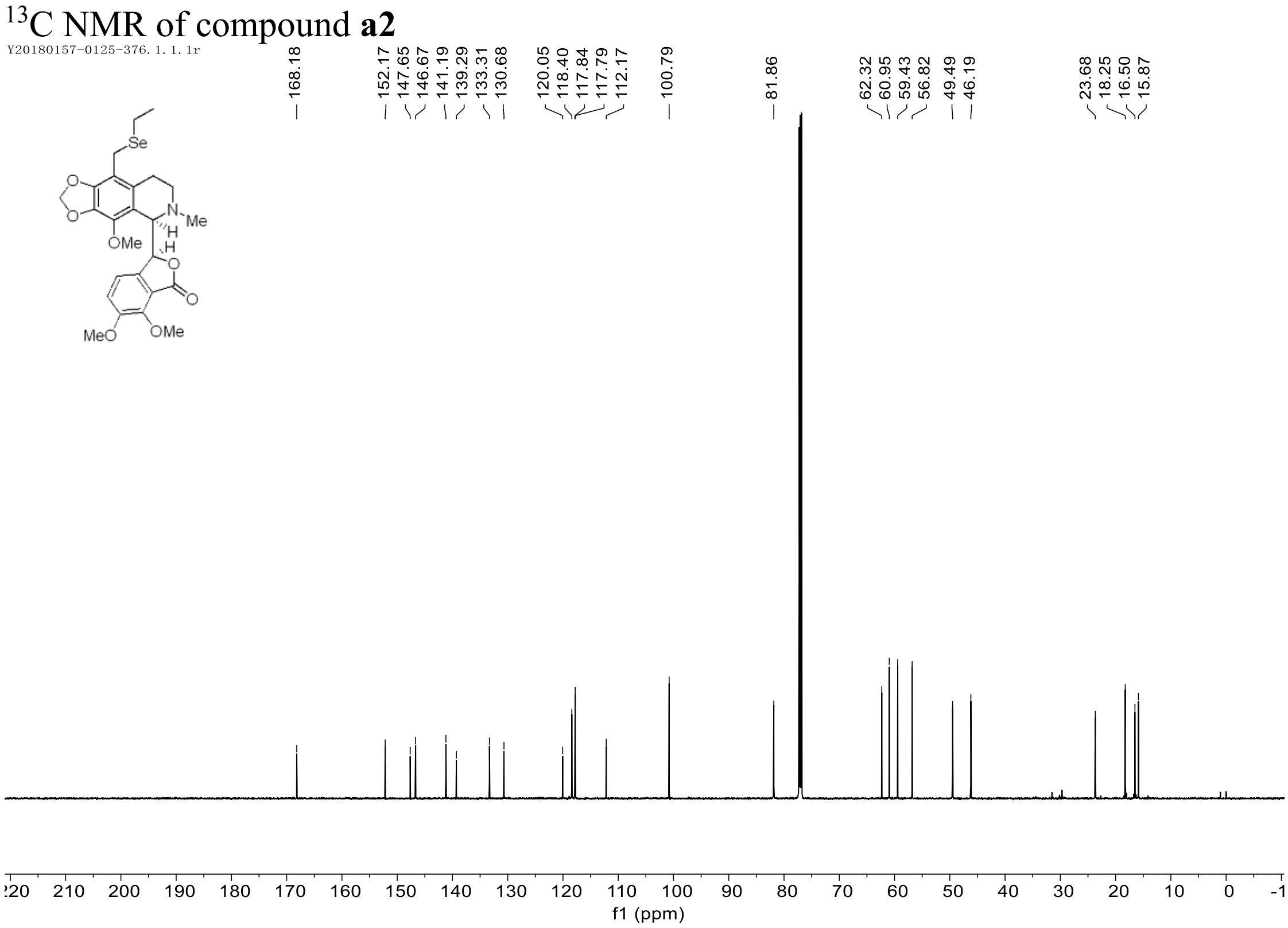

^{1}H NMR of compound **a3**

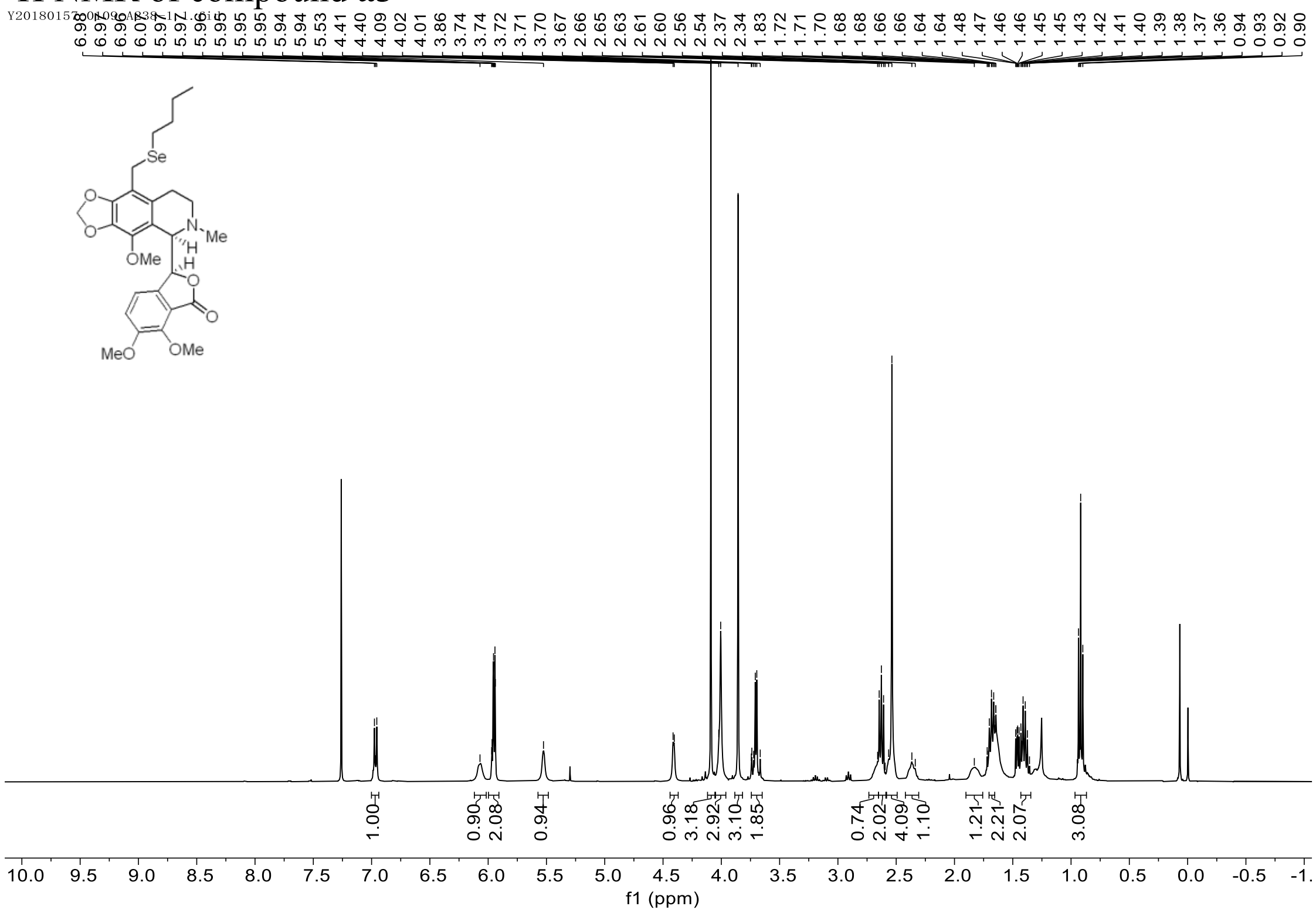

^{13}C NMR of compound **a3**

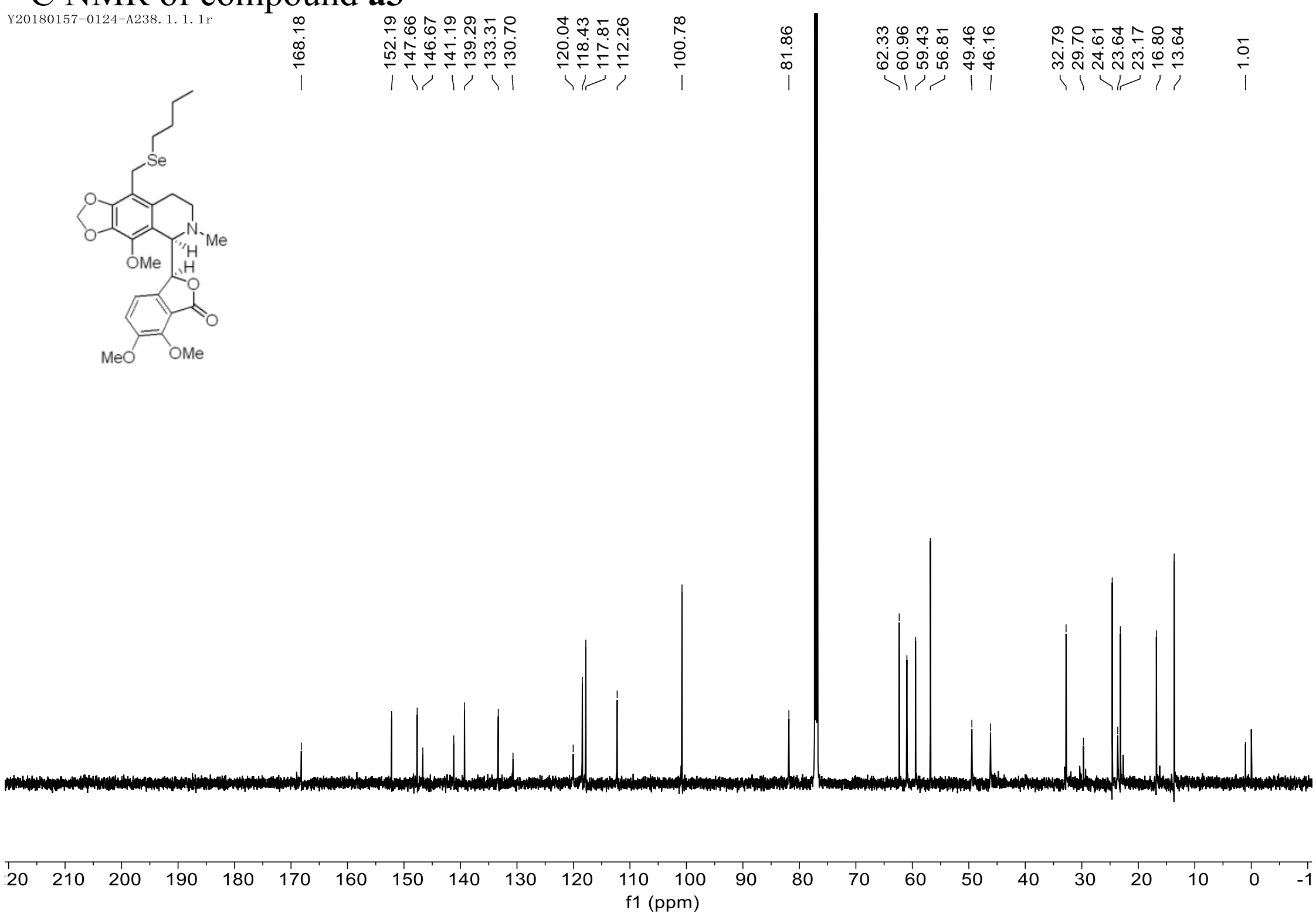

^{1}H NMR of compound **a4**

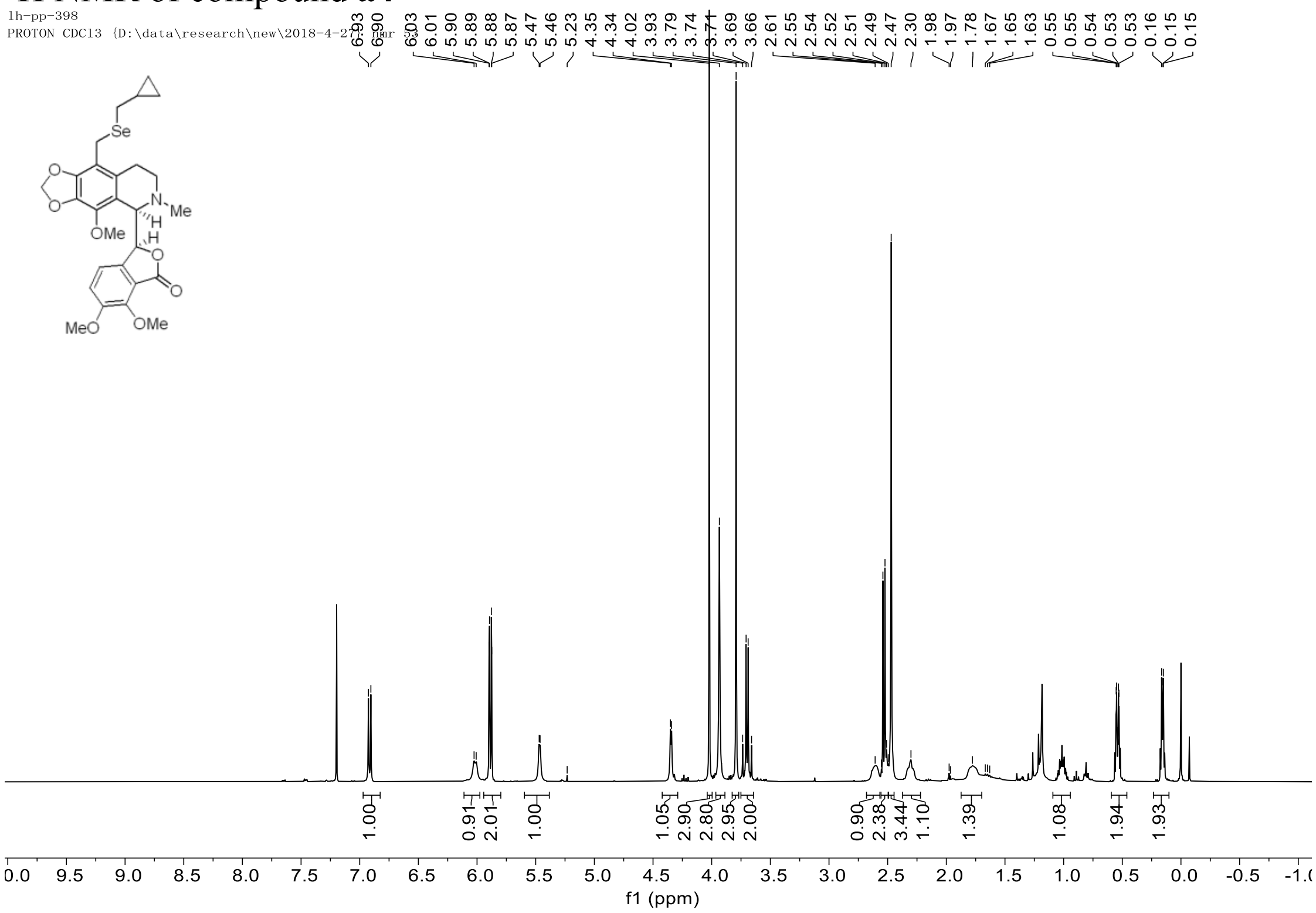

^{13}C NMR of compound **a4**

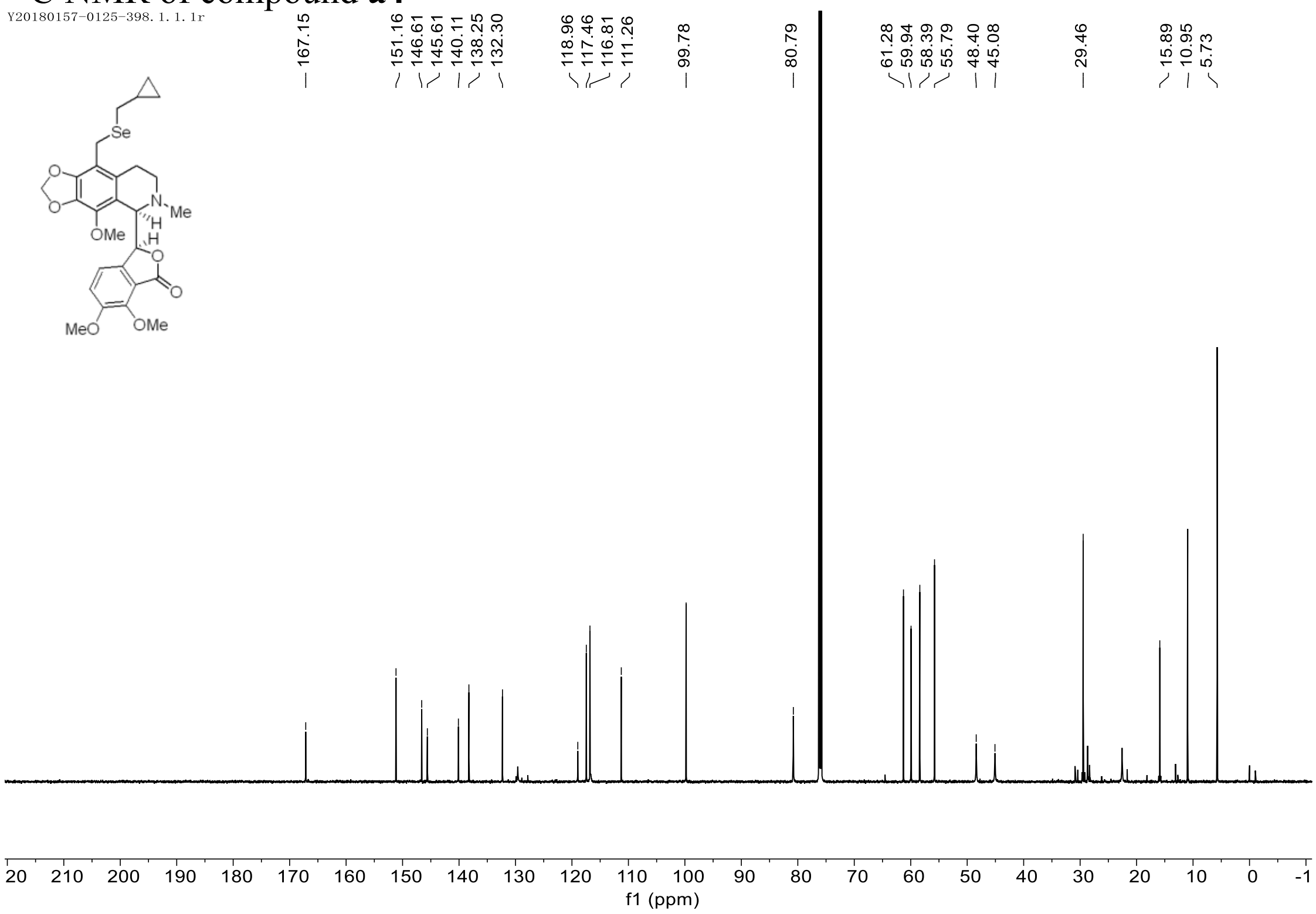

^{1}H NMR of compound **a5**

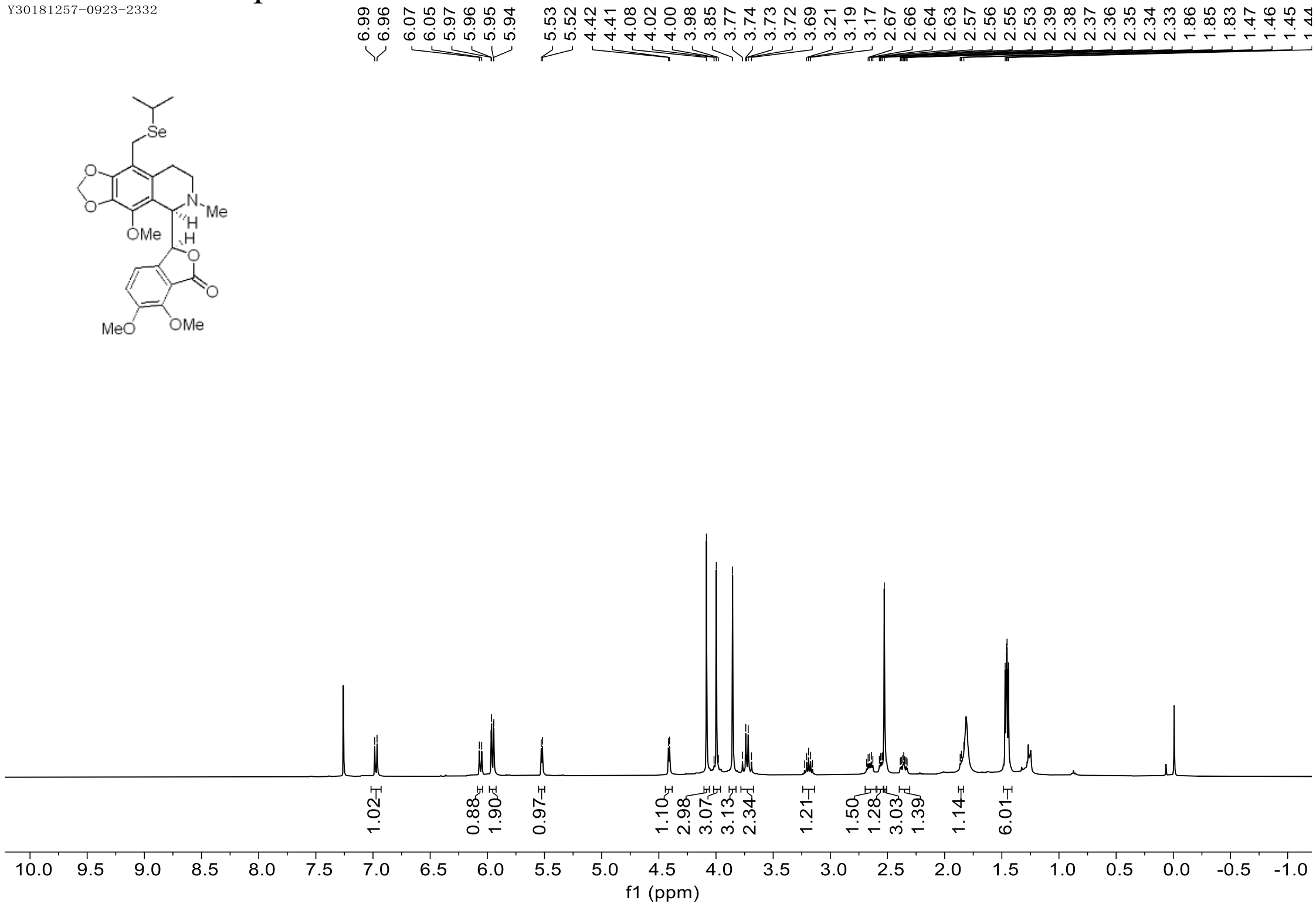

^{13}C NMR of compound **a5**

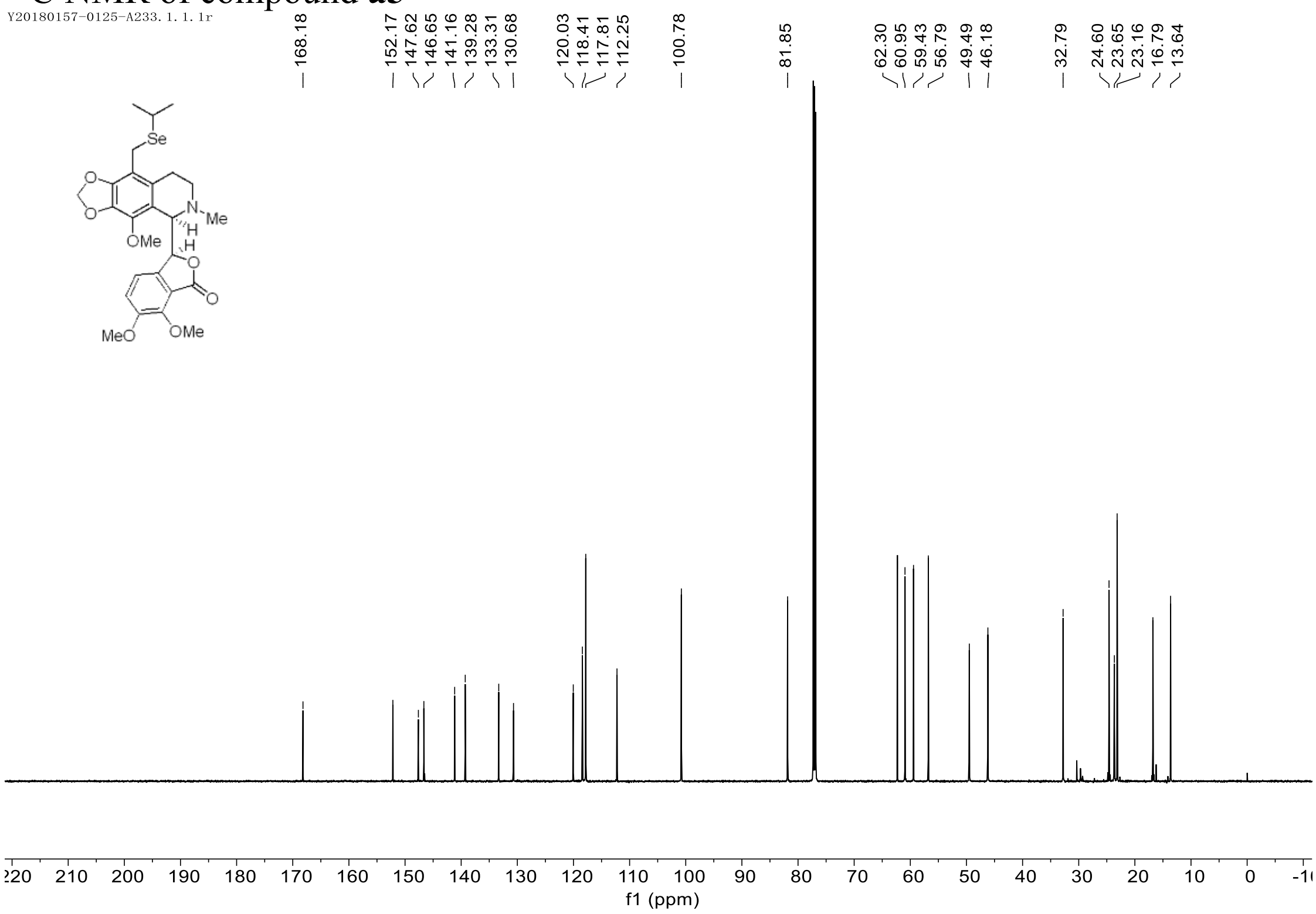

^{1}H NMR of compound **a6**

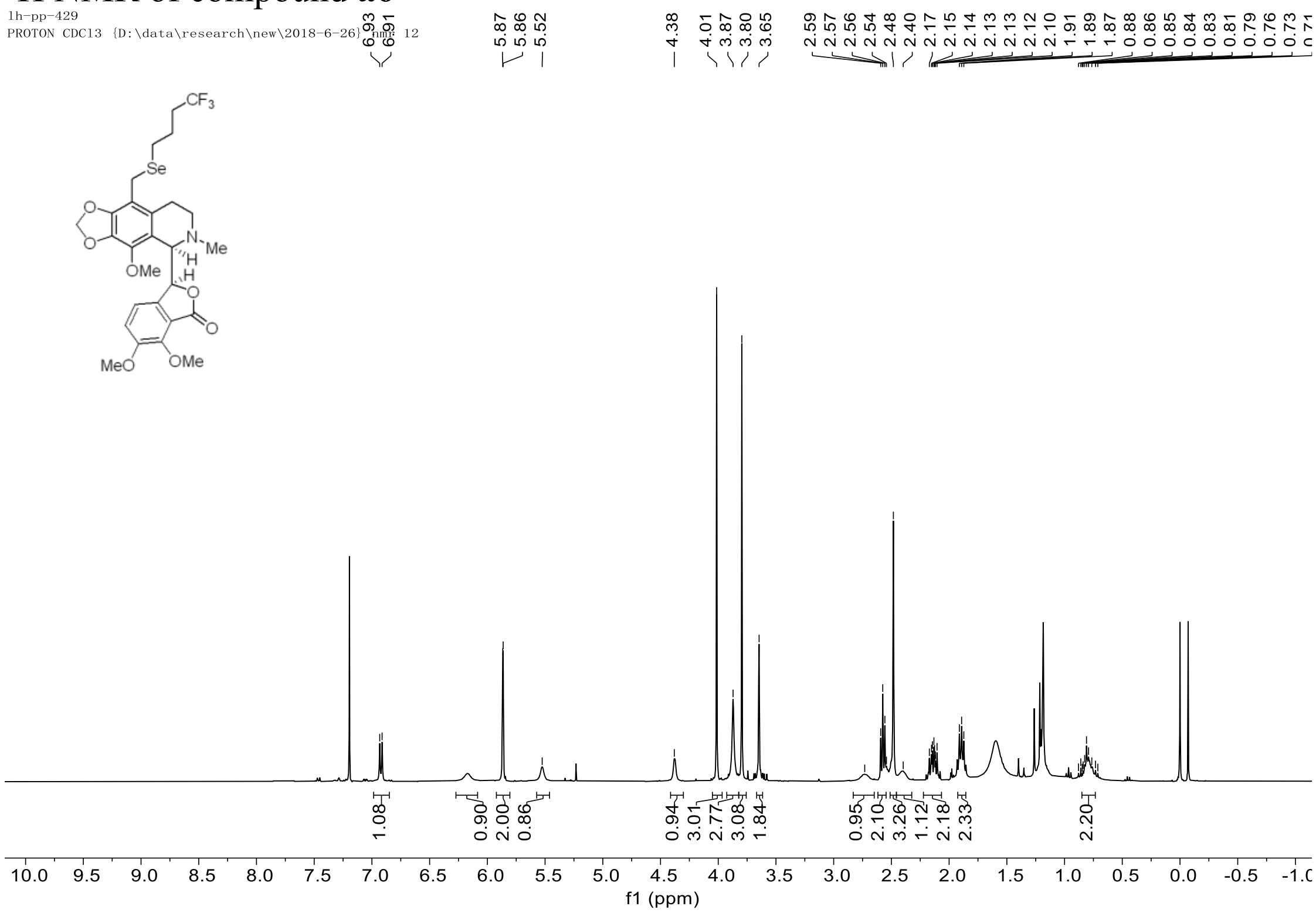

^{13}C NMR of compound **a6**

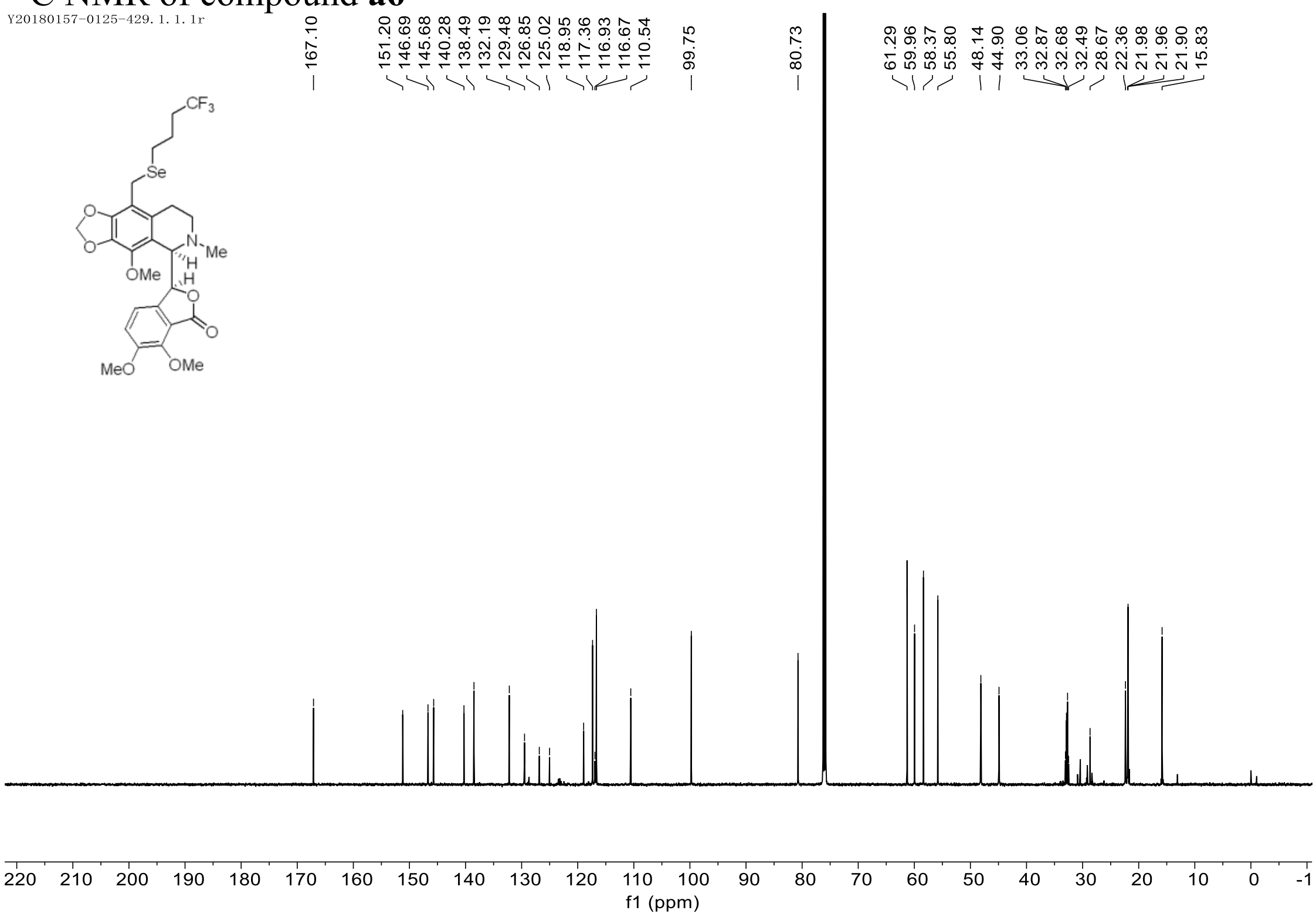

^{1}H NMR of compound **a7**

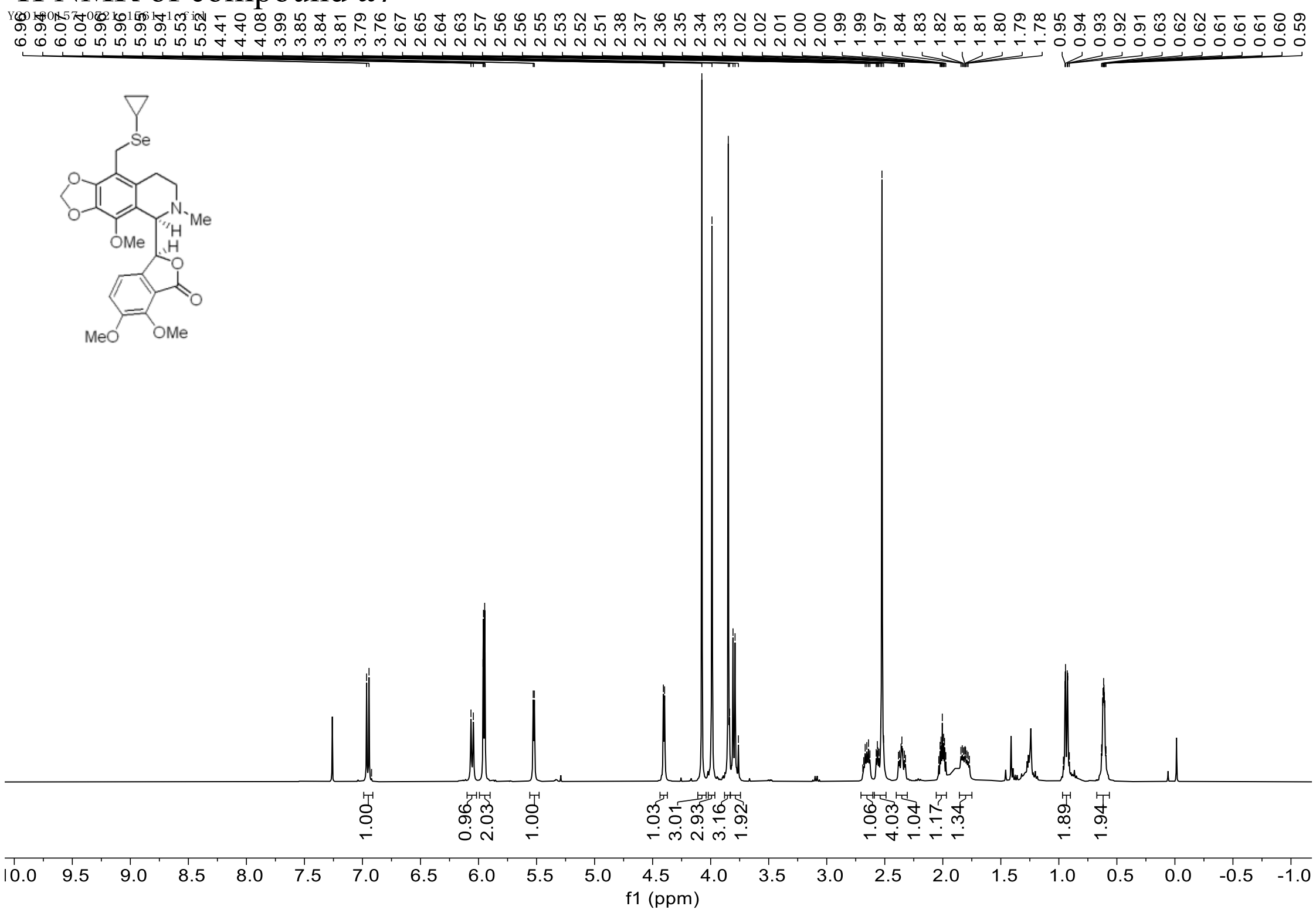

^{13}C NMR of compound **a7**

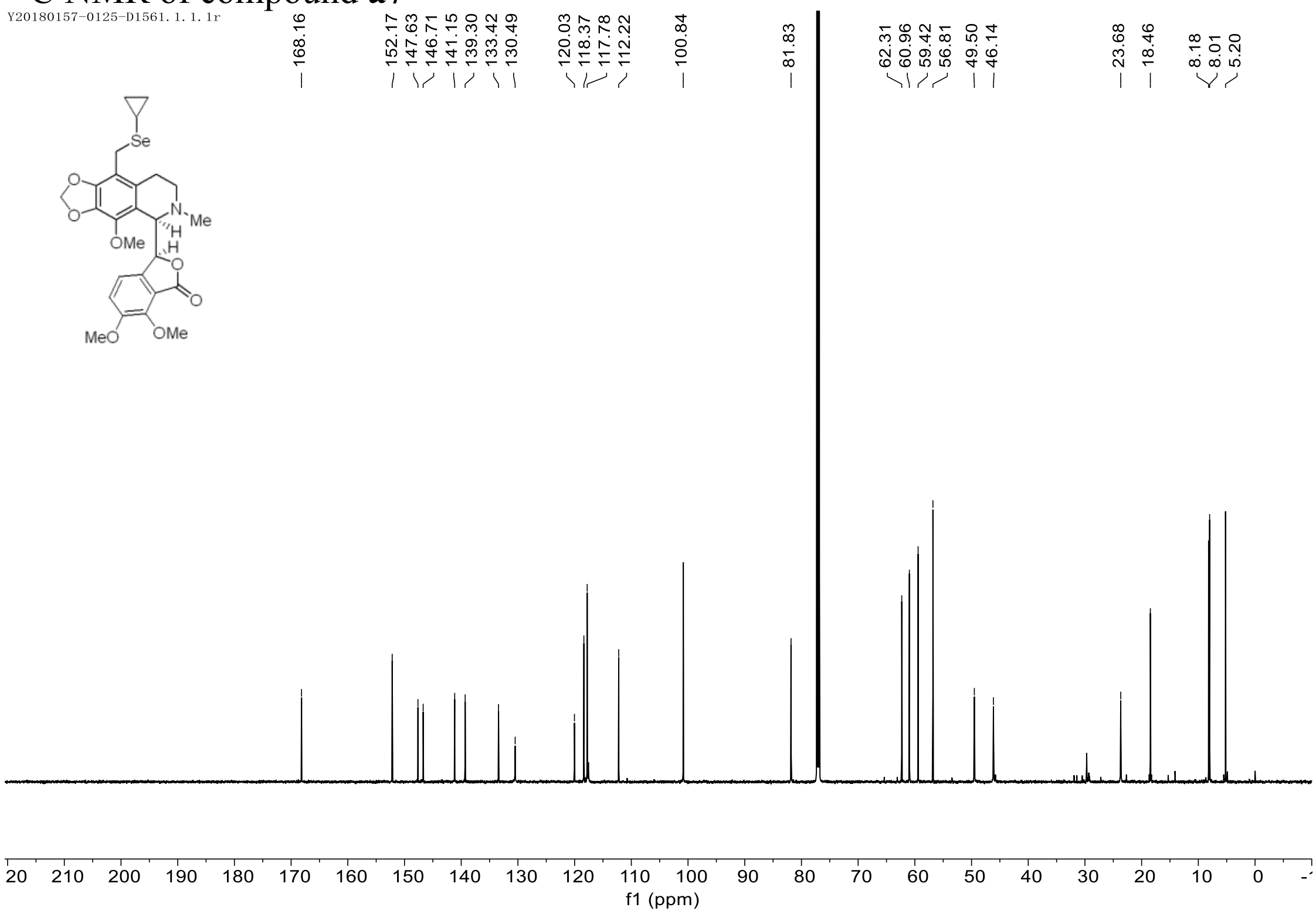

^{1}H NMR of compound **a8**

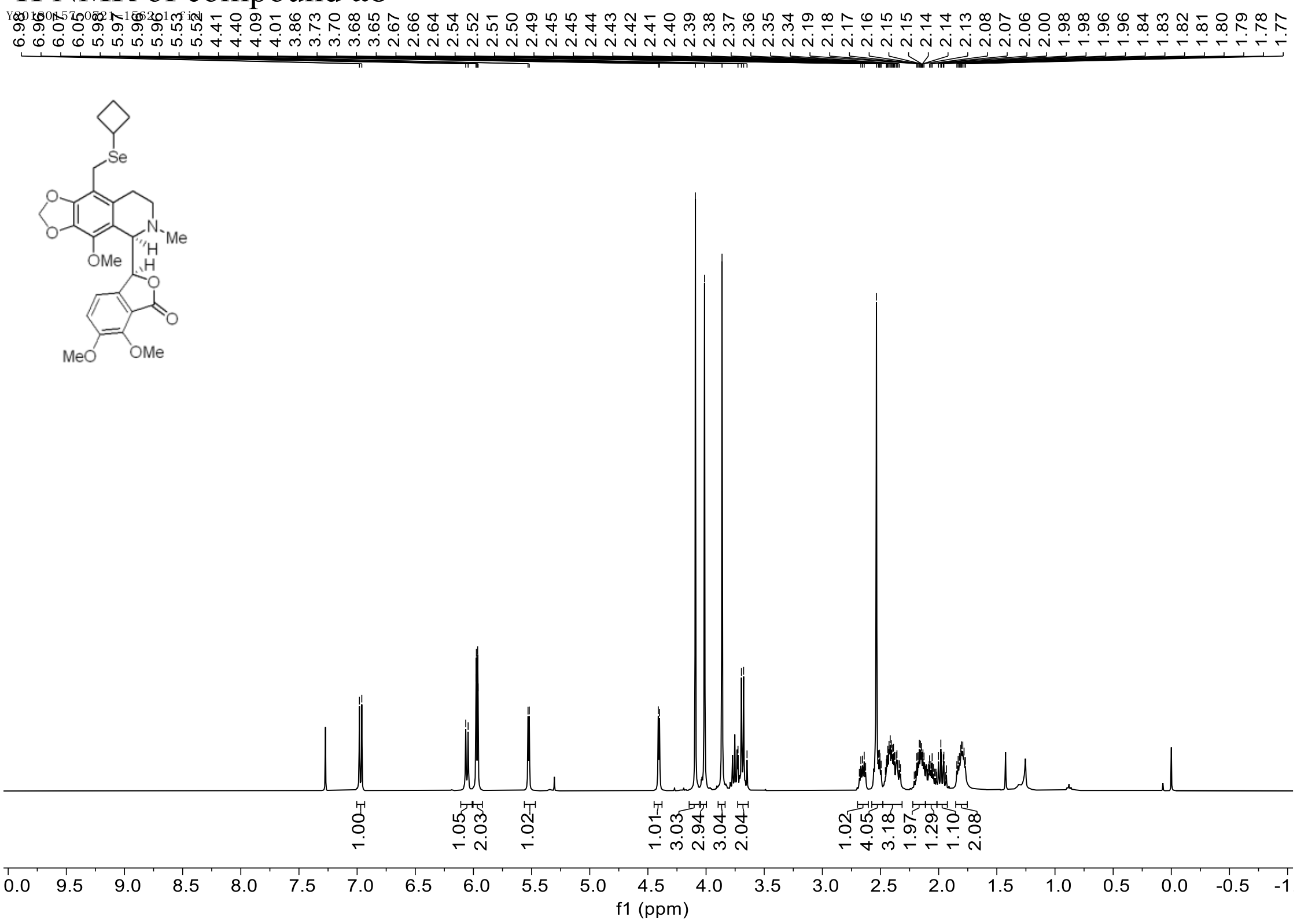

^{13}C NMR of compound **a8**

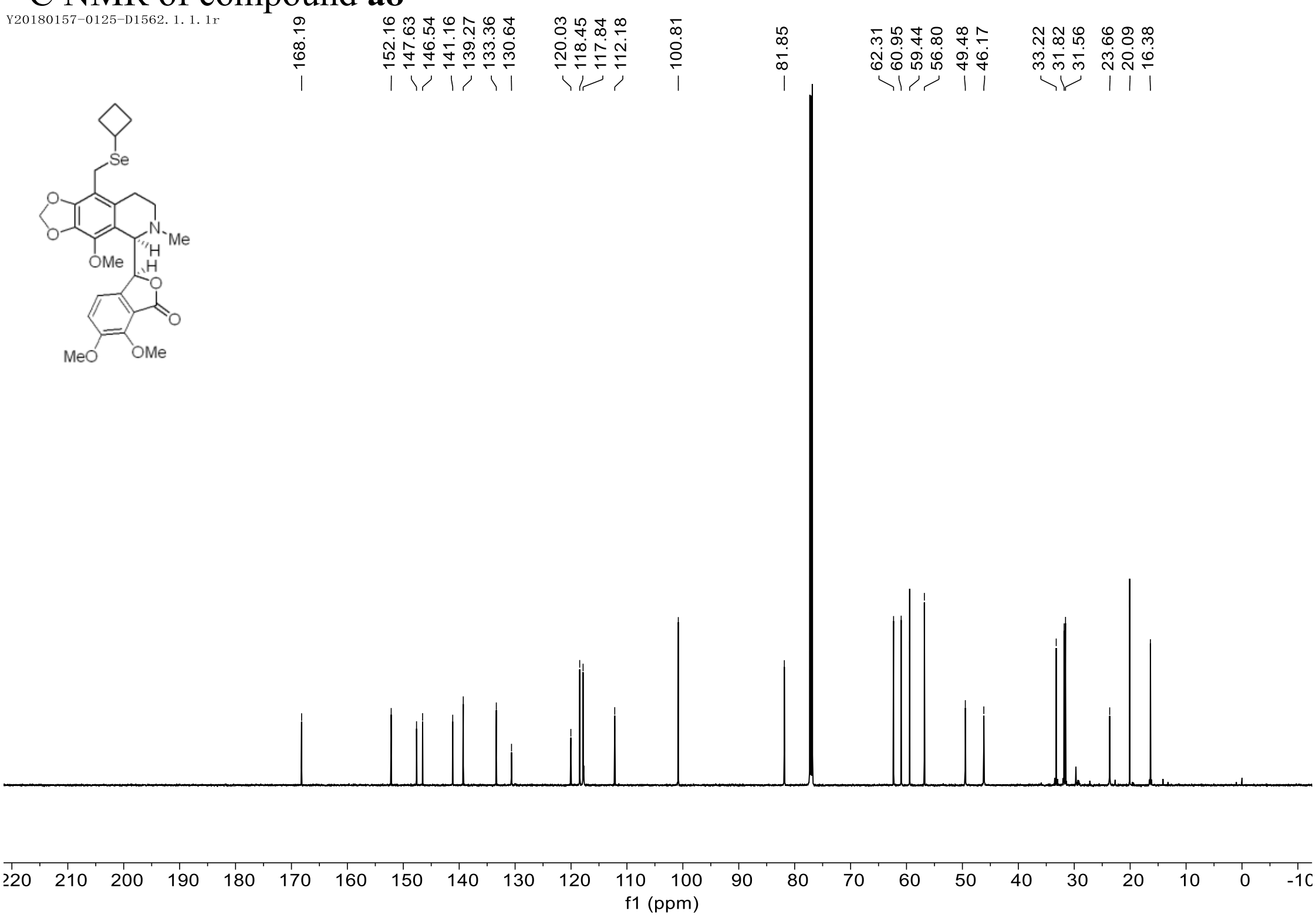

^{1}H NMR of compound **a9**

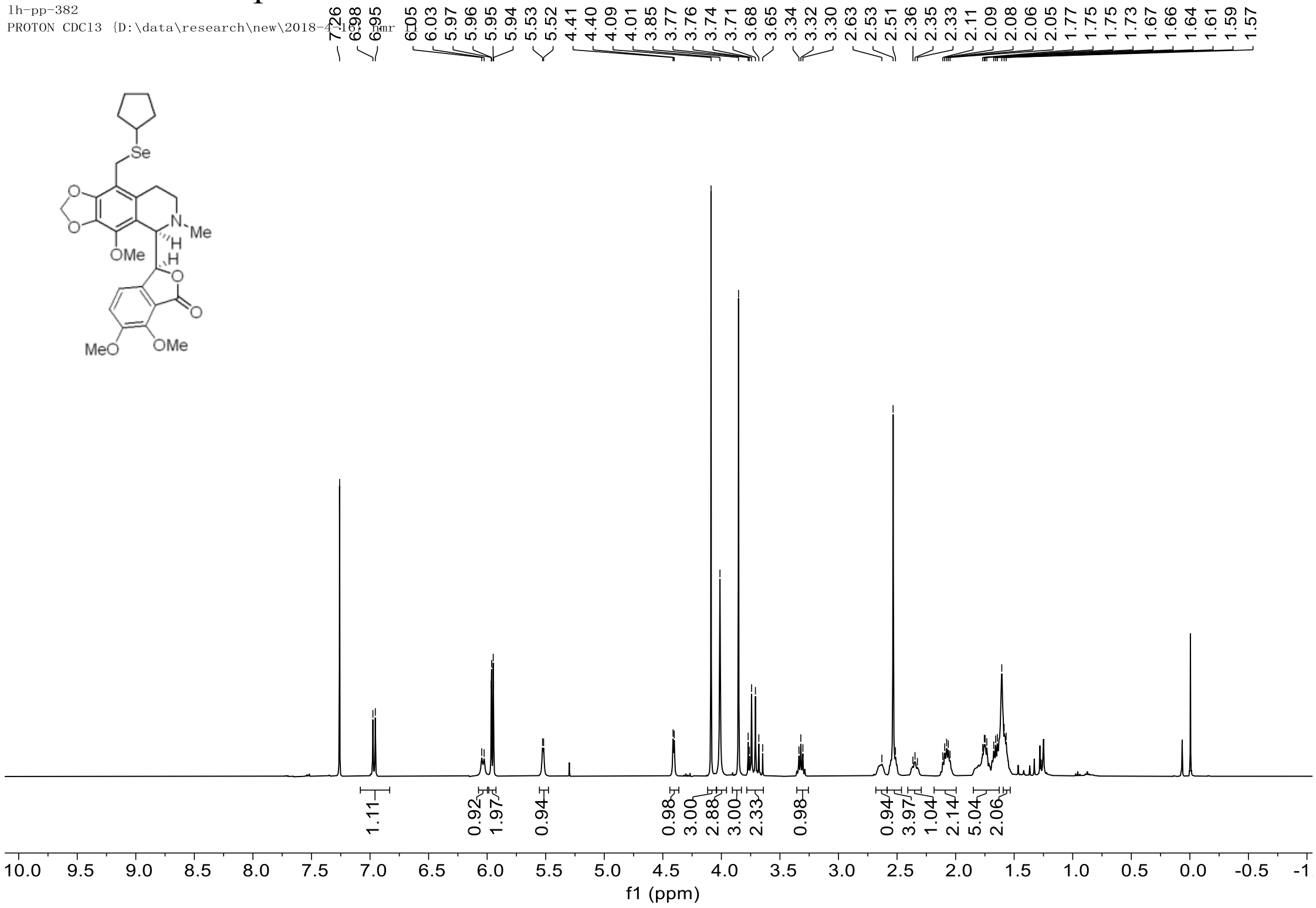

^{13}C NMR of compound **a9**

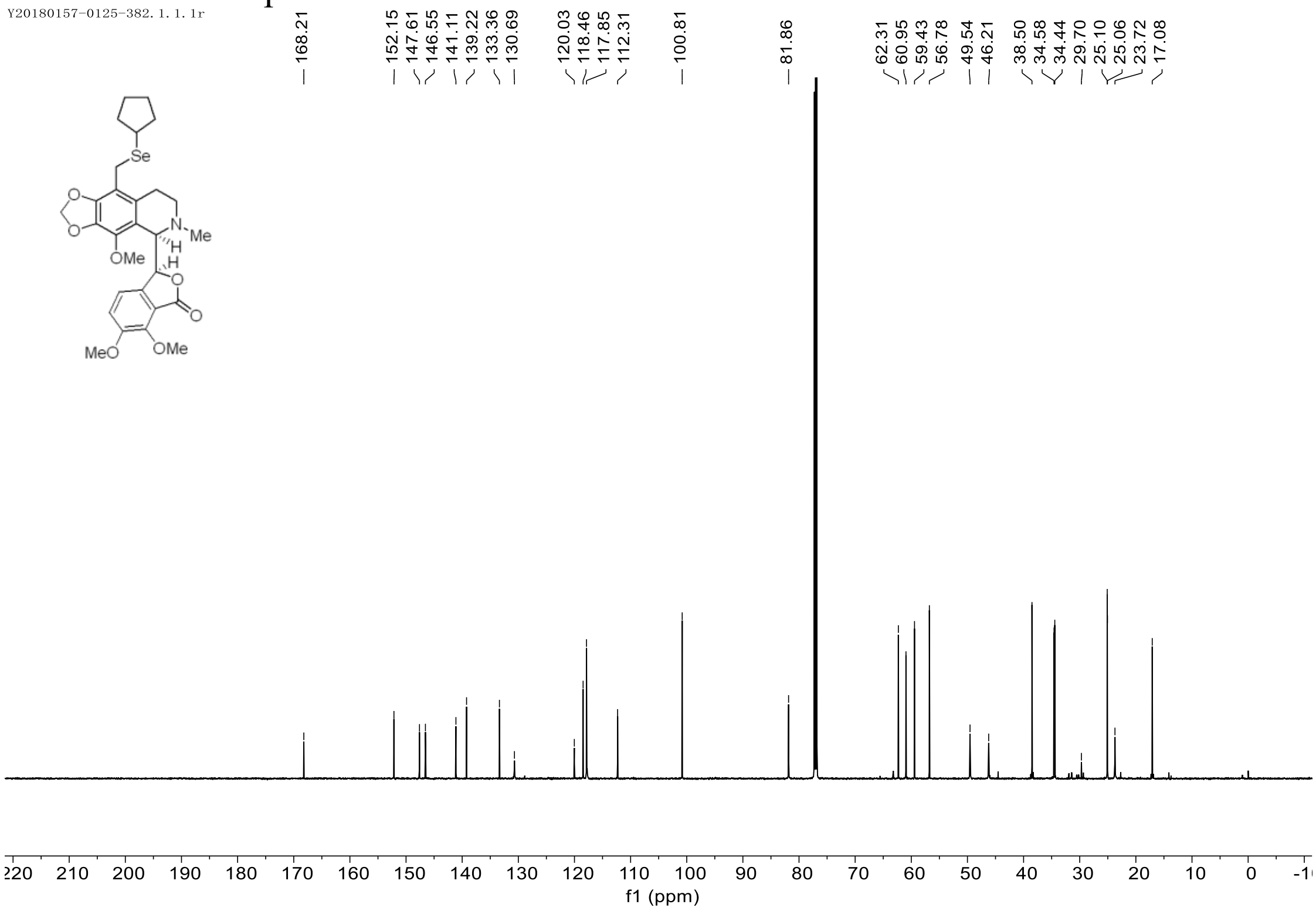

^{1}H NMR of compound **a10**

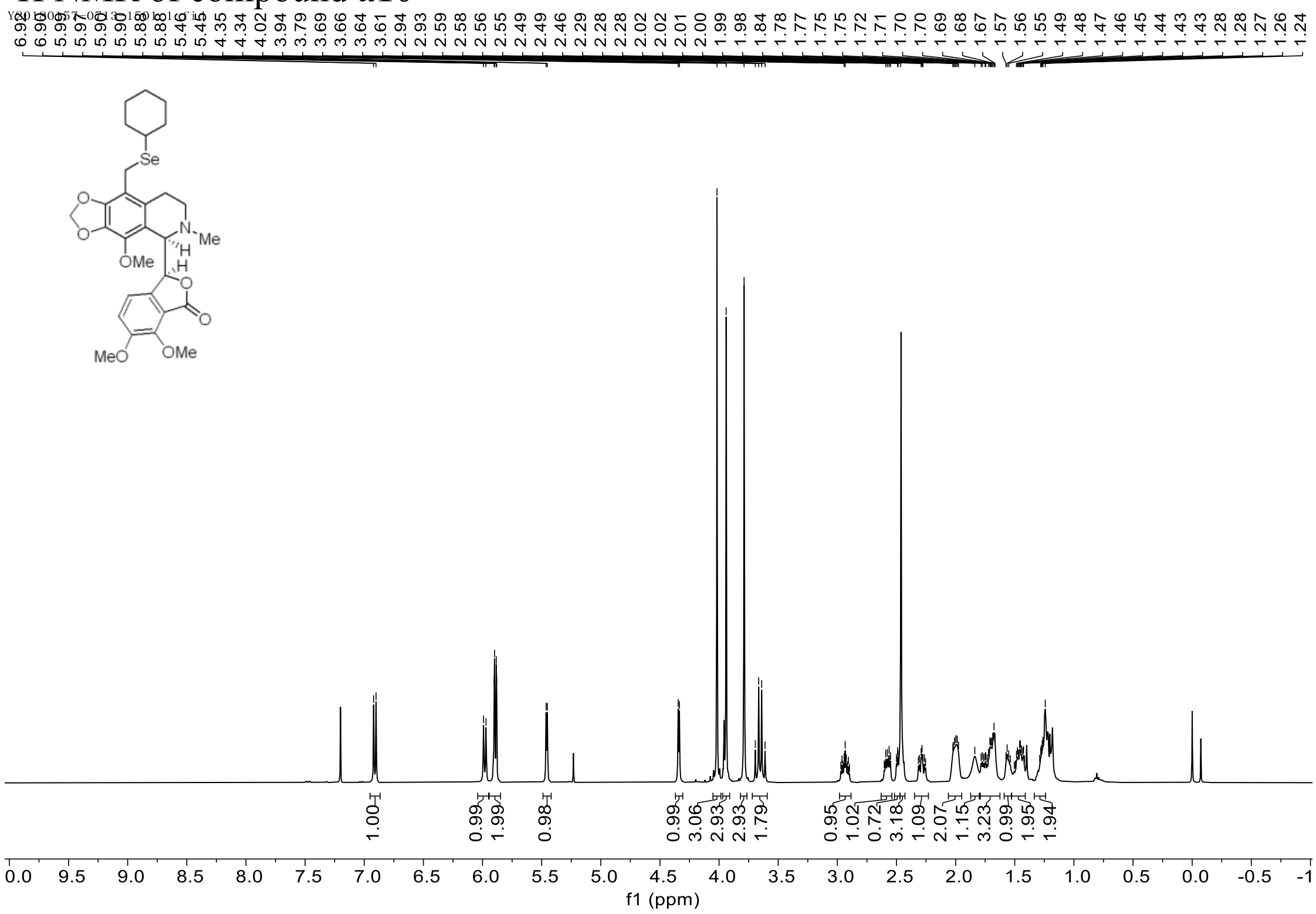

^{13}C NMR of compound **a10**

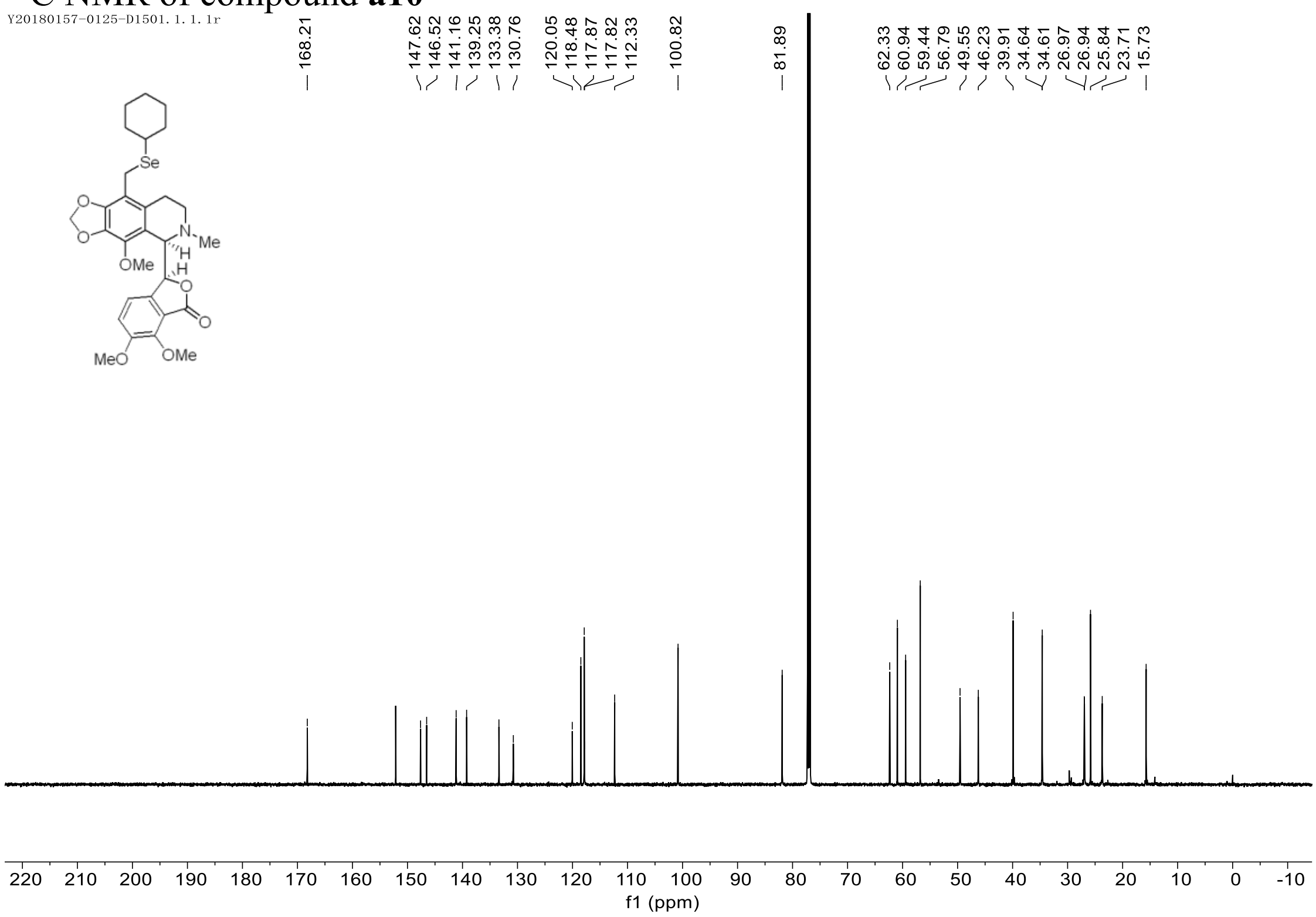

^{1}H NMR of compound **ES428**

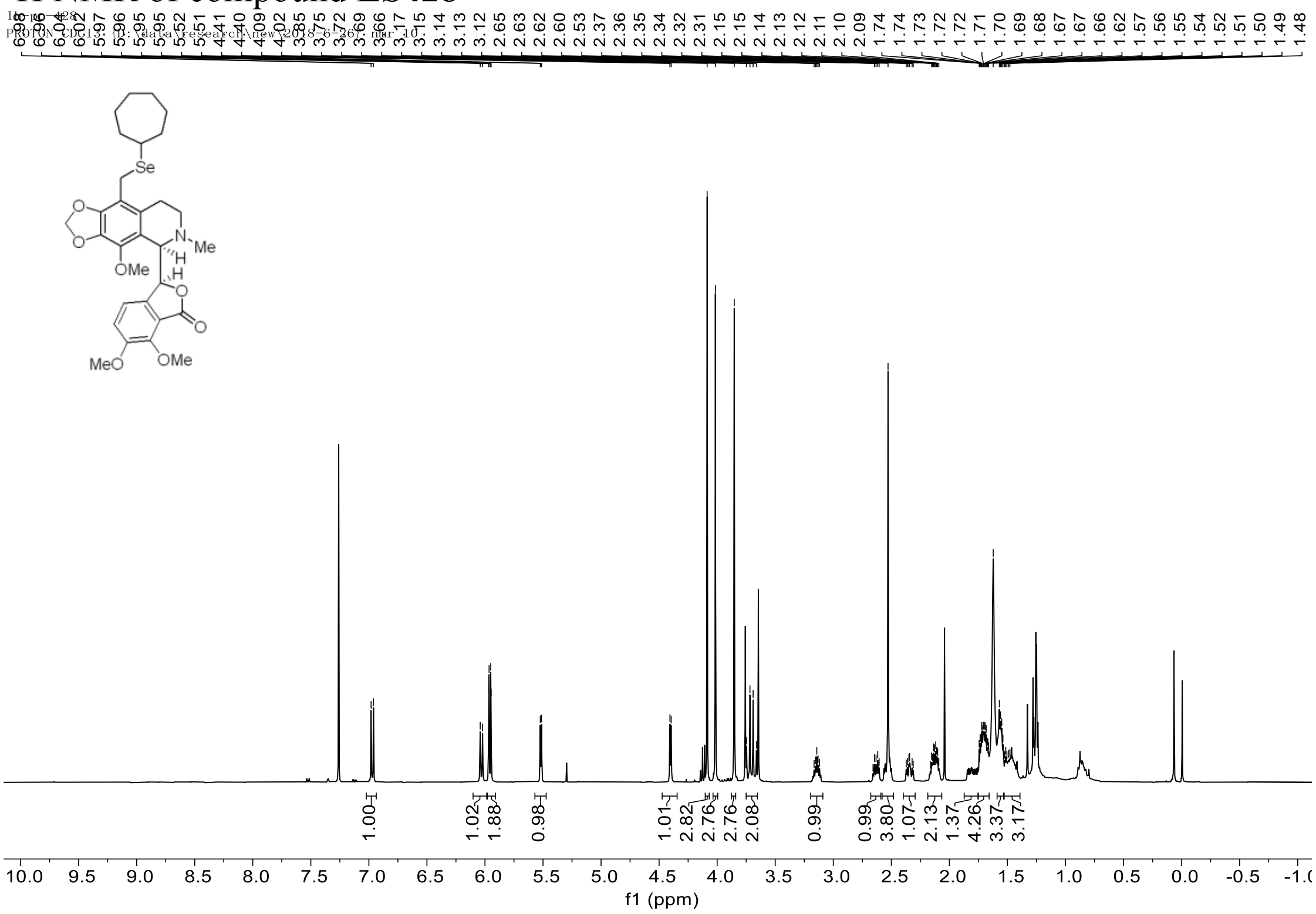

^{13}C NMR of compound **ES428**

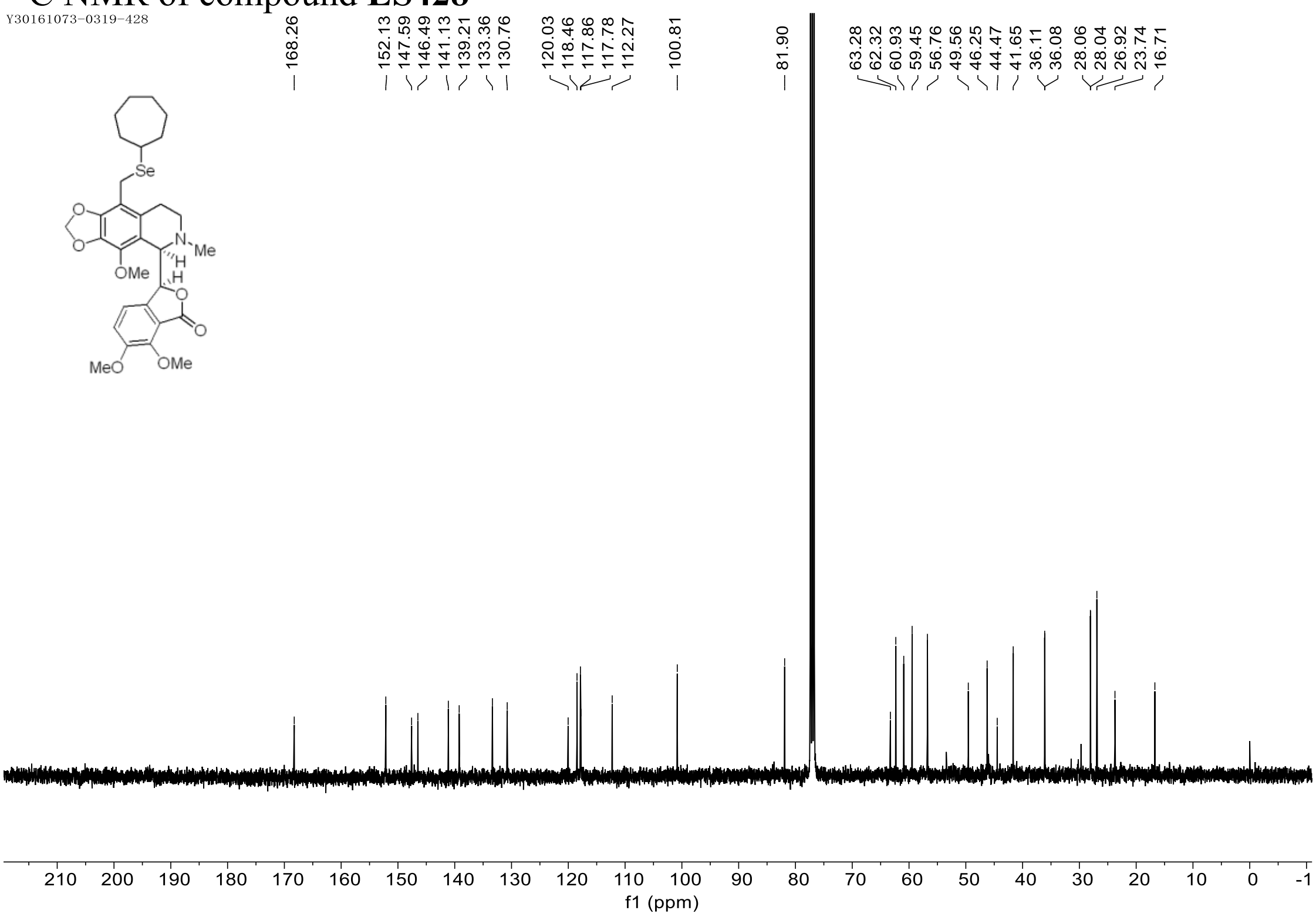

^{1}H NMR of compound **a11**

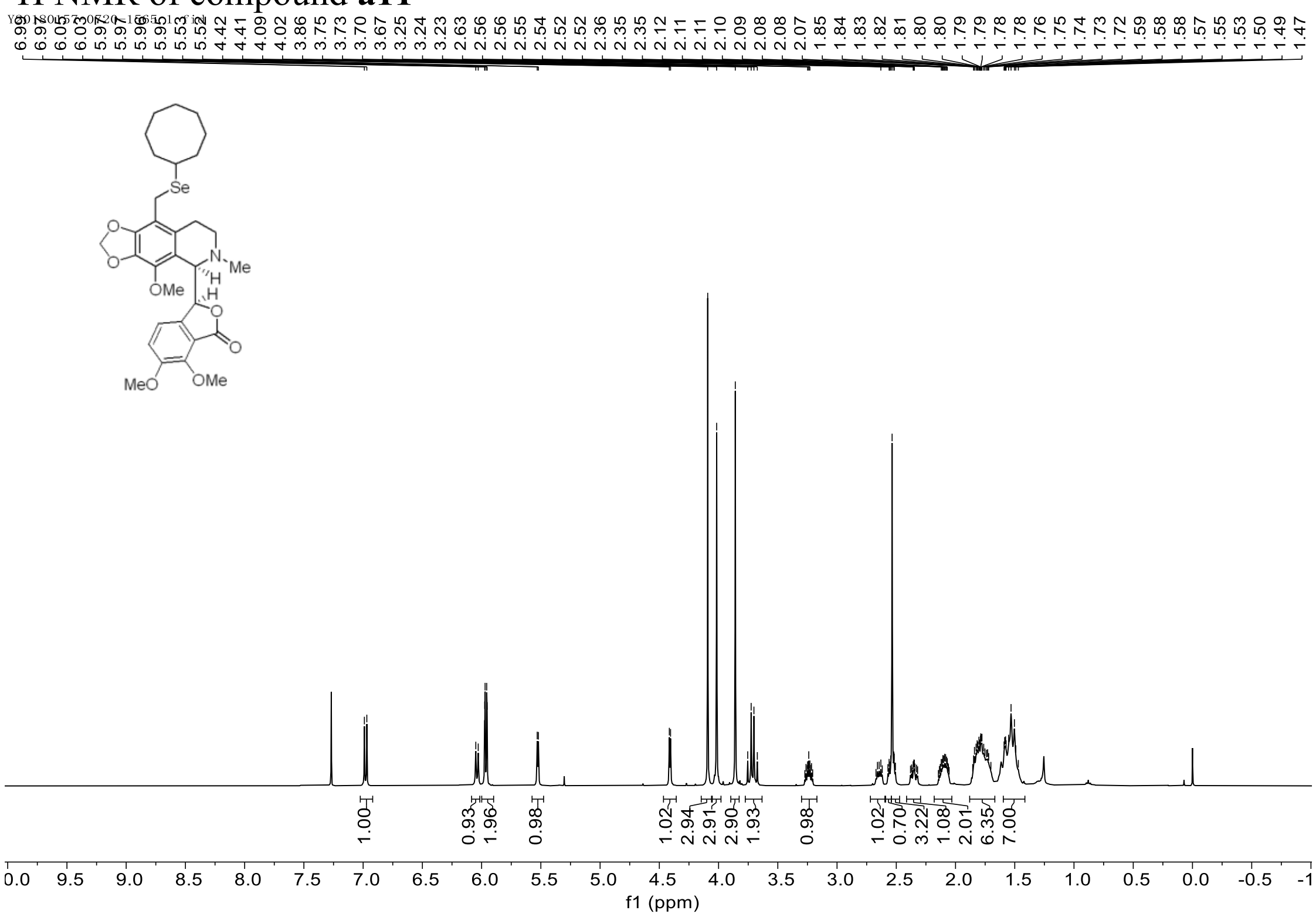

^{13}C NMR of compound **a11**

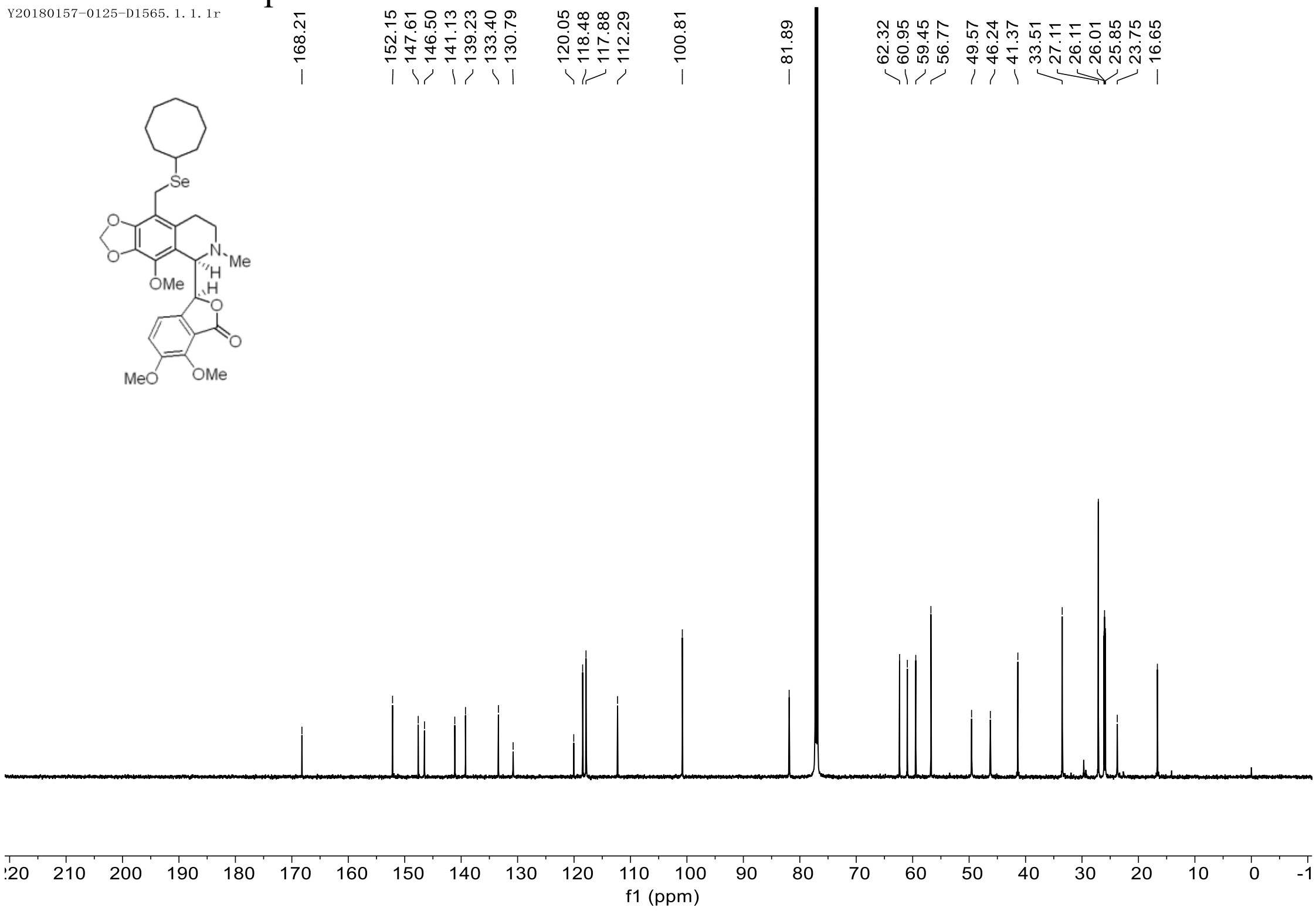

^{1}H NMR of compound **a12**

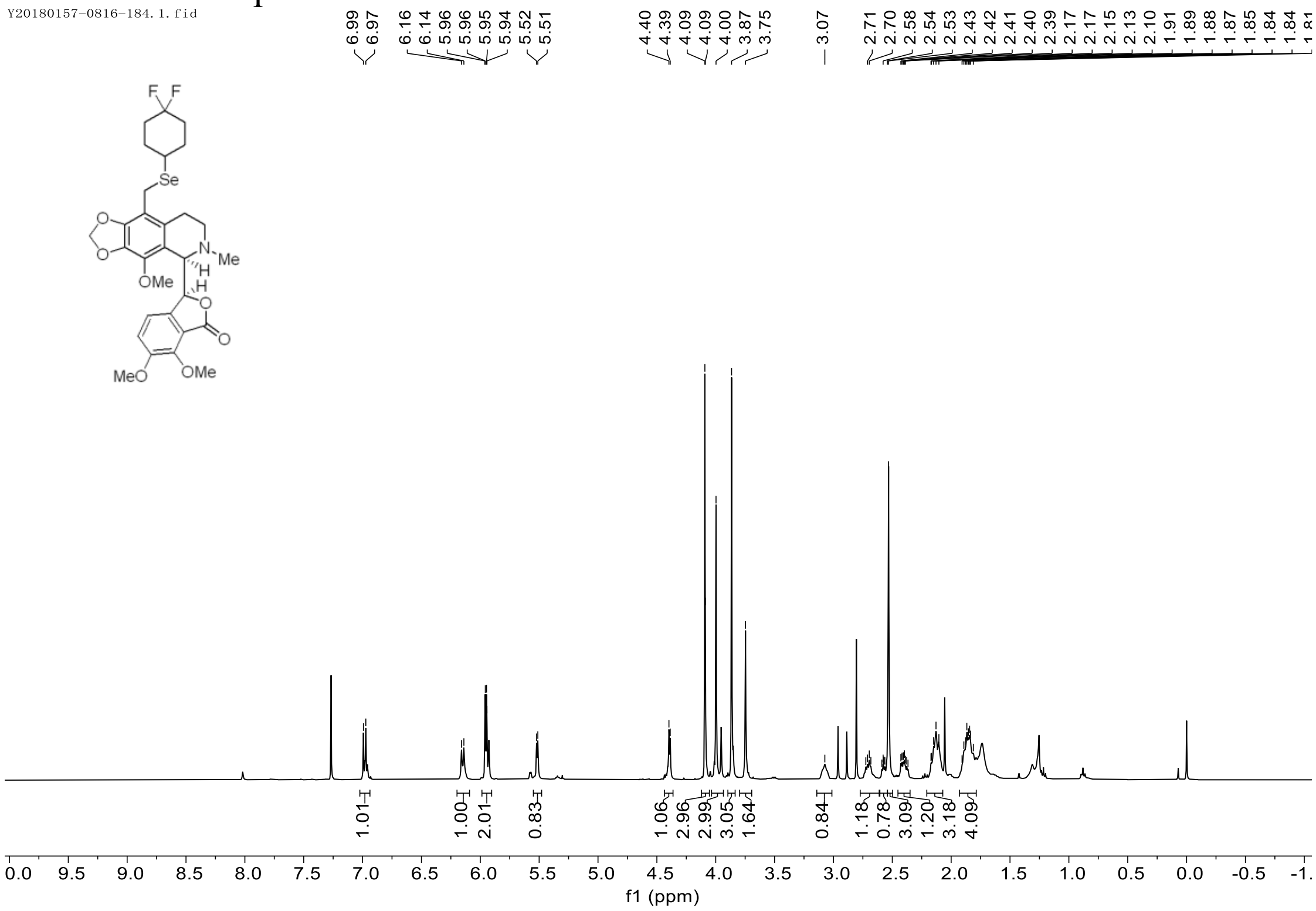

^{13}C NMR of compound **a12**

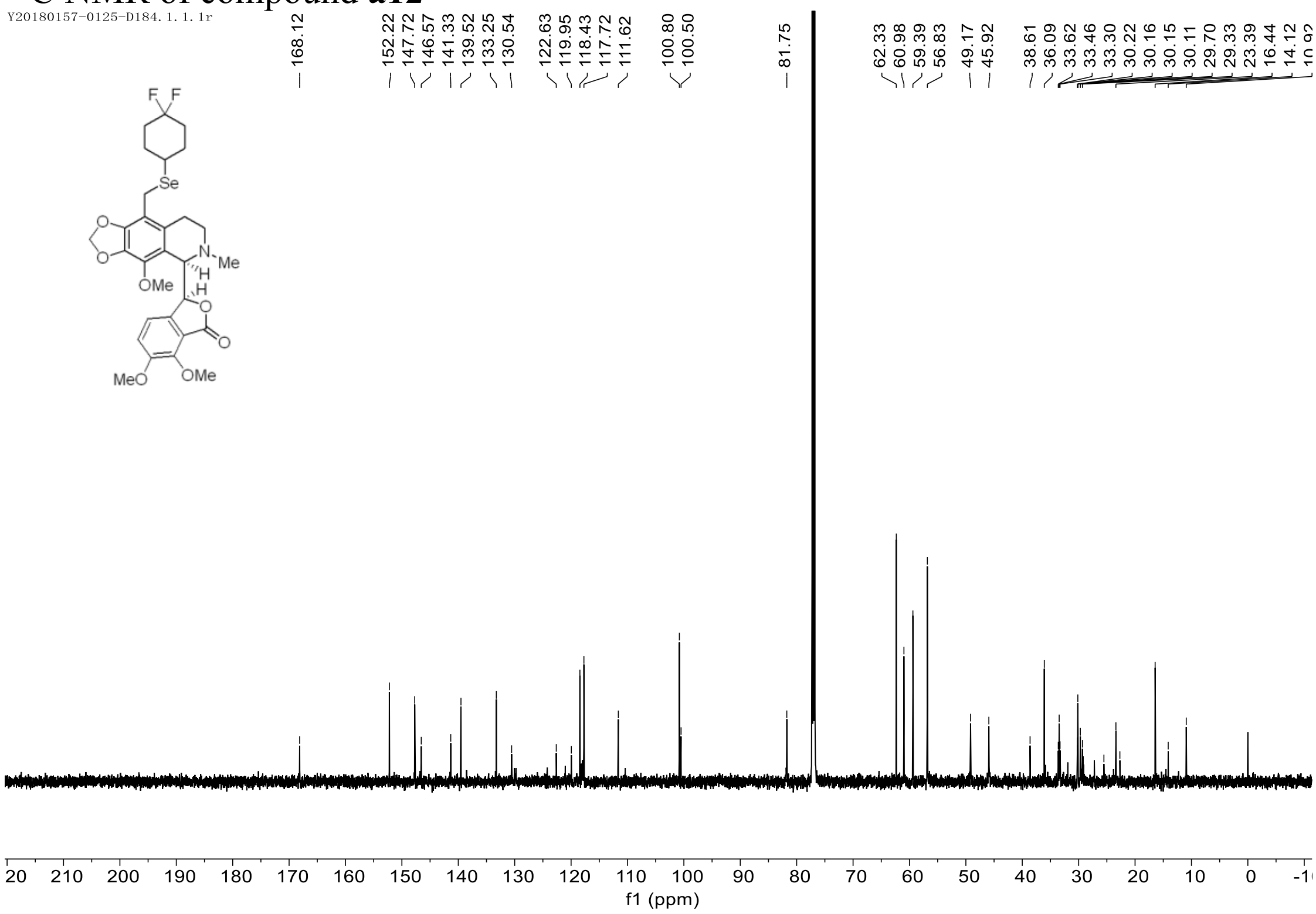

^{1}H NMR of compound **a13**

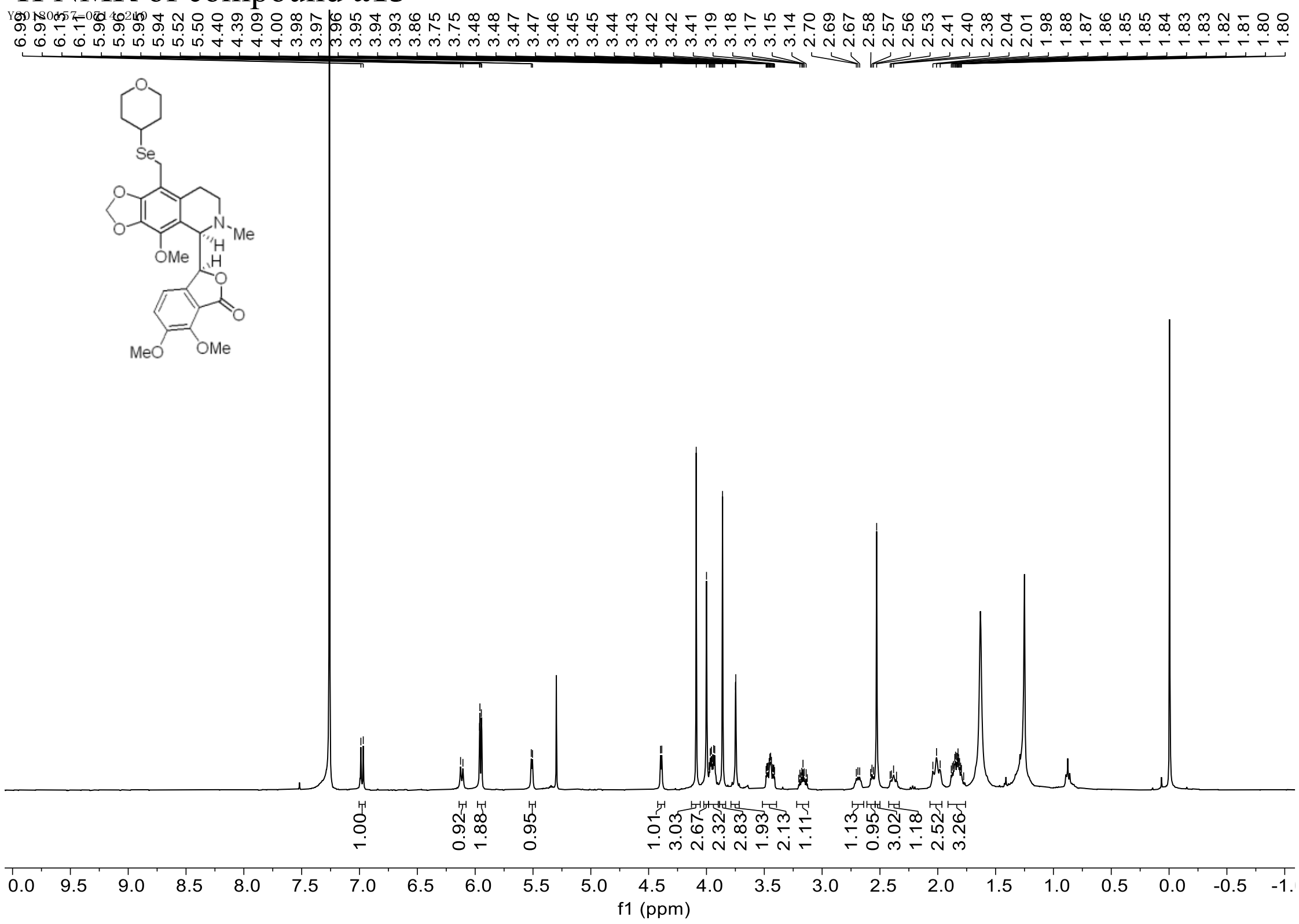

^{1}H NMR of compound **9**

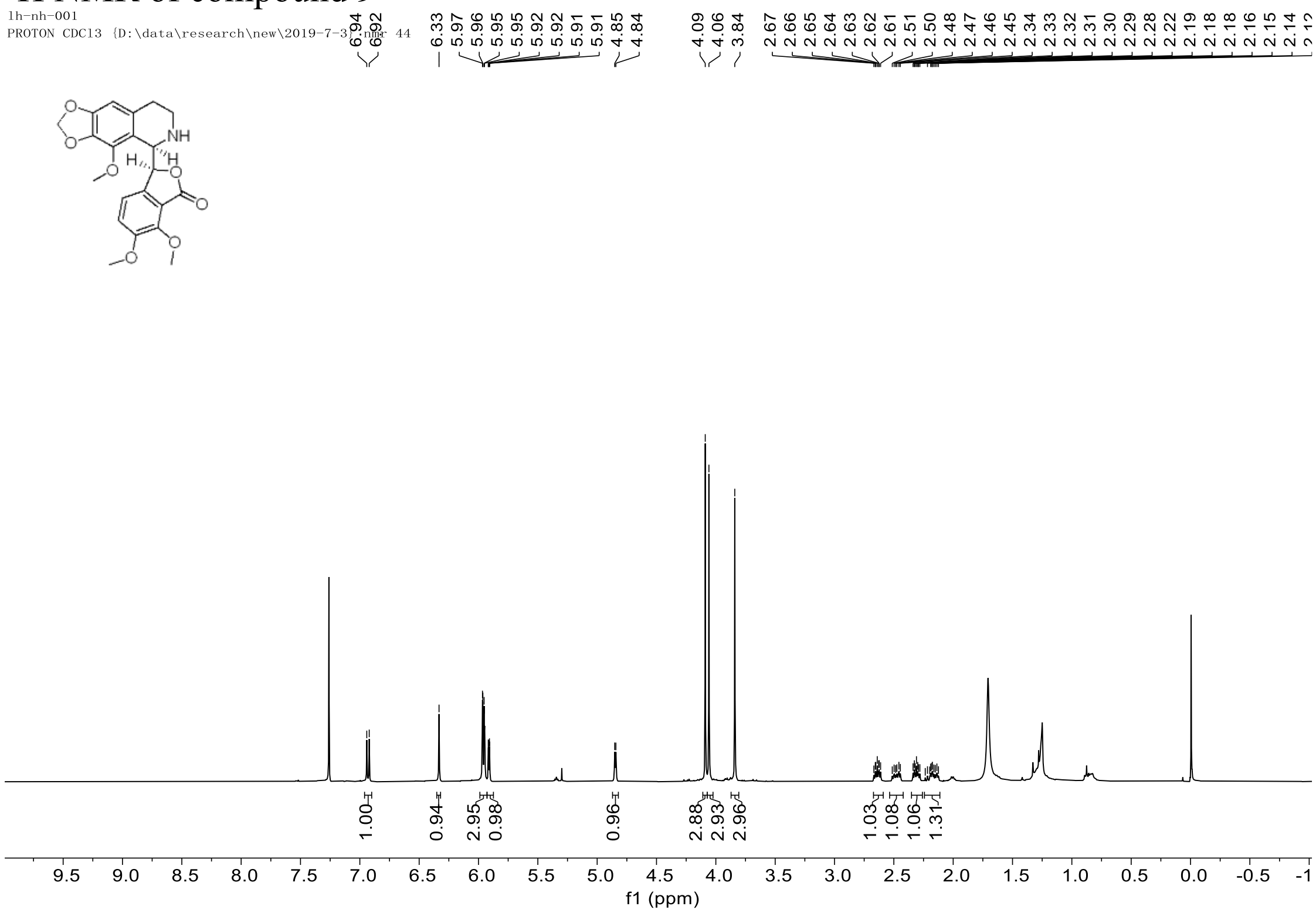

^{1}H NMR of compound **15**

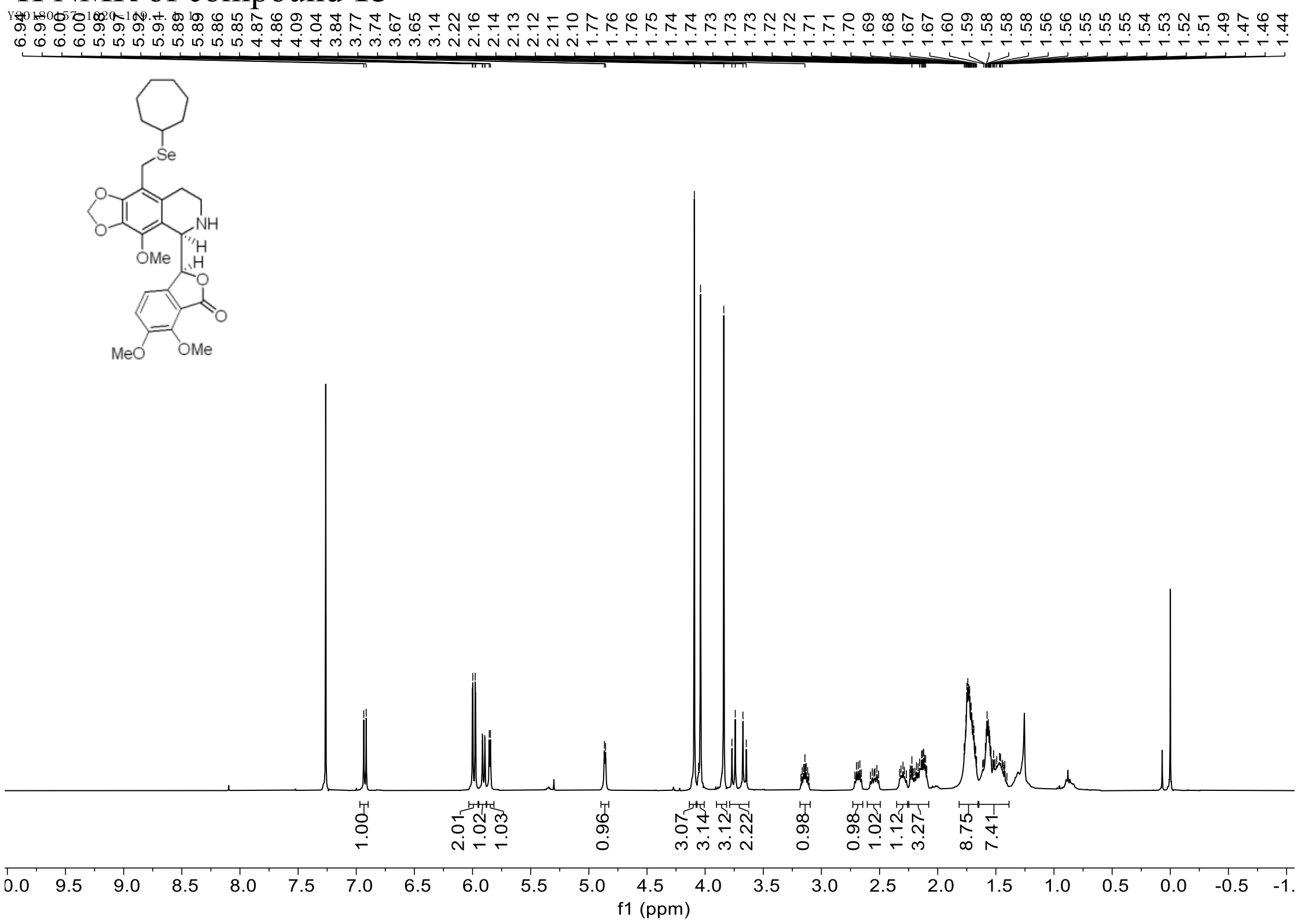

^{13}C NMR of compound **15**

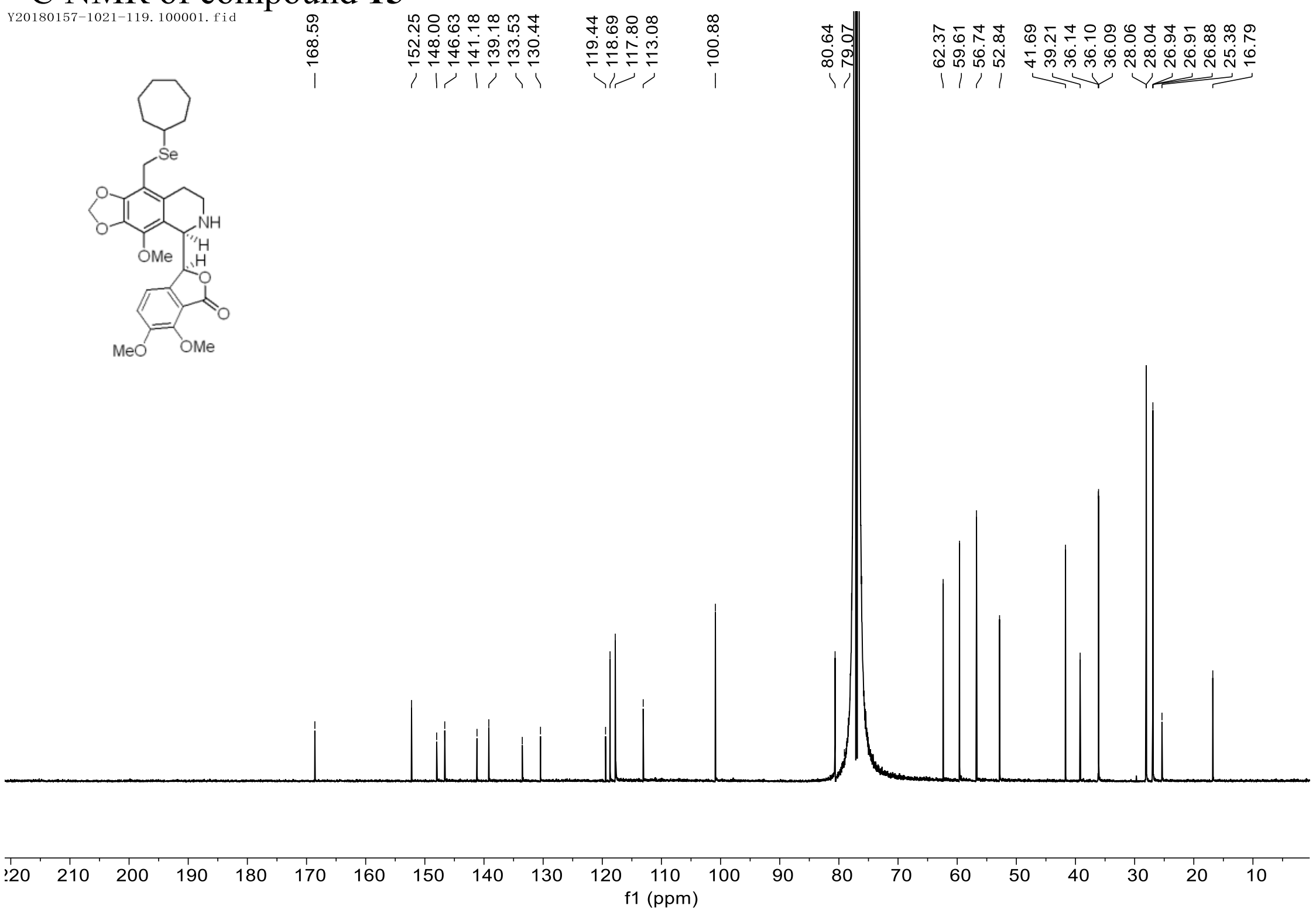

^{1}H NMR of compound **a14**

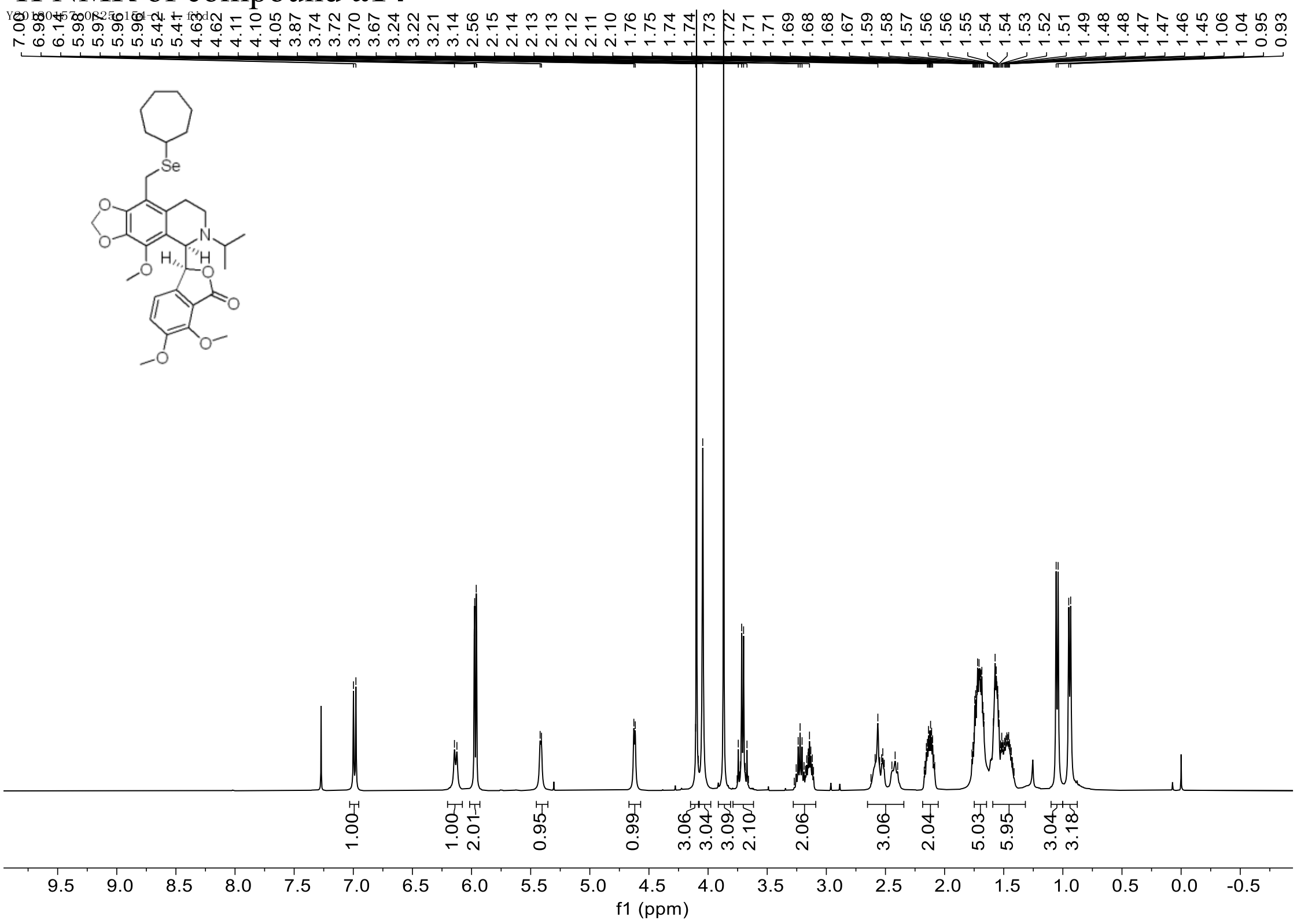

^{1}H NMR of compound **a15**

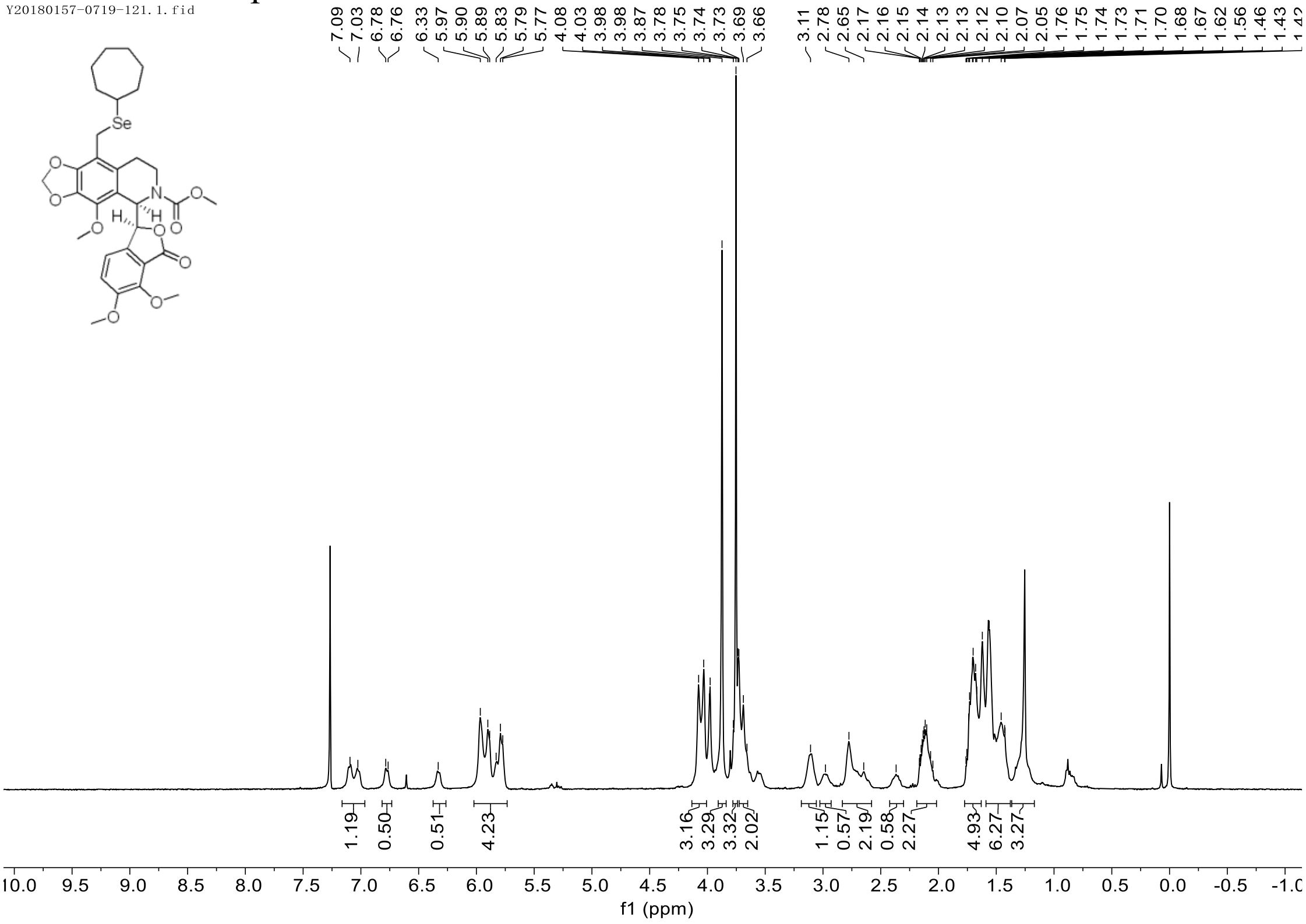

^{1}H NMR of compound **a16**

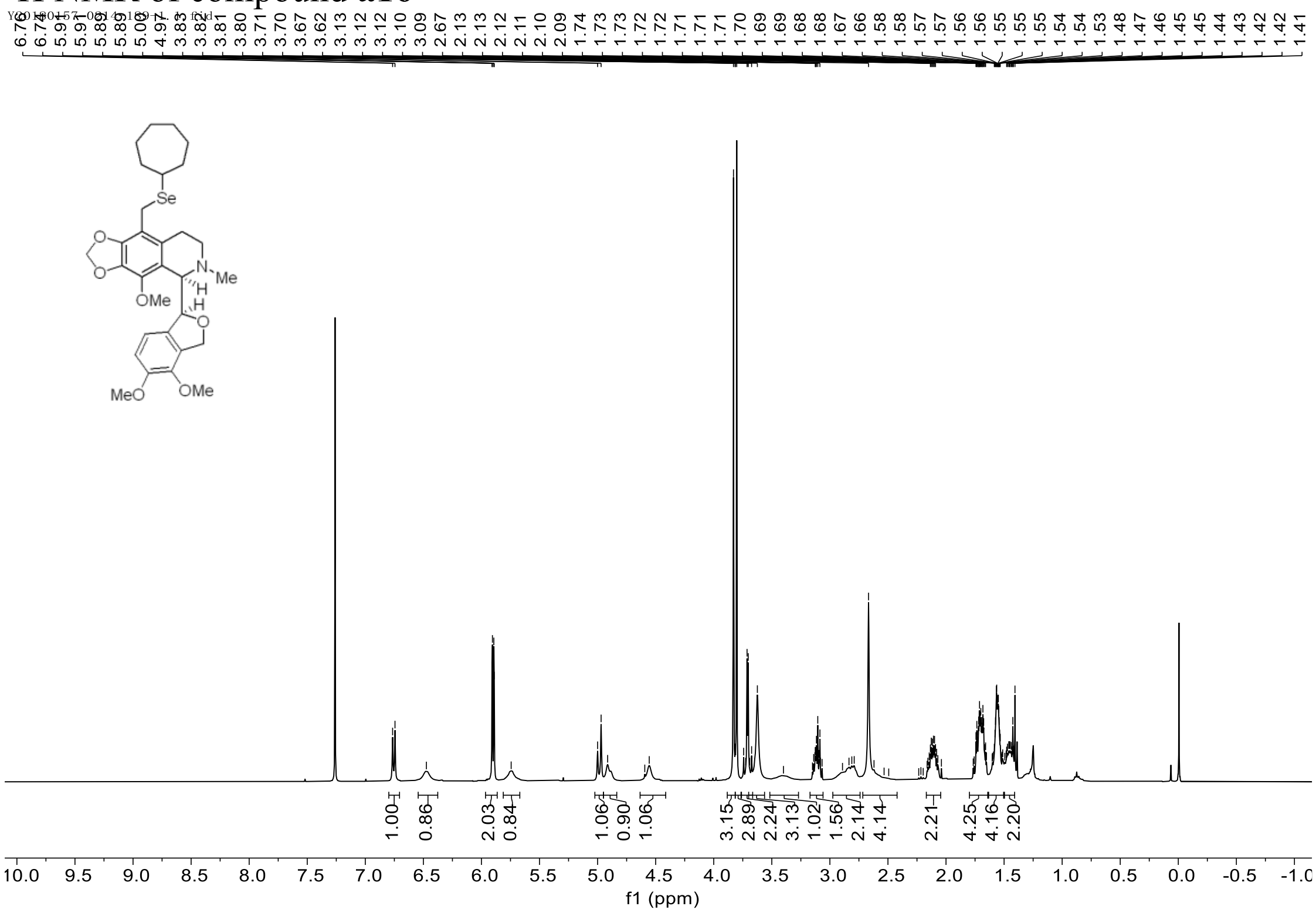

^{13}C NMR of compound **a16**

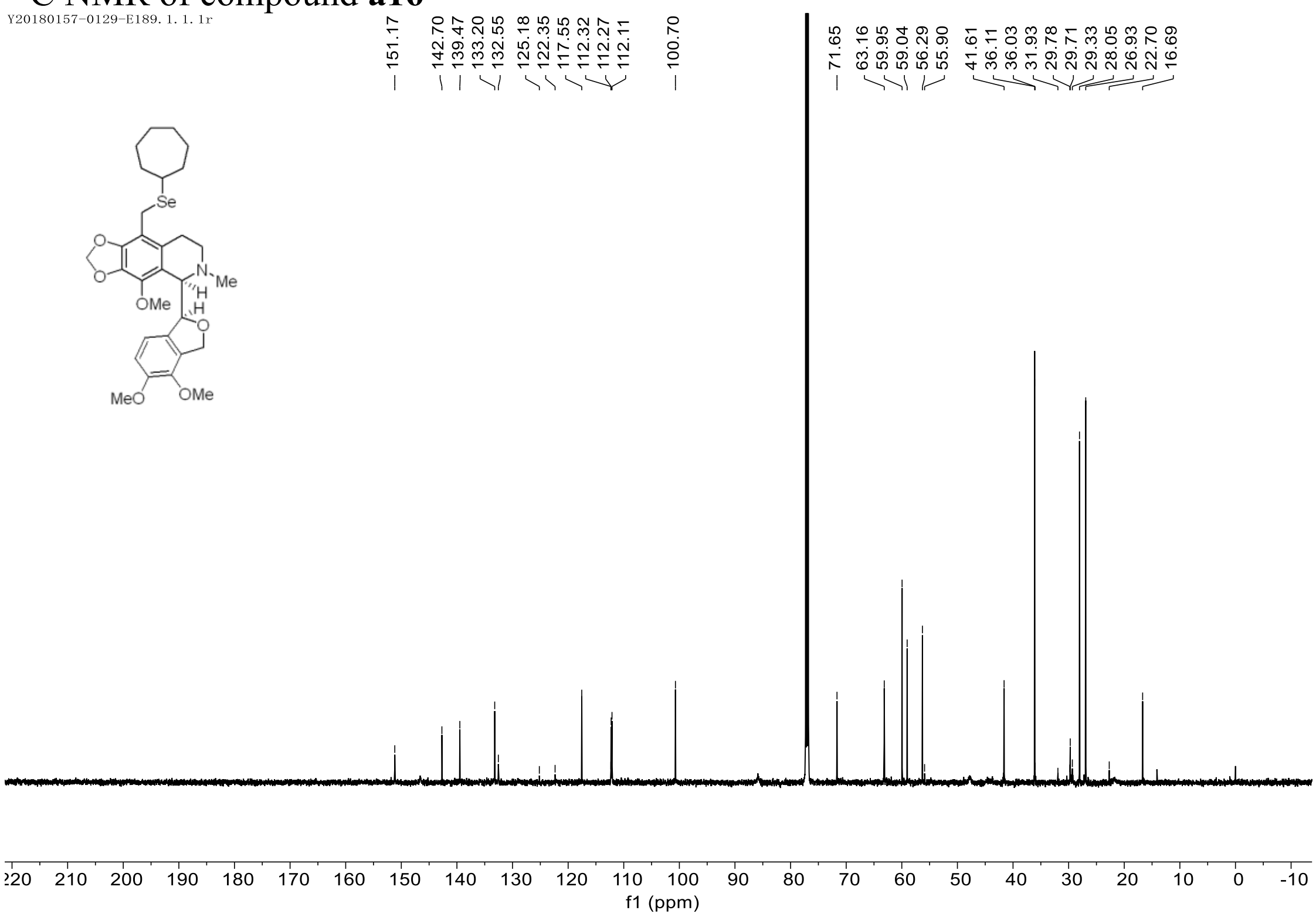

^{1}H NMR of compound **17**

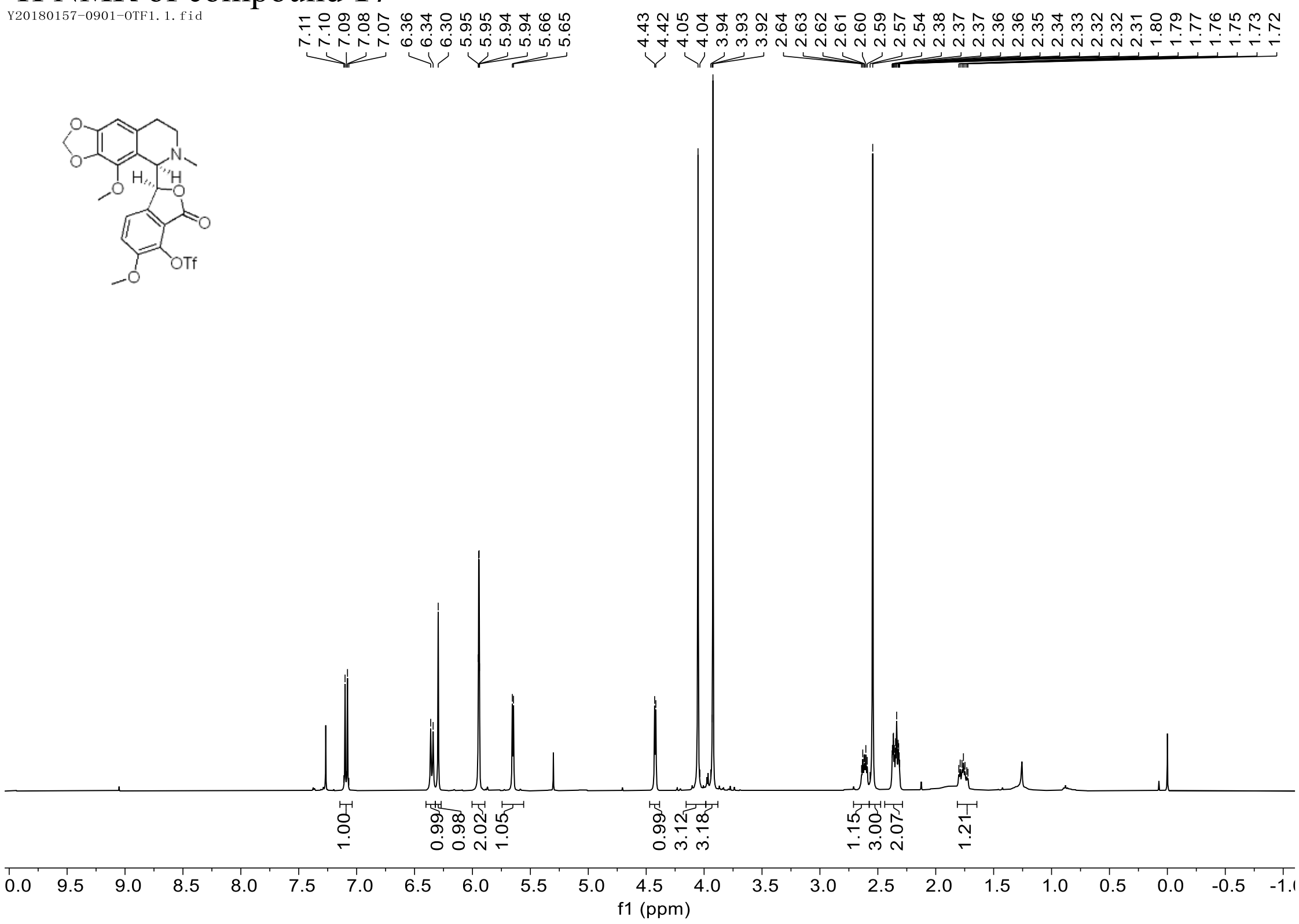

^{1}H NMR of compound **18**

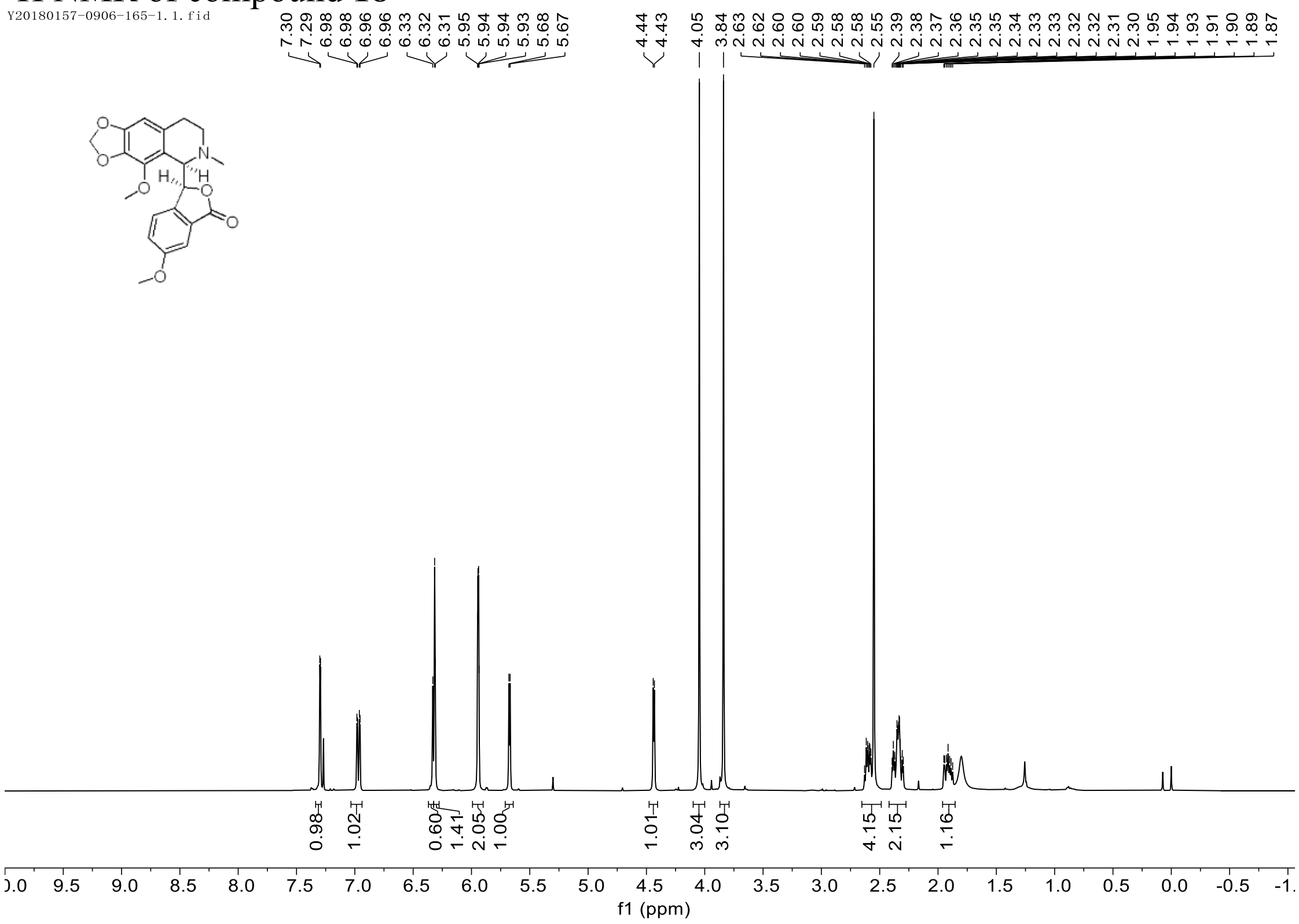

^{1}H NMR of compound **a17**

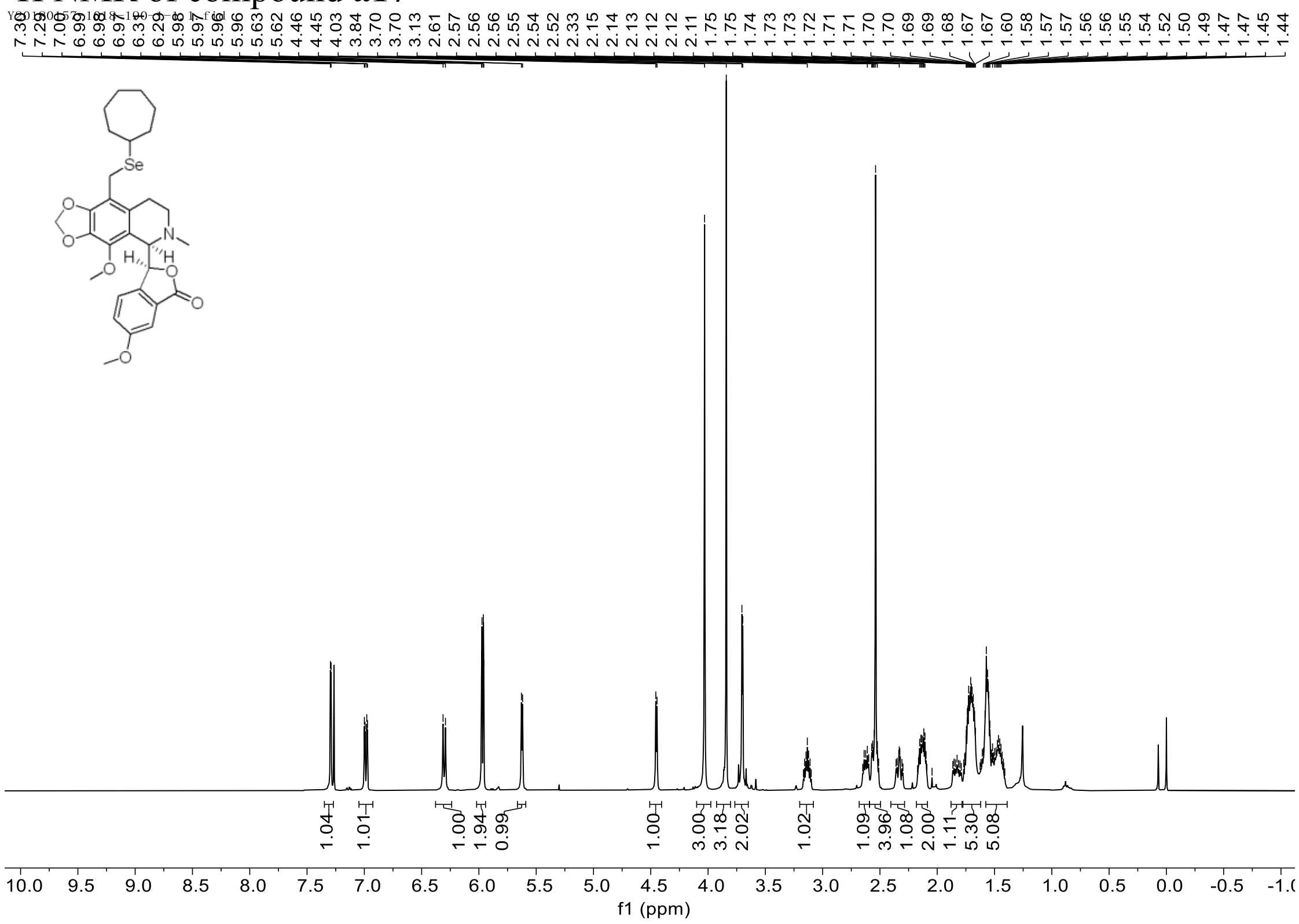

^{13}C NMR of compound **a17**

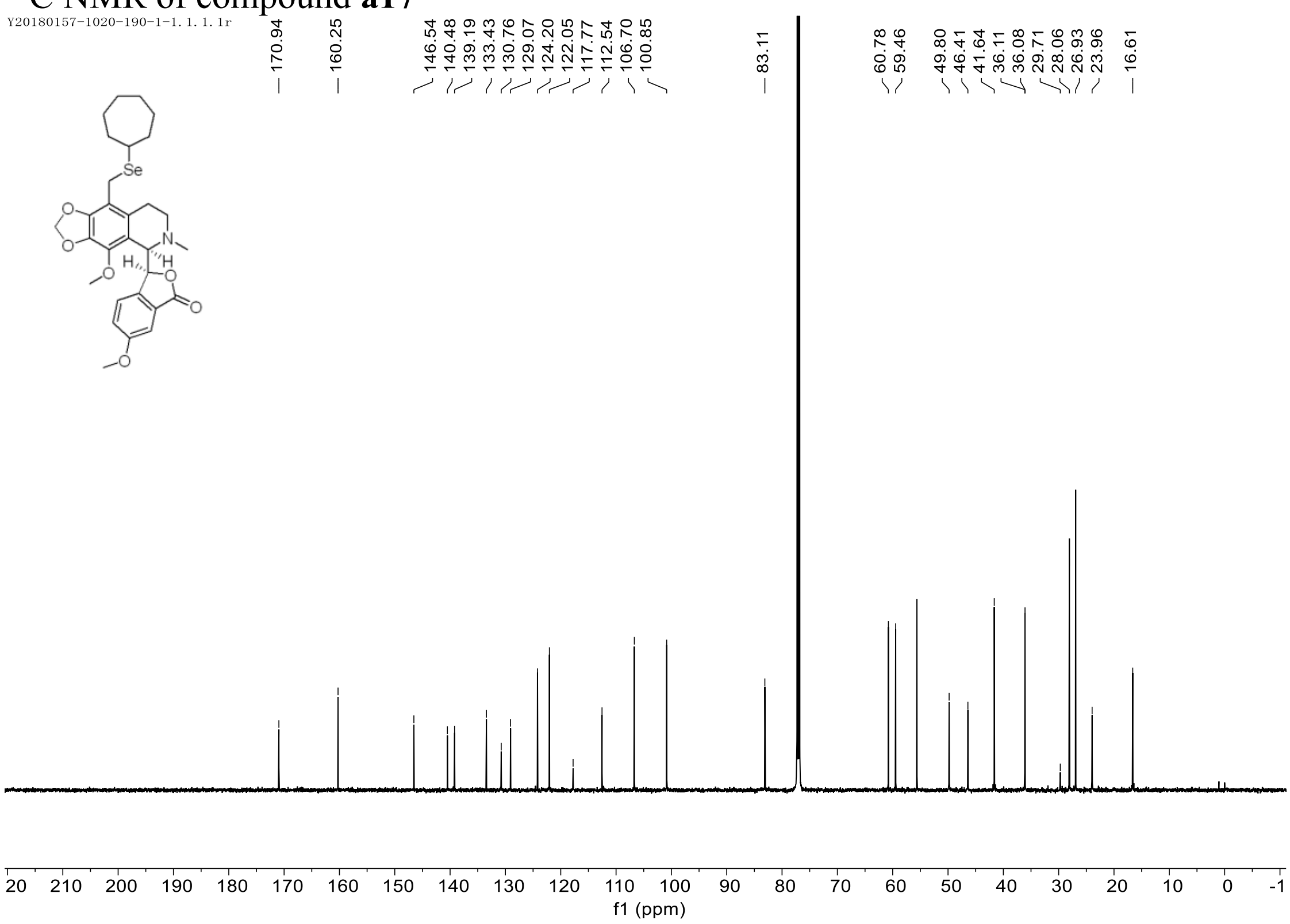

^{1}H NMR of compound **a18**

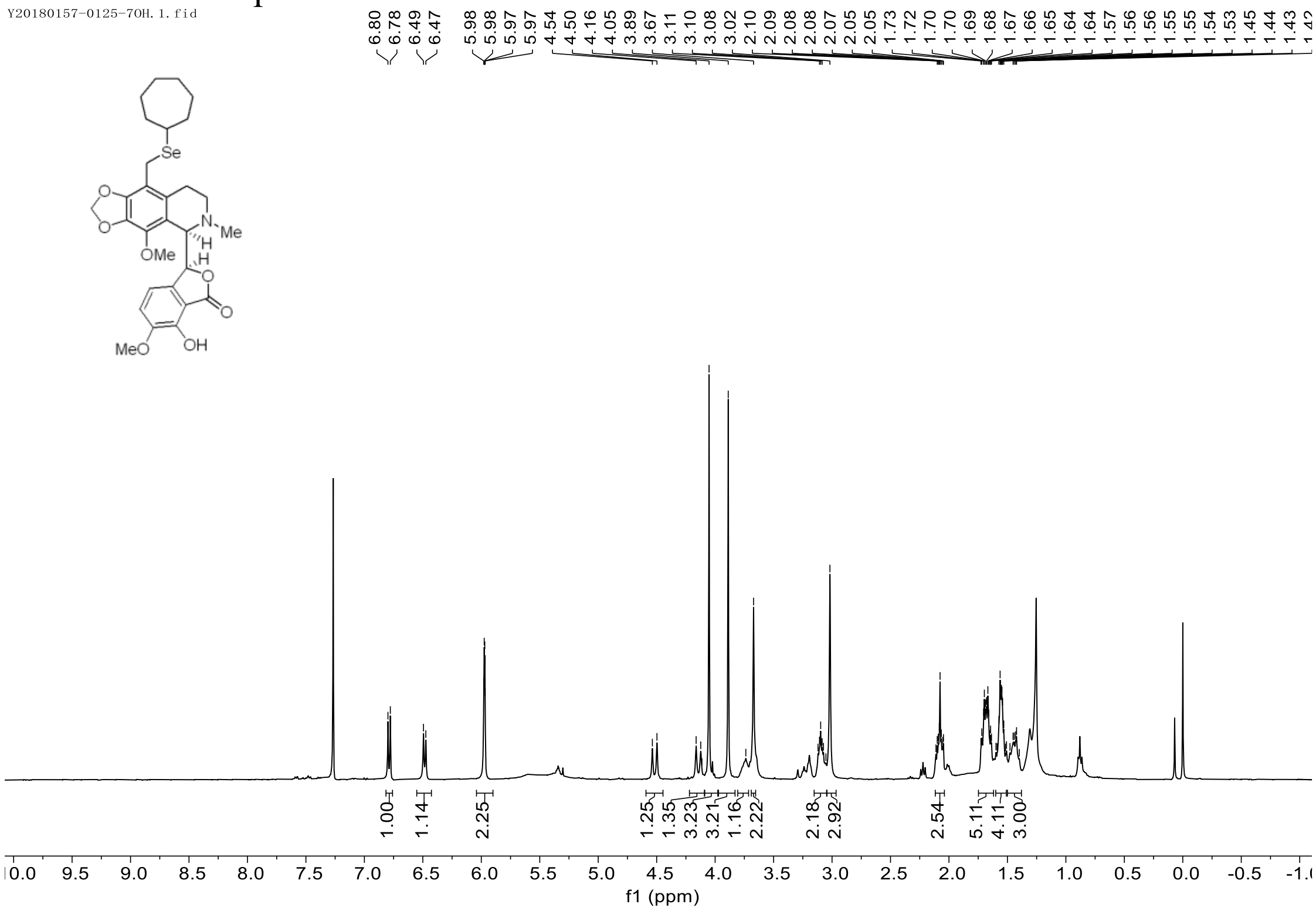

^{13}C NMR of compound **a18**

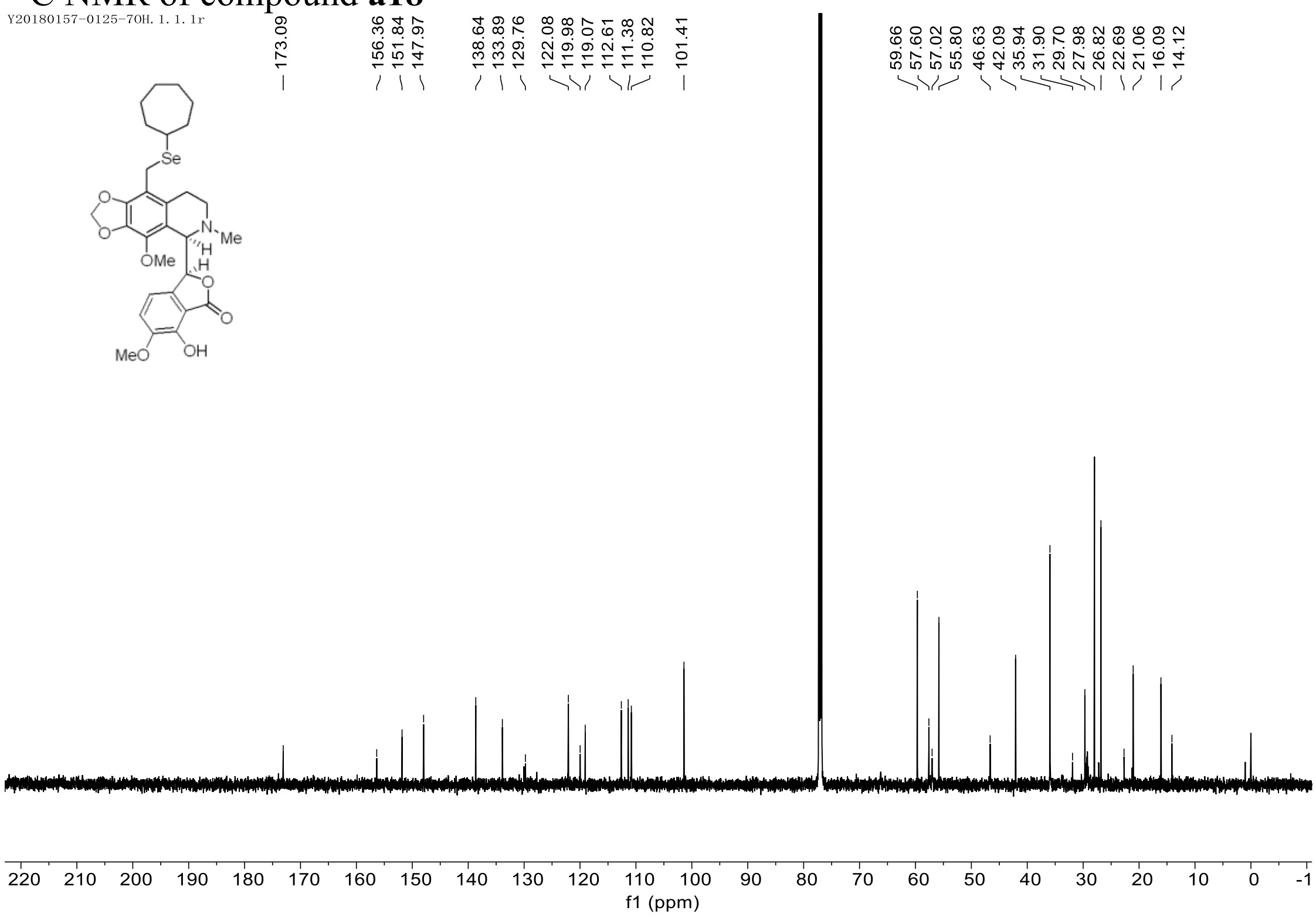

^{1}H NMR of compound **a19**

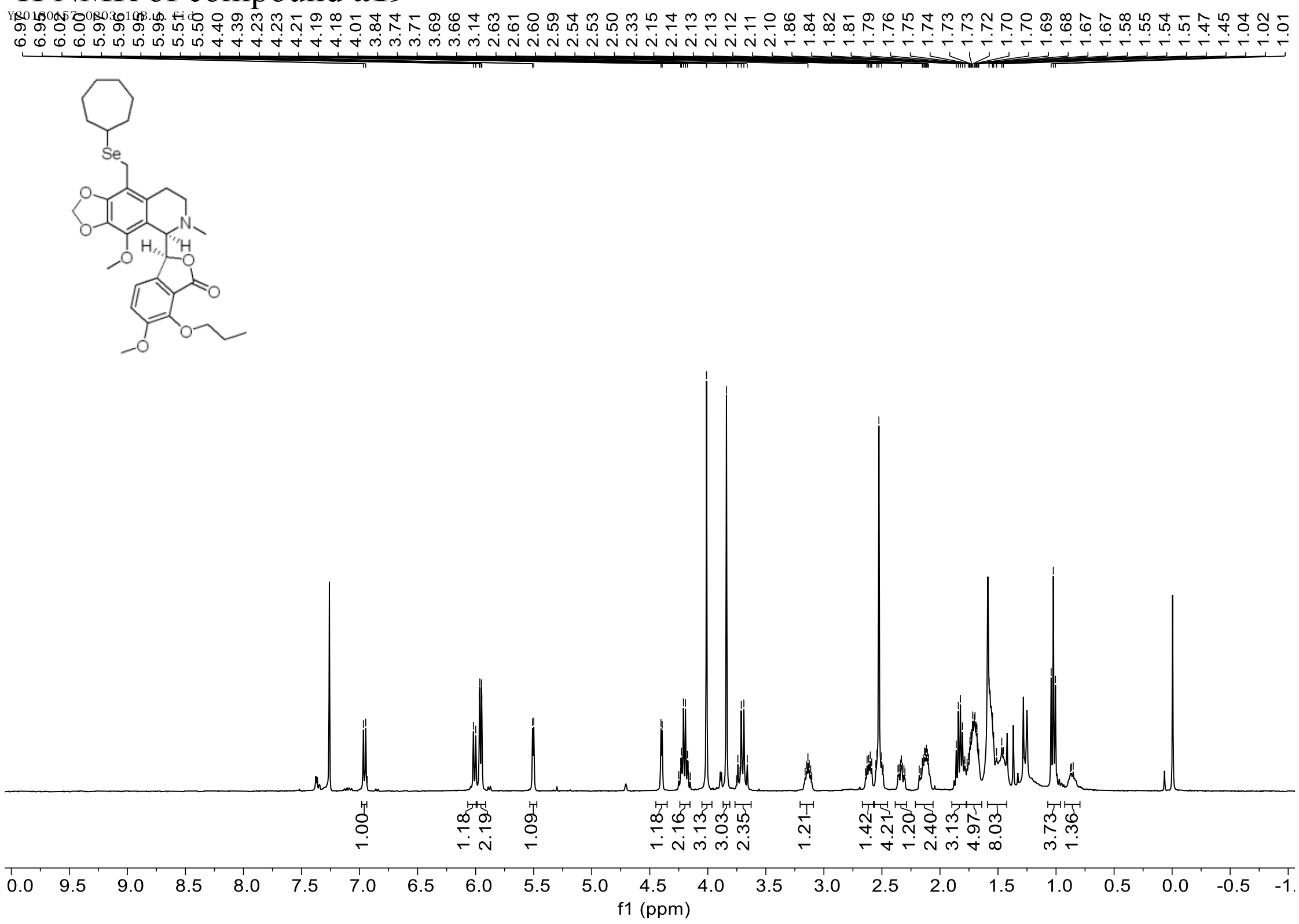

^{1}H NMR of compound **a20**

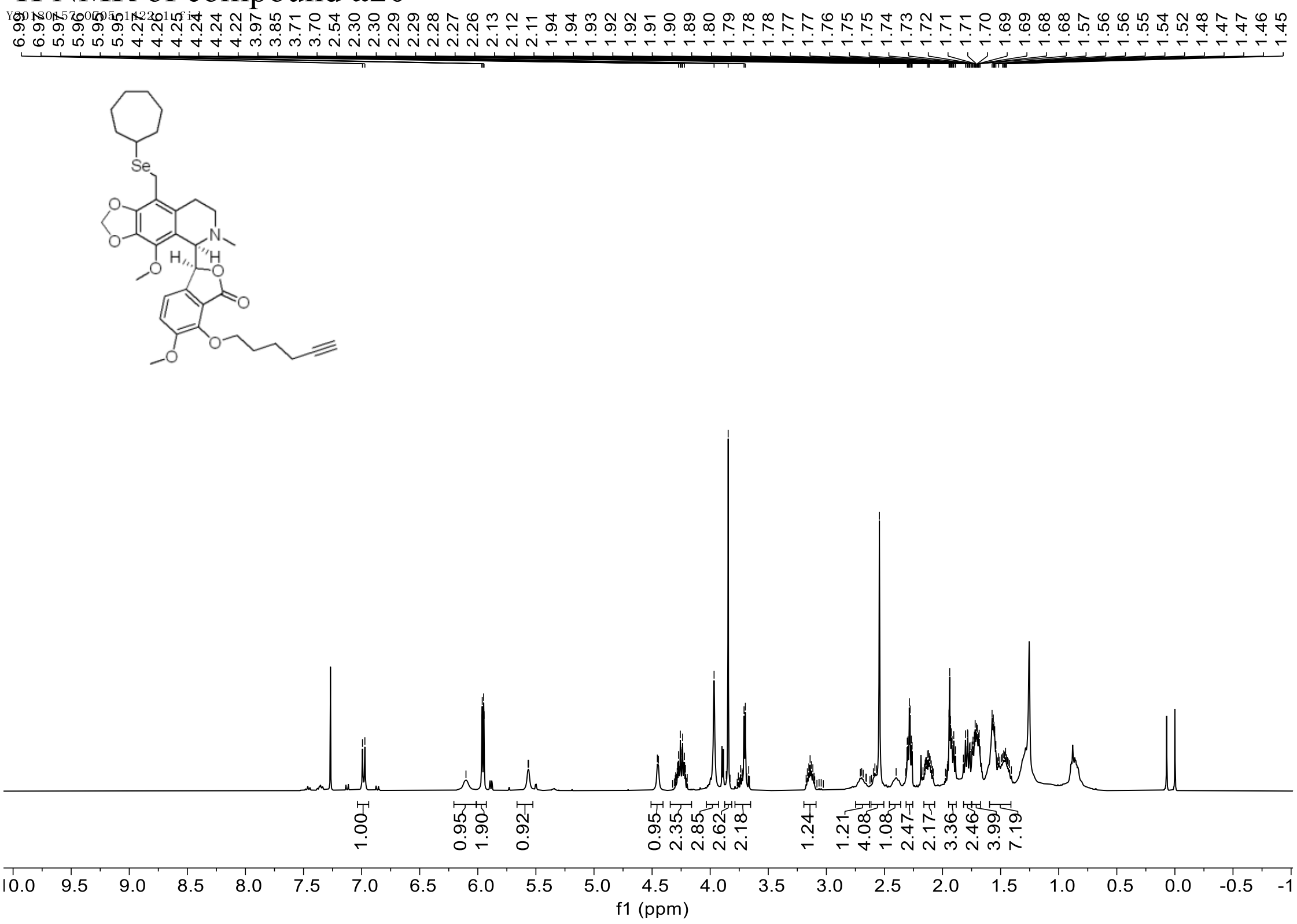

^{1}H NMR of compound **a21**

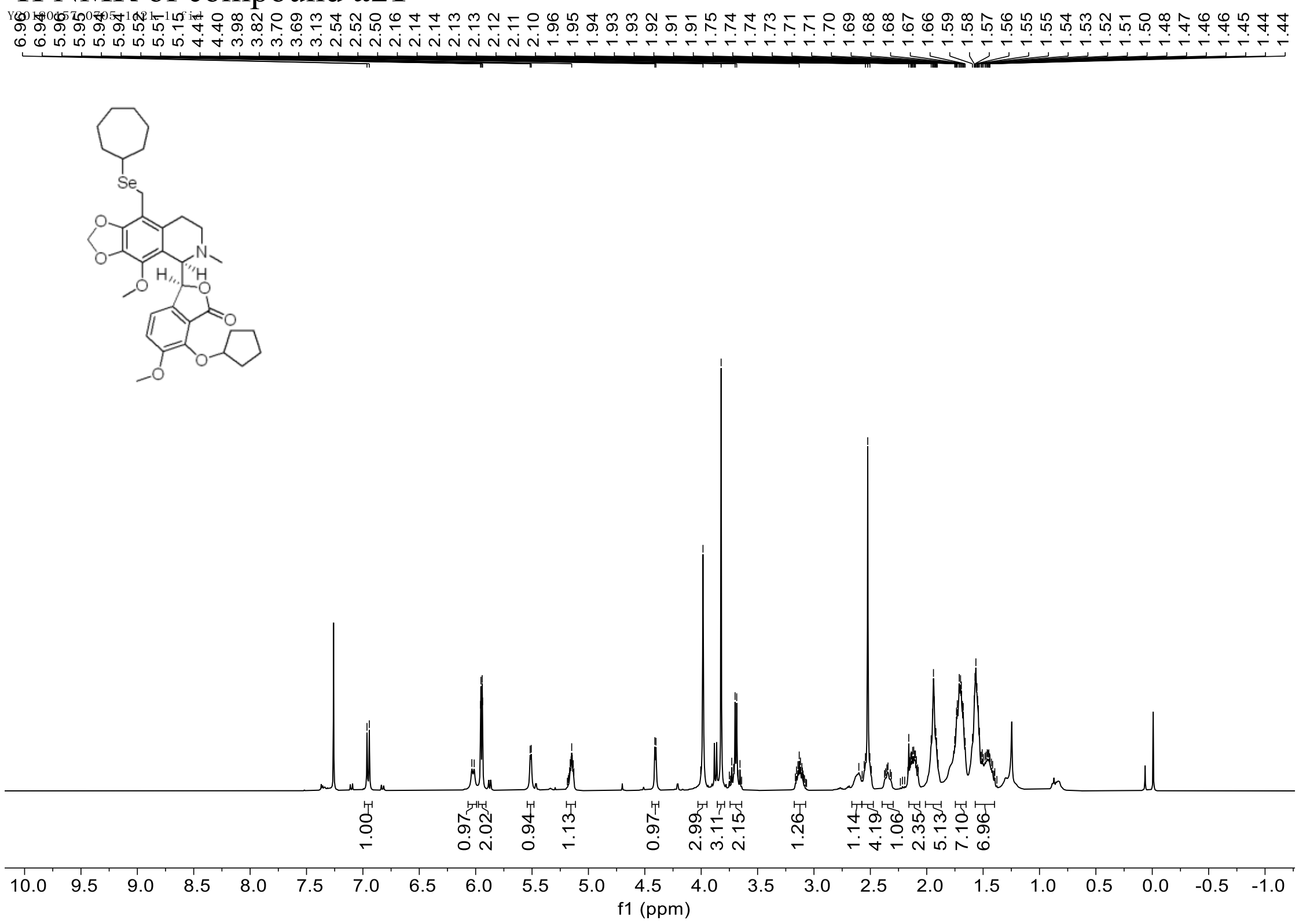

^{1}H NMR of compound **a22**

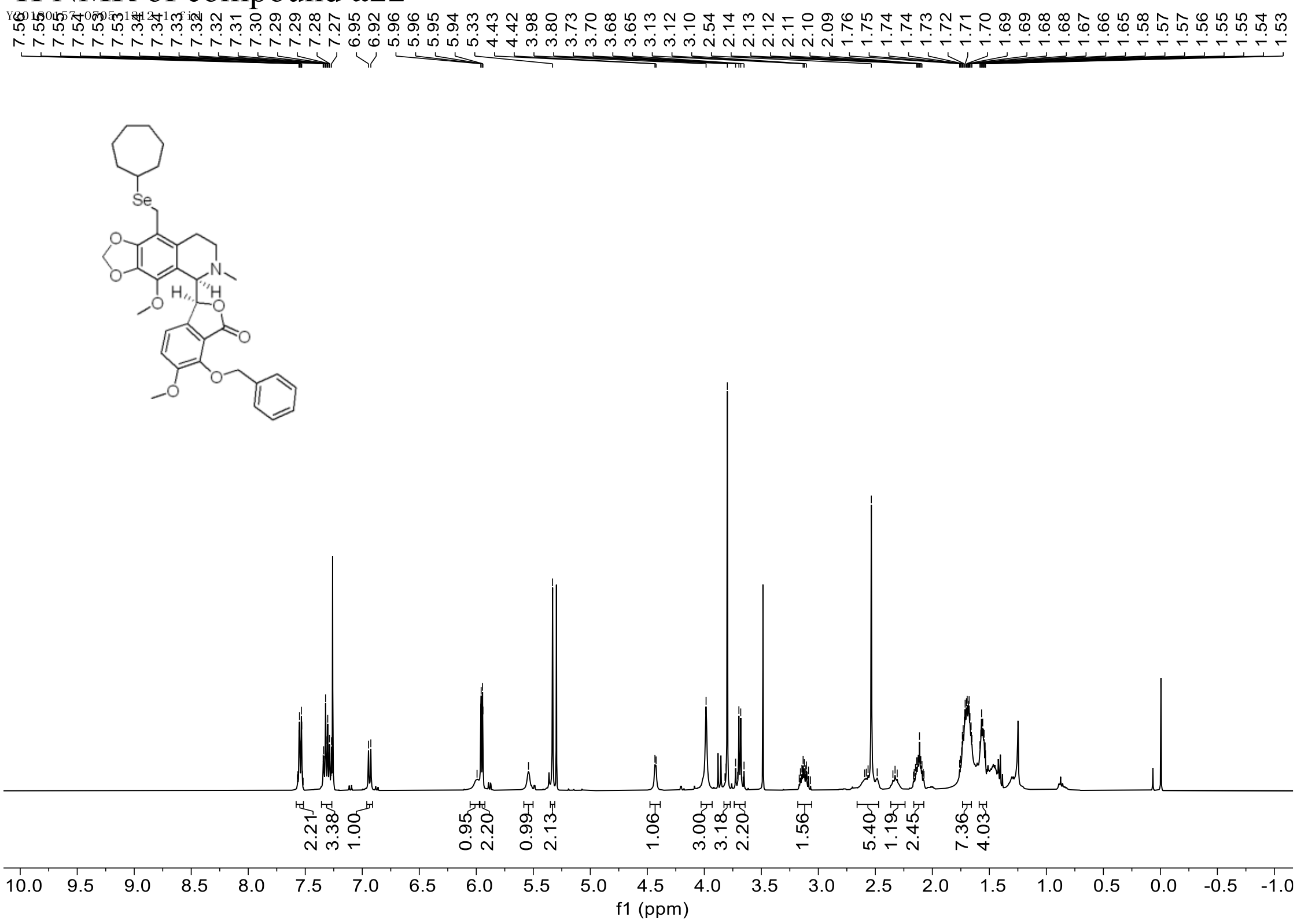

^{1}H NMR of compound **a23**

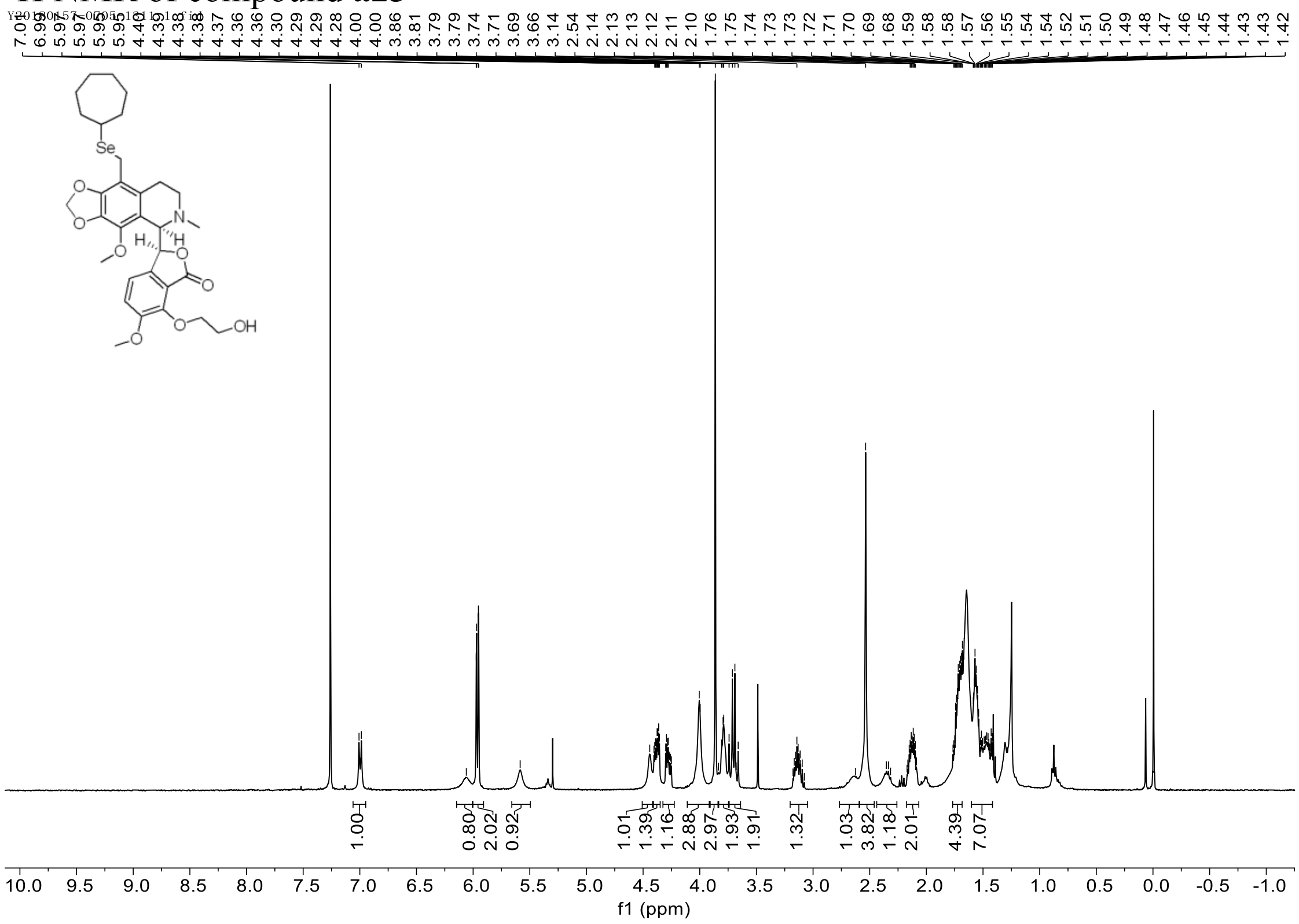

^{1}H NMR of compound **a24**

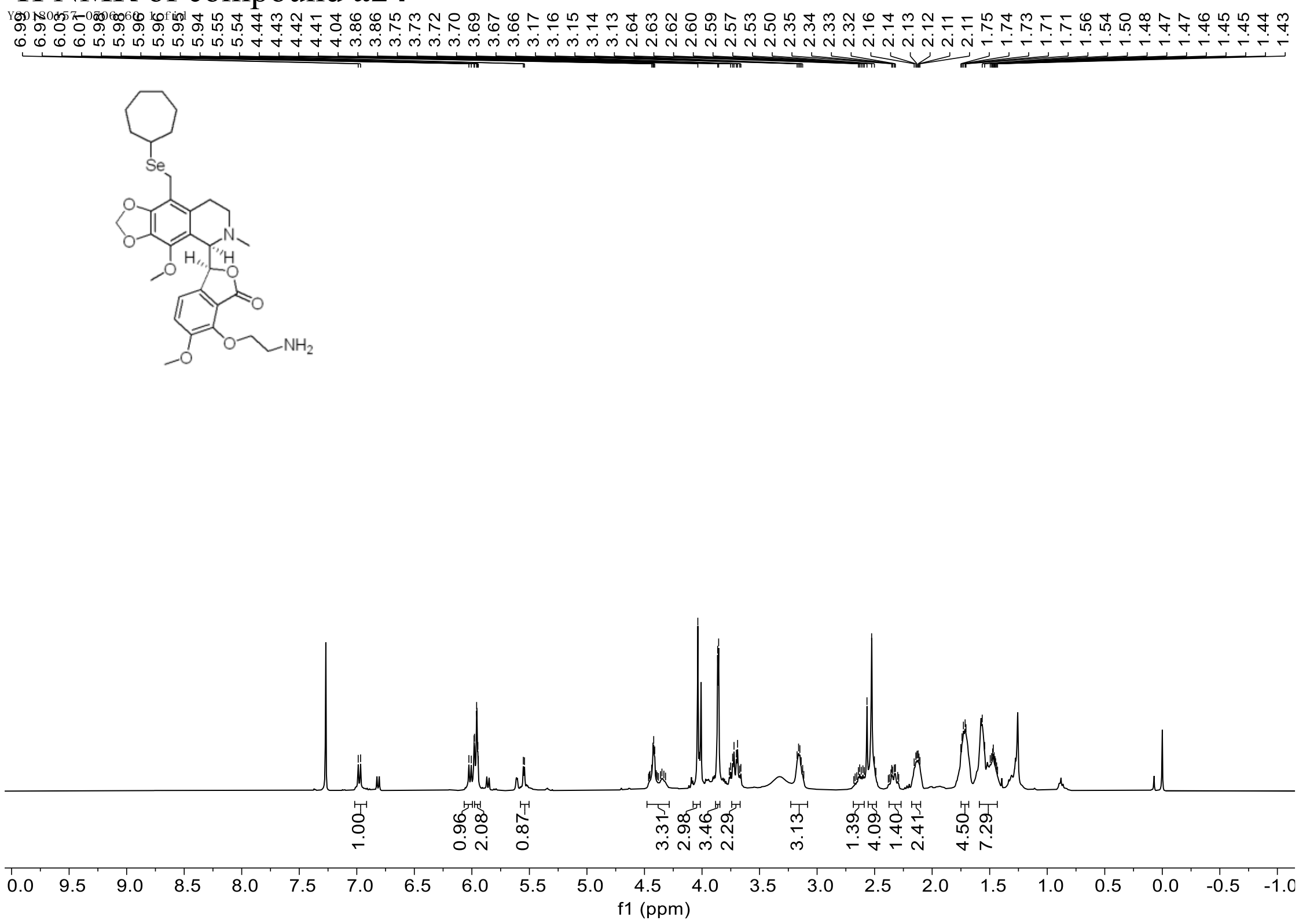

^{13}C NMR of compound **a24**

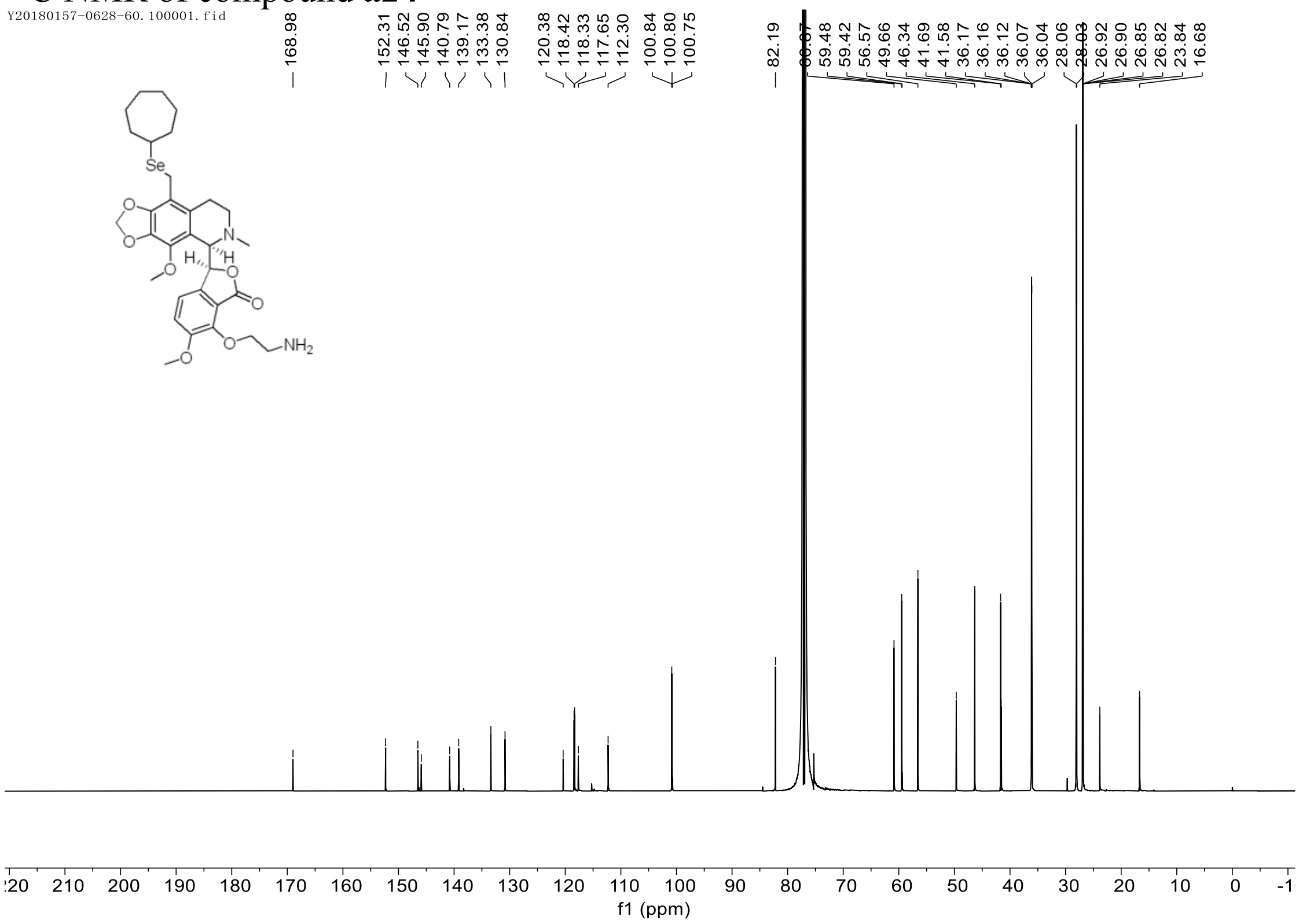

^{1}H NMR of compound **S-ES428**

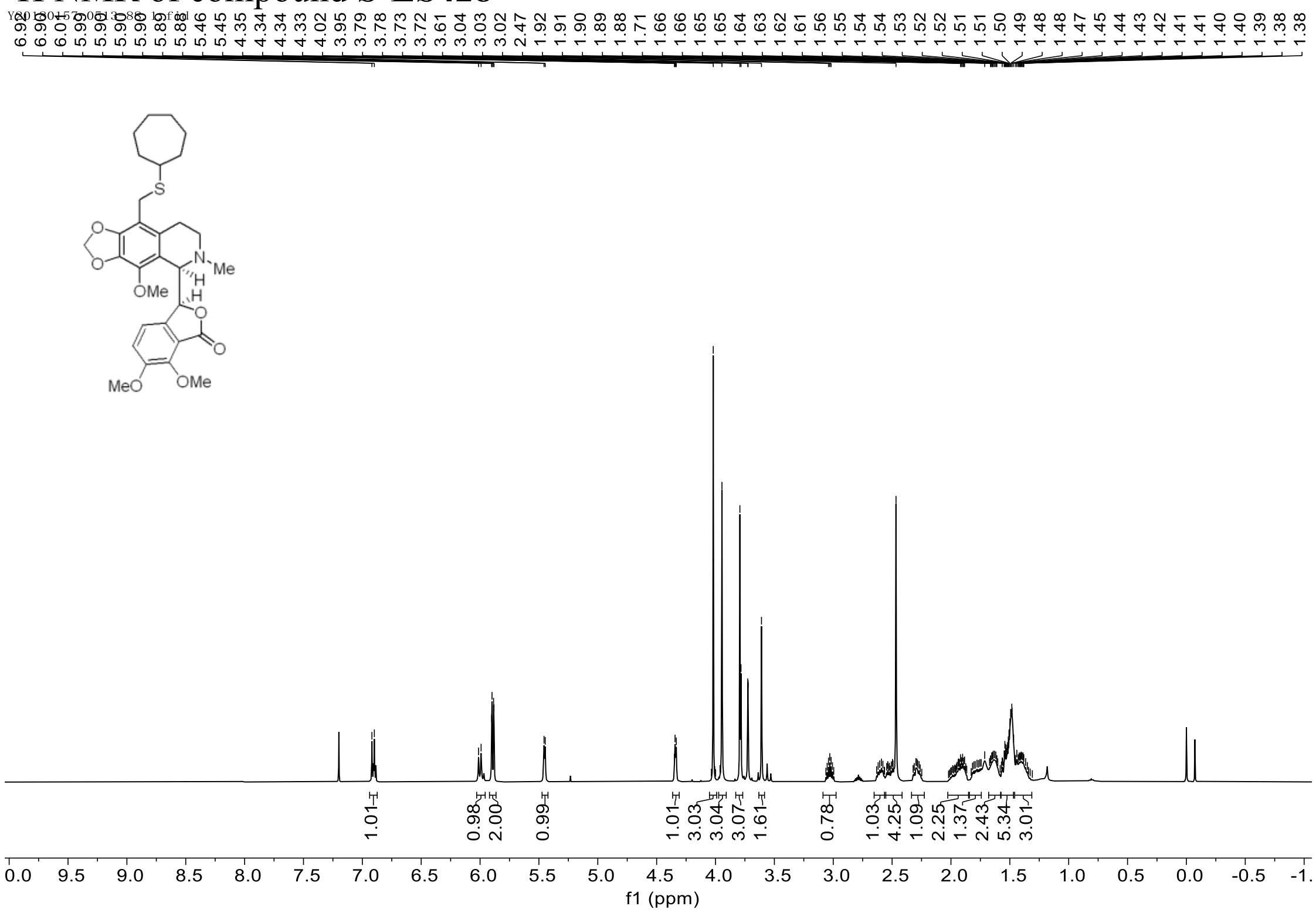

^{13}C NMR of compound **S-ES428**

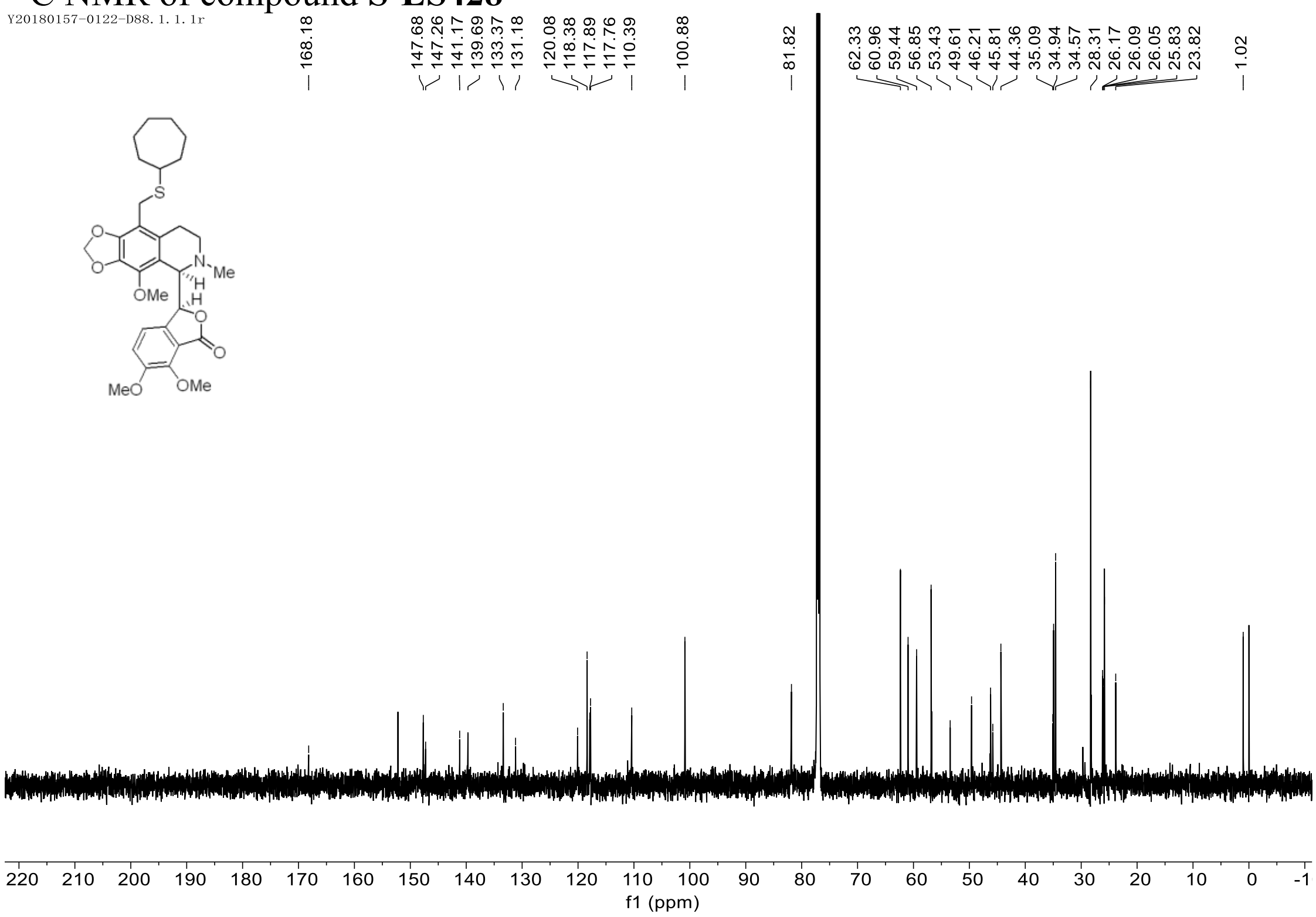

^{1}H NMR of compound **O-ES428**

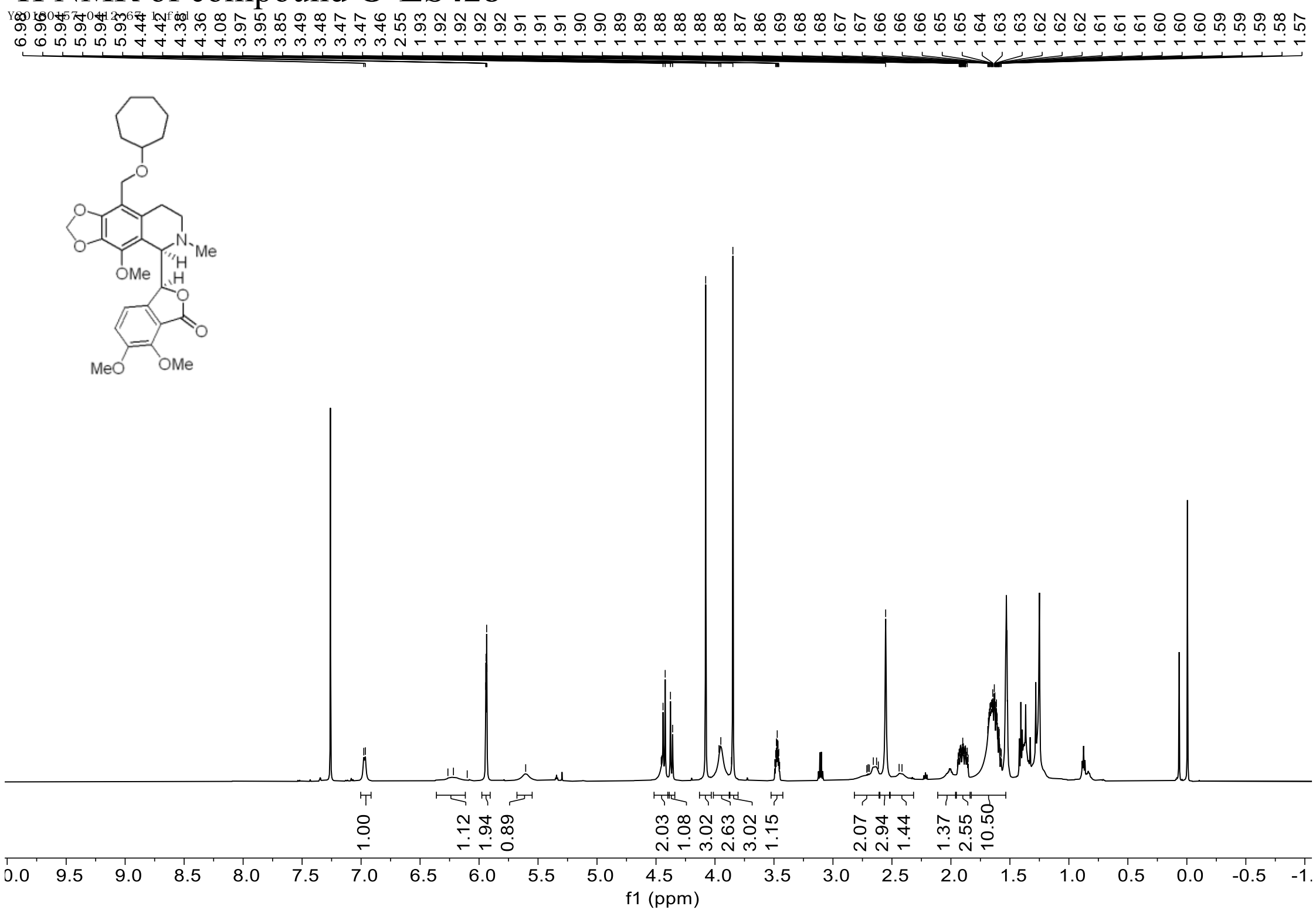

^{13}C NMR of compound **O-ES428**

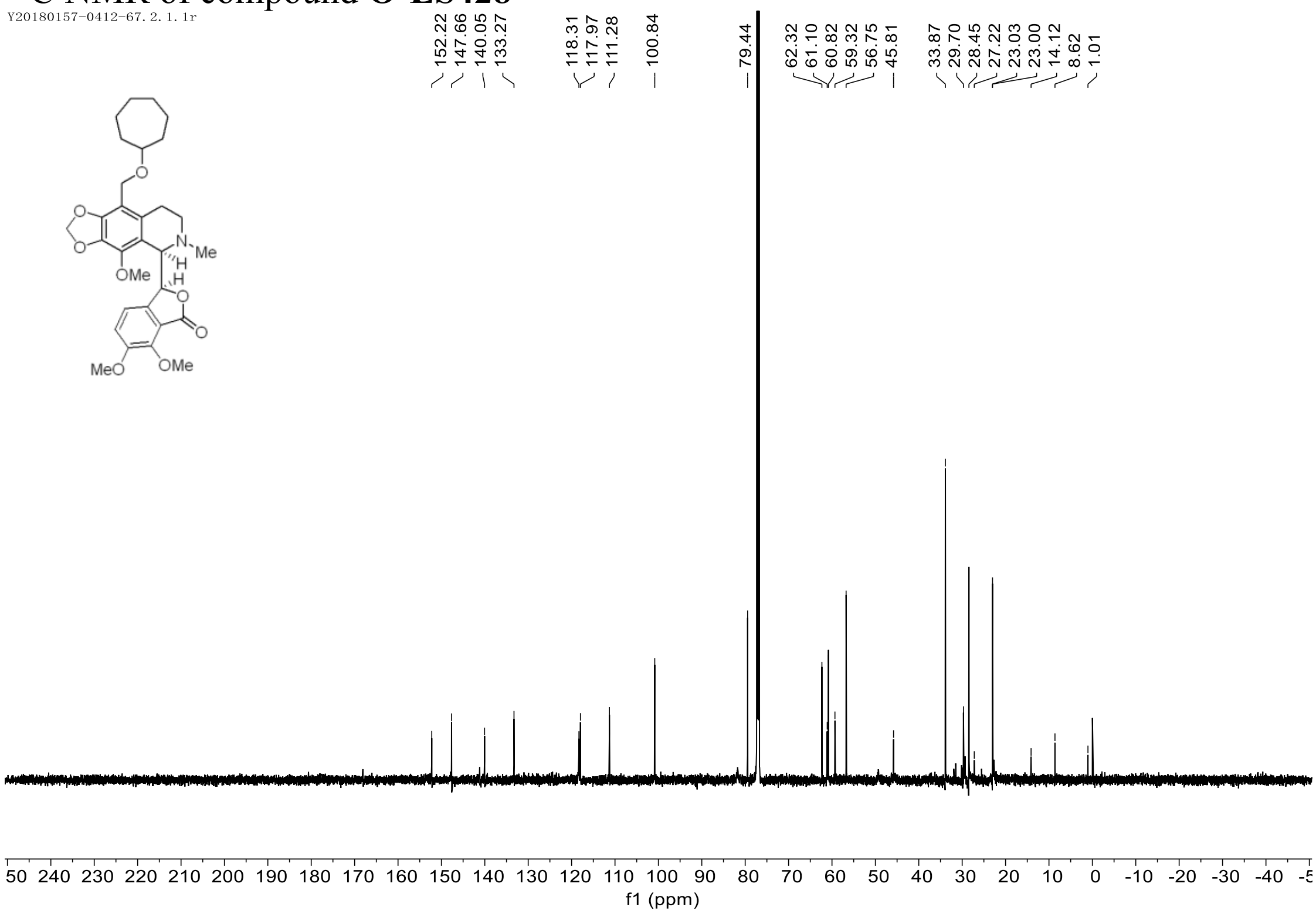

^{1}H NMR of compound **C-ES428**

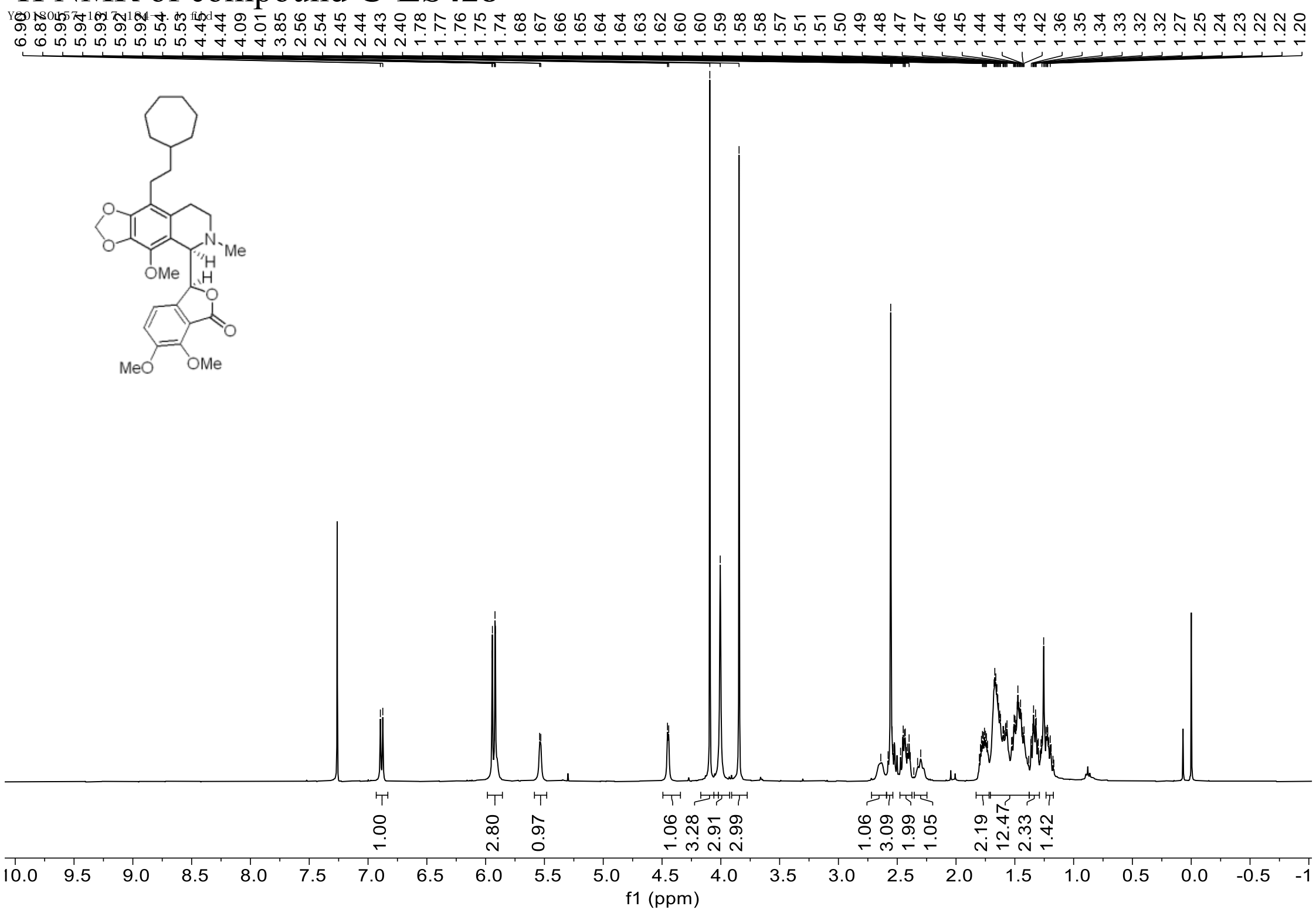

^{13}C NMR of compound **C-ES428**

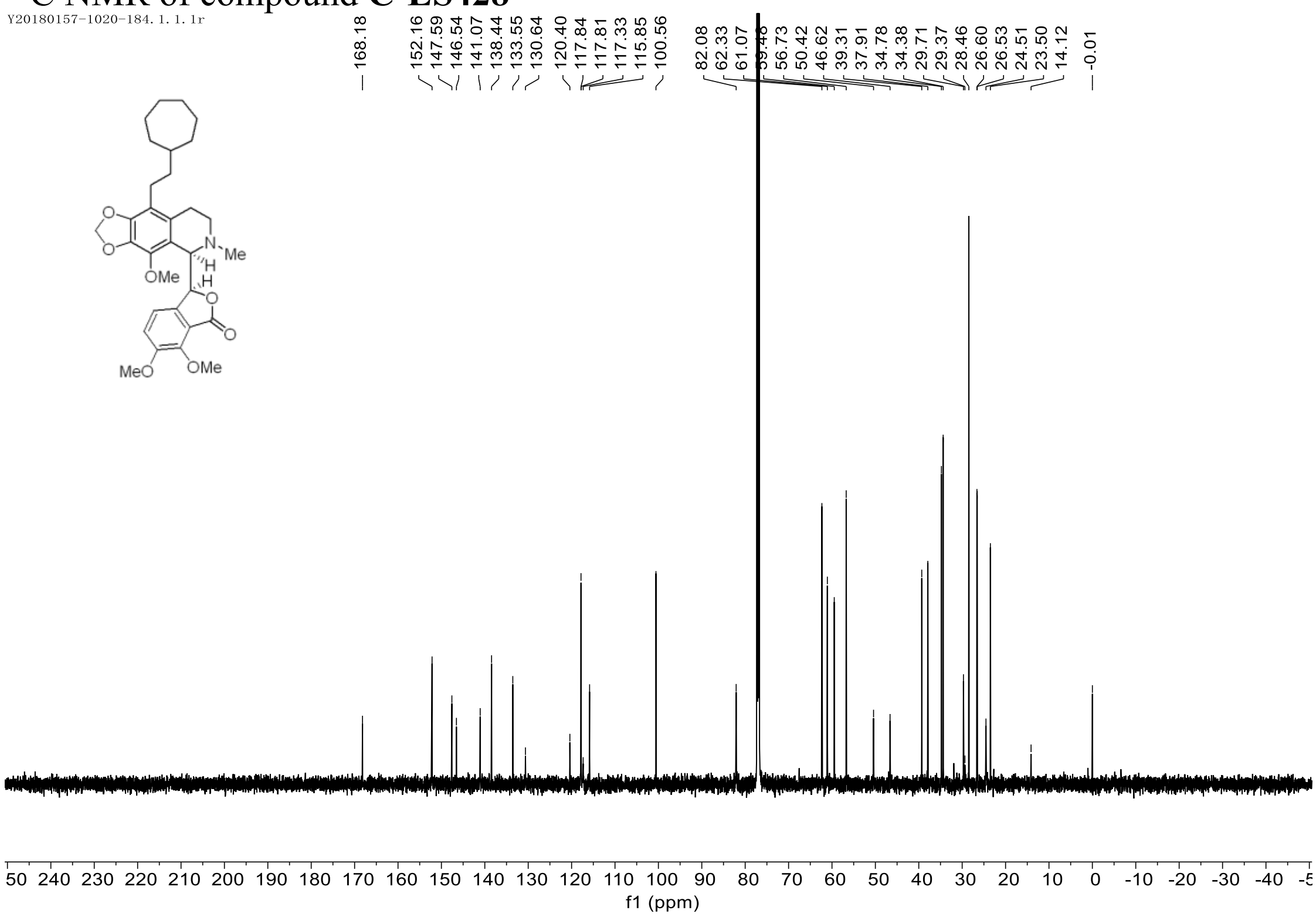

^{1}H NMR of compound **ES428-PAP-probe**

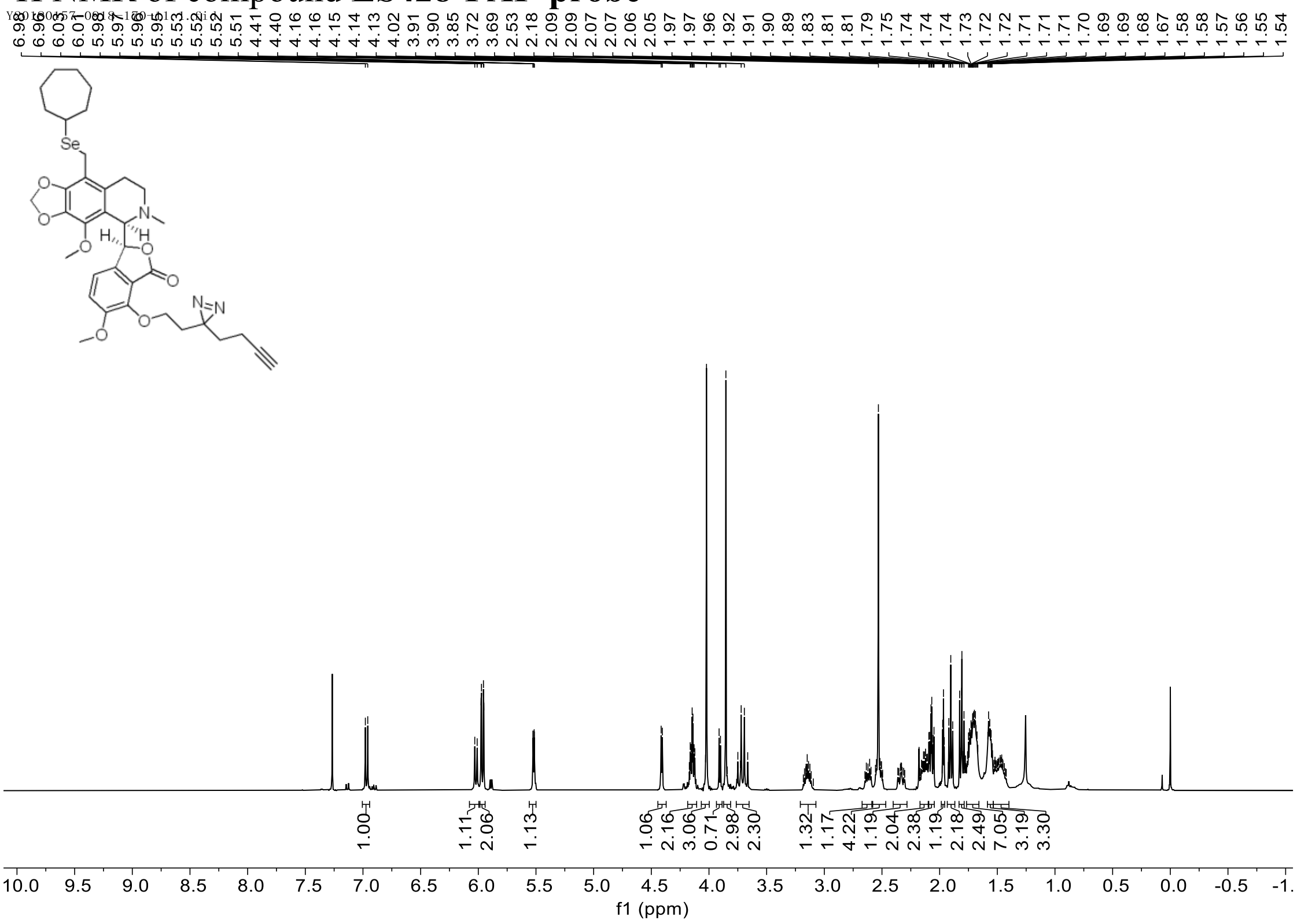

^{13}C NMR of compound **ES428-PAP-probe**

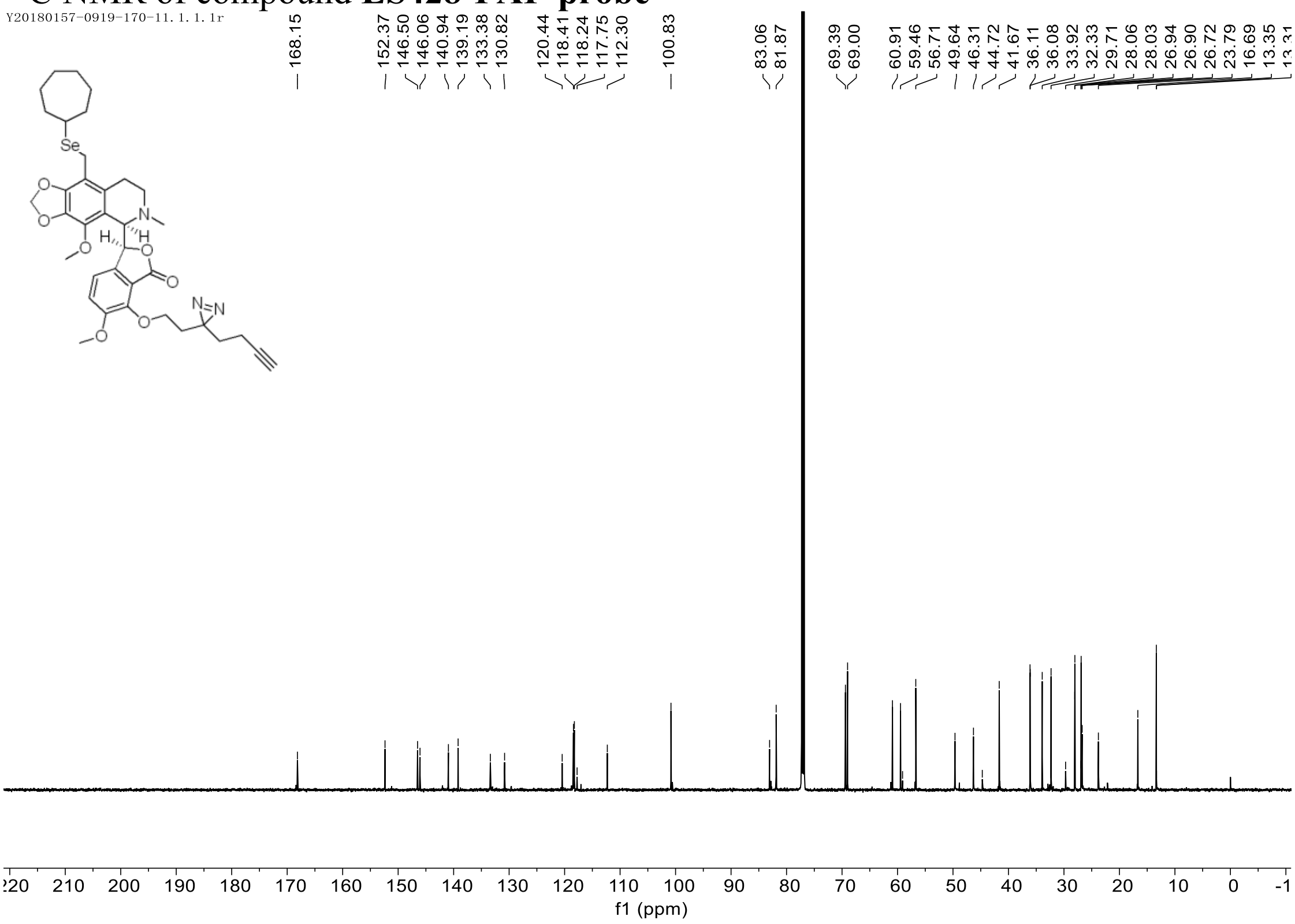

^{1}H NMR of compound **Biotin-ES428-PAP**

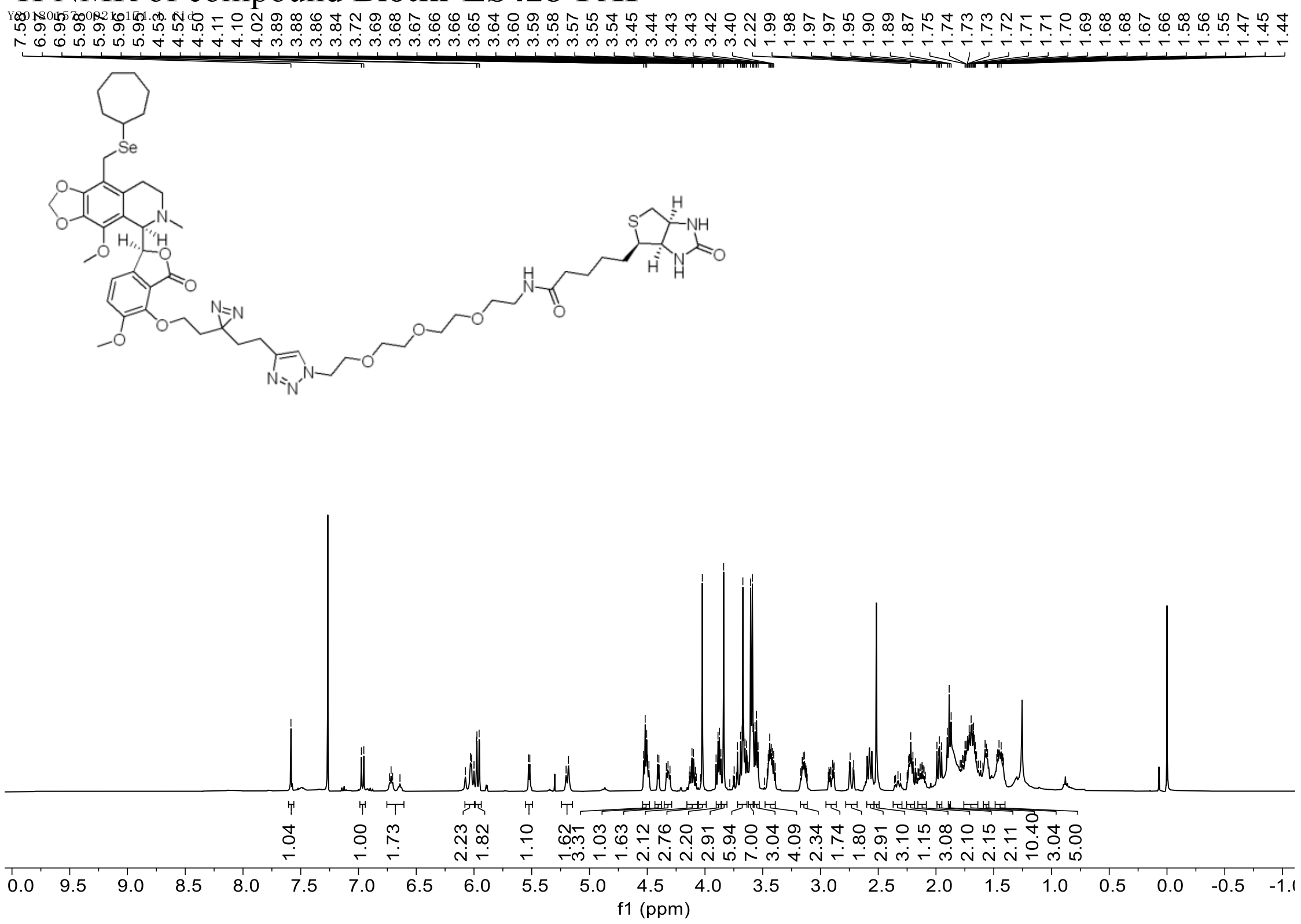

^{13}C NMR of compound **Biotin-ES428-PAP**

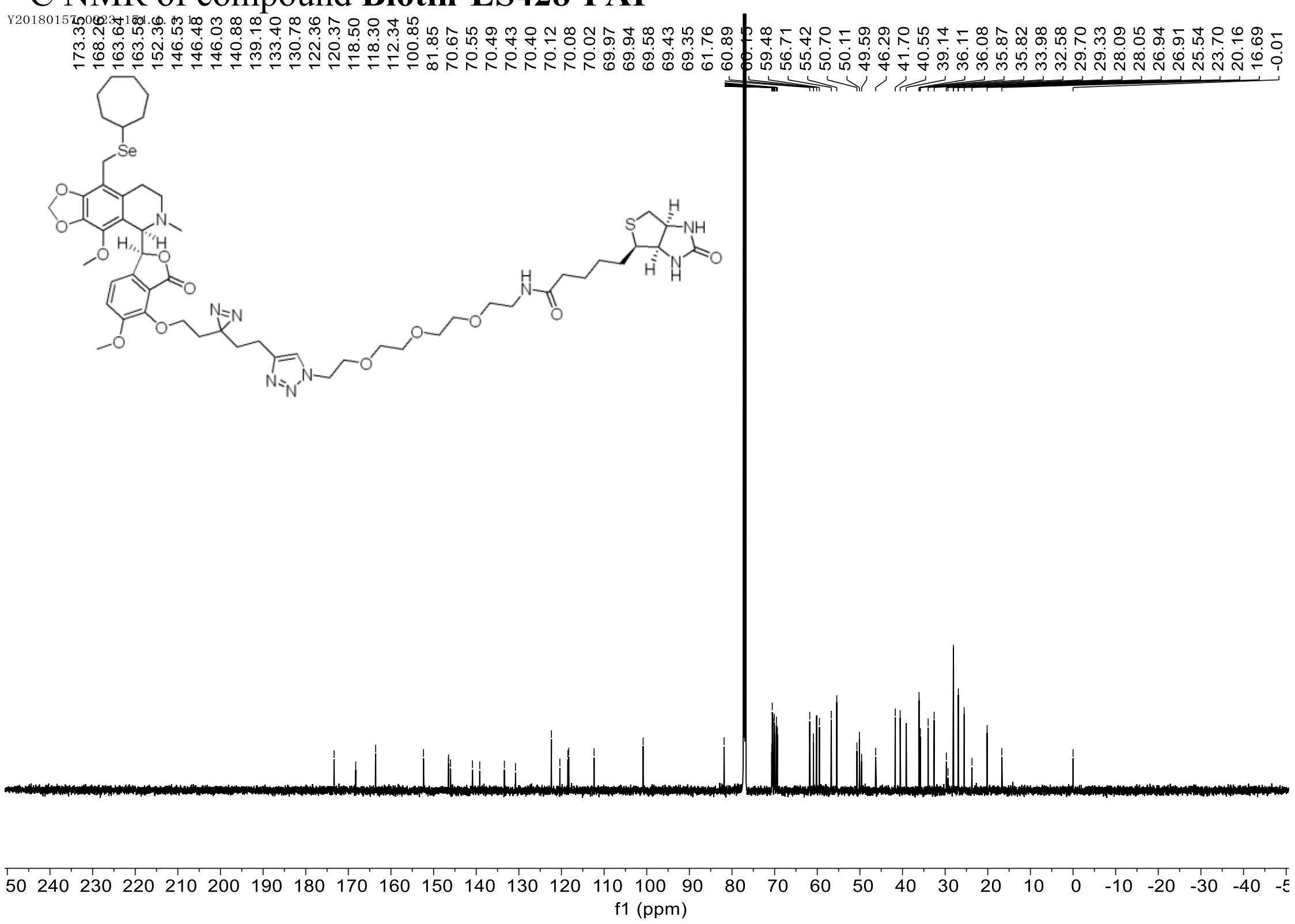

References

1 Sykes DB, Kfoury YS, Mercier FE, Wawer MJ, Law JM, Haynes MK, et al. Inhibition of dihydroorotate dehydrogenase overcomes differentiation blockade in acute myeloid leukemia. *Cell* 2016;**167**:171-86.e15.

2 Fang J, Uchiumi T, Yagi M, Matsumoto S, Amamoto R, Takazaki S, et al. Dihydro-orotate dehydrogenase is physically associated with the respiratory complex and its loss leads to mitochondrial dysfunction. *Biosci Rep* 2013;**33**:e00021.

3 Jumper J, Evans R, Pritzel A, Green T, Figurnov M, Ronneberger O, et al. Highly accurate protein structure prediction with AlphaFold. *Nature* 2021;**596**:583-9.

4 Schrödinger, L. Schrödinger Release 2022-1 2022-1 edn. 2022; **Vol. 2022**.

5 Sastry GM, Adzhigirey M, Day T, Annabhimoju R, Sherman W. Protein and ligand preparation: parameters, protocols, and influence on virtual screening enrichments. *J Comput Aided Mol Des* 2013;**27**:221-34.

6 Halgren TA. Identifying and characterizing binding sites and assessing druggability. *J Chem Inf Model* 2009;**49**:377-89.

7 Greenwood JR, Calkins D, Sullivan AP, Shelley JC. Towards the comprehensive, rapid, and accurate prediction of the favorable tautomeric states of drug-like molecules in aqueous solution. *J Comput Aided Mol Des* 2010;**24**:591-604.

8 Yang Y, Yao K, Repasky MP, Leswing K, Abel R, Shoichet BK, et al. Efficient exploration of chemical space with docking and deep learning. *J Chem Theory Comput* 2021;**17**:7106-19.

9 van Zundert GCP, Moriarty NW, Sobolev OV, Adams PD, Borrelli KW. Macromolecular refinement of X-ray and cryoelectron microscopy structures with Phenix/OPLS3e for improved structure and ligand quality. *Structure* 2021;**29**:913-21.e4.

10 Jacobson MP, Pincus DL, Rapp CS, Day TJ, Honig B, Shaw DE, et al. A hierarchical approach to all-atom protein loop prediction. *Proteins* 2004;**55**:351-67.

11 Lomize MA, Pogozheva ID, Joo H, Mosberg HI, Lomize AL. OPM database and PPM web server: resources for positioning of proteins in membranes. *Nucleic Acids Res* 2012;**40**:D370-6.

12 Schrodinger L. The PyMOL Molecular Graphics System. 2015.

13 Wang L, Wang S, Yang H, Li S, Wang X, Zhou Y, et al. Conformational space profiling enhances generic molecular representation for AI-powered ligand-based drug discovery. *Adv Sci (Weinh)* 2024;**11**:e2403998.

14 DeBono AJ, Xie JH, Ventura S, Pouton CW, Capuano B, Scammells PJ. Synthesis and biological evaluation of N-substituted noscapine analogues. *ChemMedChem* 2012;**7**:2122-33.

15 Anderson JT, Ting AE, Boozer S, Brunden KR, Danzig J, Dent T, et al. Discovery of S-phase arresting agents derived from noscapine. *J Med Chem* 2005;**48**:2756-8.

16 Anderson JT, Ting AE, Boozer S, Brunden KR, Crumrine C, Danzig J, et al. Identification of novel and improved antimitotic agents derived from noscapine. *J Med Chem* 2005;**48**:7096-8.

17 Zhou J, Gupta K, Aggarwal S, Aneja R, Chandra R, Panda D, et al. Brominated derivatives of noscapine are potent microtubule-interfering agents that perturb mitosis and inhibit cell proliferation. *Mol Pharmacol* 2003;**63**:799-807.
